# Cu-catalyzed asymmetric addition of alcohols to *β*,*γ*-alkynyl-*α*-imino esters for the construction of linear chiral *N*,*O*-ketals

**DOI:** 10.1038/s41467-022-28002-7

**Published:** 2022-01-20

**Authors:** Cheng Sheng, Zheng Ling, Yicong Luo, Wanbin Zhang

**Affiliations:** Shanghai Key Laboratory for Molecular Engineering of Chiral Drugs, Frontiers Science Center for Transformative Molecules, School of Chemistry and Chemical Engineering, Shanghai Jiao Tong University, 800 Dongchuan Road, Shanghai, 200240 China

**Keywords:** Asymmetric catalysis, Asymmetric synthesis, Stereochemistry

## Abstract

*N*,*O*-acetals are part of many synthetic intermediates and important skeletons of numerous natural products and pharmaceutical drugs. The most straightforward method of the synthesis of *N*,*O*-acetals is the enantioselective addition of *O*-nucleophiles to imines. However, using this method for the synthesis of linear chiral *N*,*O*-ketals still remains challenging due to the instability of raw materials under acidic or basic conditions. Herein, we developed a Cu-catalyzed asymmetric addition of alcohols to *β*,*γ*-alkynyl-*α*-imino esters under mild conditions, providing the corresponding linear chiral *N*,*O*-ketals with up to 96% *ee*. The method tolerates some variation in the *β*,*γ*-alkynyl-*α*-imino ester and alcohol scope, including some glucose and natural amino acid derivatives. Computational results indicate that the Boc group of the substrates assist in the extraction of hydrogen atoms from the alcohols to promote the addition reactions. These products could be synthesized on a gram-scale and can be used in several transformations. This asymmetric addition system provides an efficient, mild, gram-scale, and transition-metal-catalyzed synthesis of linear chiral *N*,*O*-ketals.

## Abstract (web summary)

*N,O*-acetals are part of many synthetic intermediates and important skeletons of numerous natural products and pharmaceutical drugs. Here the authors show a Cu-catalyzed asymmetric addition of alcohols to *β*,*γ*-alkynyl-*α*-imino esters, providing the corresponding linear chiral *N,O*-ketals with up to 96% *ee*.

## Introduction

**Fig. 1:**
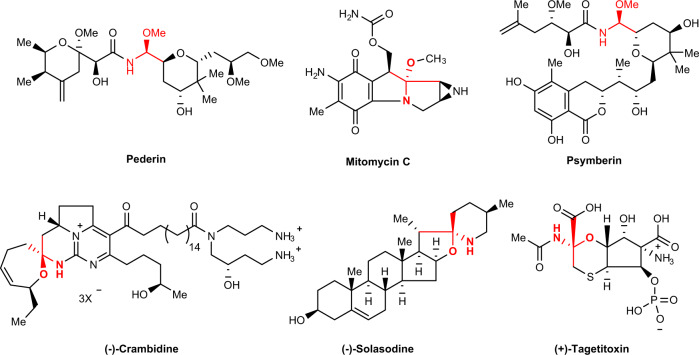
Examples of biologically molecules with *N*,*O*-acetal-structures. Natural products and bioactive molecules containing the chiral *N*,*O*-acetal-structures.

*N*,*O*-acetals (also termed *N*,*O*-aminals) are present in numerous structural motifs, which can be synthetic intermediates and important skeletons of numerous natural products and pharmaceutical drugs^1–9^. Chiral *N*,*O*-acetal subunits exist in a number of intriguing natural products such as Pederin^10^ and (−)-Crambidine^11^ and a considerable number of bioactive molecules like Mitomycin C^12^, (−)-Solasodine^13^, Psymberin^14^, and (+)-Tagetitoxin (Fig. 1)^15^. Importantly, the stereochemistry of this motif is crucial in terms of the biological activity of some anti-cancer candidates^16,17^.

**Fig. 2:**
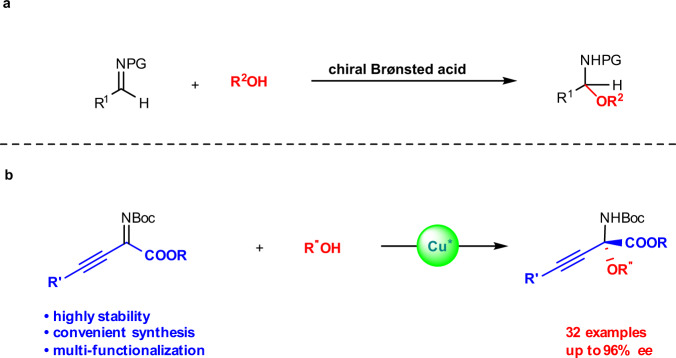
Catalytic asymmetric addition of *O*-nucleophiles to linear imines. **a** Previous work: synthesis of linear chiral *N*,*O*-acetals. **b** This work: synthesis of linear chiral *N*,*O*-ketals.

Even though many synthetic routes for the preparation of chiral *N*,*O*-acetals have been reported^18–24^, the most straightforward method is the enantioselective addition of *O*-nucleophiles to imines^25–34^. Previous reports on the synthesis of linear chiral *N*,*O*-acetals mainly focus on the addition reaction of *O*-nucleophiles to aldimines by a chiral Brønsted acid (Fig. 2a)^25–27^. To the best of our knowledge, there are almost no reports concerning the addition reaction of *O*-nucleophiles to linear ketimines due to the instability of raw materials under acidic or basic conditions and the difficulty controlling the stereoselectivity, even though linear chiral *N*,*O*-ketals are versatile scaffolds for the preparation of potential bioactive compounds^35–38^. Therefore, it is necessary to develop a synthesis of linear chiral *N*,*O*-ketals via the addition reaction of alcohols to linear ketimines with mild reaction conditions.

*β*,*γ*-Alkynyl-*α*-imino esters, as types of highly stable and easy-accessible ketimines, are also recognized as potentially useful precursors for the synthesis of alkynyl or alkenyl *α*-amino acid derivatives due to the convenient functionalization of the ester and alkynyl groups (Fig. 2b)^39–43^. Additionally, the presence of carbonyl groups may allow for easier coordination with a metal atom to control the stereoselectivity. In view of the previous work related to the asymmetric addition reaction of ketimines in our group^44–51^, copper catalysis has already been shown to provide good reactivity and enantioselectivity^45,47^. Herein, we describe an efficient protocol for the preparation of linear chiral *N*,*O*-ketals using a direct Cu-catalyzed asymmetric addition of alcohols to *β*,*γ*-alkynyl-*α*-imino esters (Fig. 2b).

## Results

### Investigation of reaction conditions

At the beginning of this study, *β*,*γ*-alkynyl-*α*-imino ester **1a** and ethanol **2a** were selected as the model substrates to investigate the asymmetric addition of *O*-nucleophiles, as shown in Table 1. If only Cu(OTf)_2_ was employed, the corresponding product **3a** was generated in only a trace amount due to the hydrolysis of **1a** (Table 1, entry 1). To our delight, a racemate of **3a** was obtained in good yield when 4 Å MS was added (entry 2). Subsequently, the chiral ligands were investigated, and bis(oxazoline) (Box) ligands were found to perform better than other ligands (see Supplementary Table 1). Changing the substituents on the oxazoline rings of the Box ligands (**L1**-**L5**) showed that phenyl-substituted groups (**L5**) afforded better results (entries 3–7). Subsequently, the effect of substituents at the carbon atom connecting the two oxazoline rings was investigated. The substituent (R) was changed to methyl, allyl and 2-methylbut-2-enyl groups for screening (**L6**-**L8**). As a result, ligand **L8** exhibited the best catalytic efficiency, providing the desired product with 71% *ee* and in 88% isolated yield (entries 8–10). Next, a variety of copper sources were examined (entries 11–13). Interestingly, copper catalysts with weakly binding anions performed better compared with those possessing strong binding anions (see Supplementary Table 2). The influence of solvent was subsequently inspected, and to our surprise, the *ee* value of the product improved dramatically when acetone was used as the solvent (entries 14–16). Reducing the temperature to 0 °C gave the desired product in 88% yield and 96% *ee* (entry 17). Finally, decreasing the equivalents of alcohol **2a** improved the product yield slightly (entry 18). The absolute configuration of **3a** was determined by X-ray diffraction.

**Table 1:** Reaction optimization^a^.


Entry	Cu salt	Ligand	Solvent	Additive	Yield %^b^	ee %^c^
1	Cu(OTf)_2_	None	THF	None	Trace	N/A
2	Cu(OTf)_2_	None	THF	4 Å MS	75	rac.
3	Cu(OTf)_2_	**L1**	THF	4 Å MS	30	7
4	Cu(OTf)_2_	**L2**	THF	4 Å MS	75	−24
5	Cu(OTf)_2_	**L3**	THF	4 Å MS	9	rac.
6	Cu(OTf)_2_	**L4**	THF	4 Å MS	21	37
7	Cu(OTf)_2_	**L5**	THF	4 Å MS	46	66
8	Cu(OTf)_2_	**L6**	THF	4 Å MS	82	60
9	Cu(OTf)_2_	**L7**	THF	4 Å MS	68	51
10	Cu(OTf)_2_	**L8**	THF	4 Å MS	88	71
11	Cu(OAc)_2_	**L8**	THF	4 Å MS	70	7
12	Cu(acac)_2_	**L8**	THF	4 Å MS	90	rac.
13	Cu(BF_4_)_2_•H_2_O	**L8**	THF	4 Å MS	94	84
14	Cu(BF_4_)_2_•H_2_O	**L8**	Dioxane	4 Å MS	69	57
15	Cu(BF_4_)_2_•H_2_O	**L8**	MeCN	4 Å MS	78	88
16	Cu(BF_4_)_2_•H_2_O	**L8**	Acetone	4 Å MS	84	94
17^d^	Cu(BF_4_)_2_•H_2_O	**L8**	Acetone	4 Å MS	88	96
18^d, e^	Cu(BF_4_)_2_•H_2_O	**L8**	Acetone	4 Å MS	90	96


^a^Reaction conditions: **1a** (0.10 mmol), **2a** (1.0 mmol), copper salt (10 mol%), ligand (15 mol%) and 4 Å MS (40 mg) at room temperature in solvent (1.0 ml) for 24 h.

^b^Isolated yield.

^c^The ee value was determined by HPLC.

^d^The reaction was conducted at 0 °C.

^e^0.20 mmol of **2a** was added.

### Scope of *β*,*γ*-alkynyl-*α*-imino esters in the reactions

**Fig. 3:**
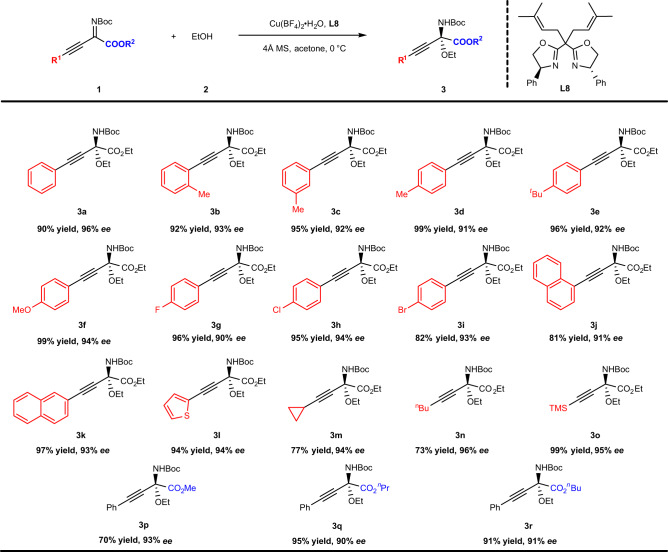
Scope of *β*,*γ*-alkynyl-*α*-imino esters. Reaction conditions: **1** (0.10 mmol), **2a** (0.20 mmol), Cu(BF_4_)_2_ ∙ H_2_O (10 mol%), **L8** (15 mol%), 4 Å MS (40 mg), acetone (1.0 ml), 0 °C, 24 h; the yield refers to isolated yield; the *ee* value was determined by HPLC.

With the optimal reaction conditions in hand, the substrate scope of the *β*,*γ*-alkynyl-*α*-imino esters was investigated. As shown in Fig. 3, this reaction had a good tolerance of imines bearing different substituents. Electron-donating groups at the *ortho*, *meta* or *para*-position of the phenyl group, such as, Me, ^*t*^Bu and OMe, were all amenable to the reaction conditions, giving the corresponding products (**3b**–**3f**) with excellent enantioselectivities (91–94% *ee*) and yields (92–99%). The presence of electron-withdrawing substituents (e.g., F, Cl and Br) at the para-position of the phenyl ring resulted in a slight decrease in yields (82–96%) while maintaining excellent enantioselectivities (90–94% *ee*) (**3g**–**3i**). Changing the phenyl group to an *α*-naphthyl group gave the expected product (**3j**) with excellent enantioselectivity (91% *ee*) and good yield (81%). Compared to this, changing the phenyl group to a *β*-naphthyl group gave the expected product (**3k**) with excellent enantioselectivity (93% *ee*) and yield (97%). Furthermore, even in the case of 2-ethynylthiophene, the expected product (**3l**) was afforded with excellent enantioselectivity (94% *ee*) and yield (94%). Moreover, substrates with alkyl groups (e.g., cyclopropyl and *n*-butyl) linked to the acetylene bond generated products in high enantioselectivities yet with slightly lower yield (**3m**, **3n**). Delightfully, a trimethylsilyl derived chiral *N*,*O*-ketal was formed in 99% yield and 95% *ee* (**3o**). Furthermore, changing the substituents on the ester (**3p**–**3r**) still gave the corresponding products with good to excellent yields and nearly 90% *ee*. The lower yield of product **3p** was attributed to the transesterification of substrates with ethanol. However, the use of phenylimino ester substrates instead of *β*,*γ*-alkynyl-*α*-imino esters resulted in no reaction under the standard conditions (see Supplementary Fig. 1a).

### Scope of alcohols in the reactions

**Fig. 4:**
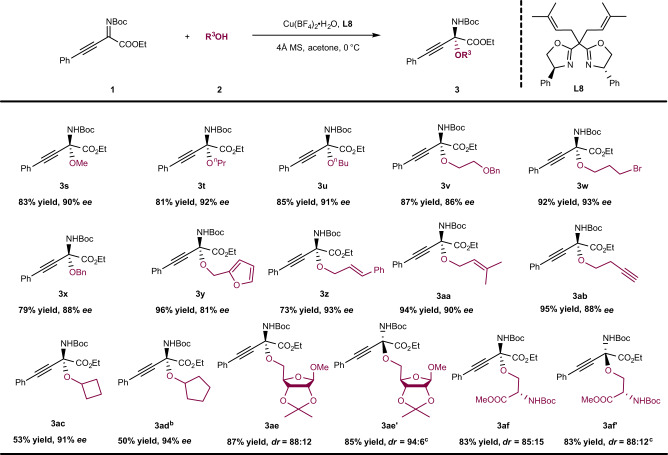
Scope of alcohols. Reaction conditions: **1** (0.10 mmol), **2** (0.20 mmol), Cu(BF_4_)_2_ ∙ H_2_O (10 mol%), **L8** (15 mol%), 4 Å MS (40 mg), acetone (1.0 ml), 0 °C, 24 h; the yield refers to isolated yield; the *ee* value was determined by HPLC. ^a^5.0 Equiv. alcohols was used and it was reacted for 48 h. ^b^Ligand *ent*-**L8** was used instead of **L8**.

With the optimal reaction conditions in hand, the scope of the alcohols was also investigated briefly (Fig. 4). As expected, methanol, *n*-propanol and *n*-butanol could react with *β*,*γ*-alkynyl-*α*-imino ester **1a** smoothly to afford the desired products (**3s**–**3u**) in good yields and enantioselectivities (81–85% yield, 90–92% *ee*). In addition, alcohols possessing other heteroatom groups in the chain, such as Br and OBn, still gave the corresponding products (**3v**, **3w**) with good to excellent yields and enantioselectivities. Benzyl alcohol gave the product (**3x**) in 88% *ee*, but only 79% yield due to the transesterification reaction of the *β*,*γ*-alkynyl-*α*-imino ester **1a**. In contrast, furfuryl alcohol provided its corresponding product (**3y**) with an excellent yield of 96% and a slightly lower enantioselectivity of 81% *ee*. Next, alcohols with unsaturated bonds (e.g., C=C, CΞC) in the chain were also studied; the products (**3z**–**3ab**) were obtained with moderate to excellent yields (73–95%) and enantioselectivities (88–93% *ee*). Subsequently, secondary alcohols (e.g., cyclobutanol and cyclopentanol) were also studied in this reaction system. The desired products (**3ac**, **3ad**) were obtained with low yields (nearly 50%), but with excellent enantioselectivities (91% *ee*, 94% *ee*). However, use of isopropanol only provided a trace amount of desired product (see Supplementary Fig. 1b). These results show that the yield decreases significantly with an increase in the steric hindrance at the *α*-position of the hydroxyl group. In addition, this reaction could also be used to modify glucose derivatives or natural amino acid derivatives with primary alcohol structures, giving the corresponding products (**3ae**, **3af**) with good yields (87%, 83%) and diastereoselectivities (88:12 *dr*, 85:15 *dr*). However, the use of ligand *ent*-**L8** gave better results affording corresponding **3ae′** and **3af′** (the diastereomers of **3ae** and **3af**) in 85% yield with 94:6 *dr* and 83% yield with 88:12 *dr*, respectively, due to the chiral matching phenomenon. Finally, the use of phenol derivatives instead of alcohols as nucleophiles in the catalytic system gave a complex mixture of products and only a trace amount of the corresponding desired product was detected (see Supplementary Fig. 1c).

### Possible reaction pathways

**Fig. 5:**
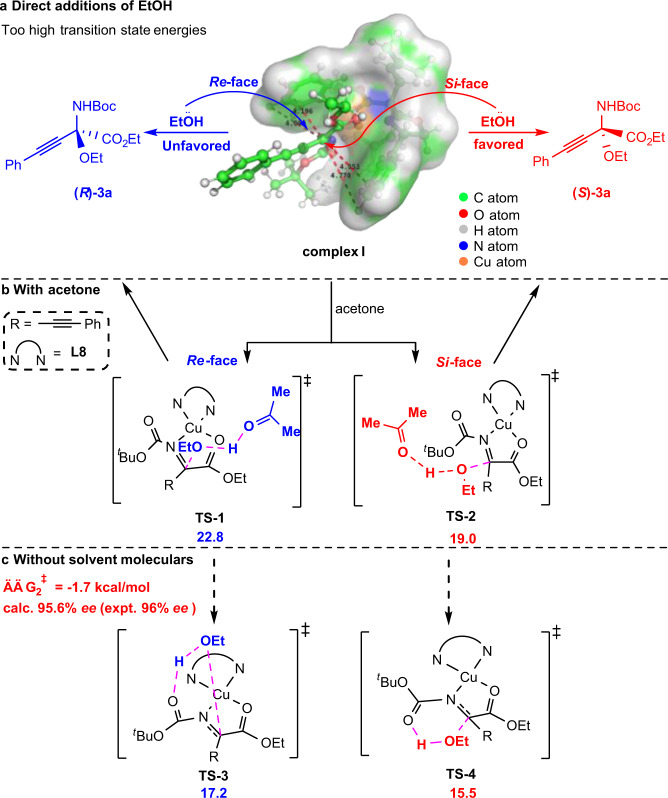
Two possible competing pathways to form product **3a**. **a** Direct additions of EtOH. **b** The transition states with acetone. **c** The transition states without solvent moleculars.

Two DFT calculations concerning the competing pathways to form chiral product **3a** are shown in Fig. 5. Due to the steric hindrance of the phenyl group of the bis(oxazoline) and the Boc group, the nitrogen and oxygen atoms of substrate **1a** coordinate to the copper atom to form a square-planar complex **I** with a distortion (DFT calculations show that the dihedral angle is about 43 degrees, see Supplementary Fig. 4). Ethanol favors attack of the C=N double bond from the *Si*-face because of the larger cavity (see complex **I**). Interestingly, the pathway for direct addition from ethanol to the C=N double bond is very high in energy (see Supplementary Fig. 5). Based on our previous studies on the influence of solvents in the reaction^52–55^, the transition state energy to product *R*-**3a** is reduced to 22.8 kcal/mol (**TS-1**) and the other to 19.0 kcal/mol (**TS-2**) when the solvent molecule (acetone) is put into the reaction system (Fig. 5b). It is worth noting that the carbonyl group of the acetone molecule plays a role as a base in assisting the extraction of hydrogen atoms from ethanol. However, the reaction can still proceed smoothly in toluene (83% yield, 63% *ee*, see Supplementary Table 3). Therefore, there appears to be another group in the reaction system that assists in the extraction of hydrogen from ethanol. Therefore, we conceived that the carbonyl group of the Boc substituent may play a key role in the reaction system. As expected, the transition state energy for *R*-**3a** is reduced to 17.2 kcal/mol (**TS-3**) and the other to 15.5 kcal/mol (**TS-4**) via synergistic extraction of the hydrogen atoms by the oxygen atom of the carbonyl group in the Boc group (Fig. 5c). The energy gap between the two transition states is ~1.7 kcal/mol, and the calculated enantioselectivity is 96% *ee* which is in agreement with the experimental value (96% *ee*). In addition, the reaction did not proceed smoothly under the standard reaction conditions if an acetyl group was used instead of a Boc group. However, when the temperature was raised to room temperature, the *N*-acyl substrates could afford the corresponding products in 34% yield. It is worth noting that if the solvent is changed to toluene, no reaction occurs at room temperature (see Supplementary Fig. 1d). These results suggested that the carbonyl functionality of the acetone or Boc group may act as a base.

### Gram-scale synthesis and synthetic applications

**Fig. 6:**
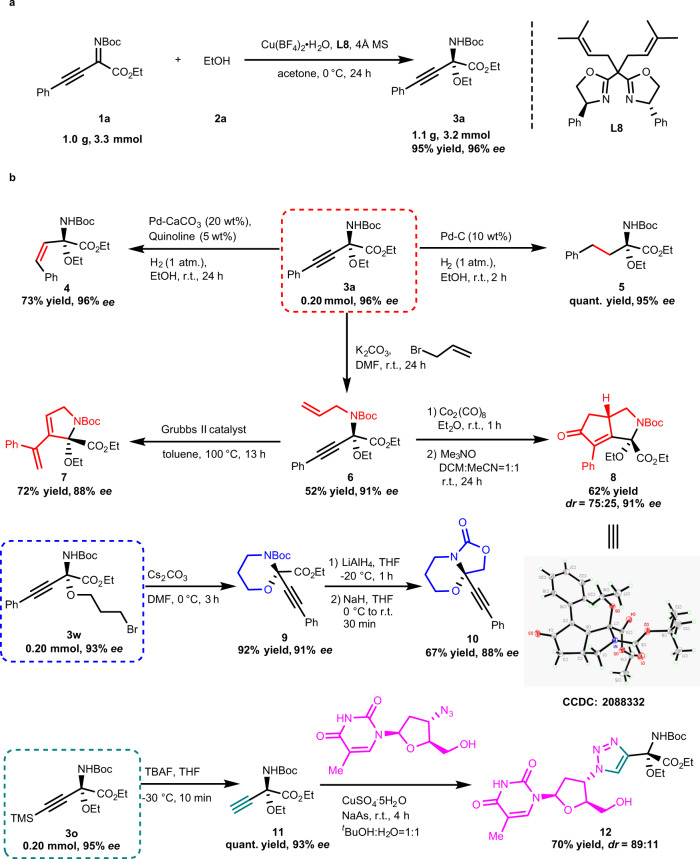
Gram-scale synthesis and synthetic applications. **a** Gram-scale synthesis of **3a**. **b** Transformation of linear chiral *N*,*O*-ketals.

Considering the importance of the structural skeleton of linear chiral *N*,*O*-ketals products, we explored the practicality of our methodology. A gram-scale reaction using *β*,*γ*-alkynyl-*α*-imino esters **1a** and ethanol **2a** was carried out (Fig. 6a). The product **3a** was obtained with results comparable to those shown in Fig. 3. In addition, the linear chiral *N*,*O*-ketals products **3** can undergo further transformations (Fig. 6b). Linear *N*,*O*-ketals products **3a** could be converted to alkenyl and alkyl compounds (**4**, **5**) via hydrogenation, without any change in enantioselectivities. Furthermore, the amino group of **3a** is easily allylated to give product **6** in 52% yield and 91% *ee*, resulting in only a slight decrease in enantioselectivity. The product **6** could be further transformed into chiral 2,5-dihydro-1*H*-pyrrole **7** in 72% yield and 88% *ee* by Grubbs II catalyst^56^. Meanwhile, the cyclopentenone derivative **8** could also be obtained from product **6** in 62% yield and 75:25 *dr* via a Pauson-Khand reaction^57^. The absolute configuration of product **8** was determined by X-ray diffraction (see Supplementary Fig. 3). In addition to product **3a**, other linear chiral *N*,*O*-ketals products with specific groups can also undergo interesting transformations. For instance, product **3w** could form chiral cyclic *N*,*O*-ketal **9** via basic cyclization in 92% yield with only a slightly decrease in enantioselectivity (91% *ee*). The oxazolidinone **10** could be obtained in 67% yield with 88% *ee* via the reduction of **9** using LiAlH_4_ as reductant followed by a cyclization reaction in the presence of NaH. The trimethylsilyl group of the *N*,*O*-ketal **3o** could be removed with TBAF to give **11** quantitatively. The alkynyl group in product **11** facilitated its application in “click” reactions, as exemplified by the synthesis of enantioenriched *N,O*-ketal-functionalized zidovudine **12** with 70% yield and 89:11 *dr*.

## Discussion

In summary, we have demonstrated a direct Cu-catalyzed asymmetric addition reaction of alcohols to *β*,*γ*-alkynyl-*α*-imino esters that was successfully applied to the enantioselective synthesis of linear chiral *N*,*O*-ketals products motifs. The protocol proceeds smoothly under mild reaction conditions and can accommodate a wide scope of *β*,*γ*-alkynyl-*α*-imino esters, delivering the corresponding products in high yields and with excellent enantioselectivities (up to 96% *ee*). Furthermore, a variety of primary alcohols, including some glucose derivatives or natural amino acid derivatives, can be used in this reaction giving the desired products in good to excellent yields and enantioselectivities. DFT computational studies indicate that direct addition of alcohols is difficult. The Boc group of the substrates can assist as a base in the extraction of hydrogen atoms from the alcohols to promote the reaction via a six-membered ring transition state. The reaction system could be conducted smoothly on a gram-scale with comparable results. Several linear chiral *N*,*O*-ketals products could be transformed to some useful scaffolds.

## Methods

### General procedure for the copper-bis(oxazoline) catalyzed asymmetric addition of alcohols to linear *β*, *γ*-alkynyl-*α*-imino esters

A flame-dried Schlenk tube equipped with a magnetic stirring bar, was charged with a mixture of Cu(BF_4_)_2_•H_2_O (10 mol%), **L2** (15 mol%) and 4 Å MS (40 mg). After being evacuated and refilled with nitrogen three times, acetone (1.0 mL) was added to the Schlenk tube and the mixture was stirred at room temperature under a N_2_ atmosphere for 1 h. Ketimine **1** (0.10 mmol) and alcohol **2** (0.20 mmol) were added sequentially. The reaction mixture was allowed to stir under a N_2_ atmosphere at 0 °C for 24 h. When the reaction was completed, solvent was evaporated in vacuo and the residue was purified by flash silica gel column chromatography (PE/EtOAc = 10/1) to give product **3a**–**3af**.

## Supplementary information

Supplementary information

## Data Availability

The data generated in this study are provided in the Supplementary Information file. For the experimental procedures, data of NMR and HPLC analysis and Cartesian coordinates of the optimized structures, see Supplementary Methods, Notes and Figures in Supplementary Information file. The X-ray crystallographic coordinates for structures reported in this study have been deposited at the Cambridge Crystallographic Data Centre (**3a**: CCDC 2063358, **8**: CCDC 2088332). These data could be obtained free of charge from The Cambridge Crystallographic Data Centre (https://www.ccdc.cam.ac.uk/data_request/cif).
