# Supplementary material for: Cu-catalyzed asymmetric addition of alcohols to β,γ-alkynyl-α-imino esters for the construction of linear chiral N,O-ketals
Source: Nat Commun. 2022 Jan 20;13:400. doi: 10.1038/s41467-022-28002-7 (PMC8776757; doi:10.1038/s41467-022-28002-7)
Supplement: Supplementary file 1 — Supplementary information [file 41467_2022_28002_MOESM1_ESM.pdf]

·Supplementary Information

Cu-catalyzed Asymmetric Addition of Alcohols to *β*,*γ*-Alkynyl-*α*-Imino Esters to Construct Linear Chiral *N*,*O*-Ketals

Cheng Sheng,[a] Zheng Ling,[a] Yicong Luo,[a] and Wanbin Zhang*[a]

[a] C. Sheng, Dr. Z. Ling and Prof. Dr. W. Zhang
Shanghai Key Laboratory for Molecular Engineering of Chiral Drugs
Frontier Science Center for Transformative Molecules
School of Chemistry and Chemical Engineering
Shanghai Jiao Tong University
800 Dongchuan Road, Shanghai 200240, China
E-mail: wanbin@sjtu.edu.cn

Supplementary Methods

General Information

All of alcohol addition reactions were performed in Schlenk tubes under an atmosphere of nitrogen. Solvents were dried and distilled before use by standard procedures. Commercially available reagents were used without further purification. The workup was carried out in air, unless otherwise noted. Column chromatography was performed using silica gel (100-200 mesh). Melting points were measured with SGW X-4 micro melting point apparatus. ^{1}H NMR (400, 500 MHz), ^{13}C NMR (101, 125MHz) and ^{19}F NMR (376 MHz) spectra were recorded on a Bruker Avance III HD 400 MHz NMR spectrometer or a Bruker Avance III HD 500 MHz NMR Spectrometer. HRMS was performed on a Waters Micromass Q-TOF Premier mass spectrometer at the Instrumental Analysis Center of Shanghai Jiao Tong University. Optical rotations were measured on a Rudolph Research Analytical Autopol VI automatic polarimeter using a 50 mm path-length cell at 589 nm. Enantioselectivity was measured by high performance liquid chromatography (HPLC) using Daicel Chiralcel columns with hexane/isopropanol as eluent. The X-ray single crystal diffraction data were collected on a Bruker D8 VENTURE CMOS photon 100 diffractometer with helios mx multilayer monochrmator Cu Kα radiation (λ = 1.54178 Å) at the Instrumental Analysis Center of Shanghai Jiao Tong University.

Supplementary Note 1

Preparation of α-Ketimino Esters 1

General Procedure A (Substrates 1a-1o were synthesized according to procedure A):

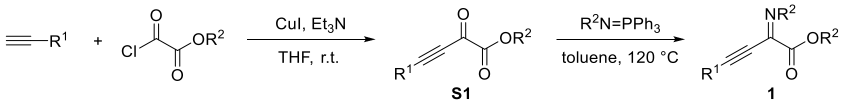

Step 1: Ketoesters **S1** were prepared based on the reported procedures.[1] To a stirred suspension of CuI (10 mol%) in THF (0.3 M), trimethylamine (2.0 equiv.) was added slowly. When the solution become transparent, alkyne (1.0 equiv.) and ethyl 2–chloro-2-oxoacetate (1.5 equiv.) were added sequentially and the resulting mixture was stirred at room temperature for 24 hours. The reaction was quenched by saturated $NaHCO_3$ aqueous solution and filtered through a layer of celite (when R^1 = trimethylsilyl, the resulting reaction was filtered directly without quenching by saturated $NaHCO_3$ aqueous solution). The filtrate was extracted with ethyl acetate. The organic phases were combined, dried over Na_2SO_4 and concentrated in vacuo. The crude product was purified by silica gel chromatography (petroleum ether/EtOAc = 10/1) to give the **S1**.

Step 2: *β,γ*-Alkynyl-*α*-imino esters **1** were prepared based on the reported procedures.[2] An oven-dried round bottom two necks flask was added ketoesters **S1** (1.0 equiv.), N-Boc-triphenyliminophosphorane (1.2 equiv.) and toluene (0.5 M). The mixture was heated to reflux at 120 °C and stirred for 24h. After cooling to room temperature, the mixture was poured into petroleum ether and then filtered. The filtrate was concentrated under vacuum. The residue was purified by flash chromatography (petroleum ether/EtOAc = 20/1 to 10/1) to give the

corresponding product **1a-1p**, **1s** was synthesized by using N-Ac-triphenyliminophosphorane, the corresponding NMR data of **1a-1m** and **1p** are consistent with literature reports. [2]

General Procedure B (Substrates 1p, 1q, 1r were synthesized according to procedure B):

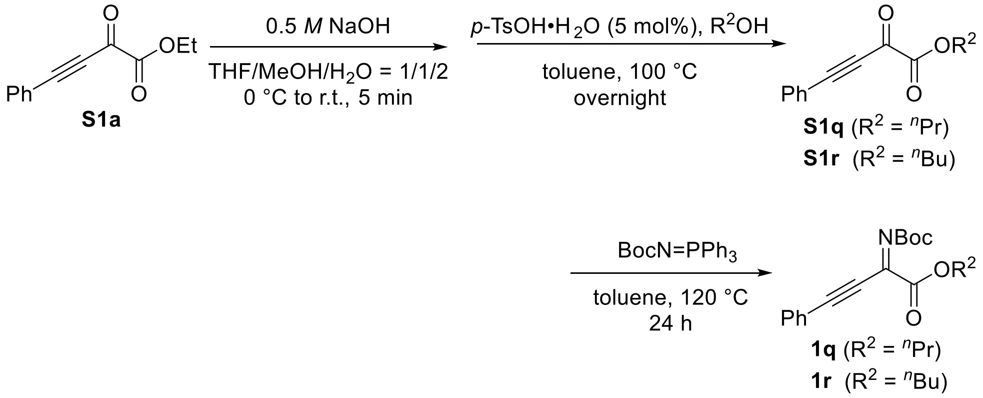

Ketoesters **S1q**, **S1r** were prepared based on the reported procedures.[3]

Step 1: To a solution of α-keto ester **S1a** (1.0 equiv.) in (THF/MeOH/H_2O = 1/1/2) was added dropwise a 0.5 M solution of aqueous NaOH (0.8 equiv.) at 0 °C. The mixture was stirred at 0 °C to room temperature for 5 min. Volatiles were then removed under vacuum, and the resulting mixture was acidified with 1 M solution of aqueous HCl until pH≤2, then the mixture was extracted with ethyl acetate, and washed with brine (100 mL). The combined organic layers were dried over Na_2SO_4, concentrated under reduced pressure, and the corresponding α-keto carboxylic acid, which was obtained almost quantitatively, was used in the next step without further purification.

Step 2: A mixture of α-keto carboxylic acid (2.0 equiv.), corresponding alcohol (1.0 equiv.), and *p*-toluenesulfonic acid monohydrate (5 mol%) in toluene (0.2 M) was stirred at reflux temperature overnight. After cooling to room temperature, the resulting mixture was quenched with saturated solution of aqueous $NaHCO_3$, extracted with ethyl acetate, and washed with brine. The combined extracts were dried over Na_2SO_4. The organic phase was concentrated under reduced pressure, and the residue was purified by silica gel chromatography (petroleum ether/EtOAc = 10/1) to give the **S1q**, **S1r**.

Step 3: An oven-dried round bottom two necks flask was added ketoesters **S1** (1.0 equiv.), N-Boc-triphenyliminophosphorane (1.2 equiv.) and toluene (0.5 M). The mixture was heated to 120 °C to reflux and stirred for 24 h. After cooling to room temperature, the mixture was poured into petroleum ether and then filtered. The filtrate was concentrated under vacuum. The residue was purified by flash chromatography (petroleum ether/EtOAc = 20/1 to 10/1) to give the corresponding product **1q**, **1r**.

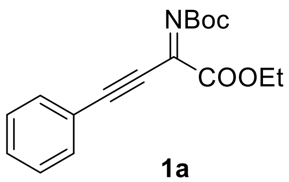

1a

Ethyl (*E*)-2-((tert-butoxycarbonyl)imino)-4-phenylbut-3-ynoate (1a): Pale yellow solid (general procedure A, 68% yield). ^{1}H NMR (400 MHz, Chloroform-*d*) δ 7.56 (d, *J* = 7.6 Hz, 2H), 7.47 (t, *J* = 7.6 Hz, 1H), 7.39 (t, *J* = 7.6 Hz, 2H), 4.42 (q, *J* = 7.2 Hz, 2H), 1.58 (s, 9H), 1.41 (t, *J* = 7.2 Hz, 3H).

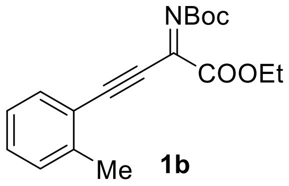

1b

Ethyl (*E*)-2-((tert-butoxycarbonyl)imino)-4-(o-tolyl)but-3-ynoate (1b): Pale yellow solid (general procedure A, 70% yield). ^{1}H NMR (400 MHz, Chloroform-*d*) δ 7.49 (d, *J* = 7.6 Hz, 1H), 7.36 (t, *J* = 7.6 Hz, 1H), 7.26 – 7.24 (m, 1H), 7.20 (t, *J* = 7.6 Hz, 1H), 4.42 (q, *J* = 7.2 Hz, 2H), 2.51 (s, 3H), 1.58 (s, 9H), 1.41 (t, *J* = 7.2 Hz, 3H).

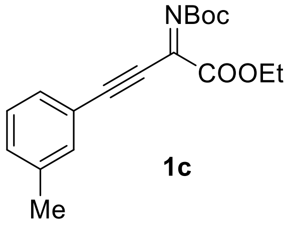

1c

Ethyl (*E*)-2-((tert-butoxycarbonyl)imino)-4-(m-tolyl)but-3-ynoate (1c): Pale yellow solid (general procedure A, 66% yield). ^{1}H NMR (400 MHz, Chloroform-*d*) δ 7.40 – 7.32 (m, 2H), 7.32 – 7.22 (m, 2H), 4.42 (q, *J* = 7.2 Hz, 2H), 2.35 (s, 3H), 1.59 (s, 9H), 1.41 (t, *J* = 7.2 Hz, 3H).

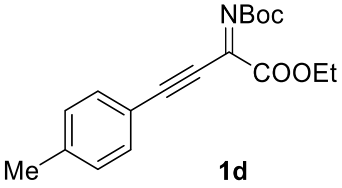

1d

Ethyl (*E*)-2-((tert-butoxycarbonyl)imino)-4-(p-tolyl)but-3-ynoate (1d): Pale yellow solid (general procedure A, 72% yield). ^{1}H NMR (400 MHz, Chloroform-*d*) δ 7.44 (d, *J* = 8.0 Hz, 2H), 7.19 (d, *J* = 7.6 Hz, 2H), 4.41 (q, *J* = 7.2 Hz, 2H), 2.39 (s, 3H), 1.58 (s, 10H), 1.41 (t, *J* = 7.2 Hz, 3H).

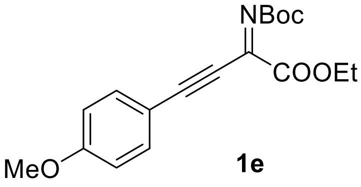

1e

Ethyl (*E*)-2-((tert-butoxycarbonyl)imino)-4-(4-methoxyphenyl)but-3-ynoate (1e): Pale yellow solid (general procedure A, 71% yield). ^{1}H NMR (400 MHz, Chloroform-*d*) δ 7.50 (d, *J* = 8.8 Hz, 2H), 6.90 (d, *J* = 8.8 Hz, 2H), 4.41 (q, *J* = 7.2 Hz, 2H), 3.85 (s, 3H), 1.59 (s, 9H), 1.41 (t, *J* = 7.2 Hz, 3H).

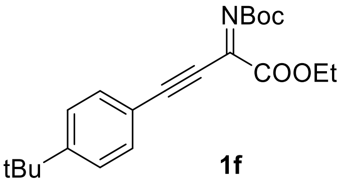

1f

Ethyl (*E*)-2-((tert-butoxycarbonyl)imino)-4-(4-(tert-butyl)phenyl)but-3-ynoate (1f): Pale yellow oil (general procedure A, 74% yield). ^{1}H NMR (400 MHz, Chloroform-*d*) δ 7.49 (d, *J* = 8.0 Hz, 2H), 7.41 (d, *J* = 8.0 Hz, 2H), 4.42 (q, *J* = 7.2 Hz, 2H), 1.59 (s, 9H), 1.41 (t, *J* = 7.2 Hz, 3H), 1.32 (s, 9H).

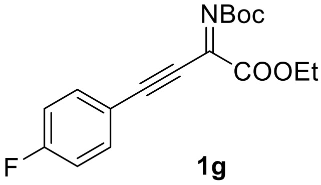

1g

Ethyl (*E*)-2-((tert-butoxycarbonyl)imino)-4-(4-fluorophenyl)but-3-ynoate (1g): Pale yellow solid (general procedure A, 71% yield). ^{1}H NMR (400 MHz, Chloroform-*d*) δ 7.60 – 7.52 (m, 2H), 7.09 (t, *J* = 8.8 Hz, 2H), 4.42 (q, *J* = 7.2 Hz, 2H), 1.58 (s, 9H), 1.41 (t, *J* = 7.2 Hz, 3H).

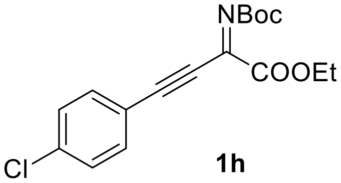

1h

Ethyl (*E*)-2-((tert-butoxycarbonyl)imino)-4-(4-chlorophenyl)but-3-ynoate (1h): Yellow solid (general procedure A, 73% yield). ^{1}H NMR (400 MHz, Chloroform-*d*) δ 7.49 (d, *J* = 7.6 Hz, 2H), 7.38 (d, *J* = 7.6 Hz, 2H), 4.42 (q, *J* = 7.2 Hz, 2H), 1.58 (s, 7H), 1.41 (t, *J* = 7.2 Hz, 3H).

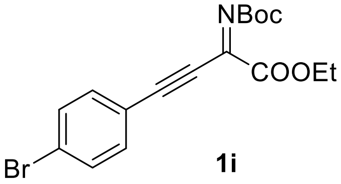

1i

Ethyl (*E*)-4-(4-bromophenyl)-2-((tert-butoxycarbonyl)imino)but-3-ynoate (1i): Yellow solid (general procedure A, 60% yield). ^{1}H NMR (400 MHz, Chloroform-*d*) δ 7.54 (d, *J* = 8.4 Hz, 2H), 7.41 (d, *J* = 8.4 Hz, 2H), 4.42 (q, *J* = 7.2 Hz, 2H), 1.57 (s, 9H), 1.41 (t, *J* = 7.2 Hz, 3H).

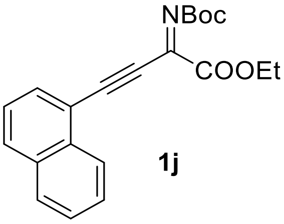

1j

Ethyl (*E*)-2-((tert-butoxycarbonyl)imino)-4-(naphthalen-1-yl)but-3-ynoate (1j): Yellow solid (general procedure A, 85% yield). ^{1}H NMR (400 MHz, Chloroform-*d*) δ 8.41 (d, *J* = 8.0 Hz, 1H), 7.97 (d, *J* = 8.4 Hz, 1H), 7.89 (d, *J* = 8.0 Hz, 1H), 7.80 (d, *J* = 7.2 Hz, 1H), 7.67 – 7.60 (m, 1H), 7.60 – 7.54 (m, 1H), 7.51 – 7.45 (m, 1H), 4.48 (q, *J* = 7.2 Hz, 2H), 1.60 (s, 9H), 1.47 (t, *J* = 7.2 Hz, 3H).

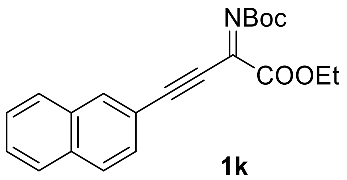

1k

Ethyl (*E*)-2-((tert-butoxycarbonyl)imino)-4-(naphthalen-2-yl)but-3-ynoate (1k): Yellow solid (general procedure A, 60% yield). ^{1}H NMR (400 MHz, Chloroform-*d*) δ 8.12 (s, 1H), 7.87 – 7.81 (m, 3H), 7.63 – 7.46 (m, 3H), 4.44 (q, *J* = 7.2 Hz, 2H), 1.61 (s, 9H), 1.43 (t, *J* = 7.2 Hz, 3H).

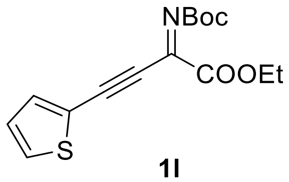

1l

Ethyl (*E*)-2-((tert-butoxycarbonyl)imino)-4-(thiophen-2-yl)but-3-ynoate (1l): Yellow solid (general procedure A, 60% yield). ^{1}H NMR (400 MHz, Chloroform-*d*) δ 7.52 (d, *J* = 5.2 Hz, 1H), 7.47 (d, *J* = 4.0 Hz, 1H), 7.08 (dd, *J* = 5.2, 4.0 Hz, 1H), 4.42 (q, *J* = 7.2 Hz, 2H), 1.60 (s, 9H), 1.41 (t, *J* = 7.2 Hz, 3H).

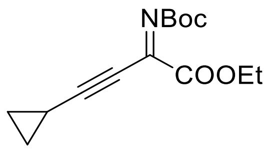

1m

Ethyl (*E*)-2-((tert-butoxycarbonyl)imino)-4-cyclopropylbut-3-ynoate (1m): Pale yellow oil (general procedure A, 27% yield). ^{1}H NMR (400 MHz, Chloroform-*d*) δ 4.36 (q, *J* = 7.2 Hz, 2H), 1.55 (s, 9H), 1.52 – 1.46 (m, 1H), 1.37 (t, *J* = 7.2 Hz, 3H), 1.06 – 0.99 (m, 2H), 0.97 – 0.90 (m, 2H).

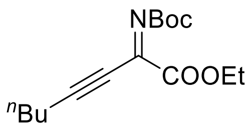

1n

Ethyl (*E*)-2-((tert-butoxycarbonyl)imino)oct-3-ynoate (1n): Yellow oil (general procedure A, 10% yield). ^{1}H NMR (400 MHz, Chloroform-*d*) δ 4.37 (q, *J* = 7.2 Hz, 2H), 2.46 (t, *J* = 7.2 Hz,

2H), 1.63 – 1.57 (m, 2H), 1.55 (s, 9H), 1.50 – 1.42 (m, 2H), 1.37 (t, J = 7.2 Hz, 3H), 0.92 (t, J = 7.2 Hz, 3H); ^{13}C NMR (101 MHz, $CDCl_3$) δ 161.4, 156.0, 145.0, 105.1, 83.7, 73.4, 63.2, 29.7, 28.0, 21.9, 19.3, 14.0, 13.5; HRMS (ESI) calcd for $C_{15}H_{23}NO_4$ $[M+Na]^+$ 304.1519, found 304.1519.

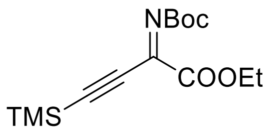

1o

Ethyl (*E*)-2-((tert-butoxycarbonyl)imino)-4-(trimethylsilyl)but-3-ynoate (1o): Pale yellow oil (general procedure A, 83% yield). ^{1}H NMR (400 MHz, Chloroform-*d*) δ 4.38 (q, J = 7.2 Hz, 2H), 1.56 (s, 9H), 1.38 (t, J = 7.2 Hz, 3H), 0.26 (s, 9H); ^{13}C NMR (101 MHz, $CDCl_3$) δ 161.0, 159.6, 144.4, 109.6, 94.2, 83.9, 63.2, 28.0, 14.0, -0.8; HRMS (ESI) calcd for $C_{14}H_{23}NO_4Si$ $[M+Na]^+$ 320.1289, found 320.1286.

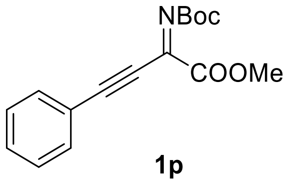

1p

Methyl (*E*)-2-((tert-butoxycarbonyl)imino)-4-phenylbut-3-ynoate (1p): Yellow oil (general procedure A, 80% yield). ^{1}H NMR (400 MHz, Chloroform-*d*) δ 7.56 (d, J = 7.6 Hz, 2H), 7.50 – 7.44 (m, 1H), 7.39 (t, J = 7.6 Hz, 2H), 3.97 (s, 3H), 1.58 (s, 9H).

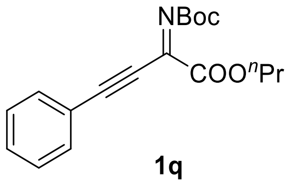

1q

Propyl (*E*)-2-((tert-butoxycarbonyl)imino)-4-phenylbut-3-ynoate (1q): Yellow oil (general procedure B, 50% yield). ^{1}H NMR (400 MHz, $CDCl_3$) δ 7.58 – 7.52 (m, 2H), 7.49 – 7.44 (m, 1H), 7.43 – 7.36 (m, 2H), 4.32 (t, J = 6.8 Hz, 2H), 1.88 – 1.75 (m, 2H), 1.59 (s, 9H), 1.03 (t, J = 7.2 Hz, 3H); ^{13}C NMR (101 MHz, $CDCl_3$) δ 161.2, 160.0, 144.9, 132.8, 131.0, 128.7, 120.0, 101.1, 84.1, 81.0, 68.7, 28.1, 21.8, 10.3; HRMS (ESI) calcd for $C_{18}H_{21}NO_4$ $[M+Na]^+$ 338.1363, found 338.1367.

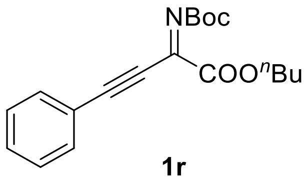

1r

Butyl (*E*)-2-((tert-butoxycarbonyl)imino)-4-phenylbut-3-ynoate (1r): Yellow oil (general procedure B, 55% yield). ^{1}H NMR (400 MHz, Chloroform-*d*) ^{1}H NMR (400 MHz, $CDCl_3$) δ 7.61

– 7.51 (m, 2H), 7.50 – 7.44 (m, 1H), 7.43 – 7.35 (m, 2H), 4.36 (t, J = 6.8 Hz, 2H), 1.80 – 1.71 (m, 2H), 1.58 (s, 9H), 1.50 – 1.42 (m, 2H), 0.97 (t, J = 7.2 Hz, 3H); ^{13}C NMR (101 MHz, $CDCl_3$) δ 161.2, 156.0, 144.9, 132.8, 131.0, 128.7, 120.0, 101.1, 84.1, 81.0, 67.1, 30.4, 28.1, 19.0, 13.7; HRMS (ESI) calcd for $C_{19}H_{23}NO_4$ [M+Na]$^+$ 352.1519, found 352.1523.

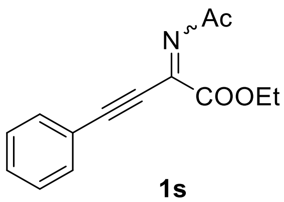

1s

Ethyl-2-(acetylimino)-4-phenylbut-3-ynoate (1s): Consists of two isomers; Red oil (general procedure A, 30% yield). One isomer: ^{1}H NMR (400 MHz, Chloroform-*d*) ^{1}H NMR (400 MHz, Chloroform-*d*) δ 8.10 – 8.03 (m, 2H), 7.51 – 7.44 (m, 3H), 4.27 (q, J = 7.2 Hz, 2H), 2.63 (s, 3H), 1.29 (t, J = 7.2 Hz, 3H); Another isomer: ^{1}H NMR (400 MHz, Chloroform-*d*) δ 7.59 – 7.54 (m, 2H), 7.51 – 7.43 (m, 1H), 7.43 – 7.36 (m, 2H), 4.42 (q, J = 7.2 Hz, 2H), 2.33 (s, 3H), 1.42 (t, J = 7.2 Hz, 3H). ^{13}C NMR (101 MHz, $CDCl_3$) δ 174.4, 165.2, 162.9, 151.1, 141.5, 133.0, 131.1, 130.8, 129.7, 129.2, 128.7, 128.5, 119.8, 63.5, 62.6, 24.0, 14.5, 14.0, 13.9; HRMS (ESI) calcd for $C_{14}H_{13}NO_3$ [M+Na]$^+$ 266.0788, found 266.0790.

General Procedure for Synthesizing Bis(oxazoline) Ligands L1-L4, L5, L8

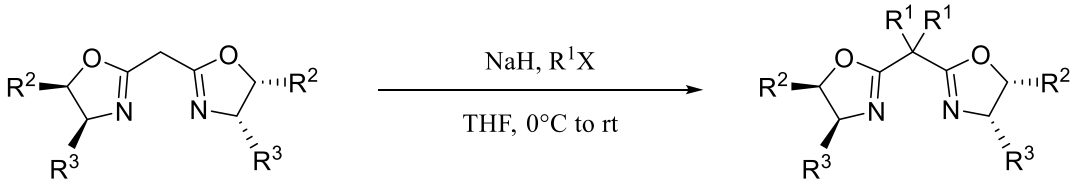

To a solution of corresponding unsubstituted Bis(oxazoline) starting material (1.0 mmol, 1.0 equiv.) in anhydrous THF (10 mL) was added NaH (60 % dispersion in mineral oil, 4.0 mmol, 4.0 equiv.) in one portion at 0 ℃ under N_2 atmosphere. Then the mixture was stirred at room temperature for 45 min. Corresponding halohydrocarbon (2.2 mmol, 2.2 equiv.) was then added to the mixture. The reaction was monitored by TLC. When completed, the reaction was quenched with H_2O (20 mL) and extracted with EtOAc. The combined organic layer was dried over anhydrous Na_2SO_4 and concentrated in vacuo. The residue was purified by silica gel chromatography (eluted with PE/EtOAc = 10/1) to afford the title product.

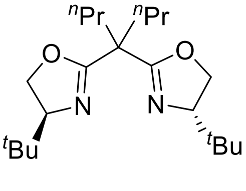

L1

(4*S*,4'*S*)-2,2'-(heptane-4,4-diyl)bis(4-(*tert*-butyl)-4,5-dihydrooxazole) (L1): Pale yellow wax-like solid (71% yield). ^{1}H NMR (500 MHz, Chloroform-*d*) δ 4.11 (dd, J = 10.0, 8.5 Hz, 2H), 4.02

(dd, *J* = 8.5, 7.5 Hz, 2H), 3.85 (dd, *J* = 10.0, 7.5 Hz, 2H), 2.00 (ddd, *J* = 13.5, 12.0, 4.5 Hz, 2H), 1.86 (ddd, *J* = 14.0, 12.5, 5.0 Hz, 2H), 1.31 – 1.17 (m, 4H), 0.92 (t, *J* = 7.5 Hz, 6H), 0.88 (s, 18H); ^{13}C NMR (126 MHz, $CDCl_3$) δ 167.4, 75.5, 68.4, 46.0, 34.9, 33.9, 25.8, 17.4, 14.5; HRMS (ESI) calcd for $C_{21}H_{38}N_2O_2$ $[M+H]^+$ 351.3006, found 351.3013.

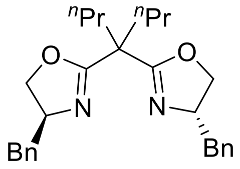

L2

(4*S*,4'*S*)-2,2'-(heptane-4,4-diyl)bis(4-benzyl-4,5-dihydrooxazole) (L2): Pale yellow oil (65% yield). ^{1}H NMR (500 MHz, Chloroform-*d*) δ 7.32 – 7.27 (m, 4H), 7.24 – 7.18 (m, 6H), 4.45 – 4.36 (m, 2H), 4.16 – 4.11 (m, 2H), 3.97 (dd, *J* = 8.5, 7.5 Hz, 2H), 3.16 (dd, *J* = 13.5, 4.5 Hz, 2H), 2.60 (dd, *J* = 13.5, 9.0 Hz, 2H), 1.99 – 1.88 (m, 4H), 1.24 – 1.10 (m, 4H), 0.93 (t, *J* = 7.5 Hz, 6H); ^{13}C NMR (126 MHz, $CDCl_3$) δ 168.2, 137.9, 129.3, 128.5, 126.4, 71.6, 67.2, 45.9, 41.6, 34.9, 17.2, 14.4; HRMS (ESI) calcd for $C_{27}H_{34}N_2O_2$ $[M+H]^+$ 419.2693, found 419.2693.

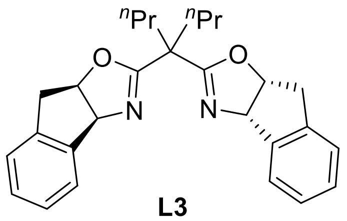

L3

(3*aS*,3*a'S*,8*aR*,8*a'R*)-2,2'-(heptane-4,4-diyl)bis(3*a*,8*a*-dihydro-8*H*-indeno[1,2-*d*]oxazole) (L3): white solid (83% yield); Mp: 106 – 107 °C. ^{1}H NMR (500 MHz, Chloroform-*d*) δ 7.53 – 7.48 (m, 2H), 7.27 – 7.24 (m, 6H), 7.24 – 7.20 (m, 2H), 5.52 (d, *J* = 7.5 Hz, 2H), 5.22 – 5.16 (m, 2H), 3.25 (dd, *J* = 18.0, 7.0 Hz, 2H), 2.84 (d, *J* = 18.0 Hz, 2H), 1.95 – 1.78 (m, 4H), 1.06 – 0.95 (m, 2H), 0.95 – 0.85 (m, 2H), 0.77 (t, *J* = 7.5 Hz, 6H); ^{13}C NMR (126 MHz, $CDCl_3$) δ 168.0, 141.9, 139.6, 128.3, 127.4, 125.6, 125.0, 82.7, 76.3, 45.6, 39.5, 33.8, 16.7, 14.2; HRMS (ESI) calcd for $C_{27}H_{30}N_2O_2$ $[M+H]^+$ 415.2380, found 415.2375.

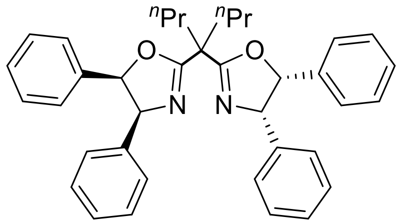

L4

(4*S*,4'*S*,5*R*,5'*R*)-2,2'-(heptane-4,4-diyl)bis(4,5-diphenyl-4,5-dihydrooxazole) (L4): white solid (78% yield); Mp: 173 – 174 °C. ^{1}H NMR (500 MHz, Chloroform-*d*) δ 7.07 – 6.98 (m, 10H), 6.98 – 6.90 (m, 10H), 5.95 (d, *J* = 10.0 Hz, 2H), 5.58 (d, *J* = 10.0 Hz, 2H), 2.40 (ddd, *J* = 14.0, 12.5, 5.0 Hz, 2H), 2.24 (ddd, *J* = 14.0, 12.5, 5.0 Hz, 2H), 1.59 – 1.47 (m, 4H), 1.07 (t, *J* = 7.5 Hz, 6H); ^{13}C NMR (126 MHz, $CDCl_3$) δ 169.2, 137.4, 136.0, 127.9, 127.6, 127.4, 126.9, 126.6,

86.1, 73.7, 46.9, 35.3, 17.6, 14.5; HRMS (ESI) calcd for $C_{37}H_{38}N_2O_2$ [M+H]$^+$ 543.3006, found 543.3014.

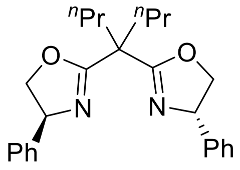

L5

(4*S*,4'*S*)-2,2'-(heptane-4,4-diyl)bis(4-phenyl-4,5-dihydrooxazole) (L5): Pale yellow solid (76% yield); Mp: 75 – 76 °C. ^{1}H NMR (500 MHz, Chloroform-*d*) δ 7.36 – 7.31 (m, 4H), 7.29 – 7.26 (m, 6H), 5.24 (dd, *J* = 10.0, 8.0 Hz, 2H), 4.66 (dd, *J* = 10.0, 8.5 Hz, 2H), 4.12 (t, *J* = 8.0 Hz, 2H), 2.16 – 2.03 (m, 4H), 1.42 – 1.30 (m, 4H), 0.97 (t, *J* = 7.5 Hz, 6H); ^{13}C NMR (126 MHz, $CDCl_3$) δ 169.1, 142.4, 128.7, 127.5, 126.7, 75.0, 69.6, 46.3, 35.0, 17.4, 14.4; HRMS (ESI) calcd for $C_{25}H_{30}N_2O_2$ [M+H]$^+$ 391.2380, found 391.2379.

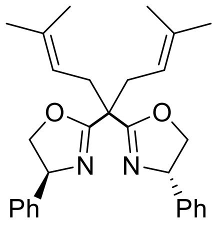

L8

(4*S*,4'*S*)-2,2'-(2,8-dimethylnona-2,7-diene-5,5-diyl)bis(4-phenyl-4,5-dihydrooxazole) (L8): Colorless oil (54% yield). ^{1}H NMR (400 MHz, Chloroform-*d*) δ 7.36 – 7.30 (m, 4H), 7.29 – 7.25 (m, 6H), 5.26 (dd, *J* = 10.0, 8.0 Hz, 2H), 5.23 – 5.17 (m, 2H), 4.65 (dd, *J* = 10.0, 8.0 Hz, 2H), 4.07 (d, *J* = 8.0 Hz, 2H), 2.88 (dd, *J* = 14.8, 8.0 Hz, 2H), 2.79 (dd, *J* = 14.8, 6.8 Hz, 2H), 1.75 (s, 6H), 1.63 (s, 6H);
^{13}C NMR (101 MHz, $CDCl_3$) δ 168.7, 142.4, 135.1, 128.7, 127.5, 126.8, 118.6, 75.2, 69.7, 46.5, 31.3, 26.2, 18.0. HRMS (ESI) calcd for $C_{29}H_{34}N_2O_2$ [M+H]$^+$ 443.2693, found 443.2701.

Supplementary Note 2

Screening of the Ligands

General Procedure: A flame-dried Schlenk tube equipped with a magnetic stirring bar, was charged with a mixture of $Cu(OTf)_2$ (3.6 mg, 10 mol%), ligand (15 mol%) and 4Å MS (40 mg). After being evacuated and backfilled with nitrogen for three times, THF (1 mL) was added to the Schlenk tube and the mixture was stirred at room temperature under a N_2 atmosphere for 1 h. Imine **1a** (0.1 mmol) and EtOH **2a** (1.0 mmol) was added sequentially. The reaction mixture was allowed to stir under a N_2 atmosphere at room temperature for 24 h. When the reaction was completed, THF was evaporated in vacuo and the residue was purified by flash silica gel column chromatography (PE/EtOAc = 10/1) to give product **3a**.

Supplementary Table 1. Optimization of the Reaction Ligands[a]

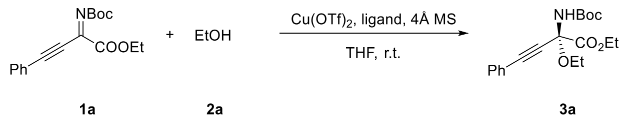

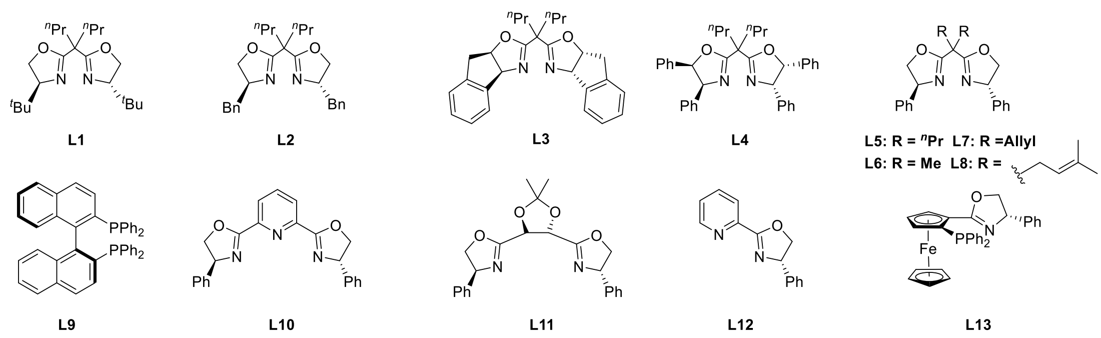

entry	ligand	yield (%)[b]	ee (%)[c]
1	**L1**	30	7
2	**L2**	75	-24
3	**L3**	9	*rac.*
4	**L4**	21	37
5	**L5**	46	66
6	**L6**	82	60
7	**L7**	68	51
8	**L8**	88	71
9[d]	**L5**	82	73
10[d]	**L6**	90	65
11[d]	**L7**	88	68
12[d]	**L8**	94	84
13	**L9**	55	5
14	**L10**	31	3
15	**L11**	74	2
16	**L12**	85	27
17	**L13**	38	47

[a] Conditions: **1a** (0.1 mmol), **2a** (1.0 mmol), $Cu(OTf)_2$ (10 mol%), ligand (15 mol%), 4Å MS (40 mg), THF (1.0 mL), r.t., 24 h. [b] Isolated yield. [c] Determined by chiral HPLC. [d] $Cu(BF_4)_2$•H_2O (10 mol%) was used instead of $Cu(OTf)_2$ (10 mol%).

Screening of the Coppers

General Procedure: A flame-dried Schlenk tube equipped with a magnetic stirring bar, was charged with a mixture of copper (10 mol%), **L8** (15 mol%) and 4Å MS (40 mg). After being evacuated and backfilled with nitrogen for three times, THF (1 mL) was added to the Schlenk tube and the mixture was stirred at room temperature under a N_2 atmosphere for 1 h. Imine **1a** (0.1 mmol) and EtOH **2a** (1.0 mmol) was added sequentially. The reaction mixture was allowed to stir under a N_2 atmosphere at room temperature for 24 h. When the reaction was completed,

THF was evaporated in vacuo and the residue was purified by flash silica gel column chromatography (PE/EtOAc = 10/1) to give product **3a**.

Supplementary Table 2. Optimization of the Reaction Coppers[a]

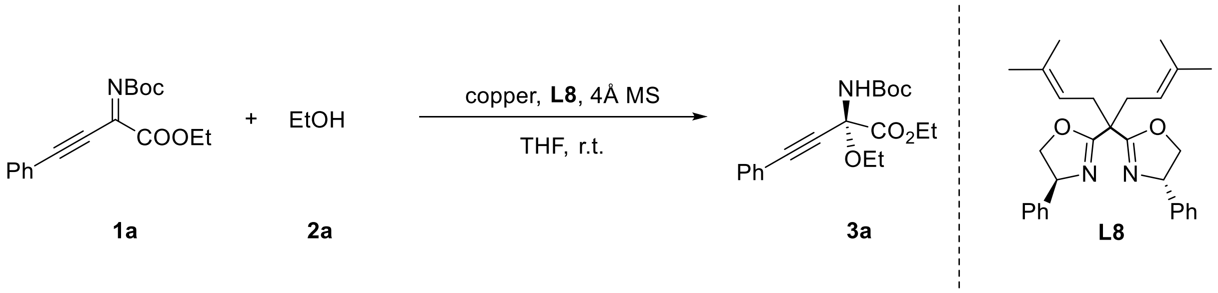

entry	copper	yield (%)[b]	ee (%)[c]
1	$Cu(OTf)_2$	88	71
2	$Cu(OAc)_2$	70	7
3	$Cu(acac)_2$	90	2
4	$Cu(ClO_4)_2$•$6H_2O$	81	70
5	$Cu(TMHD)_2$	86	1
6	$Cu(BF_4)_2$•H_2O	94	84
7	Copper(II) Hexafluoro-2,4-Pentanedionate	41	6
8	$Cu(Me_3CO_2)_2$	45	2
9	CuTc	94	6
10	$Cu(CH_3CN)_4OTf$	83	70
11	$Cu(CH_3CN)_4BF_4$	82	71
12	CuCl	69	20
13	CuBr	73	25
14	$Cu(MeCN)_4PF_6$	78	75
15	CuI	65	32

[a] Conditions: **1a** (0.1 mmol), **2a** (1.0 mmol), copper (10 mol%), **L8** (15 mol%), 4Å MS (40 mg), THF (1.0 mL), r.t., 24 h. [b] Isolated yield. [c] Determined by chiral HPLC.

Screening of the Solvents

General Procedure: A flame-dried Schlenk tube equipped with a magnetic stirring bar, was charged with a mixture of $Cu(BF_4)_2$•H_2O (10 mol%), **L8** (15 mol%) and 4Å MS (40 mg). After being evacuated and backfilled with nitrogen for three times, solvent (1 mL) was added to the Schlenk tube and the mixture was stirred at room temperature under a N_2 atmosphere for 1 h. Imine **1a** (0.1 mmol) and EtOH **2a** (1.0 mmol) was added sequentially. The reaction mixture was allowed to stir under a N_2 atmosphere at room temperature for 24 h. When the reaction was completed, solvent was evaporated in vacuo and the residue was purified by flash silica gel column chromatography (PE/EtOAc = 10/1) to give product.

Supplementary Table 3. Optimization of the Reaction Solvents[a]

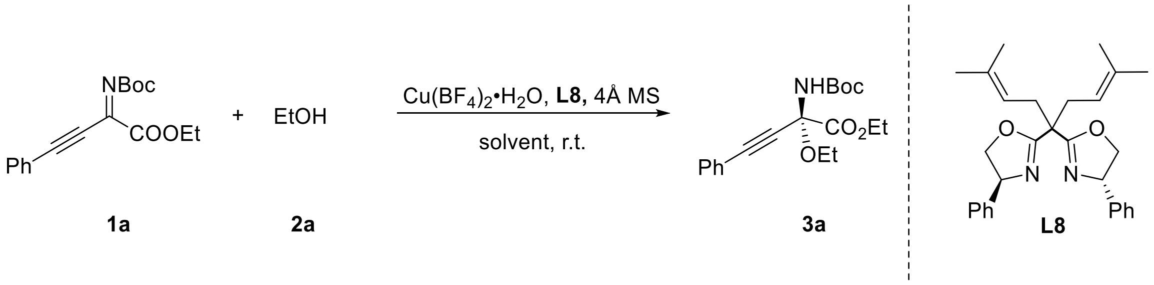

entry	solvent	yield (%)[b]	ee (%)[c]
1	1, 4-dioxane	69	57
2	MeCN	78	88
3	DMF	17	70
4	DCM	86	83
5	DCE	83	80
6	toluene	83	63
7	$CHCl_3$	83	84
8	THF	94	84
9	acetone	84	94
10[d]	acetone	88	96
11[d, e]	acetone	90	96

[a] Conditions: **1a** (0.1 mmol), **2a** (1.0 mmol), copper (10 mol%), **L8** (15 mol%), 4Å MS (40 mg), solvent (1.0 mL), r.t., 24 h. [b] Isolated yield. [c] Determined by chiral HPLC. [d] The reaction was conducted at 0 °C. [e] 0.2 mmol of **2a** was added.

Other Attempts of different substrates

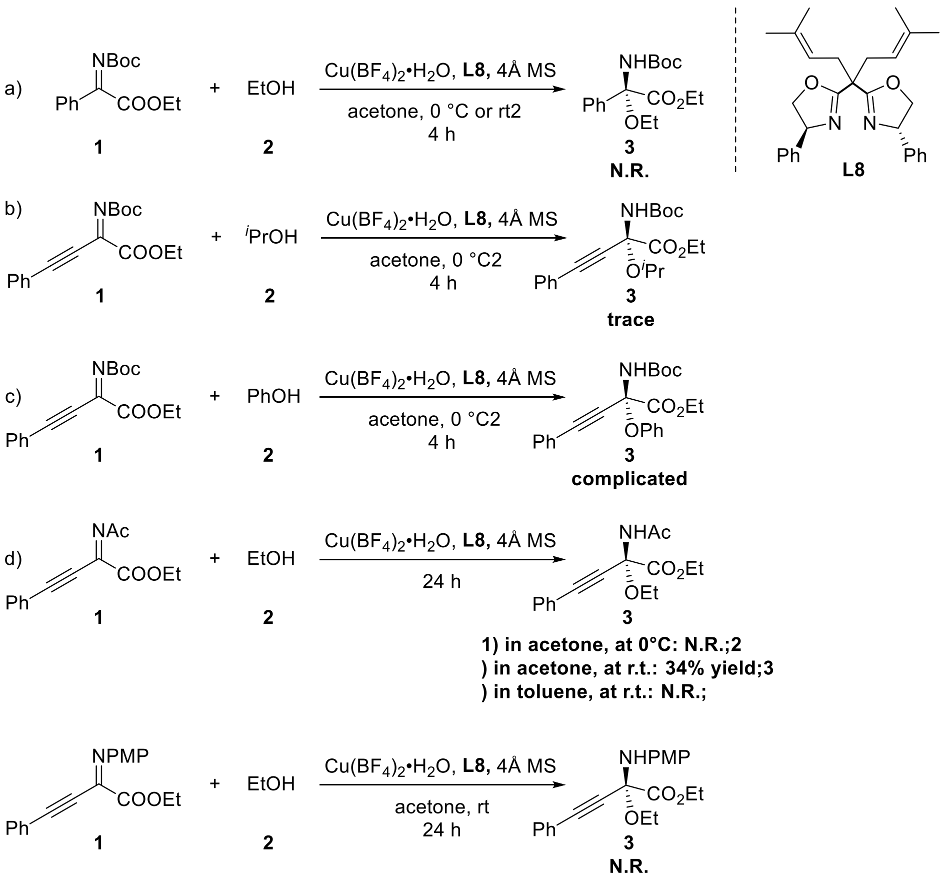

Supplementary Fig. 1. Other Attempts. [a]

[a] Conditions: **1a** (0.1 mmol), **2a** (0.2 mmol), $Cu(BF_4)_2$•H_2O (10 mol%), **L8** (15 mol%), 4Å MS (40 mg), acetone (1.0 mL), 0 °C, 24 h. [b] Toluene was used as solvent instead of acetone. [c] The reaction was conducted at rt.

Supplementary Note 3

General Procedure for the Copper-Bis(oxazoline) Catalyzed Asymmetric Addition of Alcohols to Linear *β*, *γ*-Alkynyl-*α*-Imino Esters

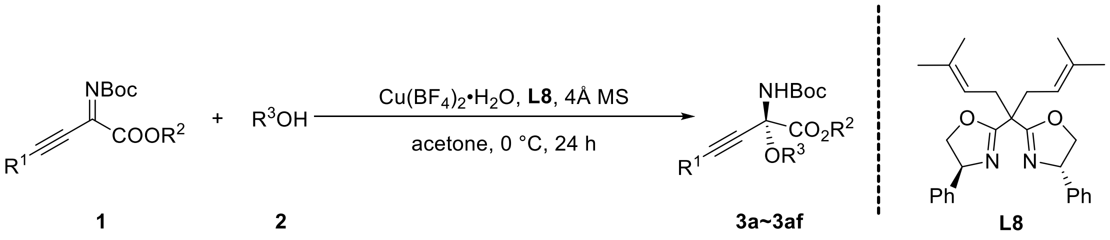

General Procedure: A flame-dried Schlenk tube equipped with a magnetic stirring bar, was charged with a mixture of $Cu(BF_4)_2$•H_2O (10 mol%), **L2** (15 mol%) and 4Å MS (40 mg). After being evacuated and refilled with nitrogen for three times, acetone (1 mL) was added to the Schlenk tube and the mixture was stirred at room temperature under a N_2 atmosphere for 1 h. Imine **1** (0.1 mmol) and alcohol **2** (0.2 mmol) was added sequentially. The reaction mixture was allowed to stir under a N_2 atmosphere at 0 °C for 24 h. When the reaction was completed,

solvent was evaporated in vacuo and the residue was purified by flash silica gel column chromatography (PE/EtOAc = 10/1) to give product **3a~3ah**.

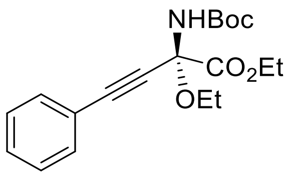

3a

Ethyl (*S*)-2-((*tert*-butoxycarbonyl)amino)-2-ethoxy-4-phenylbut-3-ynoate (3a): Pale yellow solid (31.2 mg, 90% total yield, purified by flash column chromatography, petroleum ether/EtOAc = 10/1). Mp: 57–58 °C; ^{1}H NMR (400 MHz, Chloroform-*d*) δ 7.47 – 7.44 (m, 2H), 7.39 – 7.30 (m, 3H), 5.86 (s, 1H), 4.44 – 4.27 (m, 2H), 3.90 – 3.81 (m, 1H), 3.79 – 3.69 (m, 1H), 1.46 (s, 9H), 1.35 (t, *J* = 7.2 Hz, 3H), 1.31 (t, *J* = 7.2 Hz, 3H); ^{13}C NMR (101 MHz, $CDCl_3$) δ 166.5, 152.9, 132.0, 129.3, 128.4, 121.1, 86.1, 83.0, 81.0, 80.2, 62.9, 60.4, 28.2, 15.3, 14.0; HRMS (ESI) calcd for $C_{19}H_{25}NO_5$ $[M+Na]^+$ 370.1625, found 370.1625; HPLC (Daicel Chiralcel IC-3, n-hexane/i-PrOH = 90/10, UV = 254 nm, flow rate = 1.0 mL/min) t_{R1} = 9.095 min (minor) and t_{R2} = 12.203 min (major), *ee* = 96%; $[\alpha]_D^{25}$ = +24.8 (*c* 0.3, CH_2Cl_2).

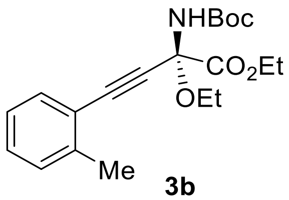

3b

Ethyl (*S*)-2-((*tert*-butoxycarbonyl)amino)-2-ethoxy-4-(o-tolyl)but-3-ynoate (3b): Pale yellow oil (33.2 mg, 92% total yield, purified by flash column chromatography, petroleum ether/EtOAc = 10/1). ^{1}H NMR (400 MHz, Chloroform-*d*) δ 7.41 (dd, *J* = 7.6, 1.2 Hz, 1H), 7.28 – 7.24 (m, 1H), 7.20 (d, *J* = 7.6 Hz, 1H), 7.14 (d, *J* = 7.6 Hz, 1H), 5.86 (s, 1H), 4.44 – 4.27 (m, 2H), 3.93 – 3.83 (m, 1H), 3.81 – 3.71 (m, 1H), 2.42 (s, 3H), 1.47 (s, 9H), 1.35 (t, *J* = 7.2 Hz, 3H), 1.31 (t, *J* = 7.2 Hz, 3H); ^{13}C NMR (101 MHz, $CDCl_3$) δ 166.6, 152.9, 140.9, 132.3, 129.5, 129.3, 125.6, 121.0, 86.8, 85.2, 80.9, 80.4, 62.9, 60.6, 28.2, 20.6, 15.3, 14.0; HRMS (ESI) calcd for $C_{20}H_{27}NO_5$ $[M+Na]^+$ 384.1781, found 384.1784; HPLC (Daicel Chiralcel IC-3, n-hexane/i-PrOH = 90/10, UV = 254 nm, flow rate = 1.0 mL/min) t_{R1} = 9.414 min (minor) and t_{R2} = 11.128 min (major), *ee* = 93%; $[\alpha]_D^{25}$ = +8.0 (*c* 0.2, CH_2Cl_2).

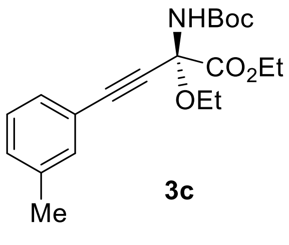

3c

Ethyl (*S*)-2-((*tert*-butoxycarbonyl)amino)-2-ethoxy-4-(m-tolyl)but-3-ynoate (3c): Pale yellow oil (34.3 mg, 95% total yield, purified by flash column chromatography, petroleum

ether/EtOAc = 10/1). ^{1}H NMR (400 MHz, Chloroform-*d*) δ 7.28 (s, 1H), 7.25 (s, 1H), 7.23 – 7.15 (m, 2H), 5.85 (s, 1H), 4.42 – 4.27 (m, 2H), 3.92 – 3.80 (m, 1H), 3.80 – 3.69 (m, 1H), 2.32 (s, 3H), 1.46 (s, 9H), 1.35 (d, *J* = 7.2 Hz, 3H), 1.30 (t, *J* = 7.2 Hz, 3H); ^{13}C NMR (101 MHz, $CDCl_3$) δ 166.5, 152.9, 138.1, 132.6, 130.2, 129.1, 128.3, 120.9, 86.4, 82.7, 81.0, 80.3, 62.9, 60.4, 28.2, 21.2, 15.3, 14.0; HRMS (ESI) calcd for $C_{20}H_{27}NO_5$ $[M+Na]^+$ 384.1781, found 384.1781; HPLC (Daicel Chiralcel IC-3, n-hexane/i-PrOH = 90/10, UV = 254 nm, flow rate = 1.0 mL/min) t_{R1} = 10.286 min (minor) and t_{R2} = 16.120 min (major), *ee* = 92%; $[\alpha]_D^{25}$ = +10.8 (*c* 1.2, CH_2Cl_2).

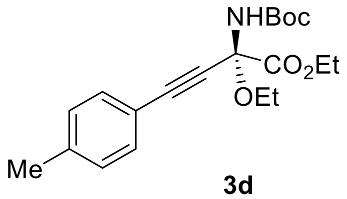

3d

Ethyl (*S*)-2-((*tert*-butoxycarbonyl)amino)-2-ethoxy-4-(*p*-tolyl)but-3-ynoate (3d): Pale yellow oil (35.8 mg, 99% total yield, purified by flash column chromatography, petroleum ether/EtOAc = 10/1). ^{1}H NMR (400 MHz, Chloroform-*d*) δ 7.34 (d, *J* = 8.4 Hz, 2H), 7.13 (d, *J* = 8.0 Hz, 2H), 5.84 (s, 1H), 4.42 – 4.27 (m, 3H), 3.89 – 3.82 (m, 1H), 3.78 – 3.70 (m, 1H), 2.35 (s, 3H), 1.46 (s, 9H), 1.34 (t, *J* = 7.2 Hz, 2H), 1.31 (t, *J* = 6.8 Hz, 2H); ^{13}C NMR (101 MHz, $CDCl_3$) δ 166.6, 152.9, 139.6, 131.9, 129.1, 118.0, 86.3, 82.4, 80.9, 80.3, 62.9, 60.3, 28.2, 21.6, 15.3, 14.0; HRMS (ESI) calcd for $C_{20}H_{27}NO_5$ $[M+Na]^+$ 384.1781, found 384.1779; HPLC (Daicel Chiralcel IC-3, n-hexane/i-PrOH = 90/10, UV = 254 nm, flow rate = 1.0 mL/min) t_{R1} = 11.971 min (minor) and t_{R2} = 17.967 min (major), *ee* = 91%; $[\alpha]_D^{25}$ = +27.2 (*c* 0.7, CH_2Cl_2).

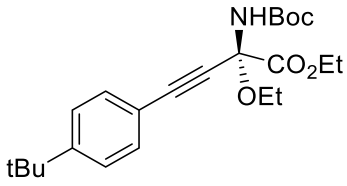

3e

Ethyl (*S*)-2-((*tert*-butoxycarbonyl)amino)-4-(4-(*tert*-butyl)phenyl)-2-ethoxybut-3-ynoate (3e): Pale yellow oil (38.7 mg, 96% total yield, purified by flash column chromatography, petroleum ether/EtOAc = 10/1). ^{1}H NMR (400 MHz, Chloroform-*d*) δ 7.39 (d, *J* = 8.6 Hz, 2H), 7.38 – 7.31 (m, 2H), 5.86 (s, 1H), 4.46 – 4.26 (m, 2H), 3.92 – 3.81 (m, 1H), 3.80 – 3.68 (m, 1H), 1.46 (s, 9H), 1.34 (t, *J* = 7.2 Hz, 3H), 1.31 (t, *J* = 7.2 Hz, 3H), 1.30 (s, 9H); ^{13}C NMR (101 MHz, $CDCl_3$) δ 166.5, 152.9, 152.7, 131.7, 125.4, 118.0, 86.3, 82.3, 89.0, 80.3, 62.8, 60.3, 34.9, 31.1, 28.2, 15.3, 14.0; HRMS (ESI) calcd for $C_{23}H_{33}NO_5$ $[M+Na]^+$ 426.2251, found 426.2251; HPLC (Daicel Chiralcel IC-3, n-hexane/i-PrOH = 90/10, UV = 254 nm, flow rate = 1.0 mL/min) t_{R1} = 13.509 min (minor) and t_{R2} = 22.842 min (major), *ee* = 92%; $[\alpha]_D^{25}$ = +19.3 (*c* 1.4, CH_2Cl_2).

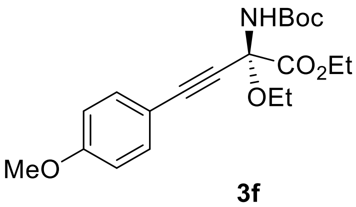

3f

Ethyl (*S*)-2-((*tert*-butoxycarbonyl)amino)-2-ethoxy-4-(4-methoxyphenyl)but-3-ynoate (3f): Pale yellow oil (37.4 mg, 99% total yield, purified by flash column chromatography, petroleum ether/EtOAc = 10/1). ^{1}H NMR (400 MHz, Chloroform-*d*) δ 7.39 (dt, *J* = 8.8, 2.0 Hz, 2H), 6.84 (dt, *J* = 8.8, 2.0 Hz, 2H), 5.85 (s, 1H), 4.42 – 4.27 (m, 2H), 3.90 – 3.82 (m, 1H), 3.81 (s, 3H), 3.77 – 3.70 (m, 1H), 1.46 (s, 9H), 1.34 (t, *J* = 7.2 Hz, 3H), 1.31 (t, *J* = 7.2 Hz, 3H); ^{13}C NMR (101 MHz, $CDCl_3$) δ 166.6, 160.3, 152.9, 133.5, 114.0, 113.1, 86.2, 81.8, 80.9, 80.3, 62.8, 60.3, 55.3, 28.2, 15.3, 14.0; HRMS (ESI) calcd for $C_{20}H_{27}NO_6$ $[M+Na]^+$ 400.1731, found 400.1730; HPLC (Daicel Chiralcel IC-3, n-hexane/i-PrOH = 90/10, UV = 254 nm, flow rate = 1.0 mL/min) t_{R1} = 15.809 min (minor) and t_{R2} = 18.694 min (major), *ee* = 94%; $[\alpha]_D^{25}$ = +27.1 (*c* 0.9, CH_2Cl_2).

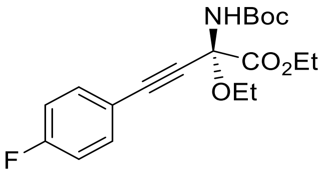

3g

Ethyl (*S*)-2-((*tert*-butoxycarbonyl)amino)-2-ethoxy-4-(4-fluorophenyl)but-3-ynoate (3g): Pale yellow oil (35.1 mg, 96% total yield, purified by flash column chromatography, petroleum ether/EtOAc = 10/1). ^{1}H NMR (400 MHz, Chloroform-*d*) δ 7.47 – 7.42 (m, 2H), 7.07 – 6.98 (m, 2H), 5.87 (s, 1H), 4.43 – 4.27 (m, 2H), 3.90 – 3.79 (m, 1H), 3.78 – 3.67 (m, 1H), 1.46 (s, 9H), 1.35 (t, *J* = 7.2 Hz, 3H), 1.31 (t, *J* = 7.2 Hz, 3H); ^{13}C NMR (101 MHz, $CDCl_3$) δ 166.5, 163.0 (d, *J* = 252.2 Hz), 152.9, 134.0 (d, *J* = 8.6 Hz), 117.2 (d, *J* = 3.3 Hz), 115.7 (d, *J* = 22.1 Hz), 85.0, 82.9, 81.0, 80.2, 63.0, 60.4, 28.2, 15.2, 14.0; ^{19}F NMR (376 MHz, $CDCl_3$) δ -109.1; HRMS (ESI) calcd for $C_{19}H_{24}FNO_5$ $[M+Na]^+$ 388.1531, found 388.1536; HPLC (Daicel Chiralcel IC-3, n-hexane/i-PrOH = 90/10, UV = 254 nm, flow rate = 1.0 mL/min) t_{R1} = 9.198 min (minor) and t_{R2} = 12.211 min (major), *ee* = 90%; $[\alpha]_D^{25}$ = +16.7 (*c* 1.2, CH_2Cl_2).

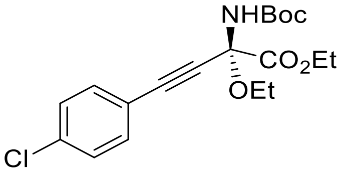

3h

Ethyl (*S*)-2-((*tert*-butoxycarbonyl)amino)-4-(4-chlorophenyl)-2-ethoxybut-3-ynoate (3h): Pale yellow oil (36.3 mg, 95% total yield, purified by flash column chromatography, petroleum ether/EtOAc = 10/1). ^{1}H NMR (400 MHz, Chloroform-*d*) δ 7.39 (dt, *J* = 8.4, 2.4 Hz, 2H), 7.30 (dt, *J* = 8.4, 2.4 Hz, 2H), 5.88 (s, 1H), 4.42 – 4.28 (m, 2H), 3.86 – 3.79 (m, 1H), 3.75 – 3.68 (m, 1H), 1.46 (s, 9H), 1.35 (t, *J* = 7.2 Hz, 3H), 1.31 (t, *J* = 7.2 Hz, 3H); ^{13}C NMR (101 MHz, $CDCl_3$)

δ 166.4, 152.8, 135.6, 133.2, 128.8, 119.6, 84.9, 84.0, 81.0, 80.2, 63.0, 60.5, 28.2, 15.2, 14.0; HRMS (ESI) calcd for $C_{19}H_{24}ClNO_5$ $[M+Na]^+$ 404.1235, found 404.1239; HPLC (Daicel Chiralcel IC-3, n-hexane/i-PrOH = 90/10, UV = 254 nm, flow rate = 1.0 mL/min) t_{R1} = 9.184 min (minor) and t_{R2} = 12.713 min (major), *ee* = 94%; $[\alpha]_D^{25}$ = +20.8 (*c* 0.9, CH_2Cl_2).

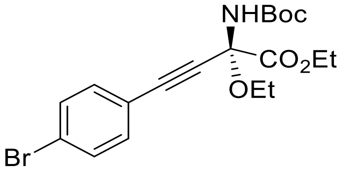

3i

Ethyl (*S*)-4-(4-bromophenyl)-2-((*tert*-butoxycarbonyl)amino)-2-ethoxybut-3-ynoate (3i): Yellow oil (35.0 mg, 82% total yield, purified by flash column chromatography, petroleum ether/EtOAc = 10/1). ^1H NMR (400 MHz, Chloroform-*d*) δ 7.46 (d, *J* = 8.4 Hz, 2H), 7.32 (d, *J* = 8.5 Hz, 2H), 5.89 (s, 1H), 4.43 – 4.27 (m, 2H), 3.87 – 3.77 (m, 1H), 3.76 – 3.66 (m, 1H), 1.46 (s, 9H), 1.35 (t, *J* = 7.2 Hz, 3H), 1.30 (t, *J* = 7.2 Hz, 3H); ^{13}C NMR (101 MHz, $CDCl_3$) δ 166.4, 152.9, 133.4, 131.7, 123.7, 120.1, 84.9, 84.2, 81.1, 80.2, 63.0, 60.5, 28.2, 15.2, 14.0; HRMS (ESI) calcd for $C_{19}H_{24}BrNO_5$ $[M+Na]^+$ 448.0730, found 448.0735; HPLC (Daicel Chiralcel IC-3, n-hexane/i-PrOH = 90/10, UV = 254 nm, flow rate = 1.0 mL/min) t_{R1} = 9.197 min (minor) and t_{R2} = 12.823 min (major), *ee* = 93%; $[\alpha]_D^{25}$ = +18.1 (*c* 1.1, CH_2Cl_2).

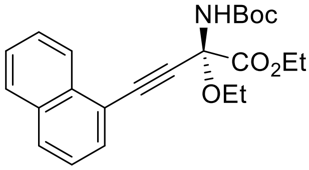

3j

Ethyl (*S*)-2-((*tert*-butoxycarbonyl)amino)-2-ethoxy-4-(naphthalen-1-yl)but-3-ynoate (3j): Yellow oil (32.2 mg, 81% total yield, purified by flash column chromatography, petroleum ether/EtOAc = 10/1). ^1H NMR (400 MHz, Chloroform-*d*) δ 8.29 (d, *J* = 8.4 Hz, 1H), 7.90 – 7.82 (m, 2H), 7.73 – 7.67 (m, 1H), 7.60 – 7.49 (m, 2H), 7.46 – 7.39 (m, 1H), 6.00 (s, 1H), 4.48 – 4.32 (m, 2H), 4.00 – 3.90 (m, 1H), 3.88 – 3.77 (m, 1H), 1.49 (s, 9H), 1.39 (t, *J* = 7.2 Hz, 3H), 1.34 (t, *J* = 7.2 Hz, 3H); ^{13}C NMR (101 MHz, $CDCl_3$) δ 166.7, 153.0, 133.4, 133.0, 131.2, 129.8, 128.3, 127.1, 126.6, 126.0, 125.0, 118.8, 87.8, 84.4, 81.0, 80.5, 63.0, 60.8, 28.2, 15.3, 14.1; HRMS (ESI) calcd for $C_{23}H_{27}NO_5$ $[M+Na]^+$ 420.1781, found 420.1778; HPLC (Daicel Chiralcel IC-3, n-hexane/i-PrOH = 90/10, UV = 254 nm, flow rate = 1.0 mL/min) t_{R1} = 10.305 min (minor) and t_{R2} = 15.554 min (major), *ee* = 91%; $[\alpha]_D^{25}$ = +8.1 (*c* 1.0, CH_2Cl_2).

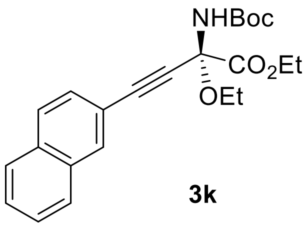

3k

Ethyl (*S*)-2-((*tert*-butoxycarbonyl)amino)-2-ethoxy-4-(naphthalen-2-yl)but-3-ynoate (3k): Yellow solid (38.6 mg, 97% total yield, purified by flash column chromatography, petroleum ether/EtOAc = 10/1). Mp: 65-66 °C; ^{1}H NMR (400 MHz, Chloroform-*d*) δ 8.00 (s, 1H), 7.83 – 7.77 (m, 3H), 7.53 – 7.50 (m, 2H), 7.48 (dd, *J* = 8.4, 1.6 Hz, 1H), 5.91 (s, 1H), 4.47 – 4.28 (m, 2H), 3.94 – 3.86 (m, 1H), 3.82 – 3.74 (m, 1H), 1.48 (s, 9H), 1.37 (t, *J* = 7.2 Hz, 3H), 1.34 (t, *J* = 7.2 Hz, 3H); ^{13}C NMR (101 MHz, $CDCl_3$) δ 166.5, 152.9, 133.2, 132.7, 132.4, 128.1, 128.1, 127.9, 127.8, 127.2, 126.7, 118.4, 86.5, 83.3, 81.0, 80.3, 63.0, 60.5, 28.2, 15.3, 14.0; HRMS (ESI) calcd for $C_{23}H_{27}NO_5$ [M+Na]$^+$ 420.1781, found 420.1771; HPLC (Daicel Chiralcel IC-3, n-hexane/i-PrOH = 90/10, UV = 254 nm, flow rate = 1.0 mL/min) t_{R1} = 13.617 min (minor) and t_{R2} = 21.948 min (major), *ee* = 93%; $[\alpha]_D^{25}$ = +28.0 (*c* 1.7, CH_2Cl_2).

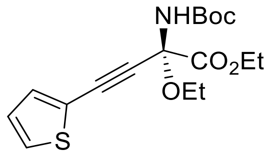

3l

Ethyl (*S*)-2-((*tert*-butoxycarbonyl)amino)-2-ethoxy-4-(thiophen-2-yl)but-3-ynoate (3l): Yellow solid (33.2 mg, 94% total yield, purified by flash column chromatography, petroleum ether/EtOAc = 10/1). Mp: 86-87 °C; ^{1}H NMR (400 MHz, Chloroform-*d*) δ 7.31 (dd, *J* = 5.2, 0.8 Hz, 1H), 7.28 (dd, *J* = 3.6, 1.2 Hz, 1H), 6.99 (dd, *J* = 5.2, 3.6 Hz, 1H), 5.86 (s, 1H), 4.42 – 4.28 (m, 2H), 3.86 – 3.79 (m, 1H), 3.76 – 3.68 (m, 1H), 1.46 (s, 10H), 1.35 (t, *J* = 7.2 Hz, 3H), 1.31 (t, *J* = 7.2 Hz, 4H); ^{13}C NMR (101 MHz, $CDCl_3$) δ 166.3, 152.8, 133.5, 128.4, 127.0, 120.9, 86.8, 81.1, 80.3, 79.6, 63.0, 60.5, 28.2, 15.2, 14.0; HRMS (ESI) calcd for $C_{17}H_{23}NO_5S$ [M+Na]$^+$ 376.1189, found 376.1190; HPLC (Daicel Chiralcel IC-3, n-hexane/i-PrOH = 90/10, UV = 254 nm, flow rate = 1.0 mL/min) t_{R1} = 10.305 min (minor) and t_{R2} = 12.970 min (major), *ee* = 94%; $[\alpha]_D^{25}$ = +18.2 (*c* 1.1, CH_2Cl_2).

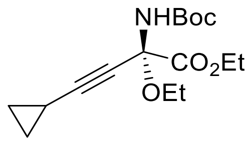

3m

Ethyl (*S*)-2-((*tert*-butoxycarbonyl)amino)-4-cyclopropyl-2-ethoxybut-3-ynoate (3m): Yellow solid (24.0 mg, 77% total yield, purified by flash column chromatography, petroleum ether/EtOAc = 10/1). Mp: 74-75 °C; ^{1}H NMR (400 MHz, Chloroform-*d*) δ 5.68 (s, 1H), 4.40 – 4.22 (m, 2H), 3.80 – 3.68 (m, 1H), 3.68 – 3.56 (m, 1H), 1.43 (s, 9H), 1.32 (t, *J* = 7.2 Hz, 3H), 1.25 (t, *J* = 7.2 Hz, 3H), 1.29 – 1.22 (m, 1H), 0.83 – 0.78 (m, 2H), 0.75 – 0.69 (m, 2H); ^{13}C NMR (101 MHz, $CDCl_3$) δ 167.4, 153.5, 91.3, 81.6, 80.5, 70.1, 63.4, 60.7, 28.9, 15.9, 14.7, 9.1, 9.1, 0.7; HRMS (ESI) calcd for $C_{16}H_{25}NO_5$ [M+Na]$^+$ 334.1625, found 334.1626; HPLC (Daicel Chiralcel IC-3, n-hexane/i-PrOH = 90/10, UV = 220 nm, flow rate = 1.0 mL/min) t_{R1} = 13.506 min (major) and t_{R2} = 15.515 min (minor), *ee* = 94%; $[\alpha]_D^{25}$ = +16.5 (*c* 0.3, CH_2Cl_2).

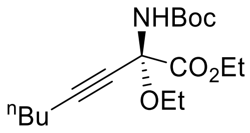

3n

Ethyl (*S*)-2-((*tert*-butoxycarbonyl)amino)-2-ethoxyoct-3-ynoate (3n): Pale yellow oil (23.9 mg, 73% total yield, purified by flash column chromatography, petroleum ether/EtOAc = 10/1). ^{1}H NMR (400 MHz, Chloroform-*d*) δ 5.70 (s, 1H), 4.38 – 4.24 (m, 2H), 3.80 – 3.72 (m, 1H), 3.68 –3.61 (m, 1H), 2.23 (t, *J* = 7.2 Hz, 2H), 1.53 – 1.36 (m, 4H), 1.44 (s, 9H), 1.32 (t, *J* = 7.2 Hz, 3H), 1.28 (t, *J* = 7.2 Hz, 3H), 0.90 (t, *J* = 7.6 Hz, 3H); ^{13}C NMR (101 MHz, $CDCl_3$) δ 166.8, 152.9, 87.7, 80.8, 79.8, 74.5, 62.6, 60.0, 30.1, 28.2, 21.9, 18.3, 15.2, 14.0, 13.5; HRMS (ESI) calcd for $C_{17}H_{29}NO_5$ $[M+Na]^+$ 350.1938, found 350.1945; HPLC (Daicel Chiralcel IC-3, n-hexane/i-PrOH = 90/10, UV = 220 nm, flow rate = 1.0 mL/min) t_{R1} = 10.793 min (minor) and t_{R2} = 11.899 min (major), *ee* = 96%; $[\alpha]_D^{25}$ = +7.9 (*c* 0.8, CH_2Cl_2).

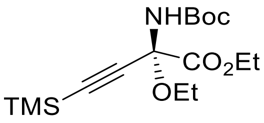

3o

Ethyl (*S*)-2-((*tert*-butoxycarbonyl)amino)-2-ethoxy-4-(trimethylsilyl)but-3-ynoate (3o): Colorless oil (34.0 mg, 99% total yield, purified by flash column chromatography, petroleum ether/EtOAc = 10/1). ^{1}H NMR (400 MHz, Chloroform-*d*) δ 5.74 (s, 1H), 4.39 – 4.26 (m, 2H), 3.81 – 3.74 (m, 1H), 3.69 – 3.62 (m, 1H), 1.44 (s, 9H), 1.32 (t, *J* = 7.2 Hz, 3H), 1.28 (t, *J* = 7.2 Hz, 3H), 0.18 (s, 9H); ^{13}C NMR (101 MHz, $CDCl_3$) δ 166.3, 152.8, 98.1, 92.0, 81.0, 79.8, 62.8, 60.2, 28.1, 15.2, 13.9, -0.5; HRMS (ESI) calcd for $C_{16}H_{29}NO_5Si$ $[M+Na]^+$ 366.1707, found 366.1713; HPLC (Daicel Chiralcel AD-H, n-hexane/i-PrOH = 95/5, UV = 220 nm, flow rate = 1.0 mL/min) t_{R1} = 5.107 min (major) and t_{R2} = 6.739 min (minor), *ee* = 95%; $[\alpha]_D^{25}$ = +10.0 (*c* 1.0, CH_2Cl_2).

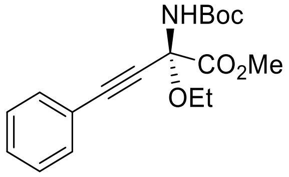

3p

Methyl (*S*)-2-((*tert*-butoxycarbonyl)amino)-2-ethoxy-4-phenylbut-3-ynoate (3p): Pale yellow solid (23.3 mg, 70% total yield, purified by flash column chromatography, petroleum ether/EtOAc = 10/1). Mp: 71-72 °C; ^{1}H NMR (400 MHz, Chloroform-*d*) δ 7.53 – 7.40 (m, 2H), 7.40 – 7.27 (m, 3H), 5.85 (s, 1H), 3.89 (s, 3H), 3.88 – 3.82 (m, 1H), 3.80 – 3.70 (m, 1H), 1.46 (s, 9H), 1.32 (t, *J* = 7.2 Hz, 3H); ^{13}C NMR (101 MHz, $CDCl_3$) δ 167.2, 152.9, 132.0, 129.4, 128.4, 121.0, 86.3, 82.8, 81.1, 80.3, 60.4, 53.8, 28.2, 15.3; HRMS (ESI) calcd for $C_{18}H_{23}NO_5$ $[M+Na]^+$

356.1468, found 356.1467; HPLC (Daicel Chiralcel IC-3, n-hexane/i-PrOH = 90/10, UV = 254 nm, flow rate = 1.0 mL/min) t_{R1} = 11.096 min (minor) and t_{R2} = 15.105 min (major), *ee* = 93%; $[\alpha]_D^{25}$ = +13.6 (*c* 0.8, CH_2Cl_2).

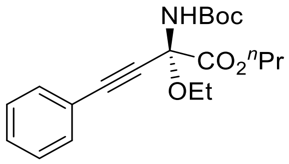

3q

Propyl (*S*)-2-((*tert*-butoxycarbonyl)amino)-2-ethoxy-4-phenylbut-3-ynoate (3q): Pale yellow solid (34.3 mg, 95% total yield, purified by flash column chromatography, petroleum ether/EtOAc = 10/1). Mp: 57-58 °C; ^{1}H NMR (400 MHz, Chloroform-*d*) δ 7.47 – 7.44 (m, 2H), 7.38 – 7.30 (m, 3H), 5.88 (s, 1H), 4.29 – 4.20 (m, 2H), 3.89 – 3.82 (m, 1H), 3.78 – 3.70 (m, 1H), 1.75 (h, *J* = 7.2 Hz, 2H), 1.46 (s, 9H), 1.31 (t, *J* = 7.2 Hz, 3H), 0.98 (t, *J* = 7.6 Hz, 3H); ^{13}C NMR (101 MHz, $CDCl_3$) δ 166.6, 152.9, 132.0, 129.3, 128.4, 121.2, 86.0, 83.1, 80.9, 80.2, 68.4, 60.5, 28.2, 21.9, 15.3, 10.3; HRMS (ESI) calcd for $C_{20}H_{27}NO_5$ $[M+Na]^+$ 384.1781, found 384.1785; HPLC (Daicel Chiralcel IC-3, n-hexane/i-PrOH = 90/10, UV = 254 nm, flow rate = 1.0 mL/min) t_{R1} = 9.517 min (minor) and t_{R2} = 12.979 min (major), *ee* = 90%; $[\alpha]_D^{25}$ = +18.7 (*c* 1.3, CH_2Cl_2).

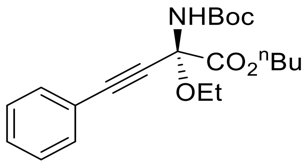

3r

Butyl (*S*)-2-((*tert*-butoxycarbonyl)amino)-2-ethoxy-4-phenylbut-3-ynoate (3r): Pale yellow solid (34.2 mg, 91% total yield, purified by flash column chromatography, petroleum ether/EtOAc = 10/1). Mp: 48-49 °C; ^{1}H NMR (400 MHz, Chloroform-*d*) δ 7.47 – 7.44 (m, 2H), 7.39 – 7.30 (m, 3H), 5.88 (s, 1H), 4.34 – 4.24 (m, 2H), 3.89 – 3.81 (m, 1H), 3.78 – 3.70 (m, 1H), 1.74 – 1.67 (m, 2H), 1.46 (s, 9H), 1.46 – 1.38 (m, 2H), 1.31 (t, *J* = 7.2 Hz, 3H), 0.92 (t, *J* = 7.2 Hz, 3H); ^{13}C NMR (101 MHz, $CDCl_3$) δ 166.6, 152.9, 132.0, 129.3, 128.3, 121.2, 86.0, 83.1, 80.9, 80.2, 66.7, 60.4, 30.5, 28.2, 19.0, 15.3, 13.7; HRMS (ESI) calcd for $C_{21}H_{29}NO_5$ $[M+Na]^+$ 398.1938, found 398.1938; HPLC (Daicel Chiralcel IC-3, n-hexane/i-PrOH = 90/10, UV = 254 nm, flow rate = 1.0 mL/min) t_{R1} = 9.092 min (minor) and t_{R2} = 12.386 min (major), *ee* = 91%; $[\alpha]_D^{25}$ = +18.1 (*c* 1.1, CH_2Cl_2).

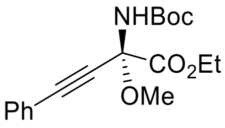

3s

Ethyl (*S*)-2-((*tert*-butoxycarbonyl)amino)-2-methoxy-4-phenylbut-3-ynoate (3s): Yellow oil (27.7 mg, 83% total yield, purified by flash column chromatography, petroleum ether/EtOAc =

10/1). ^{1}H NMR (400 MHz, Chloroform-*d*) δ 7.50 – 7.43 (m, 2H), 7.40 – 7.30 (m, 3H), 5.86 (s, 1H), 4.44 – 4.29 (m, 2H), 3.54 (s, 3H), 1.47 (s, 9H), 1.35 (t, *J* = 7.2 Hz, 3H); ^{13}C NMR (101 MHz, $CDCl_3$) δ 166.2, 152.9, 132.0, 129.4, 128.4, 120.9, 86.7, 82.2, 81.2, 80.8, 63.0, 51.7, 28.2, 14.0; HRMS (ESI) calcd for $C_{18}H_{23}NO_5$ [M+Na]$^+$ 356.1468, found 356.1472; HPLC (Daicel Chiralcel IC-3, n-hexane/i-PrOH = 90/10, UV = 254 nm, flow rate = 1.0 mL/min) t_{R1} = 25.710 min (minor) and t_{R2} = 29.317 min (major), *ee* = 90%; $[\alpha]_D^{25}$ = +29.2 (*c* 0.9, CH_2Cl_2).

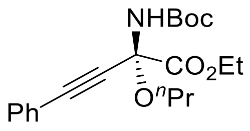

3t

Ethyl (*S*)-2-((*tert*-butoxycarbonyl)amino)-4-phenyl-2-propoxybut-3-ynoate (3t): Pale yellow oil (29.3 mg, 81% total yield, purified by flash column chromatography, petroleum ether/EtOAc = 10/1). ^{1}H NMR (400 MHz, Chloroform-*d*) δ 7.47 – 7.44 (m, 2H), 7.39 – 7.30 (m, 3H), 5.87 (s, 1H), 4.42 – 4.28 (m, 2H), 3.78 – 3.72 (m, 1H), 3.64 – 3.58 (m, 1H), 1.70 (h, *J* = 7.2 Hz, 2H), 1.46 (s, 9H), 1.35 (t, *J* = 7.2 Hz, 3H), 0.96 (t, *J* = 7.2 Hz, 3H); ^{13}C NMR (101 MHz, $CDCl_3$) δ 166.5, 152.9, 132.0, 129.3, 128.3, 121.2, 86.1, 83.1, 80.9, 80.2, 66.4, 62.9, 28.2, 22.8, 14.0, 10.7; HRMS (ESI) calcd for $C_{20}H_{27}NO_5$ [M+Na]$^+$ 384.1781, found 384.1785; HPLC (Daicel Chiralcel IC-3, n-hexane/i-PrOH = 90/10, UV = 254 nm, flow rate = 1.0 mL/min) t_{R1} = 8.243 min (minor) and t_{R2} = 12.540 min (major), *ee* = 92%; $[\alpha]_D^{25}$ = +16.5 (*c* 0.7, CH_2Cl_2).

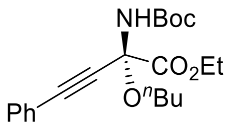

3u

Ethyl (*S*)-2-butoxy-2-((*tert*-butoxycarbonyl)amino)-4-phenylbut-3-ynoate (3u): Yellow oil (31.9 mg, 85% total yield, purified by flash column chromatography, petroleum ether/EtOAc = 10/1). ^{1}H NMR (400 MHz, Chloroform-*d*) δ 7.48 – 7.44 (m, 2H), 7.39 – 7.29 (m, 3H), 5.86 (s, 1H), 4.42 – 4.27 (m, 2H), 3.81 – 3.76 (m, 1H), 3.68 – 3.63 (m, 1H), 1.70 – 1.62 (m, 2H), 1.46 (s, 9H), 1.45 – 1.38 (m, 2H), 1.34 (t, *J* = 7.2 Hz, 3H), 0.92 (t, *J* = 7.2 Hz, 3H); ^{13}C NMR (101 MHz, $CDCl_3$) δ 166.5, 152.9, 132.0, 129.3, 128.4, 121.2, 86.1, 83.1, 80.9, 80.3, 64.4, 62.9, 31.6, 28.2, 19.3, 14.0, 13.8; HRMS (ESI) calcd for $C_{21}H_{29}NO_5$ [M+Na]$^+$ 398.1938, found 398.1937; HPLC (Daicel Chiralcel IC-3, n-hexane/i-PrOH = 90/10, UV = 254 nm, flow rate = 1.0 mL/min) t_{R1} = 7.370 min (minor) and t_{R2} = 11.475 min (major), *ee* = 91%; $[\alpha]_D^{25}$ = +18.0 (*c* 1.1, CH_2Cl_2).

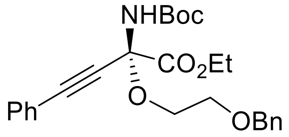

3v

Ethyl (*S*)-2-(2-(benzyloxy)ethoxy)-2-((*tert*-butoxycarbonyl)amino)-4-phenylbut-3-ynoate (3v): Pale yellow oil (39.5 mg, 87% total yield, purified by flash column chromatography,

petroleum ether/EtOAc = 10/1). ^{1}H NMR (400 MHz, Chloroform-*d*) δ 7.46 – 7.42 (m, 2H), 7.39 – 7.27 (m, 8H), 5.98 (s, 1H), 4.61 (s, 2H), 4.42 – 4.27 (m, 2H), 4.05 – 3.94 (m, 2H), 3.77 – 3.73 (m, 2H), 1.45 (s, 9H), 1.34 (t, *J* = 7.2 Hz, 3H); ^{13}C NMR (101 MHz, $CDCl_3$) δ 166.2, 152.9, 138.3, 132.0, 129.3, 128.3, 128.3, 127.8, 127.5, 121.0, 86.7, 82.6, 81.0, 80.5, 73.2, 68.9, 64.7, 62.9, 28.2, 14.0; HRMS (ESI) calcd for $C_{26}H_{31}NO_6$ $[M+Na]^+$ 476.2044, found 476.2044; HPLC (Daicel Chiralcel IC-3, n-hexane/i-PrOH = 90/10, UV = 254 nm, flow rate = 1.0 mL/min) t_{R1} = 19.600 min (minor) and t_{R2} = 36.955 min (major), *ee* = 86%; $[\alpha]_D^{25}$ = +10.8 (*c* 1.0, CH_2Cl_2).

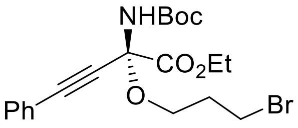

3w

Ethyl (*S*)-2-(3-bromopropoxy)-2-((tert-butoxycarbonyl)amino)-4-phenylbut-3-ynoate (3w): Yellow oil (40.5 mg, 92% total yield, purified by flash column chromatography, petroleum ether/EtOAc = 10/1). ^{1}H NMR (400 MHz, Chloroform-*d*) δ 7.49 – 7.44 (m, 2H), 7.40 – 7.30 (m, 3H), 5.91 (s, 1H), 4.44 – 4.25 (m, 2H), 3.98 – 3.90 (m, 1H), 3.88 – 3.79 (m, 1H), 3.57 (t, *J* = 6.4 Hz, 2H), 2.32 – 2.14 (m, 2H), 1.47 (s, 9H), 1.35 (t, *J* = 7.2 Hz, 3H); ^{13}C NMR (101 MHz, $CDCl_3$) δ 166.2, 152.9, 132.0, 129.4, 128.5, 128.4, 120.9, 86.5, 82.6, 81.1, 80.2, 63.0, 61.8, 32.6, 30.9, 28.2, 14.0; HRMS (ESI) calcd for $C_{20}H_{26}BrNO_5$ $[M+Na]^+$ 462.0887, found 462.0893; HPLC (Daicel Chiralcel IC-3, n-hexane/i-PrOH = 90/10, UV = 254 nm, flow rate = 1.0 mL/min) t_{R1} = 7.576 min (minor) and t_{R2} = 10.907 min (major), *ee* = 93%; $[\alpha]_D^{25}$ = +13.1 (*c* 1.0, CH_2Cl_2).

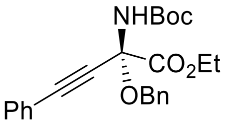

3x

Ethyl (*S*)-2-(benzyloxy)-2-((*tert*-butoxycarbonyl)amino)-4-phenylbut-3-ynoate (3x): Yellow oil (32.3 mg, 79% total yield, purified by flash column chromatography, petroleum ether/EtOAc = 10/1). ^{1}H NMR (400 MHz, Chloroform-*d*) δ 7.47 – 7.40 (m, 4H), 7.40 – 7.27 (m, 6H), 5.94 (s, 1H), 4.86 (dd, *J* = 32.4, 12.0 Hz, 2H), 4.44 – 4.26 (m, 2H), 1.45 (s, 9H), 1.35 (t, *J* = 7.2 Hz, 3H); ^{13}C NMR (101 MHz, $CDCl_3$) δ 166.3, 152.9, 137.8, 132.0, 129.4, 128.4, 128.3, 127.8, 127.5, 121.0, 86.8, 82.8, 81.1, 80.5, 67.1, 63.0, 28.2, 14.0; HRMS (ESI) calcd for $C_{24}H_{27}NO_5$ $[M+Na]^+$ 432.1781, found 432.1786; HPLC (Daicel Chiralcel IC-3, n-hexane/i-PrOH = 90/10, UV = 254 nm, flow rate = 1.0 mL/min) t_{R1} = 16.582 min (minor) and t_{R2} = 23.377 min (major), *ee* = 88%; $[\alpha]_D^{25}$ = +6.4 (*c* 0.8, CH_2Cl_2).

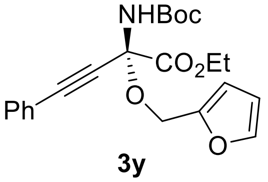

3y

Ethyl (*S*)-2-((*tert*-butoxycarbonyl)amino)-2-(furan-2-ylmethoxy)-4-phenylbut-3-ynoate (3y): Pale yellow oil (35.6 mg, 90% total yield, purified by flash column chromatography, petroleum ether/EtOAc = 10/1). ^{1}H NMR (400 MHz, Chloroform-*d*) δ 7.50 – 7.44 (m, 2H), 7.41 – 7.29 (m, 4H), 6.37 (d, *J* = 3.2 Hz, 1H), 6.32 (dd, *J* = 3.2, 2.0 Hz, 1H), 5.88 (s, 1H), 4.89 – 4.79 (m, 2H), 4.43 – 4.25 (m, 2H), 1.45 (s, 9H), 1.34 (d, *J* = 7.2 Hz, 3H); ^{13}C NMR (101 MHz, $CDCl_3$) δ 166.2, 152.8, 151.1, 142.8, 132.1, 129.4, 128.4, 121.0, 110.4, 109.8, 87.0, 82.5, 80.3, 63.0, 59.5, 28.2, 14.0; HRMS (ESI) calcd for $C_{22}H_{25}NO_6$ $[M+Na]^+$ 422.1574, found 422.1574; HPLC (Daicel Chiralcel IC-3, n-hexane/i-PrOH = 90/10, UV = 254 nm, flow rate = 1.0 mL/min) t_{R1} = 17.858 min (minor) and t_{R2} = 22.679 min (major), *ee* = 81%; $[\alpha]_D^{25}$ = -1.9 (*c* 1.0, CH_2Cl_2).

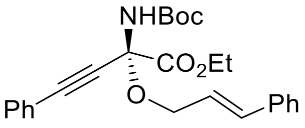

3z

Ethyl (*S*)-2-((*tert*-butoxycarbonyl)amino)-2-(cinnamyloxy)-4-phenylbut-3-ynoate (3z): Yellow solid (31.8 mg, 73% total yield, purified by flash column chromatography, petroleum ether/EtOAc = 10/1). Mp: 84-85 °C; ^{1}H NMR (400 MHz, Chloroform-*d*) δ 7.48 – 7.45 (m, 2H), 7.42 – 7.27 (m, 7H), 7.27 – 7.18 (m, 1H), 6.66 (d, *J* = 15.6 Hz, 1H), 6.38 (dt, *J* = 15.6, 6.0 Hz, 1H), 5.92 (s, 1H), 4.50 (ddd, *J* = 34.0, 12.4, 1.2 Hz, 2H), 4.42 – 4.29 (m, 2H), 1.45 (s, 9H), 1.36 (t, *J* = 7.2 Hz, 3H); ^{13}C NMR (101 MHz, $CDCl_3$) δ 166.3, 152.8, 136.7, 132.5, 132.0, 129.4, 128.5, 128.4, 127.6, 126.5, 125.7, 86.7, 82.7, 81.1, 80.3, 66.0, 63.0, 28.2, 14.0; HRMS (ESI) calcd for $C_{26}H_{29}NO_5$ $[M+Na]^+$ 458.1938, found 458.1942; HPLC (Daicel Chiralcel IC-3, n-hexane/i-PrOH = 90/10, UV = 254 nm, flow rate = 1.0 mL/min) t_{R1} = 14.264 min (minor) and t_{R2} = 18.762 min (major), *ee* = 93%; $[\alpha]_D^{25}$ = -10.9 (*c* 0.7, CH_2Cl_2).

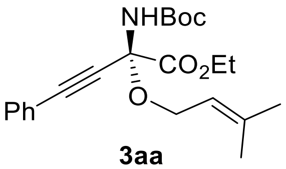

3aa

Ethyl (*S*)-2-((*tert*-butoxycarbonyl)amino)-2-((3-methylbut-2-en-1-yl)oxy)-4-phenylbut-3-ynoate (3aa): Pale yellow oil (36.4 mg, 94% total yield, purified by flash column chromatography, petroleum ether/EtOAc = 10/1). ^{1}H NMR (400 MHz, Chloroform-*d*) δ 7.49 – 7.43 (m, 2H), 7.40 – 7.29 (m, 3H), 5.88 (s, 1H), 5.48 – 5.41 (m, 1H), 4.44 – 4.21 (m, 4H), 1.74 (s, 3H), 1.69 (s, 3H), 1.46 (s, 9H), 1.35 (t, *J* = 7.2 Hz, 3H); ^{13}C NMR (101 MHz, $CDCl_3$) δ 166.5, 152.8, 137.4, 132.0, 129.3, 128.4, 121.2, 120.4, 86.3, 83.0, 80.9, 80.2, 62.9, 61.7, 28.2, 25.9, 18.1, 14.0; HRMS (ESI) calcd for $C_{22}H_{29}NO_5$ $[M+Na]^+$ 410.1938, found 410.1946; HPLC (Daicel Chiralcel IC-3, n-hexane/i-PrOH = 90/10, UV = 254 nm, flow rate = 1.0 mL/min) t_{R1} = 11.221 min (minor) and t_{R2} = 14.700 min (major), *ee* = 90%; $[\alpha]_D^{25}$ = +21.3 (*c* 1.2, CH_2Cl_2).

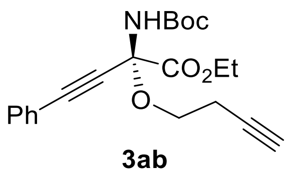

3ab

Ethyl (*S*)-2-(but-3-yn-1-yloxy)-2-((*tert*-butoxycarbonyl)amino)-4-phenylbut-3-ynoate (3ab): Pale yellow oil (35.2 mg, 95% total yield, purified by flash column chromatography, petroleum ether/EtOAc = 10/1). ^{1}H NMR (400 MHz, Chloroform-*d*) δ 7.49 – 7.42 (m, 2H), 7.41 – 7.29 (m, 3H), 5.89 (s, 1H), 4.45 – 4.26 (m, 2H), 4.01 – 3.90 (m, 1H), 3.90 – 3.81 (m, 1H), 2.69 – 2.52 (m, 2H), 1.97 (t, *J* = 2.8 Hz, 1H), 1.47 (s, 9H), 1.35 (t, *J* = 7.2 Hz, 3H); ^{13}C NMR (101 MHz, $CDCl_3$) δ 166.12, 152.84, 132.04, 129.44, 128.39, 120.89, 86.66, 82.42, 81.20, 80.74, 80.30, 69.53, 63.22, 63.00, 28.17, 19.85, 14.00; HRMS (ESI) calcd for $C_{21}H_{25}NO_5$ $[M+Na]^+$ 394.1625, found 394.1631; HPLC (Daicel Chiralcel IC-3, n-hexane/i-PrOH = 90/10, UV = 254 nm, flow rate = 0.5 mL/min) t_{R1} = 15.638 min (minor) and t_{R2} = 20.993 min (major), *ee* = 88%; $[\alpha]_D^{25}$ = +9.0 (*c* 0.4, CH_2Cl_2).

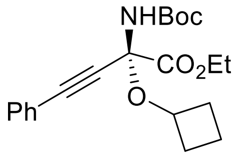

3ac

Ethyl (*S*)-2-((*tert*-butoxycarbonyl)amino)-2-cyclobutoxy-4-phenylbut-3-ynoate (3ac): Pale yellow oil (19.8 mg, 53% total yield, purified by flash column chromatography, petroleum ether/EtOAc = 10/1). ^{1}H NMR (400 MHz, Chloroform-*d*) δ 7.49 – 7.41 (m, 2H), 7.40 – 7.29 (m, 3H), 5.82 (s, 1H), 4.54 – 4.43 (m, 1H), 4.43 – 4.24 (m, 2H), 2.34 – 2.19 (m, 3H), 2.20 – 2.08 (m, 1H), 1.75 – 1.64 (m, 1H), 1.56 – 1.48 (m, 1H), 1.46 (s, 9H), 1.34 (t, *J* = 7.2 Hz, 3H); ^{13}C NMR (101 MHz, $CDCl_3$) δ 166.5, 152.7, 132.0, 129.3, 128.4, 121.2, 85.8, 81.0, 79.6, 69.1, 62.9, 32.2, 31.7, 28.2, 14.0, 13.5; HRMS (ESI) calcd for $C_{21}H_{27}NO_5$ $[M+Na]^+$ 396.1781, found 396.1778; HPLC (Daicel Chiralcel IC-3, n-hexane/i-PrOH = 90/10, UV = 254 nm, flow rate = 1.0 mL/min) t_{R1} = 9.196 min (minor) and t_{R2} = 16.159 min (major), *ee* = 91%; $[\alpha]_D^{25}$ = +4.6 (*c* 1.0, CH_2Cl_2).

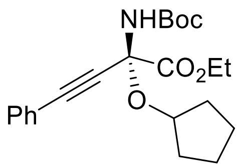

3ad

Ethyl (*S*)-2-((*tert*-butoxycarbonyl)amino)-2-(cyclopentyloxy)-4-phenylbut-3-ynoate (3ad): Pale yellow oil (19.4 mg, 50% total yield, purified by flash column chromatography, petroleum ether/EtOAc = 10/1). ^{1}H NMR (400 MHz, Chloroform-*d*) δ 7.48 – 7.42 (m, 2H), 7.38 – 7.29 (m, 3H), 5.88 (s, 1H), 4.53 – 4.46 (m, 1H), 4.40 – 4.25 (m, 2H), 1.93 – 1.79 (m, 3H), 1.79 – 1.66 (m, 3H), 1.54 – 1.42 (m, 2H), 1.46 (s, 9H), 1.34 (t, *J* = 7.2 Hz, 3H); ^{13}C NMR (101 MHz, $CDCl_3$) δ 166.9, 152.9, 132.0, 129.2, 128.3, 121.4, 85.8, 84.0, 80.8, 80.2, 77.8, 62.8, 33.5, 33.4, 28.2,

23.7, 23.6, 14.0; HRMS (ESI) calcd for $C_{22}H_{29}NO_5$ $[M+Na]^+$ 410.1938, found 410.1939; HPLC (Daicel Chiralcel IC-3, n-hexane/i-PrOH = 90/10, UV = 254 nm, flow rate = 1.0 mL/min) t_{R1} = 6.595 min (minor) and t_{R2} = 12.542 min (major), *ee* = 94%; $[\alpha]_D^{25}$ = +4.6 (*c* 0.4, CH_2Cl_2).

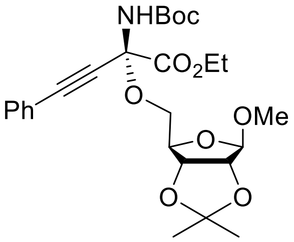

3ae

Ethyl (*S*)-2-((*tert*-butoxycarbonyl)amino)-2-(((3*aR*,4*R*,6*R*,6*aR*)-6-methoxy-2,2-dimethyltetrahydrofuro[3,4-*d*][1,3]dioxol-4-yl)methoxy)-4-phenylbut-3-ynoate (3ae): Colorless oil (44.0 mg, 87% total yield, purified by flash column chromatography, petroleum ether/EtOAc = 8/1). ^{1}H NMR (400 MHz, Chloroform-*d*) δ 7.51 – 7.42 (m, 2H), 7.42 – 7.31 (m, 3H), 5.91 (s, 1H), 4.96 (s, 1H), 4.81 (d, *J* = 6.0 Hz, 1H), 4.57 (d, *J* = 6.0 Hz, 1H), 4.45 (dd, *J* = 9.2, 5.2 Hz, 1H), 4.40 – 4.27 (m, 2H), 3.88 (dd, *J* = 9.6, 5.2 Hz, 1H), 3.74 (d, *J* = 9.6 Hz, 1H), 3.32 (s, 3H), 1.47 (s, 9H), 1.37 – 1.26 (m, 9H); ^{13}C NMR (101 MHz, $CDCl_3$) δ 166.1, 152.9, 132.0, 129.4, 128.4, 121.0, 112.2, 109.6, 87.0, 85.2, 84.7, 82.2, 80.4, 65.4, 63.0, 54.9, 31.4, 30.2, 28.2, 26.5, 25.0, 14.0; HRMS (ESI) calcd for $C_{26}H_{35}NO_9$ $[M+Na]^+$ 528.2204, found 528.2199; *dr* = 88:12; $[\alpha]_D^{25}$ = -9.1 (*c* 1.1, CH_2Cl_2).

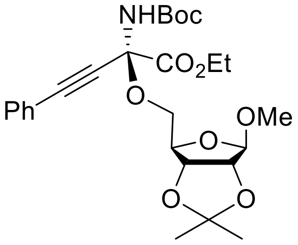

3ae'

Ethyl (*R*)-2-((tert-butoxycarbonyl)amino)-2-(((3*aR*,4*R*,6*R*,6*aR*)-6-methoxy-2,2-dimethyltetrahydrofuro[3,4-*d*][1,3]dioxol-4-yl)methoxy)-4-phenylbut-3-ynoate (3ae'): **Notice:** enantiomer of **L8** (***ent*-L8**) was used as ligand instead of **L8**. White solid (43.0 mg, 85% total yield, purified by flash column chromatography, petroleum ether/EtOAc = 8/1); Mp: 95-96 °C ^{1}H NMR (400 MHz, Chloroform-*d*) δ 7.49 – 7.44 (m, 2H), 7.40 – 7.31 (m, 3H), 5.90 (s, 1H), 4.96 (s, 1H), 4.85 (d, *J* = 6.0 Hz, 1H), 4.57 (d, *J* = 5.6 Hz, 1H), 4.43 (dd, *J* = 8.8, 6.0 Hz, 1H), 4.40 – 4.27 (m, 2H), 3.87 – 3.77 (m, 2H), 3.31 (s, 3H), 1.46 (s, 9H), 1.35 (t, *J* = 7.2 Hz, 3H); ^{13}C NMR (101 MHz, $CDCl_3$) δ 166.1, 152.9, 132.0, 129.4, 128.4, 120.9, 112.2, 109.5, 86.8, 85.1, 84.9, 82.5, 82.1, 81.2, 80.4, 65.6, 63.0, 54.9, 28.2, 26.5, 25.0, 14.0; HRMS (ESI) calcd for $C_{26}H_{35}NO_9$ $[M+Na]^+$ 528.2204, found 528.2210; *dr* = 94:6; $[\alpha]_D^{25}$ = -37.3 (*c* 0.9, CH_2Cl_2).

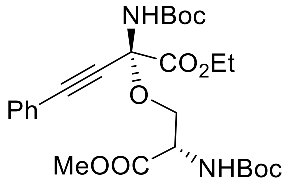

3af

Ethyl (*S*)-2-((*tert*-butoxycarbonyl)amino)-2-((*S*)-2-((*tert*-butoxycarbonyl)amino)-3-methoxy-3-oxopropoxy)-4-phenylbut-3-ynoate (3af): Colorless oil (43.2 mg, 83% total yield, purified by flash column chromatography, petroleum ether/EtOAc = 5/1). ^{1}H NMR (400 MHz, Chloroform-*d*) δ 7.49 – 7.44 (m, 2H), 7.41 – 7.30 (m, 3H), 5.94 (s, 1H), 5.63 (d, *J* = 8.4 Hz, 1H), 4.55 – 4.45 (m, 1H), 4.40 – 4.28 (m, 2H), 4.20 (dd, *J* = 9.2, 2.8 Hz, 1H), 4.07 (dd, *J* = 9.2, 2.8 Hz, 1H), 3.74 (s, 3H), 1.47 (s, 9H), 1.44 (s, 9H), 1.35 (t, *J* = 6.8 Hz, 3H), 1.34 (s, 3H); ^{13}C NMR (101 MHz, $CDCl_3$) δ 170.7, 165.9, 155.7, 152.9, 132.1, 129.5, 128.4, 120.8, 87.3, 81.9, 80.2, 79.8, 65.3, 63.1, 53.8, 52.5, 31.4, 30.2, 28.3, 28.2, 14.0; HRMS (ESI) calcd for $C_{26}H_{36}N_2O_9$ $[M+Na]^+$ 543.2313, found 543.2312; *dr* = 85:15; $[\alpha]_D^{25}$ = -2.9 (*c* 1.1, CH_2Cl_2).

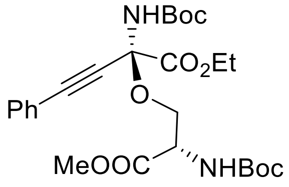

3af'

Ethyl (*R*)-2-((tert-butoxycarbonyl)amino)-2-((*S*)-2-((tert-butoxycarbonyl)amino)-3-methoxy-3-oxopropoxy)-4-phenylbut-3-ynoate (3af'): Notice: enantiomer of **L8** (***ent*-L8**) was used as ligand instead of **L8**. Colorless oil (43.3 mg, 83% total yield, purified by flash column chromatography, petroleum ether/EtOAc = 5/1). ^{1}H NMR (400 MHz, Chloroform-*d*) δ 7.50 – 7.43 (m, 2H), 7.41 – 7.29 (m, 3H), 5.93 (s, 1H), 5.76 (s, 1H), 4.49 (dt, *J* = 8.8, 2.8 Hz, 1H), 4.39 – 4.30 (m, 2H), 4.28 (dd, *J* = 6.8, 3.2 Hz, 1H), 4.01 (dd, *J* = 9.6, 3.2 Hz, 1H), 3.75 (s, 3H), 1.48 (s, 9H), 1.43 (s, 9H), 1.34 (t, *J* = 7.2 Hz, 3H); ^{13}C NMR (101 MHz, $CDCl_3$) δ 170.8, 165.9, 155.9, 153.1, 132.1, 129.5, 128.4, 120.7, 87.4, 81.7, 81.4, 80.3, 79.8, 65.4, 63.1, 53.7, 52.5, 28.3, 28.2, 14.0; HRMS (ESI) calcd for $C_{26}H_{36}N_2O_9$ $[M+Na]^+$ 543.2313, found 543.2316; *dr* = 88:12; $[\alpha]_D^{25}$ = -5.1 (*c* 0.9, CH_2Cl_2).

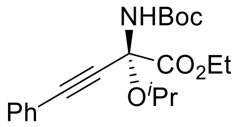

3ag

Ethyl (*S*)-2-((*tert*-butoxycarbonyl)amino)-2-isopropoxy-4-phenylbut-3-ynoate (3ag): trace yield, HRMS (ESI) calcd for $C_{20}H_{27}NO_5$ $[M+Na]^+$ 384.1781, found 384.1782.

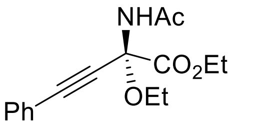

3ah

Ethyl (*S*)-2-acetamido-2-ethoxy-4-phenylbut-3-ynoate (3ah): white solid (9.9 mg, 34% total yield, purified by flash column chromatography, petroleum ether/EtOAc = 5/1). ^{1}H NMR (400 MHz, Chloroform-*d*) δ 7.49 – 7.45 (m, 2H), 7.39 – 7.30 (m, 3H), 6.68 (s, 1H), 4.36 (q, *J* = 7.2 Hz, 2H), 3.93 – 3.82 (m, 1H), 3.81 – 3.72 (m, 1H), 2.07 (s, 3H), 1.35 (t, *J* = 7.2 Hz, 3H), 1.31 (t, *J* = 7.2 Hz, 3H); ^{13}C NMR (101 MHz, $CDCl_3$) δ 168.8, 166.4, 132.1, 129.4, 128.4, 121.0, 86.5, 82.8, 79.7, 63.1, 61.1, 23.3, 15.2, 14.0; HRMS (ESI) calcd for $C_{16}H_{19}NO_4$ $[M+Na]^+$ 312.1206, found 312.1205.

Supplementary Note 4

Gram-scale synthesis of 3a

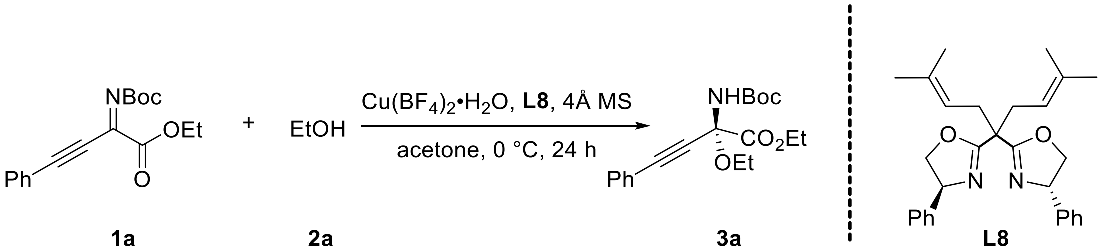

1a (1.0 g, 3.3 mmol) **2a** (0.96 mL, 16.5mmol) **3a** 95% yield, 96% ee **L8**

A flame-dried Schlenk tube equipped with a magnetic stirring bar, was charged with a mixture of $Cu(BF_4)_2 \bullet H_2O$ (10 mol%), **L2** (15 mol%) and 4Å MS (1.3 g). After being evacuated and refilled with nitrogen for three times, acetone (5 mL) was added to the Schlenk tube and the mixture was stirred at room temperature under a N_2 atmosphere for 1 h. Imines **1** (1.0 g, 3.3 mmol) and alcohol **2** (0.96 mL, 16.5 mmol) was added sequentially. The reaction mixture was allowed to stir under a N_2 atmosphere at 0 °C for 24 h. When the reaction was completed, solvent was evaporated in vacuo and the residue was purified by flash silica gel column chromatography (PE/EtOAc = 10/1) to give product **3a** as a pale yellow solid (1.1 g, 95% yield, 96% *ee*).

Procedure for synthesis of 4 and characterization data

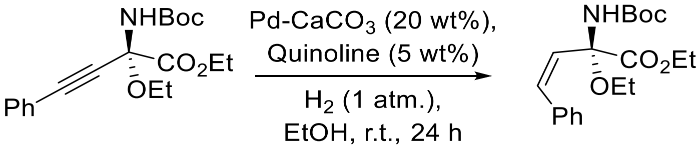

3a **4**

(0.2 mmol, 96% *ee*) (73% yield, 96% *ee*)

A flame dried 5 mL vial, equipped with a stir bar, was charged with **3a** (69.5 mg, 0.2 mmol) and dry ethanol (2.0 mL). Lindlar Catalyst (20 wt% $Pd\text{-}CaCO_3$, 14.0 mg) was added to the solution and the vial was equipped with a H_2 balloon. The reaction mixture was stirred under room temperature for 24 h. When the reaction was completed, the reaction mixture was filtered

through a plug of Celite and concentrated in vacuo. The crude product was purified by silica gel column chromatography (petroleum ether/EtOAc = 20/1) to give pure product **4** as a colorless oil to give the title product (51.1 mg, 73% yield) as a colorless oil without any further purification. ^{1}H NMR (400 MHz, Chloroform-*d*) δ 7.31 – 7.20 (m, 5H), 6.65 (d, *J* = 12.4 Hz, 1H), 6.10 (s, 1H), 6.02 (d, *J* = 12.4 Hz, 1H), 4.06 – 3.98 (m, 1H), 3.92 – 3.84 (m, 1H), 3.51 – 3.43 (m, 1H), 3.24 – 3.17 (m, 1H), 1.40 (s, 9H), 1.15 (t, *J* = 7.2 Hz, 3H), 1.12 (t, *J* = 7.2 Hz, 3H); ^{13}C NMR (101 MHz, $CDCl_3$) δ 169.3, 152.8, 135.7, 131.8, 130.3, 129.0, 127.8, 127.4, 84.9, 80.1, 62.4, 58.5, 28.2, 15.1, 13.8; HRMS (ESI) calcd for $C_{19}H_{27}NO_5$ $[M+Na]^+$ 372.1781, found 372.1789 HPLC (Daicel Chiralcel IC-3, n-hexane/i-PrOH = 90/10, UV = 220 nm, flow rate = 1.0 mL/min) t_{R1} = 6.022 min (minor) and t_{R2} = 6.515 min (major), *ee* = 96%; $[\alpha]_D^{25}$ = -59.7 (*c* 0.7, CH_2Cl_2).

Procedure for synthesis of 5 and characterization data

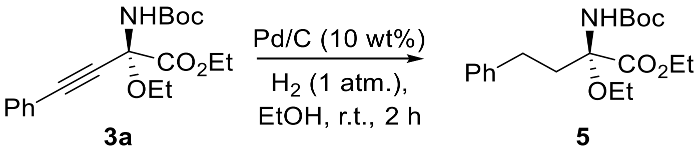

3a (0.2 mmol, 96% *ee*) **5** > 99% yield, 95% *ee*

A flame dried 5 mL vial, equipped with a stir bar, was charged with **3a** (69.5 mg, 0.2 mmol) and dry ethanol (2.0 mL). Pd/C (10 wt% Pd, 7.0 mg) was added to the solution and the vial was equipped with a H_2 balloon. The reaction mixture was stirred under room temperature for 2 h. When the reaction was completed, the reaction mixture was filtered through a plug of Celite and concentrated in vacuo and the residue was purified by flash silica gel column chromatography (PE/EtOAc = 10/1) to give product **4** (70.1 mg, > 99% yield) as a colorless oil. 1H NMR (400 MHz, Chloroform-d) δ 7.28 – 7.24 (m, 2H), 7.19 – 7.14 (m, 3H), 5.85 (s, 1H), 4.25 – 4.13 (m, 2H), 3.58 – 3.50 (m, 1H), 3.38 – 3.30 (m, 1H), 2.80 (s, 1H), 2.64 – 2.57 (m, 1H), 2.49 – 2.42 (m, 1H), 2.29 – 2.22 (m, 1H), 1.46 (s, 9H), 1.30 (t, *J* = 7.2 Hz, 3H), 1.21 (t, *J* = 7.2 Hz, 3H);^{13}C NMR (101 MHz, $CDCl_3$) δ 170.4, 153.3, 140.8, 128.4, 128.3, 126.0, 87.4, 80.0, 62.2, 59.2, 37.0, 30.1, 28.3, 15.3, 14.2; HRMS (ESI) calcd for $C_{19}H_{29}NO_5$ $[M+Na]^+$ 374.1938, found 374.1944 HPLC (Daicel Chiralcel IC-3, n-hexane/i-PrOH = 90/10, UV = 220 nm, flow rate = 1.0 mL/min) t_{R1} = 5.327 min (minor) and t_{R2} = 6.120 min (major), *ee* = 95%; $[\alpha]_D^{25}$ = -3.2 (*c* 2.3 CH_2Cl_2).

Procedure for synthesis of 6 and characterization data

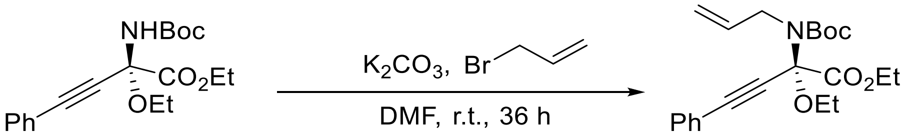

3a (0.2 mmol, 96% *ee*) **6** (71% yield, 91% *ee*)

A flame dried 5 mL vial, equipped with a stir bar, was charged with **3a** (69.5 mg, 0.2 mmol) and dry DMF (2.0 mL). 3-bromoprop-1-ene (0.17 mL, 2.0 mmol) was added to the solution followed by K_2CO_3 (138.0 mg, 1.0 mmol). The reaction mixture was stirred under N_2 atmosphere at room temperature. Another portion of K_2CO_3 (138.0 mg, 1.0 mmol) was added after 24 h.

The reaction was allowed to stir at room temperature for another 12 h. When the reaction was completed, the reaction mixture was quenched by 1M HCl and extracted with EtOAc (3×2mL), the combined organic layer was washed with brine (3×2mL), dried over Na_2SO_4 and concentrated in vacuo. The residue was purified by silica gel column chromatography (petroleum ether/EtOAc = 20/1) to give pure product **6** as a colorless oil to give the title product (55.1 mg, 71% yield) as a colorless oil. ^{1}H NMR (400 MHz, Chloroform-*d*) δ 7.47 – 7.43 (m, 2H), 7.40 – 7.30 (m, 3H), 6.03 – 5.93 (m, 1H), 5.34 – 5.29 (m, 1H), 5.17 – 5.13 (m, 1H), 4.34 – 4.23 (m, 4H), 3.89 – 3.82 (m, 1H), 3.75 – 3.68 (m, 1H), 1.45 (s, 9H), 1.32 (t, *J* = 7.2 Hz, 3H), 1.30 (t, *J* = 7.2 Hz, 3H); ^{13}C NMR (101 MHz, $CDCl_3$) δ 166.7, 153.9, 135.0, 131.9, 129.3, 128.4, 121.3, 116.0, 89.0, 85.7, 82.5, 81.4, 62.3, 60.8, 49.3, 28.2, 15.3, 14.0; HRMS (ESI) calcd for $C_{22}H_{29}NO_5$ $[M+Na]^+$ 410.1938, found 410.1935; HPLC (Daicel Chiralcel OD-H, n-hexane/i-PrOH = 97/3, UV = 254 nm, flow rate = 0.3 mL/min) t_{R1} = 14.055 min (minor) and t_{R2} = 15.046 min (major), *ee* = 91%; $[\alpha]_D^{25}$ = +47.4 (*c* 0.8, CH_2Cl_2).

Procedure for synthesis of 7 and characterization data

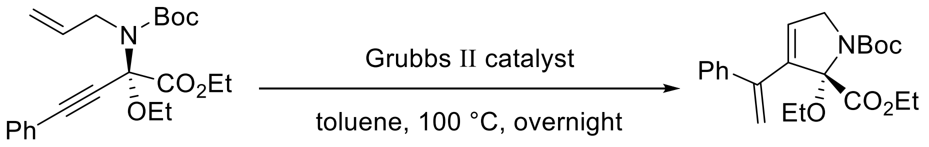

6 (0.1 mmol, 91% *ee*)

7 (72% yield, 88% *ee*)

Product **7** was synthesized based on a reported procedure.[4] A flame dried 10 mL Schlenk tube, equipped with a stir bar, was charged with **6** (38.7 mg, 0.1 mmol) and Grubbs II catalyst (4.2 mg, 0.005 mmol). Dry toluene (1.0 mL) was added through a syringe. The reaction mixture was allowed to stirr under N_2 atmosphere at 100 °C overnight. When the reaction was completed, the mixture was concentrated in vacuo. The residue was purified by silica gel column chromatography (petroleum ether/EtOAc/DCM = 10/1/1) to give pure product **7** (28 mg, 72% yield) as a colorless oil. ^{1}H NMR (500 MHz, DMSO-*d*$_6$) δ 7.41 – 7.31 (m, 3H), 7.25 – 7.20 (m, 2H), 5.98 – 5.89 (m, 1H), 5.74 (s, 1H), 5.17 (s, 1H), 4.22 – 4.02 (m, 4H), 3.32 – 3.25 (m, 2H), 1.40 (d, *J* = 26.0 Hz, 9H), 1.16 (d, *J* = 7.0 Hz, 3H), 1.12 (d, *J* = 7.5 Hz, 3H); ^{13}C NMR (126 MHz, DMSO) δ 153.7, 153.4, 138.0, (137.9), 127.0, (126.9), 126.7, 123.04, (122.8), (117.0), 116.8, 114.3, 113.9, (113.7), (102.6), 102.5, (82.5), 82.1, 65.9, 65.8, 47.0, (46.7), (43.2), 43.1, (38.7), 38.3, (13.9), 13.8, 1.2, (1.2); There was another rotamer existing with Product **7** when it was did NMR analysis according to a literature report.[5] Singals for the minor rotamer are given in paretheses. HRMS (ESI) calcd for $C_{22}H_{29}NO_5$ $[M+Na]^+$ 410.1938, found 410.1938; HPLC (Daicel Chiralcel OX, n-hexane/i-PrOH = 97/3, UV = 254 nm, flow rate = 0.3 mL/min) t_{R1} = 29.357 min (minor) and t_{R2} = 35.492 min (major), *ee* = 88%; $[\alpha]_D^{25}$ = +25.6 (*c* 0.5, CH_2Cl_2).

Procedure for synthesis of 8 and characterization data

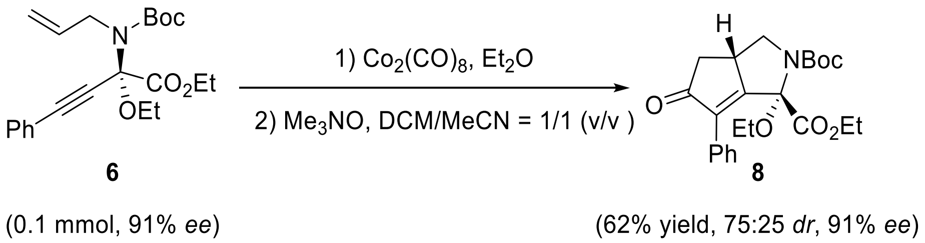

Product **8** was synthesized based on a reported procedure.[6] A flame dried 10 mL Schlenk tube, equipped with a stir bar, $Co_2(CO)_8$ was added and dissolved in Et_2O (1.0 mL) followed by the addition of **6** (38.7 mg, 0.1 mmol) dissolved in Et_2O (0.5 mL) through a syringe under the protection of N_2 flow. The mixture was stirred at room temperature for 1 h. Afterward, the solvent was evaporated in vacuo. The residue was dissolved in DCM (1.0 mL) and was injected to Me_3NO which was suspended in MeCN (1.0 mL) in a Schlenk tube. The reaction mixture was allowed to stirr under N_2 atmosphere at room temperature for 24 h. When the reaction was completed, the mixture was concentrated in vacuo. The residue was purified by silica gel column chromatography (petroleum ether/EtOAc = 10/1) to give pure product **8** (25.7 mg, 62% yield, 75:25 *dr*, 91% *ee*) as a white solid; Mp: 113 - 114 °C. ^1H NMR (400 MHz, Chloroform-*d*) δ 7.55 – 7.47 (m, 2H), 7.44 – 7.31 (m, 3H), 4.45 – 4.29 (m, 2H), 4.33 – 4.20 (m, 1H), 3.61 – 3.49 (m, 1H), 3.23 – 3.11 (m, 1H), 3.07 (t, *J* = 10.0 Hz, 1H), 2.88 (dd, *J* = 18.0, 6.4 Hz, 1H), 2.86 – 2.73 (m, 1H), 2.45 (dd, *J* = 18.0, 3.2 Hz, 1H), 1.44 (s, 9H), 1.38 (t, *J* = 7.2 Hz, 3H), 0.66 (t, *J* = 7.2 Hz, 3H); ^{13}C NMR (101 MHz, $CDCl_3$) δ 206.1, 169.9, 167.5, 152.84, 140.1, 129.9, 129.2, 128.4, 128.1, 91.0, 81.6, 62.6, 58.8, 51.7, 40.9, 39.7, 28.2, 14.4, 14.3; HRMS (ESI) calcd for $C_{23}H_{29}NO_6$ $[M+Na]^+$ 438.1887, found 438.1886; HPLC (Daicel Chiralcel OD-H, n-hexane/i-PrOH = 95/5, UV = 254 nm, flow rate = 0.5 mL/min) t_{R1} = 17.896 min (major), t_{R2} = 21.506 min (minor), t_{R3} = 25.511 min (minor), t_{R4} = 26.883 min (major); *ee* = 91%; $[\alpha]_D^{25}$ = -144.7 (*c* 0.2, CH_2Cl_2).

Procedure for synthesis of 9 and characterization data

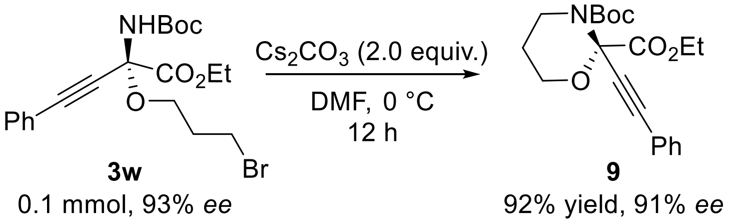

A flame dried 10 mL Schlenk tube, equipped with a stir bar, **3w** (39.6 mg, 0.1 mmol) was added and dissolved in DMF (1.0 mL) followed by the addition of Cs_2CO_3 (65.2 mg, 0.2 mmol) under the protection of N_2 flow. The reaction mixture was stirred at 0 °C for 12 h. When the reaction was completed, the mixture was quenched with 1M aq. HCl, extracted with EtOAc (2 mL×3). The combined organic layer was washed by water (5 mL×3), dried over Na_2SO_4 and concentrated in vacuo. The residue was purified by silica gel column chromatography (petroleum ether/EtOAc = 10/1) to give pure product **9** (33.0 mg, 92% yield) as a yellow solid; Mp: 77 – 78 °C. ^1H NMR (400 MHz, Chloroform-*d*) δ 7.54 – 7.47 (m, 2H), 7.39 – 7.29 (m, 3H), 4.43 – 4.25 (m, 2H), 4.25 – 4.16 (m, 1H), 4.03 (dd, *J* = 10.8, 4.8 Hz,

1H), 3.95 (dt, J = 12.8, 4.0 Hz, 1H), 3.59 – 3.39 (m, 1H), 2.12 – 1.97 (m, 1H), 1.66 – 1.59 (m, 1H), 1.46 (s, 9H), 1.35 (t, J = 7.2 Hz, 3H); ^{13}C NMR (101 MHz, $CDCl_3$) δ 165.8, 154.3, 132.0, 129.0, 128.3, 121.7, 100.0, 83.7, 82.6, 82.0, 62.5, 28.2, 28.0, 23.8, 14.0; HRMS (ESI) calcd for $C_{20}H_{25}NO_5$ $[M+Na]^+$ 382.1625, found 382.1627; HPLC (Daicel Chiralcel IC-3, n-hexane/i-PrOH = 90/10, UV = 254 nm, flow rate = 1.0 mL/min) t_{R1} = 22.912 min (major) and t_{R2} = 30.292 min (minor), *ee* = 91%; $[\alpha]_D^{25}$ = +87.4 (*c* 1.1, CH_2Cl_2).

Procedure for synthesis of 10 and characterization data

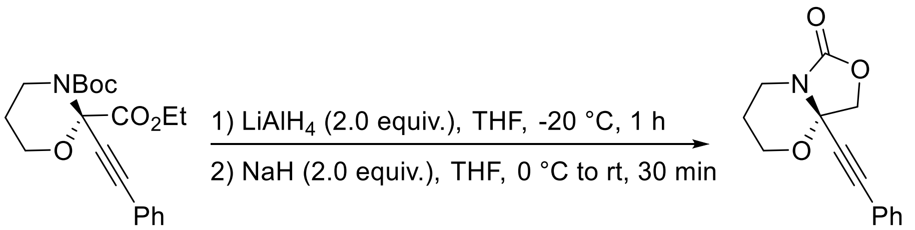

9
0.1 mmol, 91% *ee*

10
54% yield, 88% *ee*

Step 1: A flame dried 10 mL Schlenk tube equipped with a stir bar was charged with **9** (35.9 mg, 0.1 mmol). After being evacuated and backfilled with nitrogen for three times, THF (1.0 mL) was added to the Schlenk tube. The mixture was allowed to be stirred at -20 °C. Then $LiAlH_4$ (1.0 M in THF, 0.2 mL, 0.2 mmol) was added dropwise. The reaction mixture was stirred at -20 °C for 1 h. When the reaction was completed, the mixture was quenched with saturated aq. NH_4Cl (0.1 mL). Then Na_2SO_4 was added directly and the mixture was filtered, washed with DCM and concentrated in vacuo to give the crude product which was used directly in the next step without any further purification.

Step 2: A flame dried 10 mL Schlenk tube equipped with a stir bar was charged with the crude product of first step. After being evacuated and backfilled with nitrogen for three times, THF (1.0 mL) was added to the Schlenk tube. The mixture was allowed to be stirred at 0 °C and NaH (0.2 mmol) was added in one portion under a nitrogen atmosphere. Then the reaction was stirred at rt for 30 min and quenched with saturated aq. NH_4Cl (0.1 mL). Then Na_2SO_4 was added and the mixture was filtered, washed with DCM and concentrated in vacuo. The residue was purified by silica gel column chromatography (petroleum ether/EtOAc = 5/1) to give pure product **10** (16.3 mg, 67% yield) as a colorless oil; ^{1}H NMR (500 MHz, Chloroform-*d*) δ 7.55 – 7.47 (m, 2H), 7.44 – 7.33 (m, 3H), 4.48 (d, J = 9.5 Hz, 1H), 4.39 (d, J = 9.5 Hz, 1H), 4.33 (td, J = 12.5, 2.0 Hz, 1H), 4.02 (dd, J = 12.0, 5.0 Hz, 1H), 3.92 (dd, J = 13.5, 5.0 Hz, 1H), 3.52 (dd, J = 13.5, 3.5 Hz, 1H), 2.06 – 1.94 (m, 1H), 1.54 – 1.46 (m, 1H); ^{13}C NMR (126 MHz, $CDCl_3$) δ 156.2, 132.0, 129.6, 128.5, 120.8, 88.1, 83.9, 81.7, 73.6, 63.4, 38.3, 24.4; HRMS (ESI) calcd for $C_{14}H_{13}NO_3$ $[M+Na]^+$ 266.0788, found 266.0785; HPLC (Daicel Chiralcel OD-H, n-hexane/i-PrOH = 96/4, UV = 254 nm, flow rate = 0.4 mL/min) t_{R1} = 38.982 min (minor) and t_{R2} = 40.643 min (major), *ee* = 88%; $[\alpha]_D^{25}$ = -9.9 (*c* 0.4, CH_2Cl_2).

Procedure for synthesis of 11 and characterization data

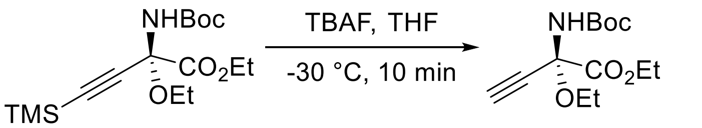

3o
0.2 mmol, 95% *ee*

11
quant. yield, 93% *ee*

A flame dried 10 mL Schlenk tube, equipped with a stir bar, **3o** (68.7 mg, 0.2 mmol) was added and dissolved in THF (2.0 mL). The mixture was stirred at -30 °C, TBAF (1M in THF, 0.24 mL, 0.24 mmol) was added slowly through a syringe under N_2 atmophere. The reaction mixture was stirred at the same temperature for 10 min then quenched by saturated NH_4Cl (aq.). The mixture was extracted with EtOAc (3×2 mL). The combined organic layer was dried over Na_2SO_4, filtered and concentrated in vacuo.The residue was purified by silica gel column chromatography (petroleum ether/EtOAc = 5/1) to give pure product **11** (54.2 mg, quant. yield) as a colorless oil. ^{1}H NMR (400 MHz, Chloroform-*d*) δ 5.81 (s, 1H), 4.43 – 4.25 (m, 2H), 3.82 – 3.73 (m, 1H), 3.71 – 3.62 (m, 1H), 2.64 (s, 1H), 1.45 (s, 9H), 1.34 (t, *J* = 7.2 Hz, 3H), 1.28 (t, *J* = 7.2 Hz, 4H); ^{13}C NMR (101 MHz, $CDCl_3$) δ 165.2, 151.8, 80.0, 78.5, 76.7, 73.6, 62.0, 59.6, 27.1, 14.1, 12.9; HRMS (ESI) calcd for $C_{13}H_{21}NO_5$ $[M+Na]^+$ 294.1312, found 294.1318; HPLC (Daicel Chiralcel AD-H, n-hexane/i-PrOH = 95/5, UV = 220 nm, flow rate = 1.0 mL/min) t_{R1} = 12.319 min (major) and t_{R2} = 14.830 min (minor), *ee* = 93%; $[\alpha]_D^{25}$ = -0.7 (*c* 1.1, CH_2Cl_2).

Procedure for synthesis of 12 and characterization data

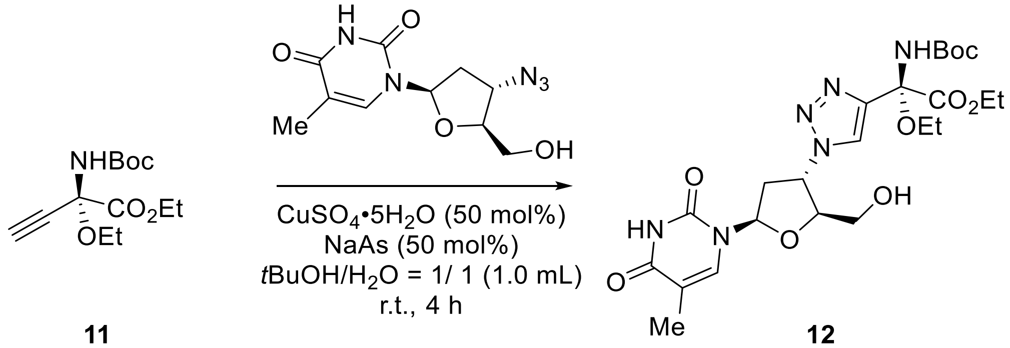

11
0.1 mmol, 93% *ee*

12
70% yield, 89:11 *dr*

Product **12** was synthesized based on a reported procedure.[7] A flame dried 10 mL Schlenk tube was charged with **11** (27.1 mg, 0.1 mmol), zidovudine (31.1 mg, 0.11 mmol) and ^{t}BuOH (0.5 mL). Subsequently, a freshly prepared solution of sodium ascorbate (9.9 mg, 0.05 mmol) and $CuSO_4{\cdot}5H_2O$ (12.5 mg, 0.05 mmol) in H_2O (0.5 mL) was added. The reaction mixture was stirred at room temperature for 4 h and then was extracted with EtOAc (3×5 mL). The combined organic layer was dried over Na_2SO_4 and concentrated in vacuo. The residue was purified by column chromatography (DCM/MeOH = 20/1) to afford the title product **12** (37.6 mg, 70% yield, 89:11 *dr*) as a white solid; Mp: 105 – 106 °C. ^{1}H NMR (500 MHz, DMSO-d_6) δ 11.36 (s, 1H), 8.42 (s, 1H), 7.99 (s, 1H), 7.81 (s, 1H), 6.41 (t, *J* = 6.5 Hz, 1H), 5.40 – 5.34 (m, 1H), 5.34 – 5.26 (m, 1H), 4.21 – 4.17 (m, 1H), 4.16 – 4.08 (m, 1H), 4.06 – 3.97 (m, 1H), 3.70 (dd, *J* = 12.0, 3.0 Hz, 1H), 3.62 (dd, *J* = 12.0, 3.5 Hz, 1H), 3.49 – 3.42 (m, 1H), 3.33 – 3.29 (m, 1H), 2.77 – 2.62 (m, 2H), 1.81 (d, *J* = 1.0 Hz, 3H), 1.40 (s, 9H), 1.11 (t, *J* = 7.0 Hz, 3H), 1.09 (t, *J* = 7.0 Hz, 3H); ^{13}C NMR (126 MHz, DMSO) δ 167.9, 164.2, 154.0, 150.9, 145.3, 136.7, 124.1, 110.1, 84.9,

84.3, 83.4, 79.7, 61.6, 61.2, 59.9, 58.6, 37.4, 28.4, 15.8, 14.3, 12.7; HRMS (ESI) calcd for $C_{23}H_{34}N_6O_9$ [M+Na]+ 561.2279, found 561.2273; *dr* = 89:11; $[\alpha]_D^{25}$ = -9.5 (*c* 0.2, CH_2Cl_2).

Supplementary Note 5

X-Ray Crystal Structure Analysis

X-Ray crystallography data for **3a** (CCDC 2063358): A pale yellow crystal suitable for X-ray crystallography was obtained from a n-hexane/dichloromethane solution at room temperature under air.

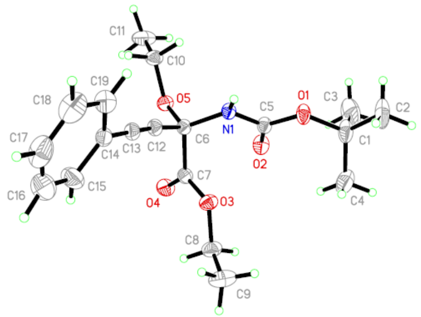

Supplementary Fig. 2. The ORTEP drawing (50% probability for thermal ellipsoids) of **3a**

Bond precision:	C-C = 0.0061 A	Wavelength=1.54178	
Cell:	a=10.0921(2)	b=10.2495(2)	c=19.8588(4)
	alpha=90	beta=90	gamma=90
Temperature:	296 K		

	Calculated	Reported
Volume	2054.17(7)	2054.17(7)
Space group	P 21 21 21	P 21 21 21
Hall group	P 2ac 2ab	P 2ac 2ab
Moiety formula	C19 H25 N O5	C19 H25 N O5
Sum formula	C19 H25 N O5	C19 H25 N O5
Mr	347.40	347.40
Dx,g cm-3	1.123	1.123
Z	4	4
Mu (mm-1)	0.665	0.665
F000	744.0	744.0
F000'	746.39	
h,k,lmax	12,12,23	12,12,23
Nref	3781[2169]	3756
Tmin,Tmax	0.887,0.911	
Tmin'	0.887	

Correction method= Not given

Data completeness= 1.73/0.99 Theta(max)= 68.388

R(reflections)= 0.0413(3466) wR2(reflections)= 0.1290(3756)

S = 1.056 Npar= 235

X-Ray crystallography data for **8** (CCDC 2088332): A colorless crystal suitable for X-ray crystallography was obtained from a n-hexane/dichloromethane solution at room temperature under air.

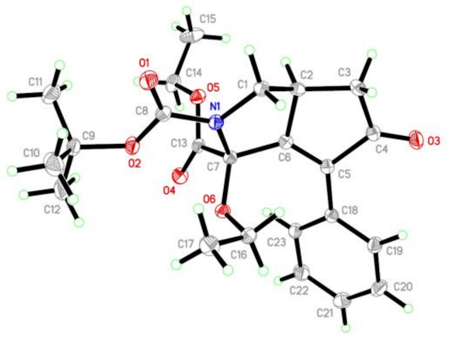

Supplementary Fig. 3. The ORTEP drawing (50% probability for thermal ellipsoids) of **8**

```
Bond precision:   C-C = 0.0029 A          Wavelength=1.54178

Cell:             a=11.520(3)     b=7.9844(13)          c=12.592(3)
                  alpha=90        beta=109.912(14)      gamma=90
Temperature:      173 K

                 Calculated                  Reported
Volume           1089.0(4)                   1088.9(5)
Space group      P 21                        P 21
Hall group       P 2yb                       P 2yb
Moiety formula   C23 H29 N O6                C23 H29 N O6
Sum formula      C23 H29 N O6                C23 H29 N O6
Mr               415.47                      415.47
Dx,g cm-3        1.267                       1.267
Z                2                           2
Mu (mm-1)        0.750                       0.750
F000             444.0                       444.0
F000'            445.43
h,k,lmax         13,9,15                     13,9,15
Nref             3997[ 2153]                 3945
Tmin,Tmax        0.887,0.914                 0.635,0.753
Tmin'            0.887

Correction method= # Reported T Limits: Tmin=0.635 Tmax=0.753
AbsCorr = MULTI-SCAN

Data completeness= 1.83/0.99      Theta(max)= 68.283

R(reflections)= 0.0272( 3762)      wR2(reflections)= 0.0716( 3945)

S = 1.006                  Npar= 276
```

Supplementary Note 6

Computational methods

Computations were performed using the Gaussian 09 (revision D.01) suite of quantum chemical program. All structures are optimized in an implicit solvent model using thePBE0[8] hybrid functional with usage of empirical Grimme's dispersion correction[9] (GD3-BJ). In optimization, all atoms except palladium are described with Dunning's correlation-consistent basis sets, cc-pVDZ[10] (a double-ζ basis set). All structures are in a local minimum potential energy surface with zero imaginary frequency or at the first order saddle point (transition state) on the potential surface with one imaginary frequency. Transition states (TSs) are calculated by the Berny algorithm.[11] Intrinsic reaction coordinate (IRC) calculations are additionally carried out to further characterize the true nature of the TSs.[12] Harmonic vibrational frequencies, thermal, and entropic corrections at 273.15K and $p^{\ominus}$ were obtained from frequency calculations. A global multiplicative harmonic frequency scaling factor for PBE0/cc-pVDZ basis set, 0.9560[13], were used as a correction for calculated harmonic frequencies and thermal data. The contribution of low frequency vibration is modified by quas*i*-harmonic approximation proposed by Grimme[14] considering the contribution of low frequency vibration (<100.0 cm^{-1}) to the

partition function, using the free-rotor approximation. For those above this threshold, the RRHO approximation is retained. All frequency and thermal corrections are computed using Shermo.[15] Single point energy in the gas phase is computed using the PBE0-D3BJ functional while the cc-pvNz complete basis set (cc-pvDz/Tz)[16] is used for all atoms. The effect of a solvent continuum, in acetone, was evaluated using the Cramer−Truhlar continuum solvation model that describes the electrostatic interaction and nonpolar interaction between solvent and solute, named as SMD.[17] Given the fitting method of the SMD model, the difference in electron energy at the SMD/M052X/6-31G* level of theory and M052X/6-31G* level of theory was calculated as the free energy of solvation.

Electron energies and Gibbs free energies.

Solvation free energies

Given the fitting methods of SMD implicit solvent model, calculating solvation free energies at the same level of theory (M052X/6-31G*) should be the best way to acquire solvation free energies.

Supplementary Table 4. Solvation free energies at M052X/6-31G* level of theory with or without SMD_{THF} implicit solvent model (unit are in a.u.).

	$E_{M052X/6-31G*}$	$E_{M052X/6-31G*, Acetone}$	G_{solve}
**I**	-4039.896853	-4040.114588	-0.21773506
**TS-1**	-4388.051051	-4388.269231	-0.21818005
**TS-2**	-4388.053492	-4388.271758	-0.218266
**TS-3**	-4194.900437	-4195.123518	-0.2230805
**TS-4**	-4194.902792	-4195.12419	-0.221398
**Acetone**	-193.11536	-193.124691	-0.009331
**Ethanol**	-154.999516	-155.006442	-0.006926

Solvation free energies

Gibbs free energies was a sum of electron energies in gas phase, solvation free energies and thermal correction. The energies were given below.

Supplementary Table 5. Electron energies and Gibbs free energies (units are in a.u.).

	$E_{cc-pvdz}$	$E_{cc-pvtz}$	E_{CBS}	$G_{correct}$	G_{CBS}
**I**	-4037.826351	-4038.430611	-4038.627126	0.787969	-4038.056892
**TS-1**	-4385.681101	-4386.38484	-4386.613707	0.938857	-4385.89303
**TS-2**	-4385.684697	-4386.388513	-4386.617405	0.936564	-4385.899107
**TS-3**	-4192.700031	-4193.350944	-4193.562631	0.862181	-4192.923531
**TS-4**	-4192.70372	-4193.354365	-4193.565965	0.861155	-4192.926208
**Acetone**	-192.943599	-193.001905	-193.0208671	0.051772	-192.9784261
**Ethanol**	-154.86631	-154.920049	-154.9375258	0.050405	-154.8940468

Ball-and-stick models of I

As shown in **Supplementary Fig. 4**, The result of Computational study of **I** indicated that the nitrogen (Fig. S3, labeled N_3) and oxygen atom (Fig. S3, labeled O) of substrate **1a** coordinated to the copper atom (Fig. S3, labeled Cu) forming a square-planar complex I with a distortion, and the dihedral angel between plane O-Cu-N_2 and plane N_1-Cu-N_2 (Fig. S3, $\theta = 43°$).

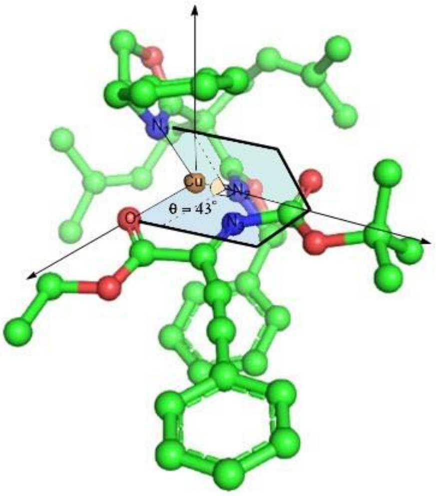

Supplementary Fig. 4. Ball-and-stick models of **I**.

As shown in **Supplementary Fig. 5**, if there are no interactions of alcohol with solvent or Boc group, the energy increases obviously when the oxygen atom of alcohol gets closer to the unsaturated carbon atom of imine.

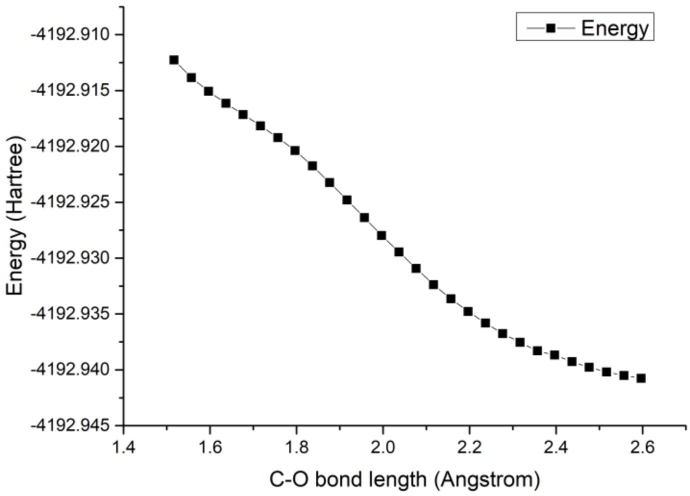

Supplementary Fig. 5. Relaxed scanning data of C-O bond between ethanol and β,γ-Alkynyl-α-Imino Esters

Fully-optimized geometries

I

Cartesian Coordinates:

2 2

C 3.888944 -0.842743 -3.027639
C 3.756878 -0.399257 -0.833873
C 2.460563 -1.137752 -2.524611
H 4.394862 -1.719022 -3.448270
H 2.219730 -2.206433 -2.620672
H 3.938926 -0.004917 -3.735583
O 4.616364 -0.448065 -1.834114
N 2.562042 -0.803275 -1.093825
C 4.299032 0.104511 0.467354
C 3.211194 0.404033 1.456388
O 3.637222 0.660297 2.682572
N 1.939185 0.473511 1.269900
C 2.468938 0.805556 3.528810
C 1.290889 0.850664 2.539543
H 2.591416 1.723323 4.113964
H 2.436383 -0.064419 4.199489
H 0.907500 1.874251 2.422733
C 5.079623 1.429821 0.193091
H 5.348606 1.858297 1.168318
H 6.019262 1.146140 -0.302176
C 5.221570 -1.001529 1.069376
H 5.937962 -1.292454 0.288738
H 5.799163 -0.526911 1.875223
C 4.309157 2.385591 -0.669306
H 4.432703 2.246011 -1.748579
C 3.495950 3.377069 -0.264288
C 4.439199 -2.163034 1.604310
H 4.083896 -2.053754 2.634719
C 4.123386 -3.298677 0.958448
C 3.243355 3.740588 1.166272
H 2.167345 3.648384 1.389029
H 3.809488 3.133546 1.883940
H 3.506754 4.796438 1.339377
C 2.770803 4.227292 -1.262563
H 1.682029 4.146514 -1.105297
H 3.031823 5.290224 -1.133596
H 3.004244 3.943095 -2.298140
C 4.561789 -3.630712 -0.435419
H 3.684589 -3.805883 -1.081871
H 5.187939 -2.857285 -0.897951
H 5.134896 -4.571970 -0.439177
C 3.301760 -4.356041 1.631615
H 2.382238 -4.559835 1.056006
H 3.853839 -5.308749 1.681062
H 3.020772 -4.072160 2.655317
C 1.335461 -0.321326 -3.119508
C 1.214331 1.038905 -2.795701
C 0.364921 -0.921784 -3.924909
C 0.132728 1.780921 -3.270051
H 1.968434 1.532474 -2.175195
C -0.710790 -0.176480 -4.402888
H 0.450560 -1.981417 -4.177526
C -0.831672 1.174035 -4.071544
H 0.053652 2.838849 -3.014830
H -1.458745 -0.651039 -5.040599
H -1.674027 1.755022 -4.451181
C 0.140276 -0.077186 2.831192
C 0.292816 -1.460313 2.672691
C -1.107942 0.437523 3.188068
C -0.797711 -2.310006 2.845812
H 1.266389 -1.878849 2.400997
C -2.197890 -0.412422 3.363945
H -1.230425 1.515701 3.316114
C -2.047409 -1.786603 3.182333
H -0.667839 -3.388320 2.731270
H -3.169753 -0.000456 3.640500
H -2.901047 -2.452664 3.319241
Cu 0.929535 -0.263341 -0.235943
N -0.913605 0.433880 -0.061548
C -1.894979 -0.423366 -0.213146
C -1.365241 -1.806914 -0.535826
O -0.160360 -1.975164 -0.724746
O -2.261975 -2.733463 -0.605497
C -1.807451 -4.090508 -0.905604
H -1.289346 -4.055566 -1.874916
H -1.077876 -4.366698 -0.130148
C -3.019665 -4.976276 -0.916915
H -3.731045 -4.665396 -1.695047
H -2.709627 -6.008638 -1.132367
H -3.525686 -4.968260 0.058787
C -3.266057 -0.264929 -0.142573
C -4.493291 -0.346765 -0.113320
C -5.888029 -0.473582 -0.078121
C -6.712428 0.636338 0.213112
C -6.467375 -1.737530 -0.337974

C -8.087791 0.477734 0.241855
H -6.256119 1.605497 0.411113
C -7.844198 -1.880013 -0.306038
H -5.817968 -2.584925 -0.560997
C -8.652125 -0.775448 -0.017110
H -8.730826 1.329684 0.465694
H -8.298635 -2.851080 -0.506015
H -9.737143 -0.893025 0.006542
C -1.065319 1.829103 0.139503
O -0.063547 2.508376 0.203891
O -2.314485 2.208249 0.219027
C -2.671247 3.654784 0.340820
C -2.115092 4.201997 1.643252
C -2.160976 4.393951 -0.883583
C -4.186721 3.605840 0.366828
H -2.472629 3.608885 2.498129
H -1.018596 4.222673 1.640545
H -2.476955 5.231468 1.778350
H -2.521384 3.910889 -1.803862
H -2.550889 5.421850 -0.867260
H -1.065242 4.443599 -0.901216
H -4.587925 4.624666 0.456103
H -4.576813 3.158704 -0.558620
H -4.537436 3.019820 1.228765

TS-1

Cartesian Coordinates:

2 2

C -1.760249 -4.458706 -0.898601
C -2.486363 -2.695603 0.276073
C -0.970792 -3.196261 -1.310357
H -2.379041 -4.871171 -1.705102
H -1.138819 -2.958593 -2.370582
H -1.128492 -5.245589 -0.468814
O -2.655063 -3.997408 0.140454
N -1.607752 -2.148086 -0.492806
C -3.336450 -2.072998 1.339736
C -3.120193 -0.595730 1.474072
O -3.962720 -0.004356 2.304365
N -2.270020 0.174088 0.884333
C -3.552159 1.379128 2.413671
C -2.597315 1.567380 1.224551
H -3.050964 1.493322 3.384084
H -4.454305 1.998628 2.368678
H -1.679091 2.072399 1.549866
C -2.999350 -2.769014 2.697361
H -3.560976 -2.238948 3.477664
H -3.412700 -3.786128 2.636773
C -4.835444 -2.301135 0.964798
H -4.976124 -3.382653 0.844286
H -5.426878 -1.992034 1.838998
C -1.530375 -2.823866 2.980465
H -0.990078 -3.647236 2.499553
C -0.820949 -1.988811 3.759991
C -5.255406 -1.528987 -0.246596
H -5.426500 -0.458530 -0.085850
C -5.455949 -2.003457 -1.488238
C -1.401425 -0.840713 4.525410
H -0.949060 0.100274 4.173927
H -2.492971 -0.764447 4.439793
H -1.154070 -0.936158 5.594893
C 0.657635 -2.171073 3.924808
H 1.191810 -1.279307 3.555909
H 0.920789 -2.279716 4.989442
H 1.028216 -3.057280 3.390642
C -5.307669 -3.440230 -1.888288
H -4.584201 -3.533815 -2.716607
H -4.987149 -4.097746 -1.071101
H -6.263457 -3.825083 -2.279598
C -5.896399 -1.084954 -2.588192
H -5.184669 -1.109862 -3.431064
H -6.867929 -1.408658 -2.996351
H -5.999114 -0.047620 -2.241808
C 0.516585 -3.282870 -1.058264
C 1.070658 -2.913260 0.171512
C 1.355100 -3.767126 -2.066303
C 2.444540 -3.021040 0.383969
H 0.430121 -2.548741 0.978949
C 2.726253 -3.886783 -1.847206
H 0.930862 -4.066232 -3.028882
C 3.275183 -3.510042 -0.621591
H 2.866700 -2.724473 1.345785
H 3.368723 -4.282354 -2.636269
H 4.349090 -3.598061 -0.449780
C -3.223358 2.304689 0.058112
C -3.475947 1.697609 -1.174030
C -3.608668 3.638015 0.246439
C -4.105771 2.408275 -2.197902
H -3.215855 0.649469 -1.333136

C -4.255734 4.340587 -0.766718
H -3.422309 4.128023 1.206427
C -4.504785 3.727421 -1.996329
H -4.304382 1.916885 -3.152082
H -4.578979 5.369185 -0.593653
H -5.018754 4.274441 -2.788484
Cu -0.803707 -0.389950 -0.268958
N 0.706020 0.790098 0.114840
C 1.708057 0.784723 -0.820944
C 1.232721 0.046185 -2.072050
O 0.111139 -0.460874 -2.109131
O 2.098043 0.009641 -3.027899
C 1.771210 -0.729366 -4.246801
H 1.149223 -1.586071 -3.957797
H 1.179396 -0.050515 -4.877965
C 3.070483 -1.136076 -4.880982
H 3.637793 -1.799765 -4.212703
H 2.862730 -1.679063 -5.814171
H 3.688114 -0.260979 -5.127015
C 3.045278 0.421793 -0.460223
C 4.118574 -0.096805 -0.208622
C 5.358101 -0.714018 0.097400
C 5.724378 -0.937417 1.436693
C 6.210026 -1.132643 -0.940720
C 6.924048 -1.573959 1.728181
H 5.059561 -0.610029 2.237215
C 7.409211 -1.764146 -0.637278
H 5.919060 -0.955957 -1.977216
C 7.765801 -1.986827 0.694165
H 7.208555 -1.747864 2.766884
H 8.073224 -2.085159 -1.441201
H 8.708892 -2.483869 0.927557
C 0.945803 1.314301 1.379200
O 0.194765 1.098510 2.312108
O 2.027293 2.081636 1.401112
C 2.498181 2.725943 2.647414
C 1.448370 3.710302 3.135639
C 2.834852 1.661028 3.677579
C 3.749228 3.449215 2.179754
H 1.156601 4.381637 2.315511
H 0.559179 3.190717 3.512605
H 1.871597 4.315488 3.950332
H 3.541943 0.930376 3.257241
H 3.314586 2.136165 4.545371
H 1.936566 1.135889 4.024217
H 4.222196 3.965920 3.026261
H 4.476176 2.739837 1.757987
H 3.498923 4.201474 1.417243
O 1.778251 2.337457 -1.533403
C 3.094975 2.901610 -1.779313
C 2.959889 4.254006 -2.431655
H 3.601771 2.183113 -2.435741
H 3.631763 2.951080 -0.821790
H 3.965730 4.634431 -2.662636
H 2.396437 4.194700 -3.373919
H 2.477433 4.981951 -1.764125
H 1.196784 2.984663 -0.942947
O 0.426980 4.008420 -0.245327
C -0.386242 4.712281 -0.853241
C -0.820853 4.399957 -2.243372
H -0.204457 3.616953 -2.699107
H -1.869300 4.057243 -2.197155
H -0.817675 5.307991 -2.864582
C -0.936181 5.932010 -0.197536
H -0.845545 5.866823 0.893211
H -0.331222 6.789545 -0.542019
H -1.971915 6.127494 -0.502894

TS-2

Cartesian Coordinates:

2 2

C 4.927257 1.358031 -1.896684
C 4.166658 -0.254071 -0.537936
C 3.382518 1.382883 -1.870420
H 5.345653 1.132480 -2.884846
H 2.967031 1.163087 -2.865203
H 5.382351 2.272300 -1.495302
O 5.283236 0.267054 -1.012749
N 3.069668 0.261204 -0.974033
C 4.359909 -1.369635 0.441720
C 3.077505 -1.826433 1.071554
O 3.207823 -2.899183 1.834939
N 1.894637 -1.330852 0.976008
C 1.901613 -3.222505 2.368353
C 0.963636 -2.157869 1.756727
H 1.969612 -3.169084 3.461733
H 1.658549 -4.245791 2.056196
H 0.527736 -1.521518 2.539015
C 5.304975 -0.850894 1.573015

H 5.327573 -1.621236 2.355776
H 6.315373 -0.796707 1.143526
C 4.975315 -2.586604 -0.319438
H 5.849362 -2.220405 -0.874443
H 5.345541 -3.283021 0.446003
C 4.890522 0.494030 2.093527
H 5.301379 1.353357 1.552425
C 4.073171 0.749520 3.130686
C 3.969666 -3.268260 -1.198210
H 3.341179 -4.014010 -0.699468
C 3.747723 -3.049779 -2.505643
C 3.445516 -0.297450 3.998065
H 2.349014 -0.256340 3.887290
H 3.792925 -1.314670 3.777926
H 3.662060 -0.090459 5.058115
C 3.725652 2.158335 3.504939
H 2.632274 2.300399 3.462949
H 4.031981 2.371661 4.541890
H 4.209666 2.894803 2.848479
C 4.531726 -2.092349 -3.350703
H 3.867346 -1.314095 -3.765591
H 5.351535 -1.600997 -2.812113
H 4.964836 -2.616216 -4.217930
C 2.672398 -3.800597 -3.232051
H 1.946423 -3.101743 -3.683601
H 3.097513 -4.385012 -4.064357
H 2.131055 -4.490646 -2.570337
C 2.776382 2.666400 -1.352427
C 2.615348 2.879888 0.022553
C 2.408381 3.673051 -2.249505
C 2.096647 4.087647 0.490234
H 2.919634 2.116077 0.743478
C 1.911758 4.886736 -1.779428
H 2.525552 3.513324 -3.324430
C 1.755741 5.096472 -0.408278
H 1.979602 4.242371 1.564208
H 1.649796 5.675454 -2.487221
H 1.375399 6.051333 -0.040758
C -0.139699 -2.735077 0.904414
C 0.036815 -2.964785 -0.464148
C -1.343846 -3.105079 1.508373
C -0.984196 -3.546262 -1.215857
H 0.984401 -2.708816 -0.945605
C -2.351889 -3.710460 0.762144
H -1.493302 -2.922453 2.575763
C -2.177433 -3.925666 -0.603777
H -0.833224 -3.727909 -2.282203
H -3.283989 -4.005655 1.246805
H -2.970172 -4.396080 -1.187941
Cu 1.311601 0.106540 -0.221168
N -0.433578 0.596512 0.424804
C -1.485755 0.456393 -0.430969
C -0.954669 0.066981 -1.815280
O 0.246760 0.138591 -2.065836
O -1.880984 -0.265810 -2.653682
C -1.505778 -0.515201 -4.041187
H -0.530586 -1.020255 -4.046801
H -2.281648 -1.205722 -4.391279
C -1.503174 0.780299 -4.811291
H -0.690661 1.442364 -4.477128
H -1.350424 0.569691 -5.879803
H -2.465024 1.299120 -4.692095
C -2.701865 -0.198678 -0.056473
C -3.740814 -0.789585 0.165519
C -4.966613 -1.464509 0.405680
C -5.494220 -1.551761 1.705549
C -5.659422 -2.048198 -0.670611
C -6.699946 -2.209363 1.919910
H -4.949338 -1.104536 2.538287
C -6.863859 -2.703609 -0.444675
H -5.234358 -1.986694 -1.673974
C -7.385028 -2.783772 0.848172
H -7.109152 -2.277610 2.928973
H -7.399495 -3.160870 -1.278172
H -8.330124 -3.300965 1.021893
C -0.590202 0.940743 1.759769
O 0.347853 0.856329 2.532768
O -1.806706 1.386086 2.033337
C -2.213006 1.706786 3.417462
C -2.094902 0.456519 4.274211
C -1.382170 2.864757 3.944854
C -3.665571 2.113040 3.239297
H -2.639593 -0.378528 3.806989
H -1.045946 0.168534 4.416867
H -2.539064 0.645830 5.261984
H -1.440683 3.723553 3.259243
H -1.781820 3.183402 4.918556
H -0.331885 2.578261 4.074479

H -4.109048 2.361192 4.213377
H -3.743928 2.997895 2.590816
H -4.245347 1.294499 2.788565
O -2.087365 2.006689 -0.828553
C -1.174656 3.064348 -1.189188
C -1.788858 4.383833 -0.794644
H -0.959062 3.007692 -2.268158
H -0.246585 2.866949 -0.633719
H -1.098019 5.199746 -1.048254
H -2.734019 4.563214 -1.327977
H -1.979007 4.410278 0.287826
H -2.944163 1.951716 -1.457794
O -4.073125 1.851654 -2.295674
C -5.203933 1.869278 -1.797421
C -6.387767 1.572270 -2.649581
H -6.803007 0.600350 -2.332523
H -7.182574 2.315793 -2.488899
H -6.108531 1.525515 -3.707959
C -5.431793 2.177214 -0.355168
H -5.961078 3.142417 -0.286650
H -6.099436 1.424079 0.090084
H -4.494110 2.228302 0.208649

TS-3

Cartesian Coordinates:
2 2
C -1.987747 -0.920684 -4.071075
C -2.885476 -0.392787 -2.087745
C -0.929396 -0.218625 -3.195349
H -2.191519 -0.406243 -5.016768
H -0.651801 0.758438 -3.618161
H -1.757846 -1.977503 -4.261514
O -3.201927 -0.876392 -3.275396
N -1.674166 0.016410 -1.946541
C -3.992908 -0.332955 -1.085384
C -3.486621 -0.171973 0.320354
O -4.445164 -0.117422 1.227005
N -2.277852 -0.052563 0.755697
C -3.828606 0.127560 2.514397
C -2.314062 0.143046 2.216236
H -4.138016 -0.681040 3.187133
H -4.205271 1.089104 2.886101
H -1.814246 -0.716003 2.682139
C -4.847800 -1.626087 -1.170792
H -5.658178 -1.528136 -0.438172
H -5.315567 -1.628638 -2.167115
C -4.870363 0.922297 -1.430313
H -5.124637 0.852303 -2.497252
H -5.808980 0.802832 -0.870650
C -4.056732 -2.881437 -0.974908
H -3.403955 -3.161073 -1.810228
C -4.118594 -3.728663 0.067533
C -4.223029 2.223742 -1.073178
H -4.388407 2.562536 -0.044547
C -3.486192 3.012776 -1.874363
C -4.984230 -3.532921 1.275100
H -4.375527 -3.592143 2.193474
H -5.526825 -2.579931 1.284234
H -5.722259 -4.348221 1.350716
C -3.336837 -5.007512 0.052721
H -2.739141 -5.123857 0.970385
H -4.023622 -5.869792 0.021939
H -2.669168 -5.075818 -0.817250
C -3.207590 2.727613 -3.318406
H -2.120158 2.712378 -3.502895
H -3.636899 1.782126 -3.673665
H -3.613260 3.535571 -3.948793
C -2.897000 4.290938 -1.358708
H -1.794719 4.256931 -1.412039
H -3.215201 5.146229 -1.976314
H -3.192545 4.493716 -0.320081
C 0.325461 -1.004699 -2.901536
C 0.278581 -2.114413 -2.047605
C 1.550712 -0.614151 -3.447552
C 1.442023 -2.820461 -1.749143
H -0.671847 -2.436281 -1.611347
C 2.711863 -1.325599 -3.152987
H 1.594205 0.250241 -4.115246
C 2.659635 -2.428704 -2.302179
H 1.393515 -3.686745 -1.086964
H 3.664361 -1.019169 -3.588963
H 3.571466 -2.982231 -2.072048
C -1.610269 1.410621 2.632723
C -1.697043 2.567813 1.850291
C -0.902374 1.450090 3.835512
C -1.089179 3.747868 2.271340
H -2.252902 2.552583 0.909559
C -0.302601 2.634151 4.261059
H -0.824811 0.552419 4.452589

C -0.397406 3.785086 3.481498
H -1.172582 4.648495 1.659745
H 0.232868 2.658130 5.211872
H 0.055856 4.717074 3.824036
Cu -0.689577 0.329254 -0.325068
N 0.886488 0.139412 0.803511
C 1.964519 0.920058 0.473454
C 1.576658 1.915453 -0.608744
O 0.479160 1.835570 -1.157599
O 2.489791 2.781988 -0.893066
C 2.201298 3.753019 -1.951800
H 1.930775 3.179625 -2.850024
H 1.324980 4.334553 -1.629776
C 3.431145 4.593156 -2.138834
H 4.293024 3.976169 -2.428764
H 3.247532 5.323314 -2.939856
H 3.681744 5.148460 -1.224126
C 3.241097 0.319542 0.270997
C 4.248754 -0.294614 -0.039683
C 5.399377 -1.038655 -0.393976
C 5.622142 -2.297069 0.196486
C 6.303930 -0.538380 -1.348365
C 6.735725 -3.040851 -0.169541
H 4.917682 -2.672673 0.940071
C 7.412389 -1.293505 -1.707474
H 6.127144 0.440257 -1.796738
C 7.628291 -2.541797 -1.120537
H 6.914620 -4.014175 0.289497
H 8.116840 -0.908102 -2.445852
H 8.502895 -3.129790 -1.404056
C 1.079699 -0.680923 1.891575
O 1.879892 -0.394695 2.788299
O 0.303021 -1.739053 1.864435
C 0.452435 -2.839576 2.858683
C 1.856255 -3.409508 2.741332
C 0.139079 -2.341877 4.259120
C -0.590429 -3.831095 2.378927
H 2.069022 -3.692387 1.699533
H 2.611914 -2.692219 3.085652
H 1.930095 -4.314123 3.361624
H -0.883168 -1.938441 4.316309
H 0.192920 -3.191083 4.955472
H 0.854068 -1.579508 4.589617
H -0.594046 -4.715201 3.031363
H -1.594188 -3.381375 2.398245
H -0.376239 -4.156696 1.351132
O 2.162552 1.951081 1.884153
C 3.422024 2.647956 2.117167
C 3.179159 4.132418 2.128599
H 4.126173 2.335341 1.330458
H 3.809597 2.283231 3.079241
H 4.132359 4.655216 2.295460
H 2.764096 4.473610 1.170361
H 2.485276 4.413152 2.932088
H 2.111770 1.130689 2.495253

TS-4

Cartesian Coordinates:
2 2
C 4.262780 2.575063 0.258282
C 3.585415 0.650501 -0.667327
C 2.721581 2.550935 0.182080
H 4.719848 3.410159 -0.284259
H 2.347641 3.311584 -0.519528
H 4.643639 2.542803 1.287739
O 4.674504 1.346960 -0.397200
N 2.464338 1.224375 -0.400963
C 3.810639 -0.688173 -1.295770
C 2.580594 -1.549482 -1.282885
O 2.728259 -2.705912 -1.903784
N 1.415357 -1.311220 -0.786282
C 1.445612 -3.382083 -1.894443
C 0.513459 -2.427936 -1.117613
H 1.589731 -4.351510 -1.402409
H 1.143235 -3.532540 -2.938563
H 0.179495 -2.878592 -0.175697
C 4.956563 -1.425757 -0.551442
H 5.092498 -2.396390 -1.044003
H 5.874114 -0.844374 -0.731809
C 4.203900 -0.438560 -2.793723
H 5.030592 0.285145 -2.793351
H 4.610395 -1.389143 -3.167672
C 4.725285 -1.566466 0.920375
H 4.778996 -0.634991 1.496977
C 4.554259 -2.707402 1.611642
C 3.052058 -0.001850 -3.644549
H 2.435766 -0.810630 -4.052313
C 2.708467 1.258304 -3.963021
C 4.512384 -4.073595 0.996717

H	3.643199	-4.638472	1.371221
H	4.467045	-4.063442	-0.099398
H	5.401644	-4.652861	1.295747
C	4.466769	-2.680000	3.108482
H	3.566848	-3.202572	3.470099
H	5.326486	-3.212866	3.547766
H	4.465703	-1.655263	3.505351
C	3.463872	2.476892	-3.528823
H	2.803866	3.156456	-2.962872
H	4.346105	2.255201	-2.915113
H	3.803973	3.046702	-4.408669
C	1.516897	1.529569	-4.830996
H	0.783214	2.160147	-4.298791
H	1.810815	2.089917	-5.733370
H	1.018742	0.604557	-5.152168
C	1.995715	2.699038	1.496867
C	1.934563	1.630181	2.400689
C	1.373134	3.905478	1.822789
C	1.258710	1.772431	3.611477
H	2.432152	0.682959	2.169354
C	0.705156	4.049134	3.036941
H	1.423631	4.745684	1.125520
C	0.644881	2.981822	3.931478
H	1.224421	0.939601	4.315028
H	0.236323	5.002214	3.288469
H	0.129937	3.096368	4.886936
C	-0.699306	-1.953010	-1.879565
C	-0.627448	-0.863805	-2.754557
C	-1.916046	-2.619268	-1.718936
C	-1.761590	-0.443618	-3.448663
H	0.321636	-0.343099	-2.907711
C	-3.043151	-2.213512	-2.429769
H	-1.983724	-3.465567	-1.030487
C	-2.970372	-1.119232	-3.290008
H	-1.692913	0.404698	-4.132959
H	-3.987056	-2.746014	-2.302939
H	-3.856291	-0.798512	-3.840667
Cu	0.732815	0.414929	-0.183751
N	-0.939597	0.045491	0.742385
C	-1.979803	0.905660	0.468700
C	-1.509677	2.015306	-0.462453
O	-0.323602	2.098941	-0.776843
O	-2.445532	2.821063	-0.839770
C	-2.079621	3.950706	-1.696709
H	-1.301096	3.605660	-2.389955
H	-3.003725	4.154626	-2.249364
C	-1.636072	5.123808	-0.862458
H	-0.730889	4.884999	-0.286055
H	-1.407360	5.969216	-1.528001
H	-2.429557	5.444691	-0.172594
C	-3.278939	0.385281	0.230438
C	-4.337759	-0.141668	-0.069966
C	-5.550812	-0.779594	-0.421588
C	-5.857894	-2.040406	0.124063
C	-6.435087	-0.171068	-1.331232
C	-7.034792	-2.679542	-0.240768
H	-5.167955	-2.500569	0.832810
C	-7.606760	-0.823331	-1.691304
H	-6.190719	0.807387	-1.746641
C	-7.906696	-2.074119	-1.148362
H	-7.279040	-3.654078	0.184279
H	-8.294694	-0.354921	-2.396524
H	-8.830709	-2.580568	-1.432507
C	-1.080770	-0.741034	1.855713
O	-1.827981	-0.429139	2.794696
O	-0.303523	-1.797756	1.828504
C	-0.194554	-2.728577	2.986357
C	0.407841	-1.983799	4.164383
C	-1.554458	-3.331548	3.291317
C	0.763414	-3.778110	2.450693
H	1.383534	-1.554265	3.891220
H	-0.259954	-1.189291	4.520197
H	0.569630	-2.689278	4.991997
H	-1.993641	-3.767182	2.381283
H	-1.427975	-4.141714	4.023929
H	-2.246346	-2.589914	3.706213
H	1.023682	-4.486322	3.249479
H	0.303385	-4.350990	1.631677
H	1.686087	-3.305411	2.084839
O	-2.107200	1.875873	1.933457
C	-3.342381	2.582114	2.215138
C	-3.381822	2.927898	3.682628
H	-4.188229	1.942488	1.917996
H	-3.329443	3.474424	1.576752
H	-4.308125	3.481005	3.896481
H	-3.381369	2.022104	4.307628
H	-2.528296	3.559048	3.966297
H	-2.059474	1.016326	2.521499

Acetone	Ethanol
Cartesian Coordinates:	Cartesian Coordinates:
0 1	0 1
C -0.000015 -0.611402 1.282967	C -1.214127 -0.219221 -0.000007
C -0.000015 0.184621 0.000000	C 0.089611 0.543947 0.000006
H 0.000977 0.068517 2.143378	H -1.280502 -0.862783 0.890200
H -0.884716 -1.266876 1.326163	H -2.073149 0.468457 0.000050
H 0.883584 -1.268405 1.325278	H -1.280544 -0.862695 -0.890273
O 0.000072 1.395328 0.000000	H 0.137905 1.202448 -0.891082
C -0.000015 -0.611402 -1.282967	H 0.137893 1.202443 0.891098
H -0.884716 -1.266876 -1.326163	O 1.141644 -0.398372 0.000014
H 0.000977 0.068517 -2.143378	H 1.972341 0.090753 -0.000101
H 0.883584 -1.268405 -1.325278	

Supplementary Figures

NMR Spectra

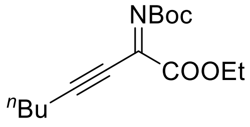

1n

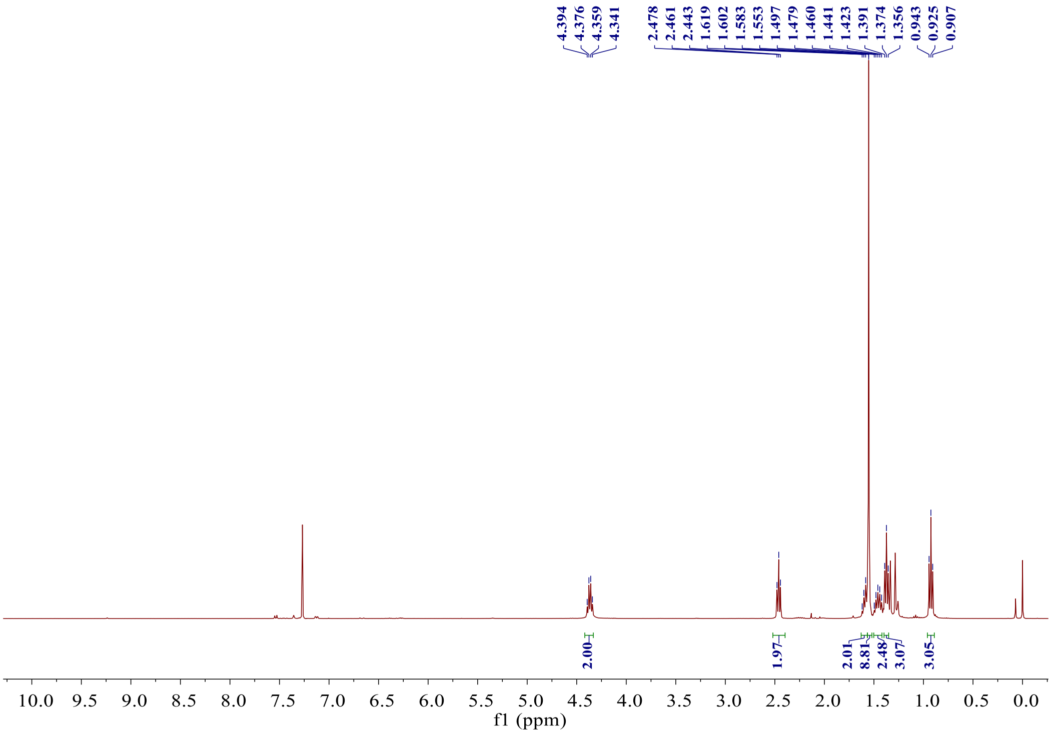

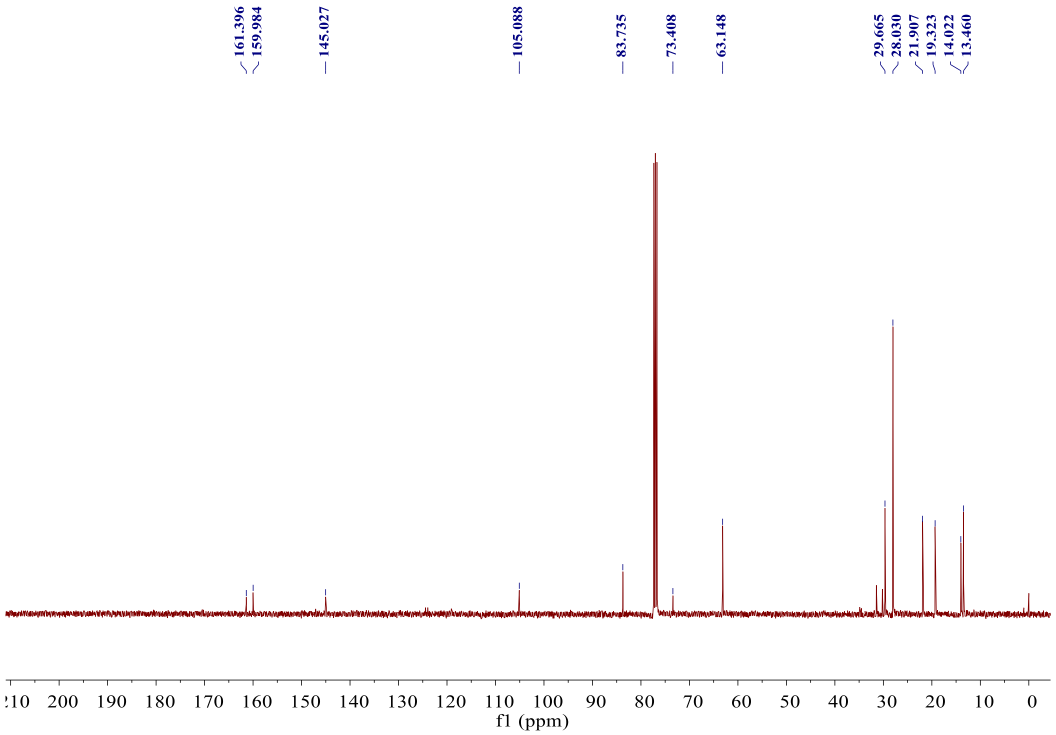

Supplementary Fig. 6. ^{1}H NMR & ^{13}C NMR spectra of compound **1n** in $CDCl_3$

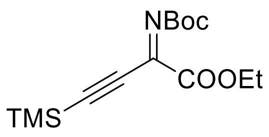

1o

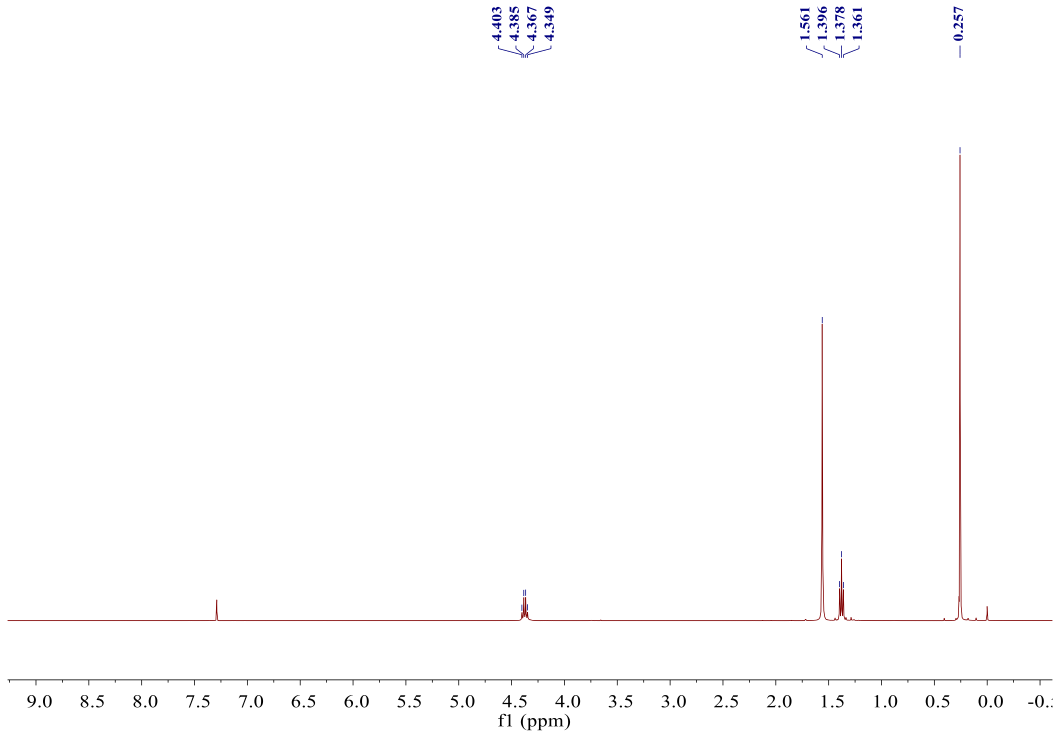

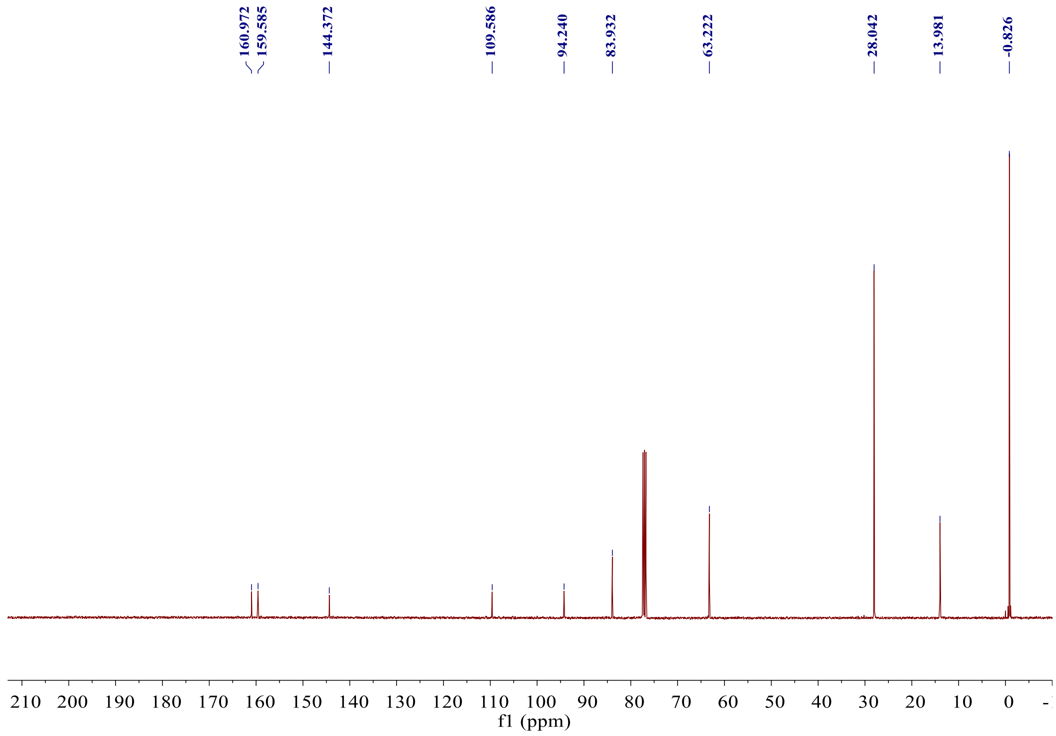

Supplementary Fig. 7. ^{1}H NMR & ^{13}C NMR spectra of compound **1o** in $CDCl_3$

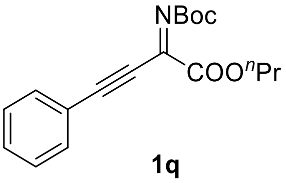

1q

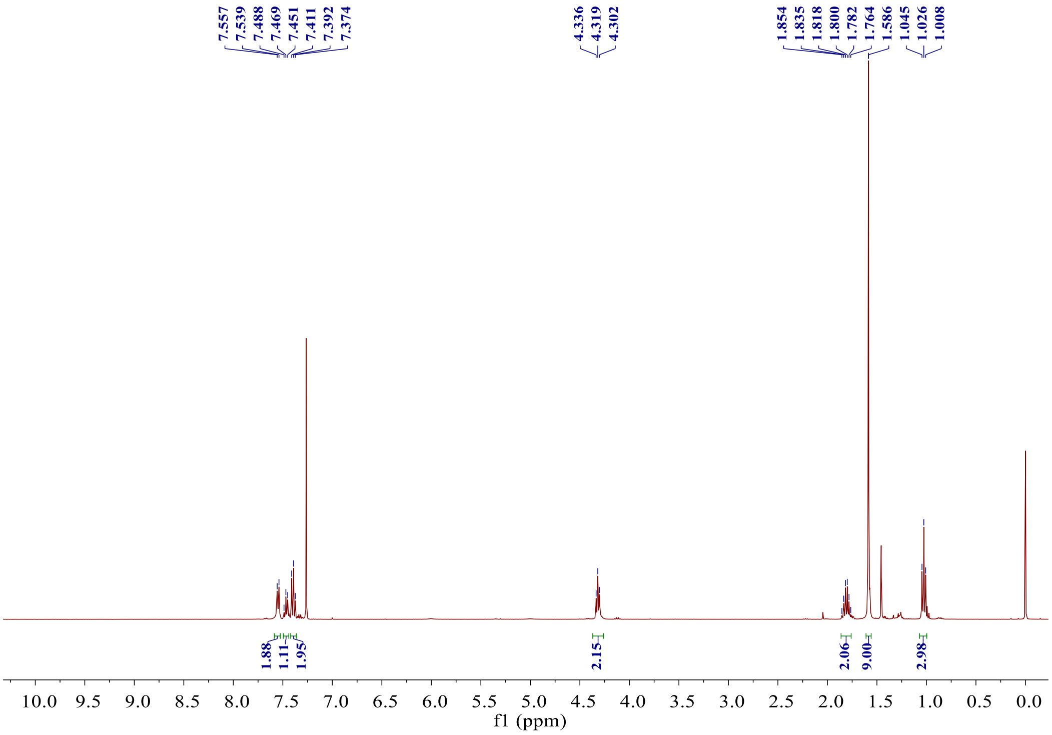

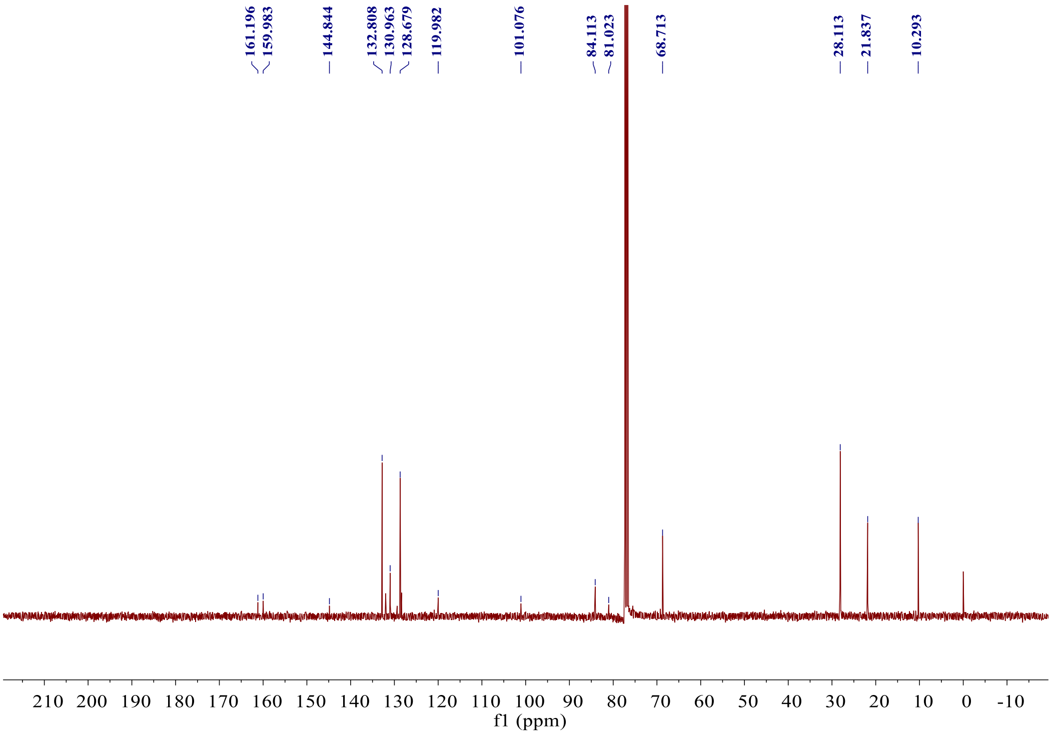

Supplementary Fig. 8. ^{1}H NMR & ^{13}C NMR spectra of compound **1q** in $CDCl_3$

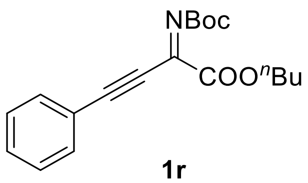

1r

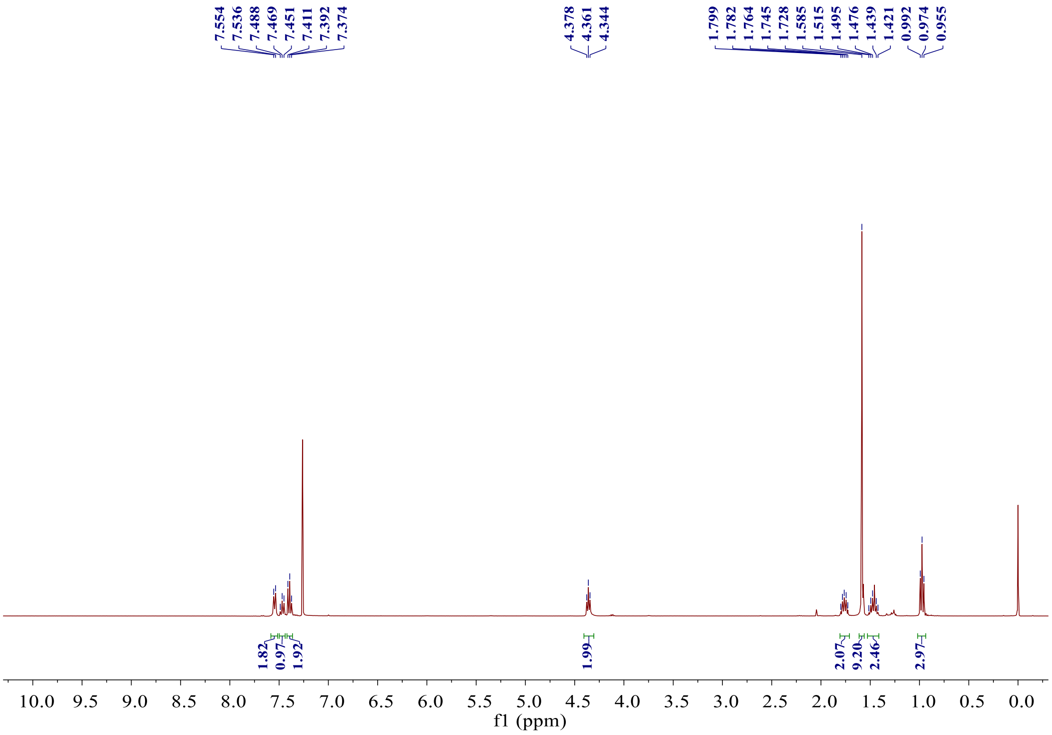

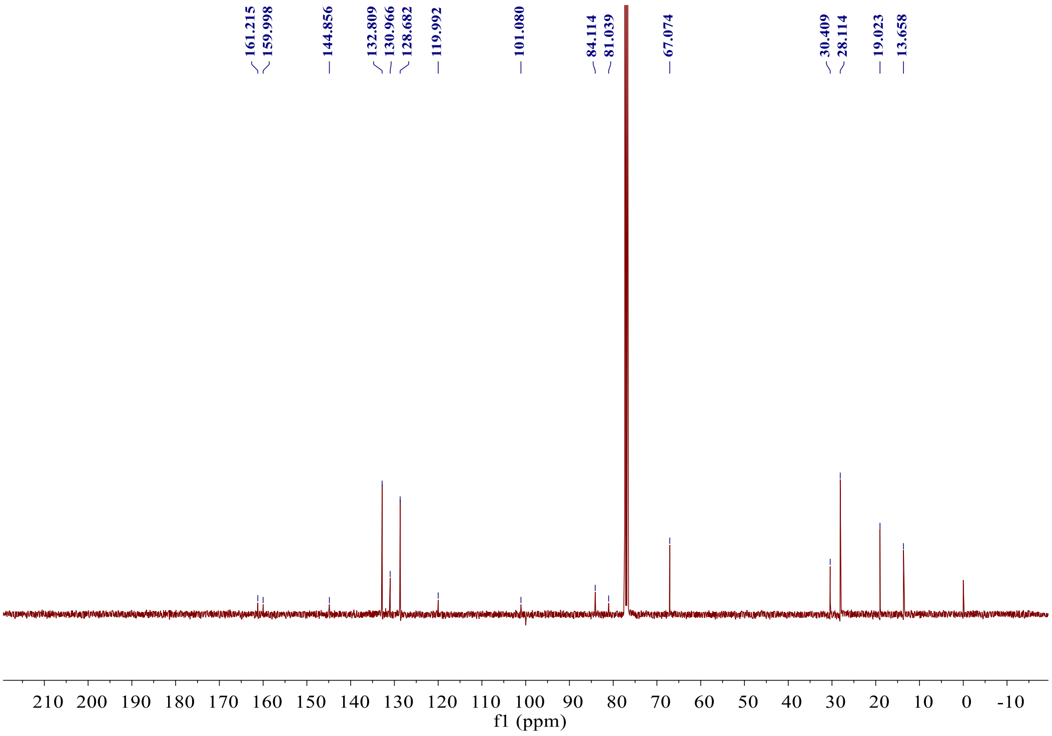

Supplementary Fig. 9. ^{1}H NMR & ^{13}C NMR spectra of compound **1r** in $CDCl_3$

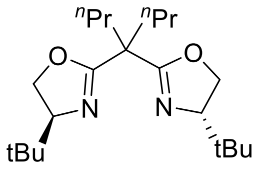

L1

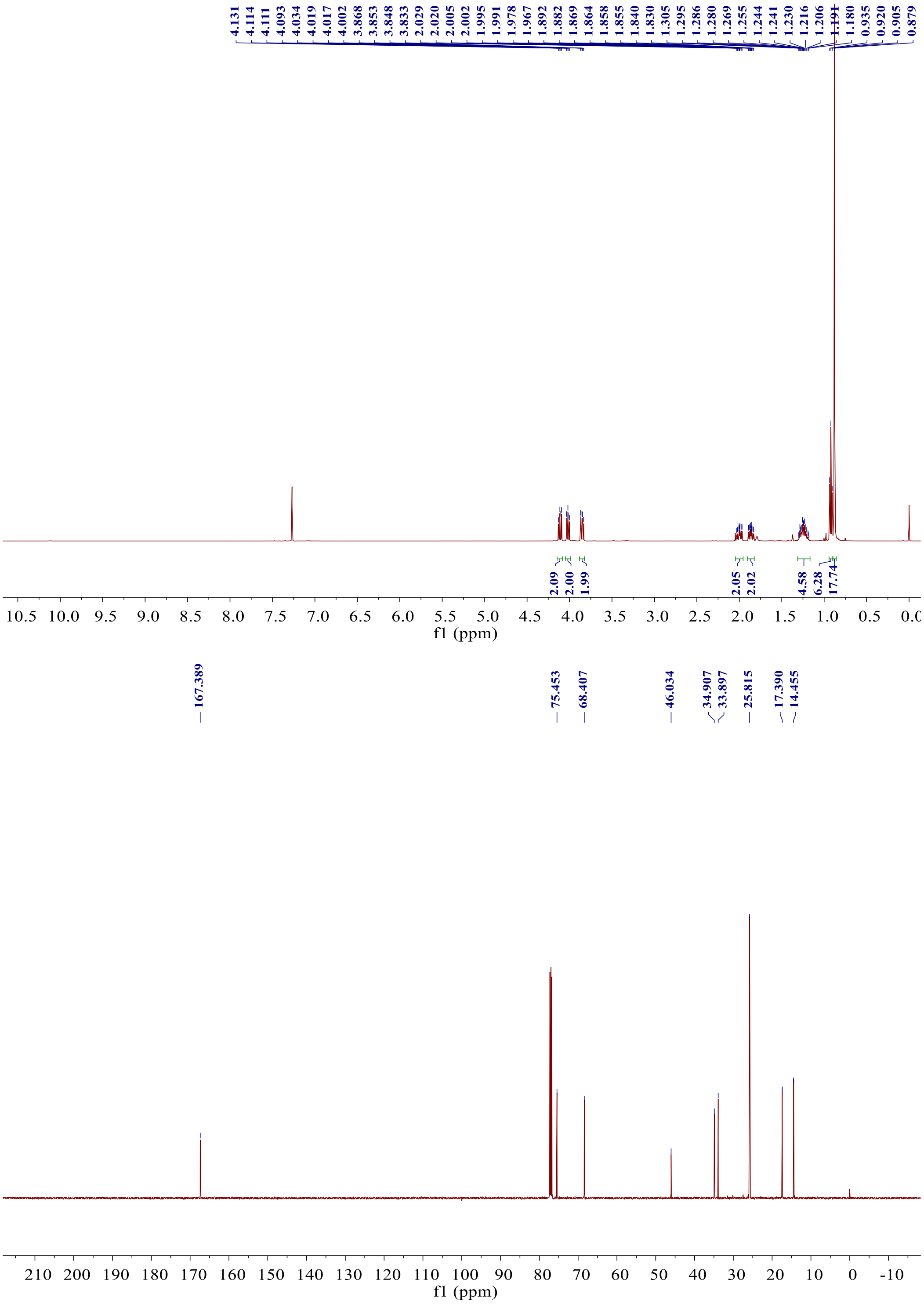

Supplementary Fig. 10. ^{1}H NMR & ^{13}C NMR spectra of compound **L1** in $CDCl_3$

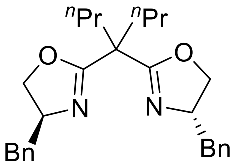

L2

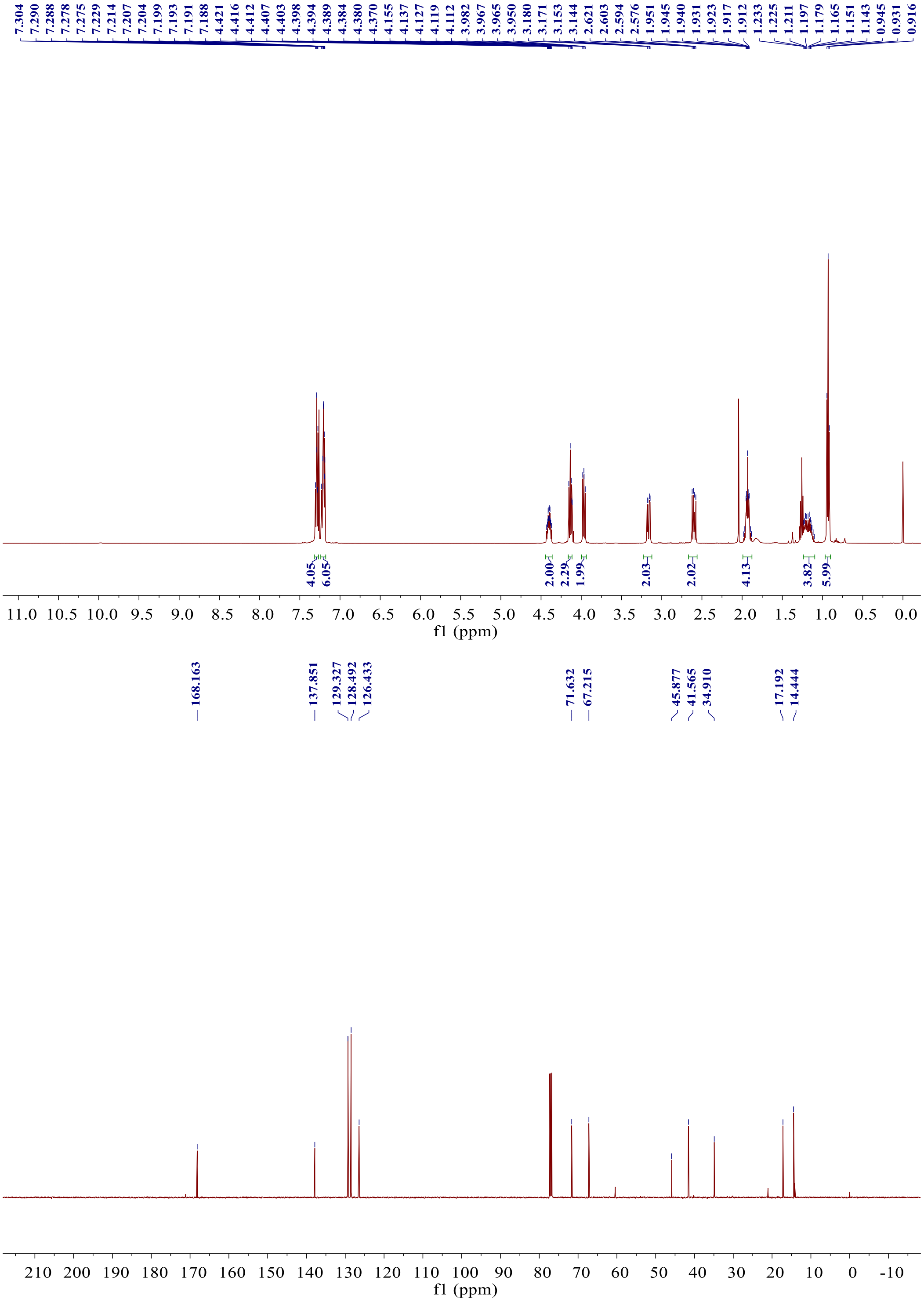

Supplementary Fig. 11. ^{1}H NMR & ^{13}C NMR spectra of compound **L2** in $CDCl_3$

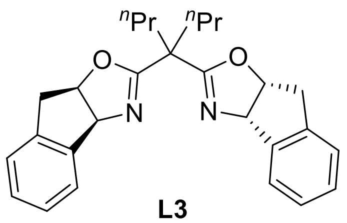

L3

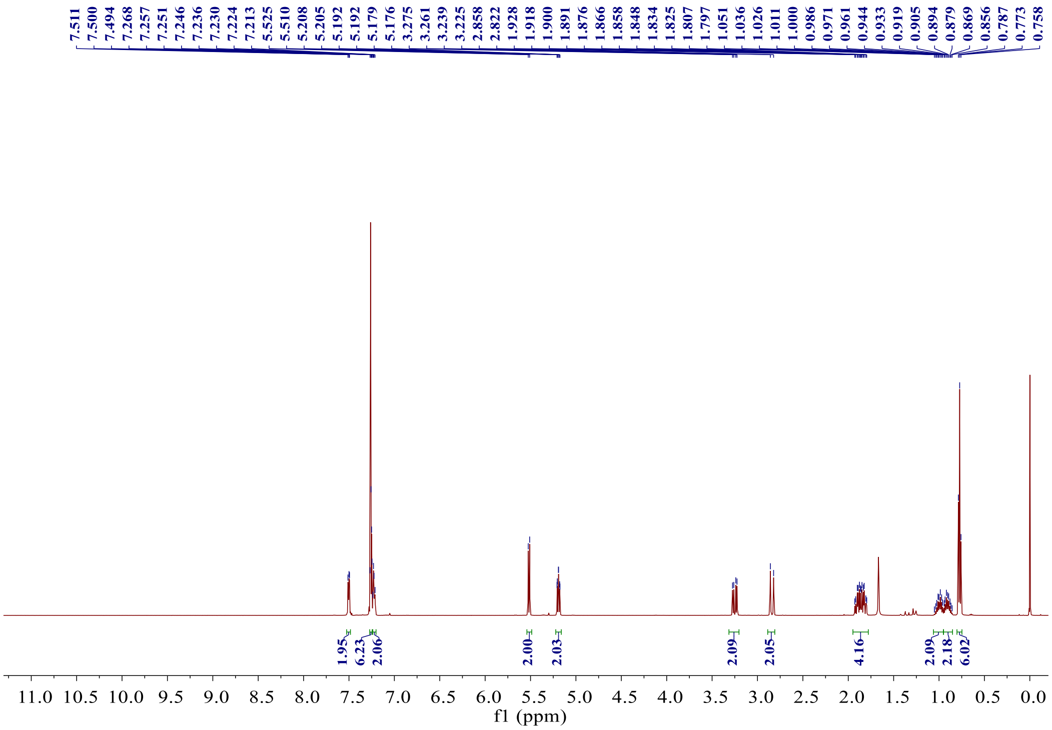

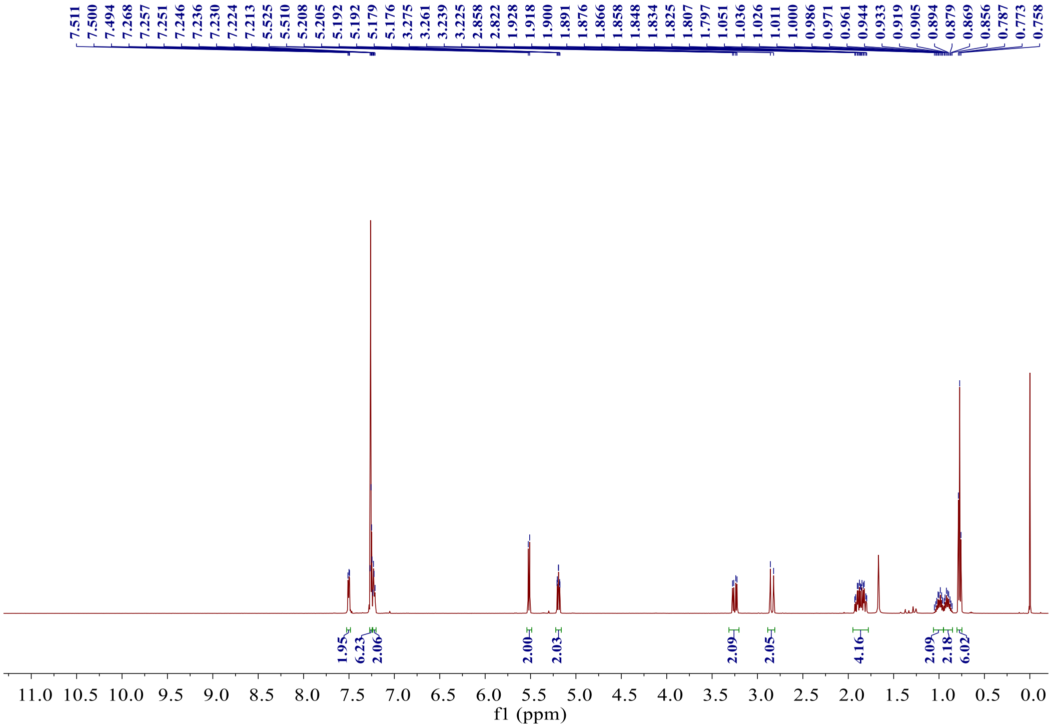

Supplementary Fig. 12. ^{1}H NMR & ^{13}C NMR spectra of compound **L3** in $CDCl_3$

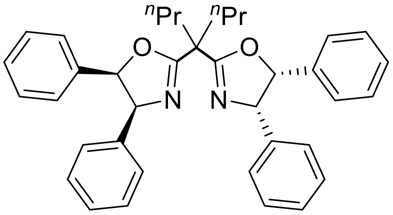

L4

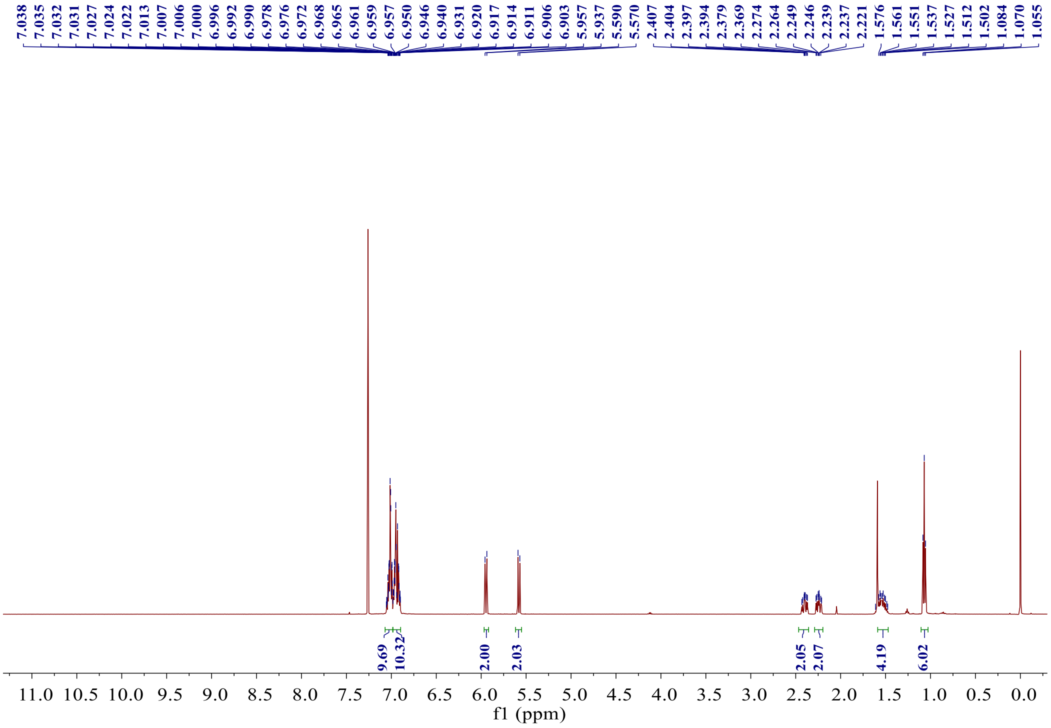

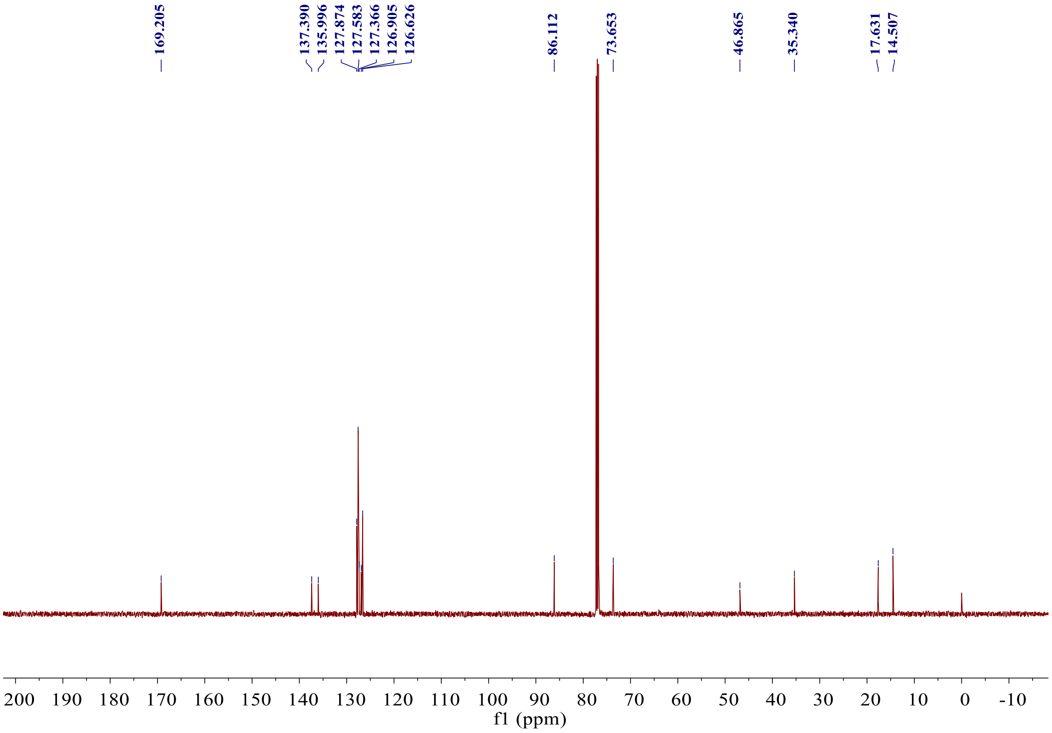

Supplementary Fig. 13. ^{1}H NMR & ^{13}C NMR spectra of compound **L4** in $CDCl_3$

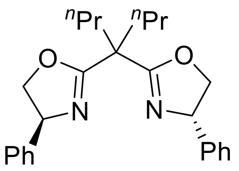

L5

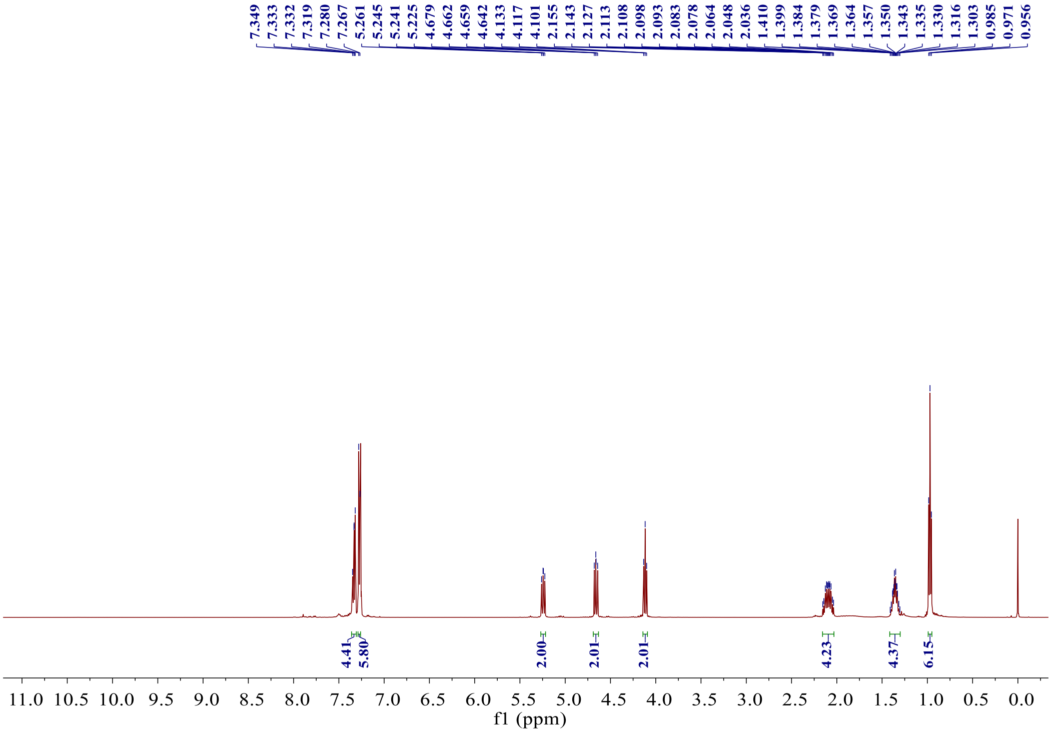

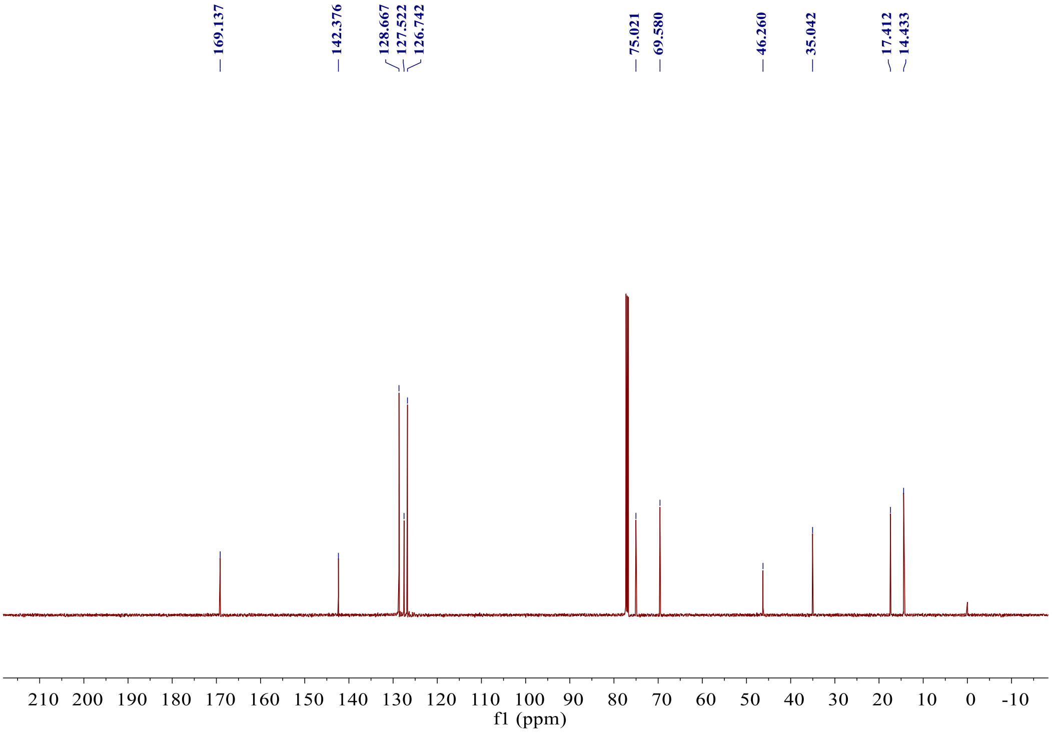

Supplementary Fig. 14. ^{1}H NMR & ^{13}C NMR spectra of compound **L5** in $CDCl_3$

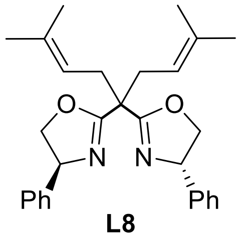

L8

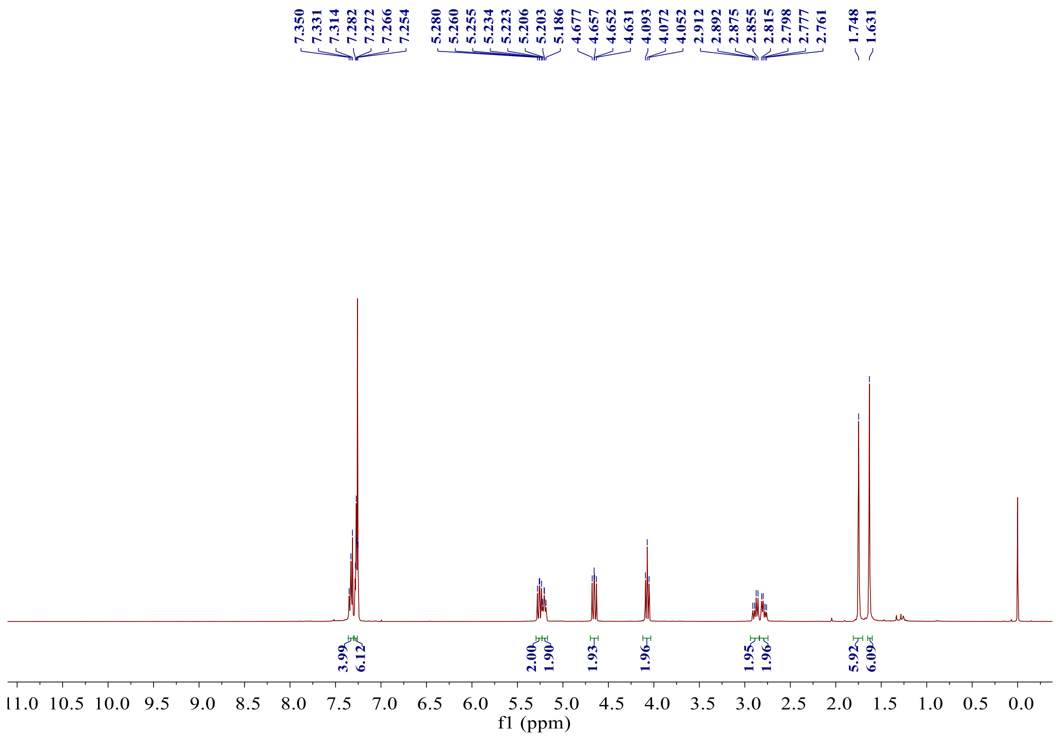

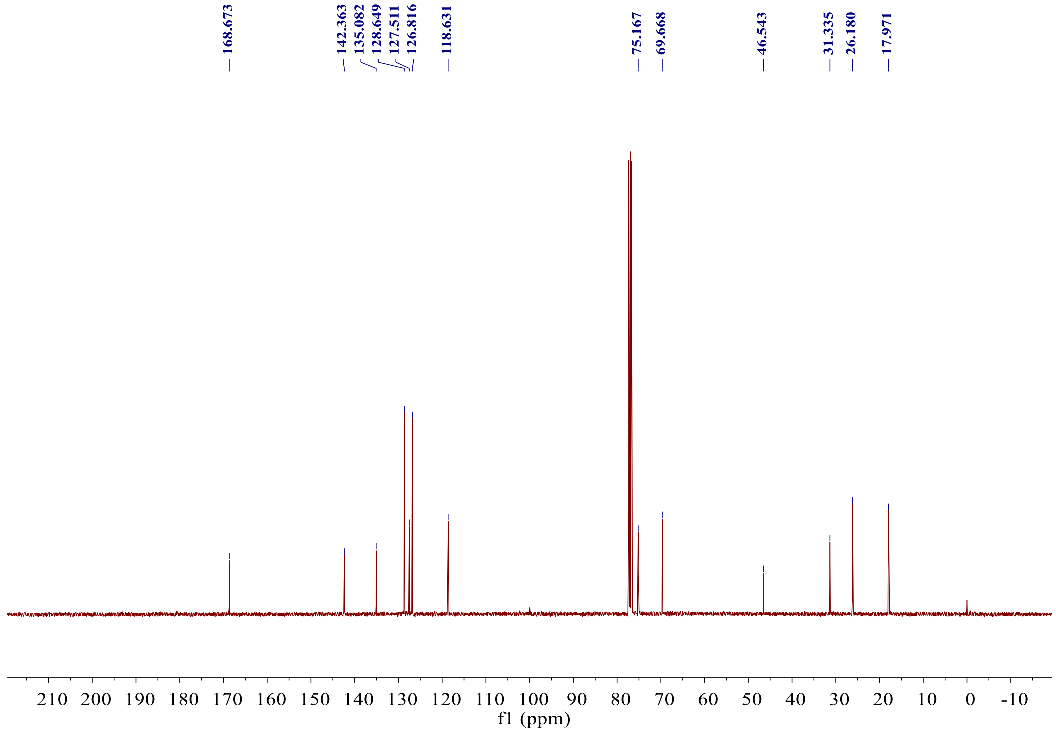

Supplementary Fig. 15. ^{1}H NMR & ^{13}C NMR spectra of compound **L8** in $CDCl_3$

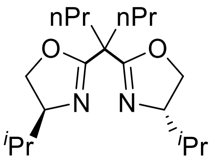

L9

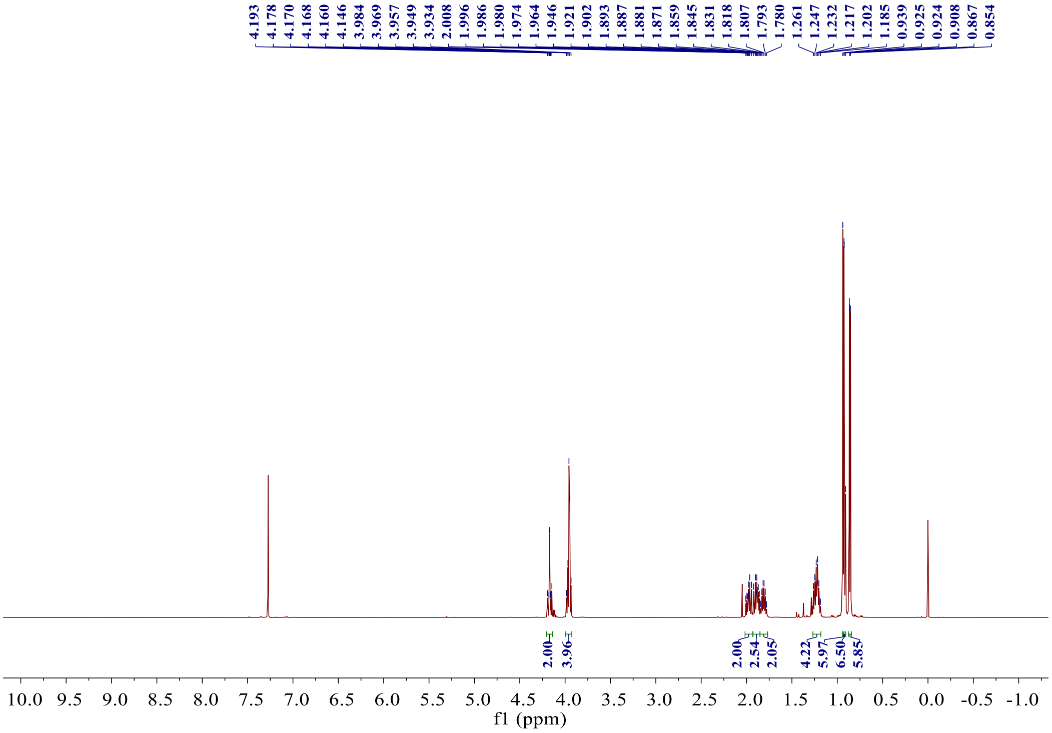

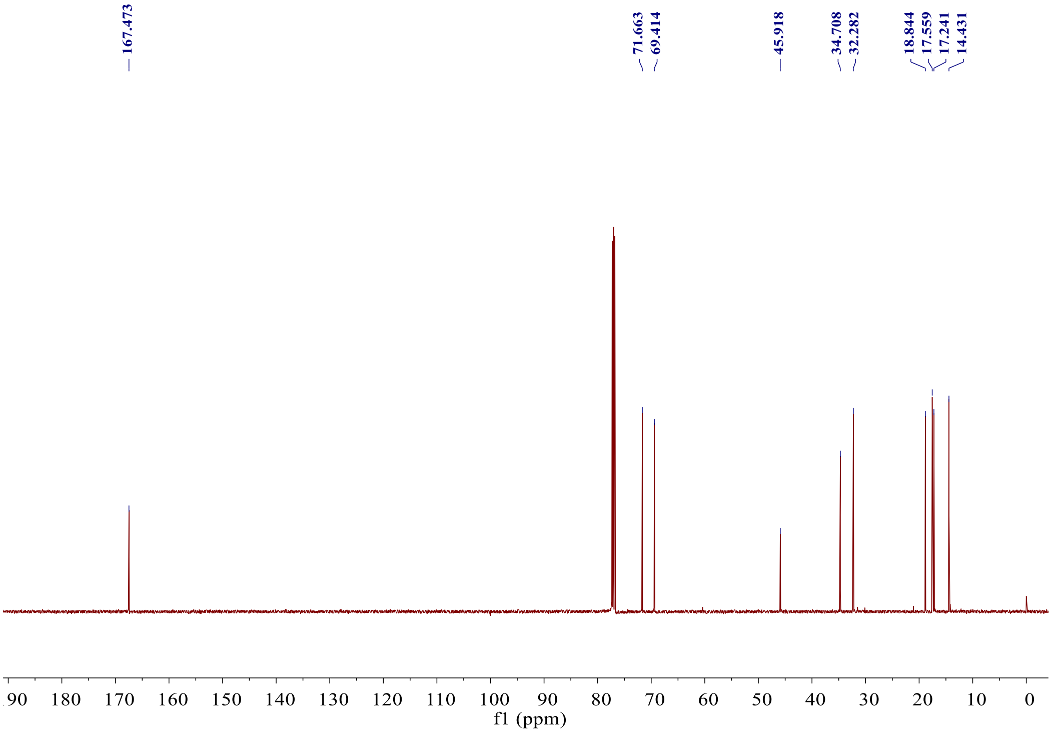

Supplementary Fig. 16. ^{1}H NMR & ^{13}C NMR spectra of compound **L9** in $CDCl_3$

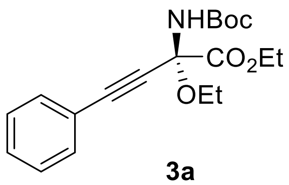

3a

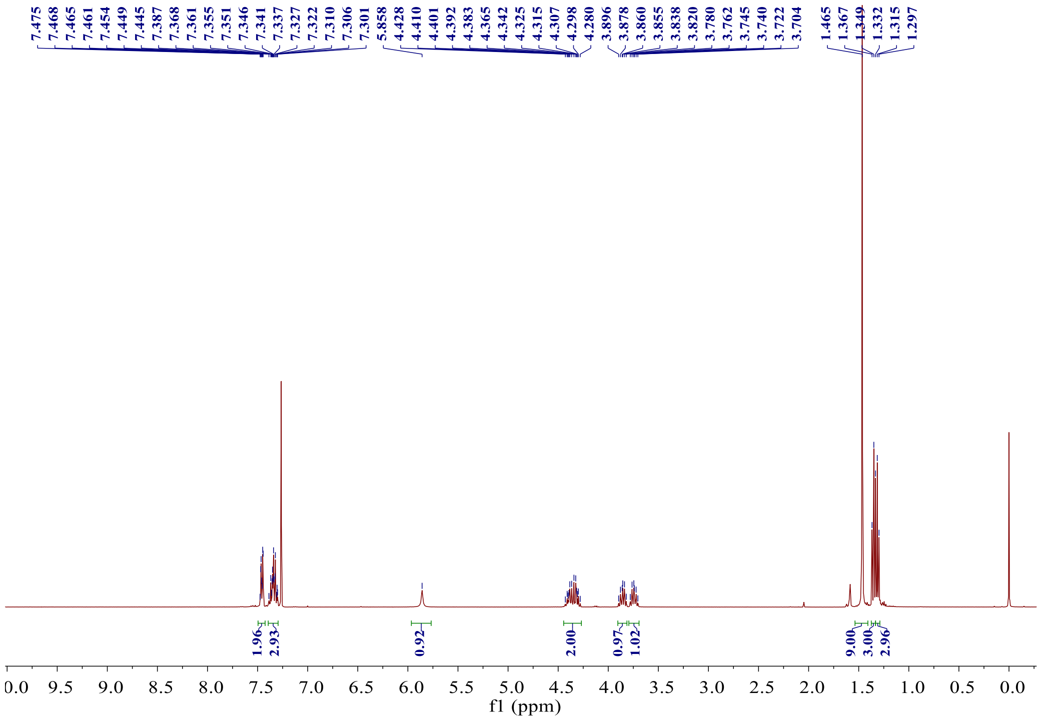

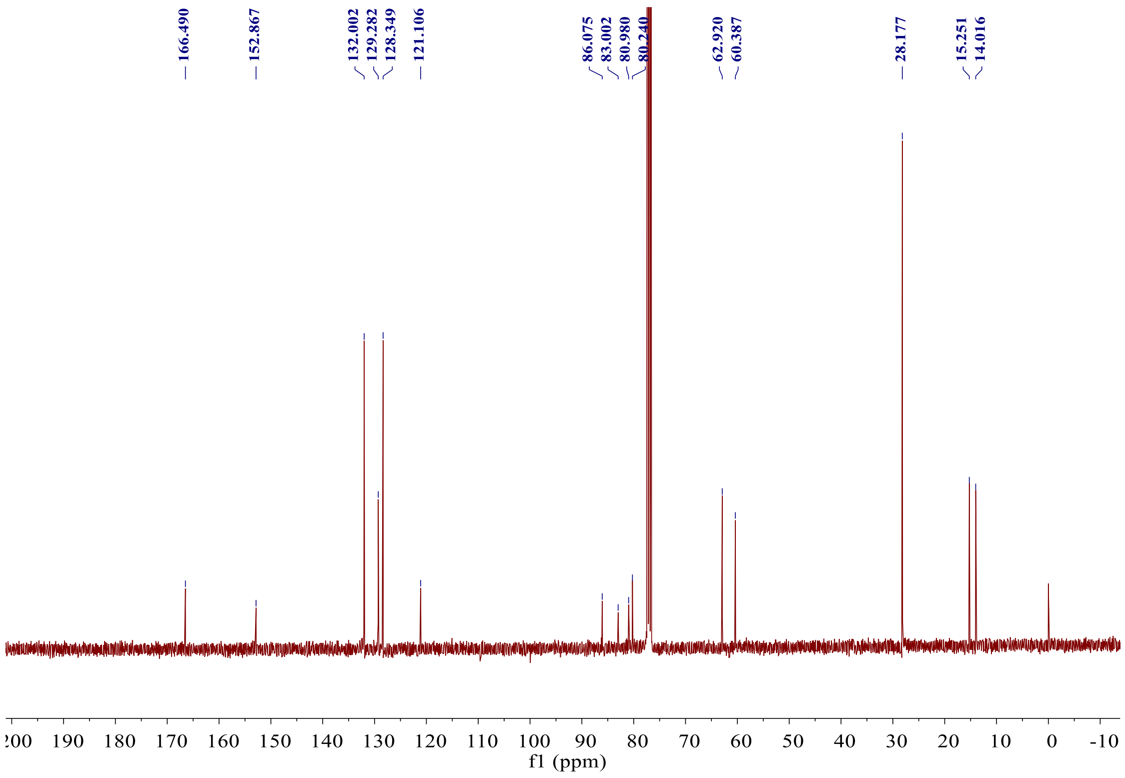

Supplementary Fig. 17. ^{1}H NMR & ^{13}C NMR spectra of compound **3a** in $CDCl_3$

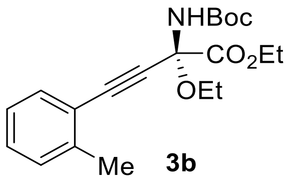

3b

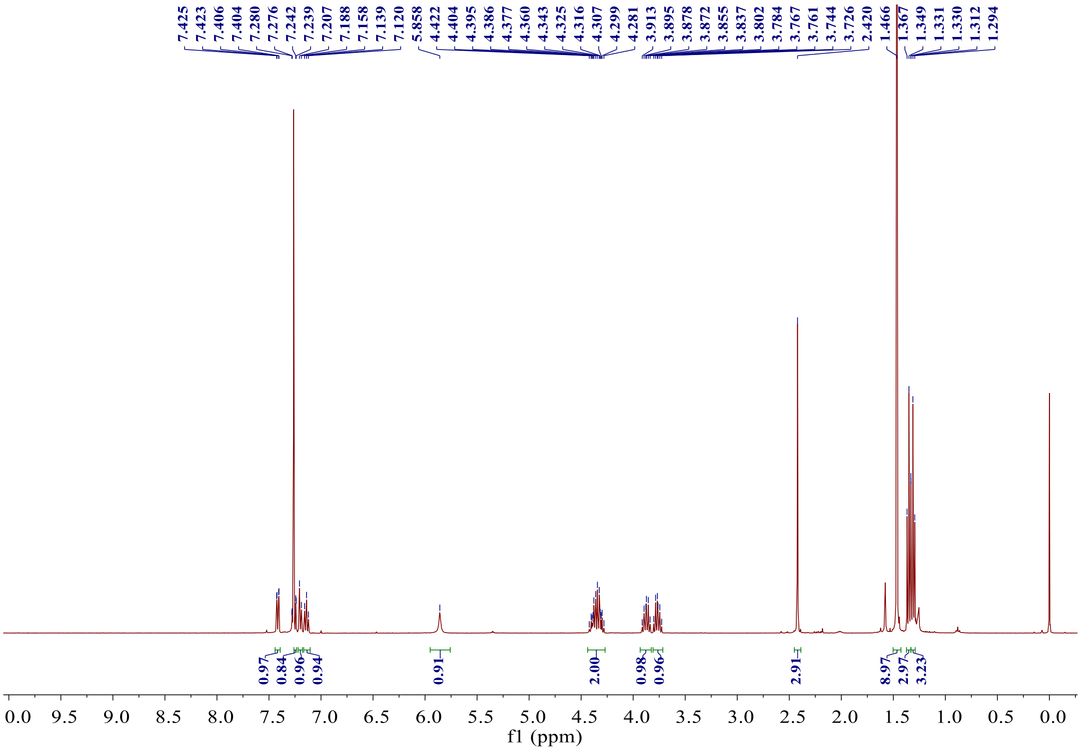

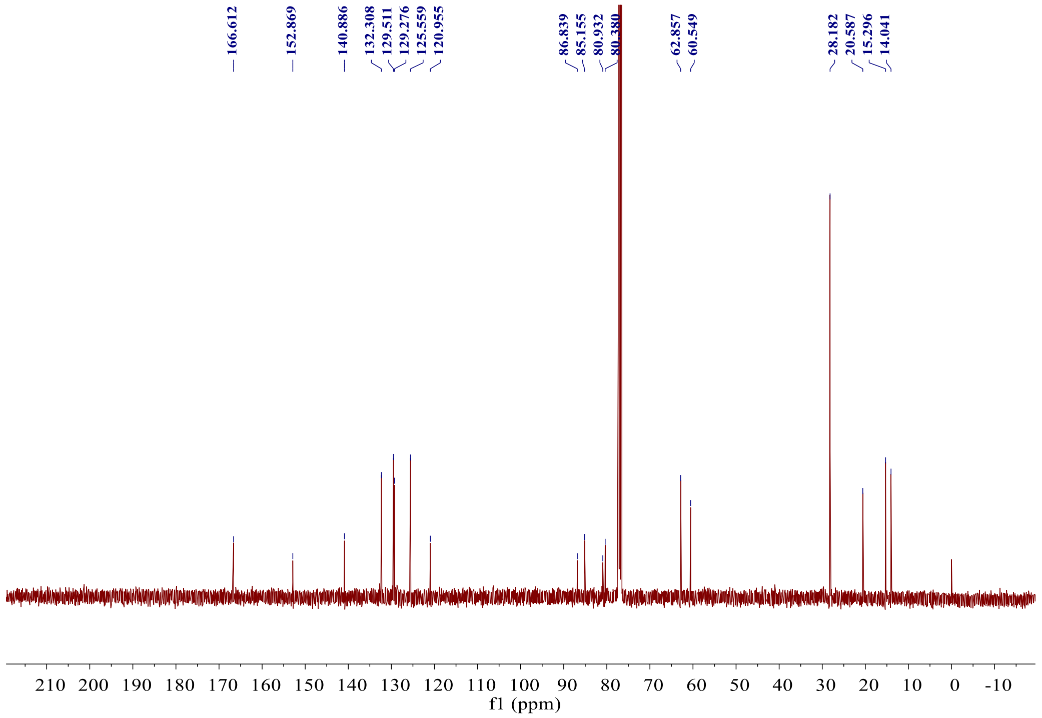

Supplementary Fig. 18. ^{1}H NMR & ^{13}C NMR spectra of compound **3b** in $CDCl_3$

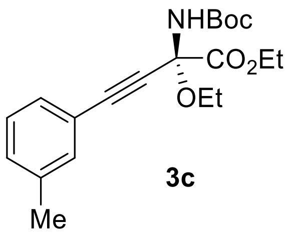

3c

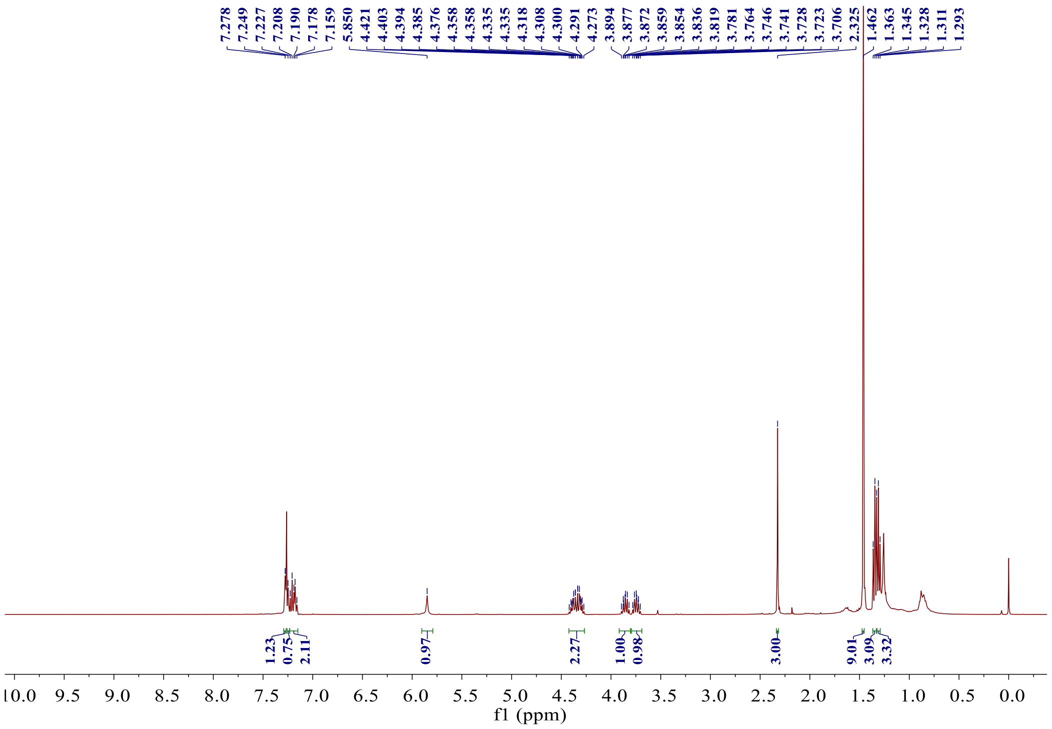

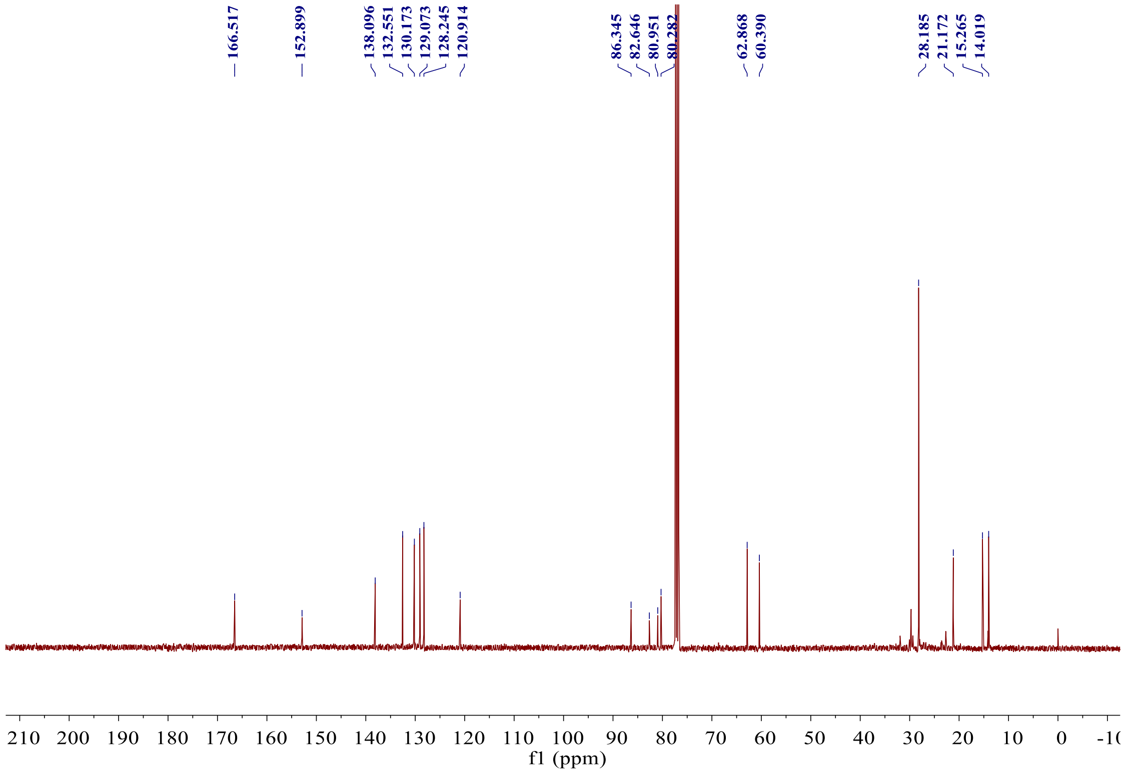

Supplementary Fig. 19. ^{1}H NMR & ^{13}C NMR spectra of compound **3c** in $CDCl_3$

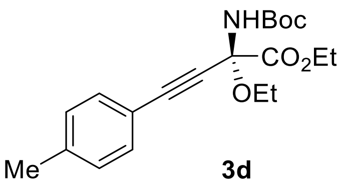

3d

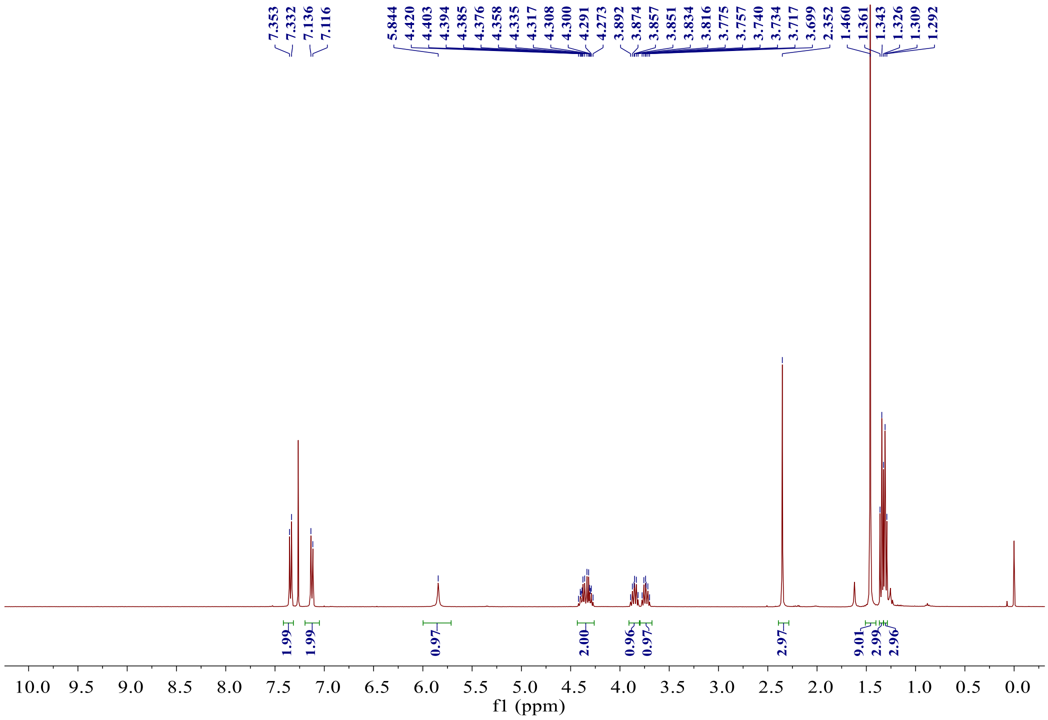

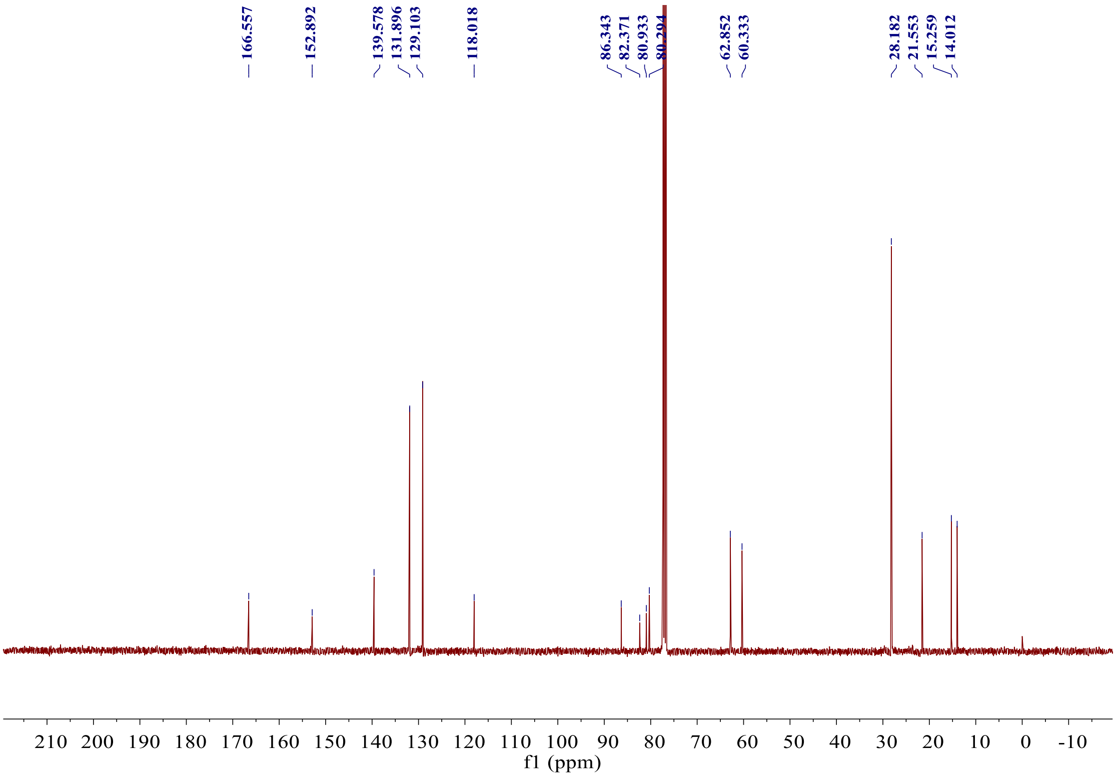

Supplementary Fig. 20. ^{1}H NMR & ^{13}C NMR spectra of compound **3d** in $CDCl_3$

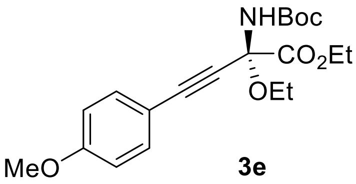

3e

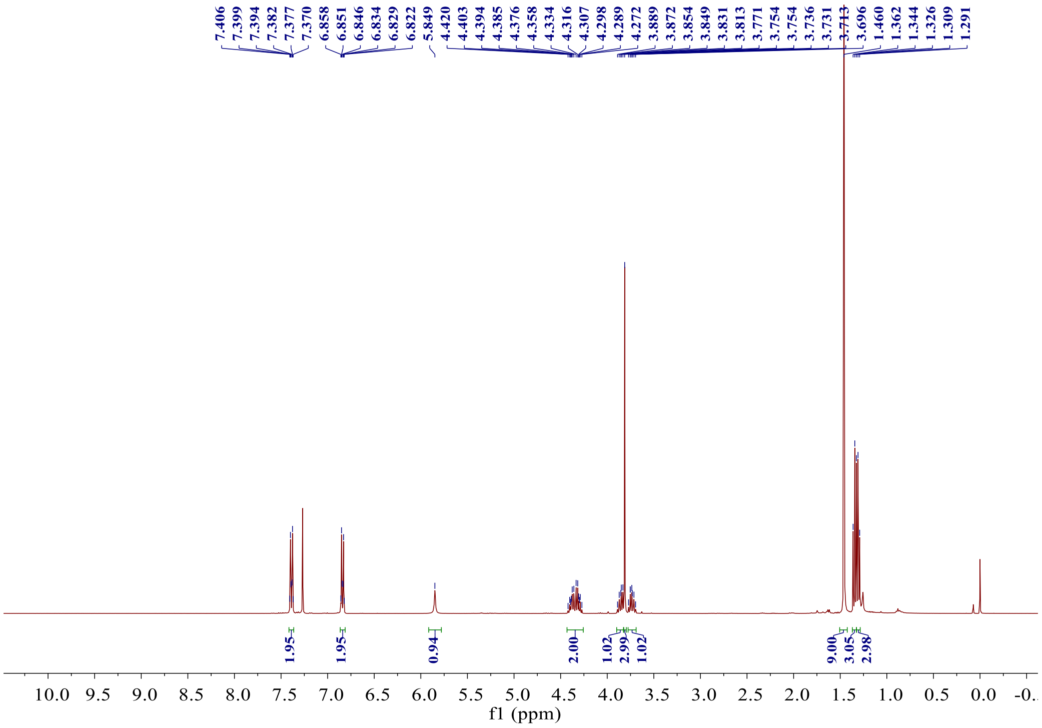

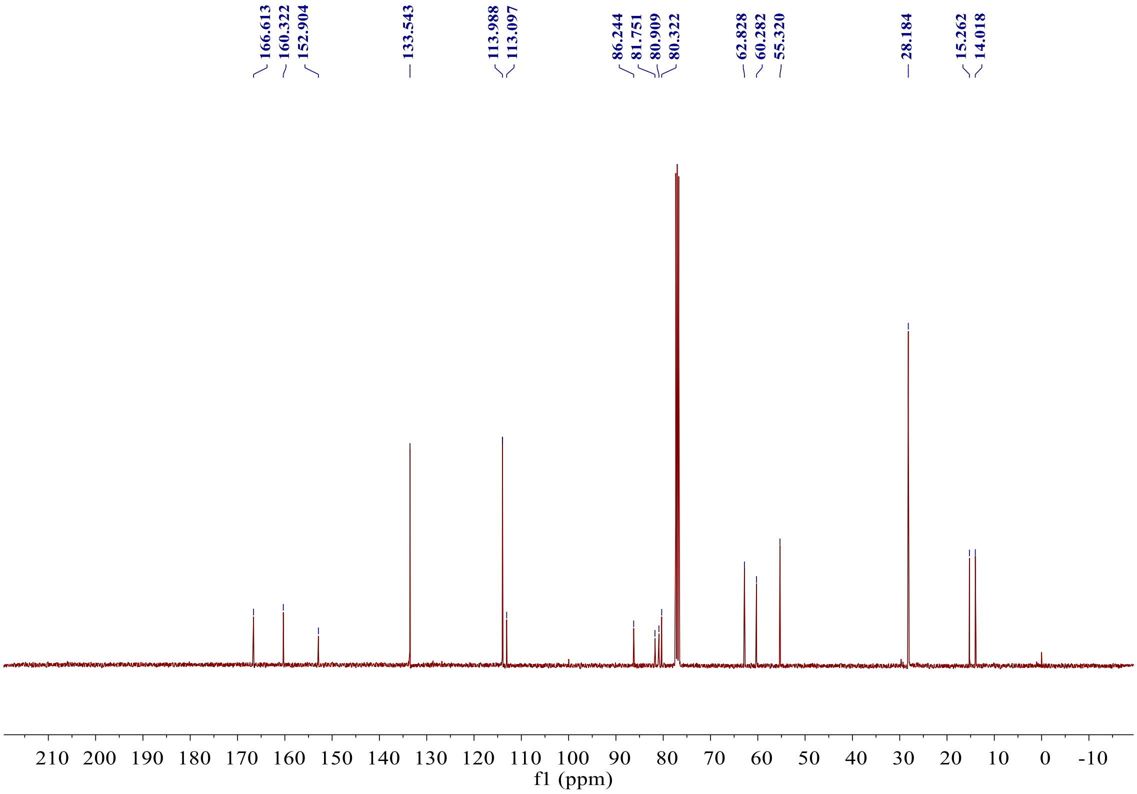

Supplementary Fig. 21. ^{1}H NMR & ^{13}C NMR spectra of compound **3e** in $CDCl_3$

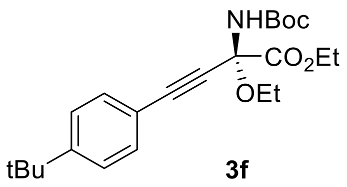

3f

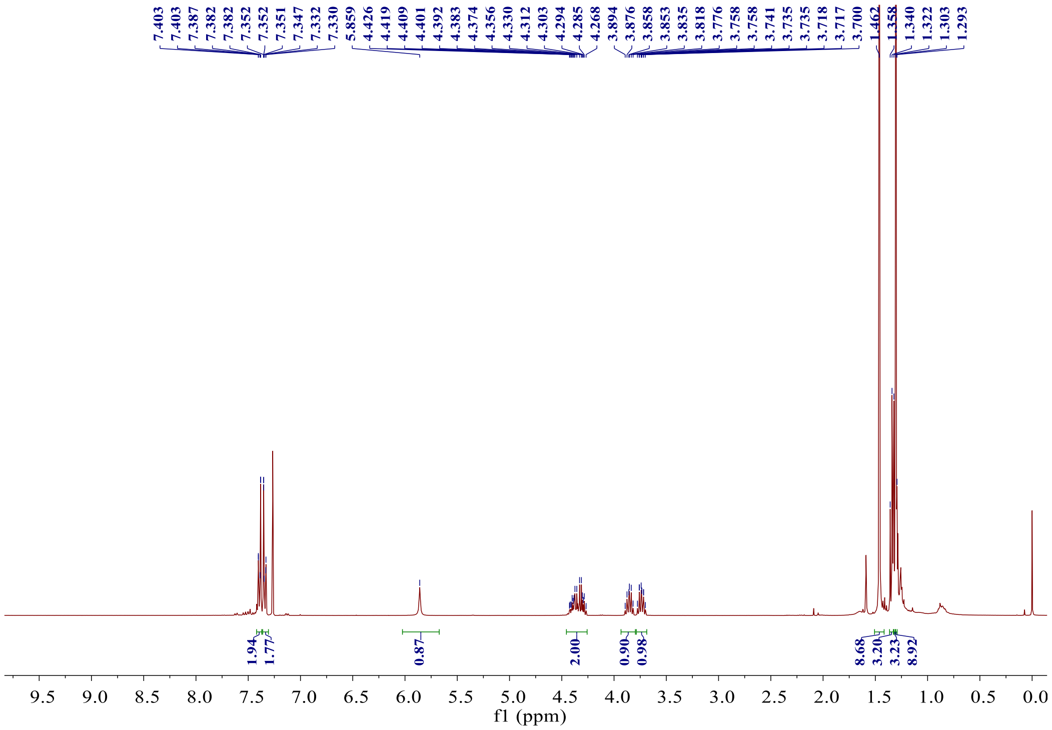

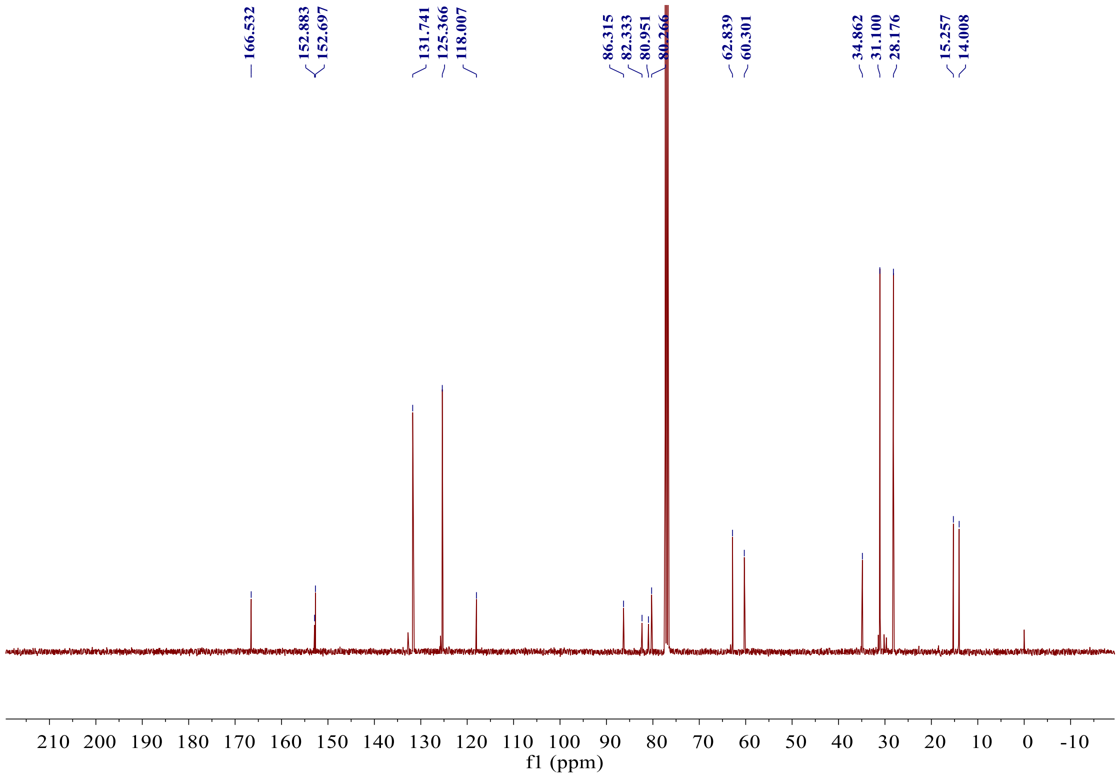

Supplementary Fig. 22. ^{1}H NMR & ^{13}C NMR spectra of compound **3f** in $CDCl_3$

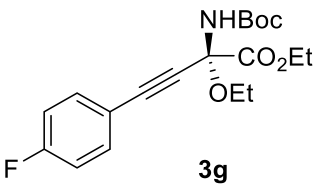
NHBoc
CO2Et
OEt
F

3g

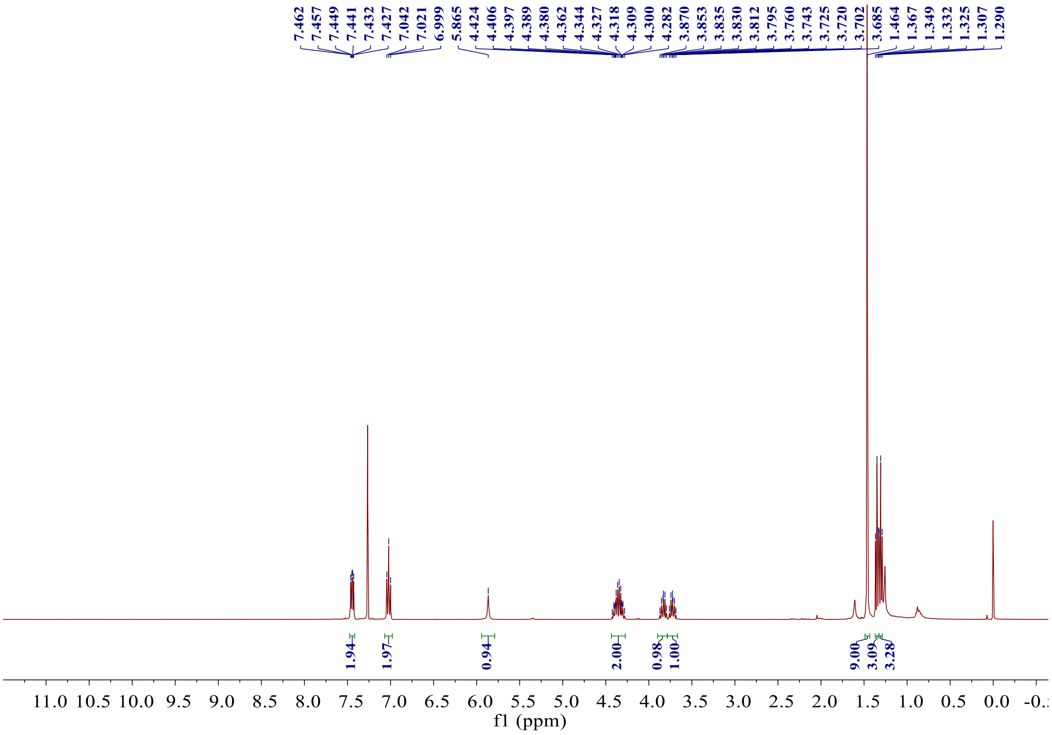
7.462
7.457
7.449
7.441
7.432
7.427
7.042
7.021
6.999
5.865
4.424
4.406
4.397
4.389
4.380
4.362
4.344
4.327
4.318
4.309
4.300
4.282
3.870
3.853
3.835
3.830
3.812
3.795
3.760
3.743
3.725
3.720
3.702
3.685
1.464
1.367
1.349
1.332
1.325
1.307
1.290
1.94
1.97
0.94
2.00
0.98
1.00
9.00
3.09
3.28
11.0 10.5 10.0 9.5 9.0 8.5 8.0 7.5 7.0 6.5 6.0 5.5 5.0 4.5 4.0 3.5 3.0 2.5 2.0 1.5 1.0 0.5 0.0
f1 (ppm)

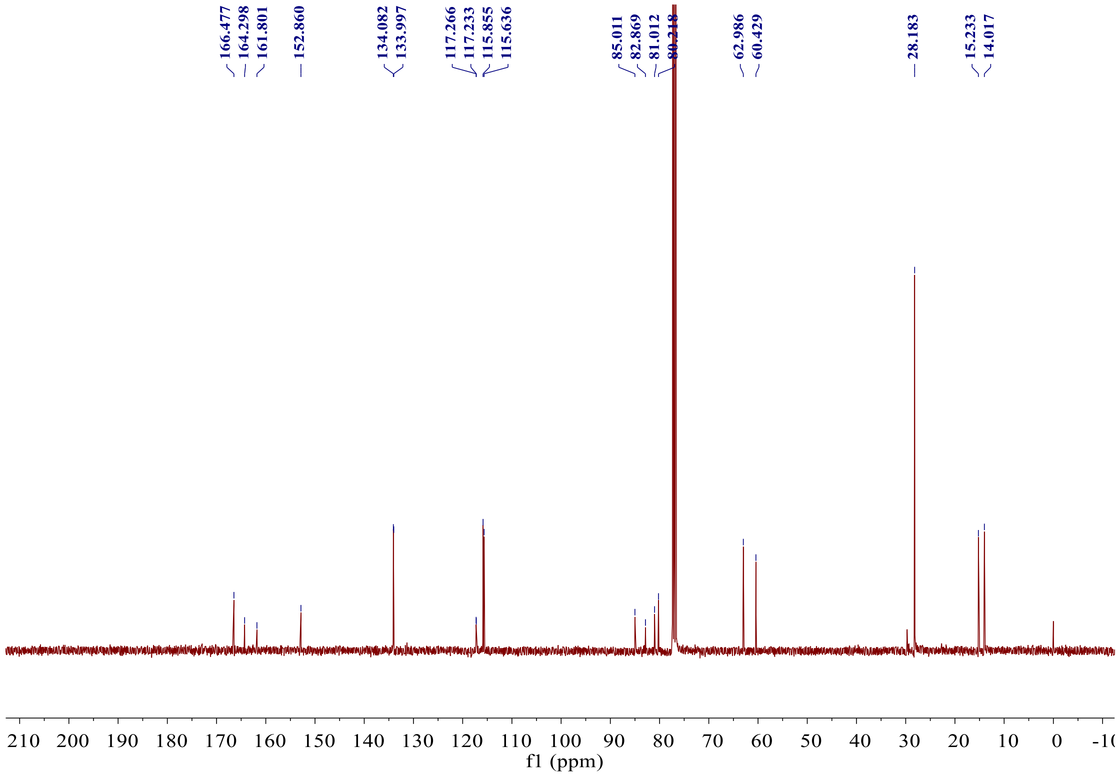
166.477
164.298
161.801
152.860
134.082
133.997
117.266
117.233
115.855
115.636
85.011
82.869
81.012
62.986
60.429
28.183
15.233
14.017
210 200 190 180 170 160 150 140 130 120 110 100 90 80 70 60 50 40 30 20 10 0
f1 (ppm)

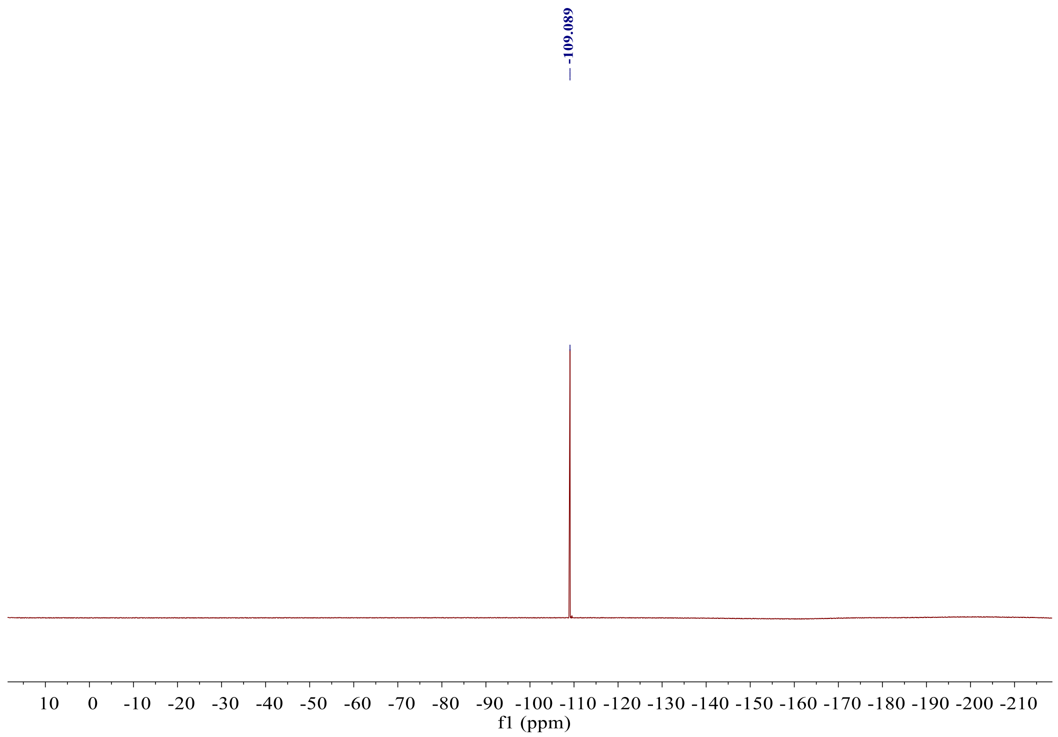

Supplementary Fig. 23. ^{1}H NMR, ^{13}C NMR & ^{19}F NMR spectra of compound **3g** in $CDCl_3$

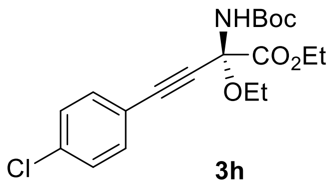

3h

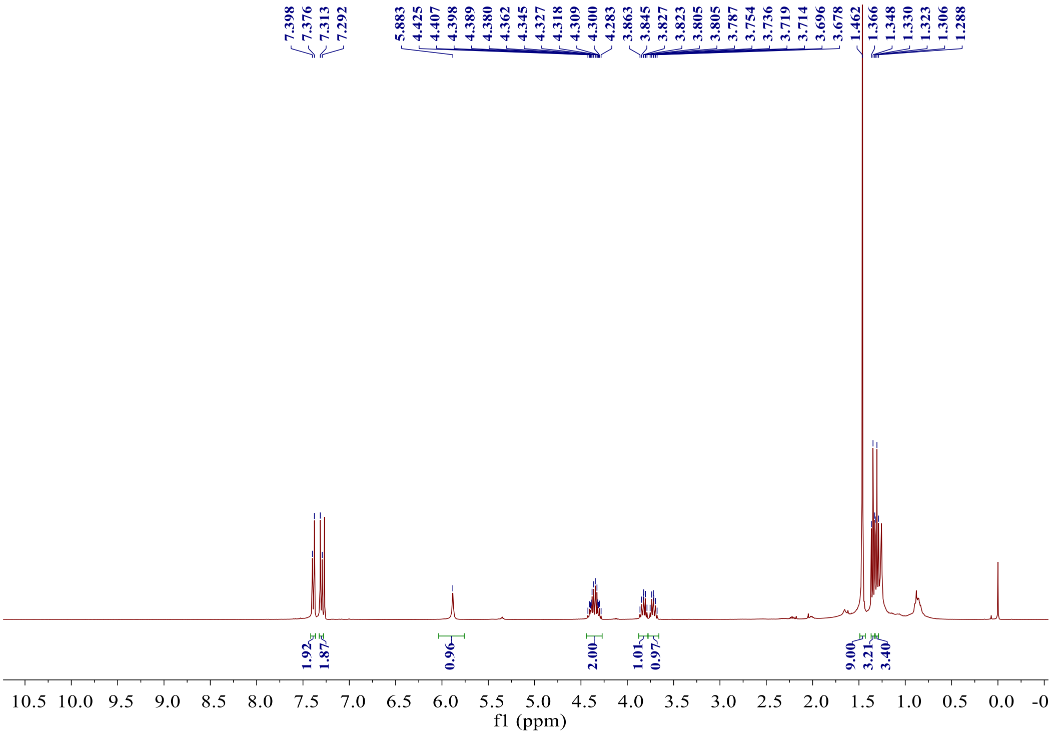

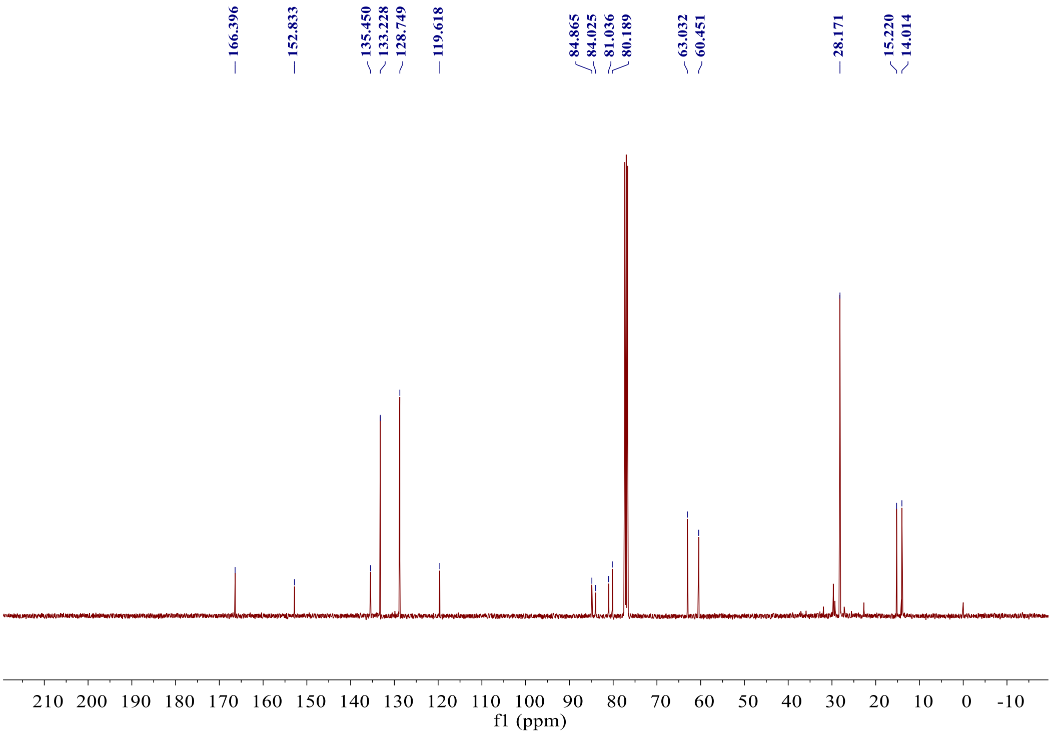

Supplementary Fig. 24. ^{1}H NMR & ^{13}C NMR spectra of compound **3h** in $CDCl_3$

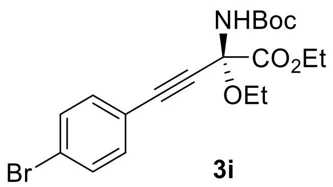

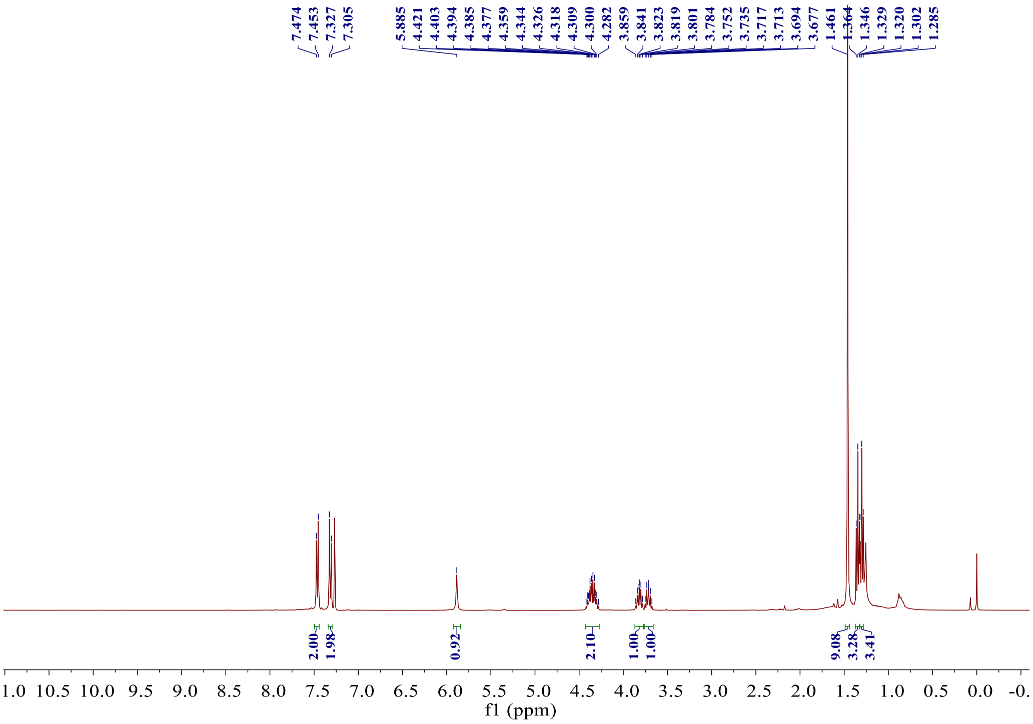

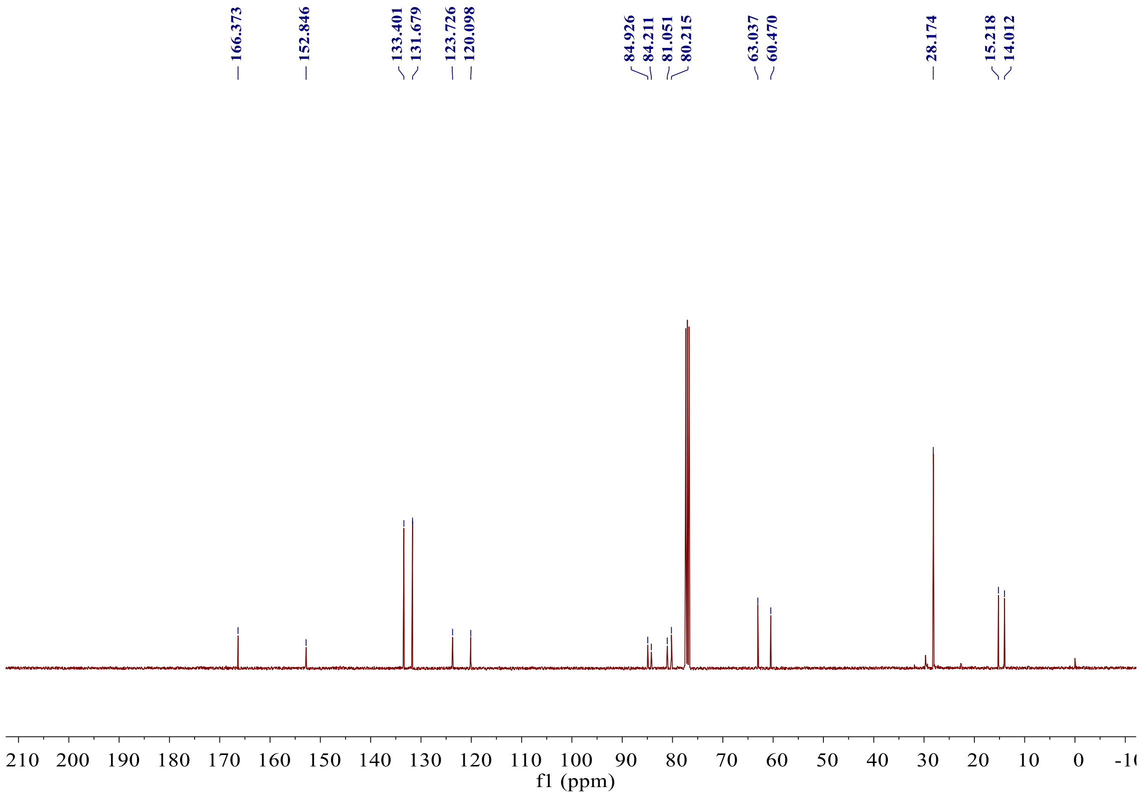

Supplementary Fig. 25. ^{1}H NMR & ^{13}C NMR spectra of compound **3i** in $CDCl_3$

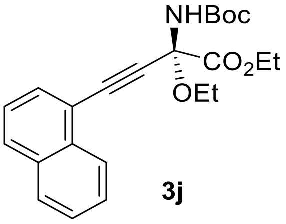

3j

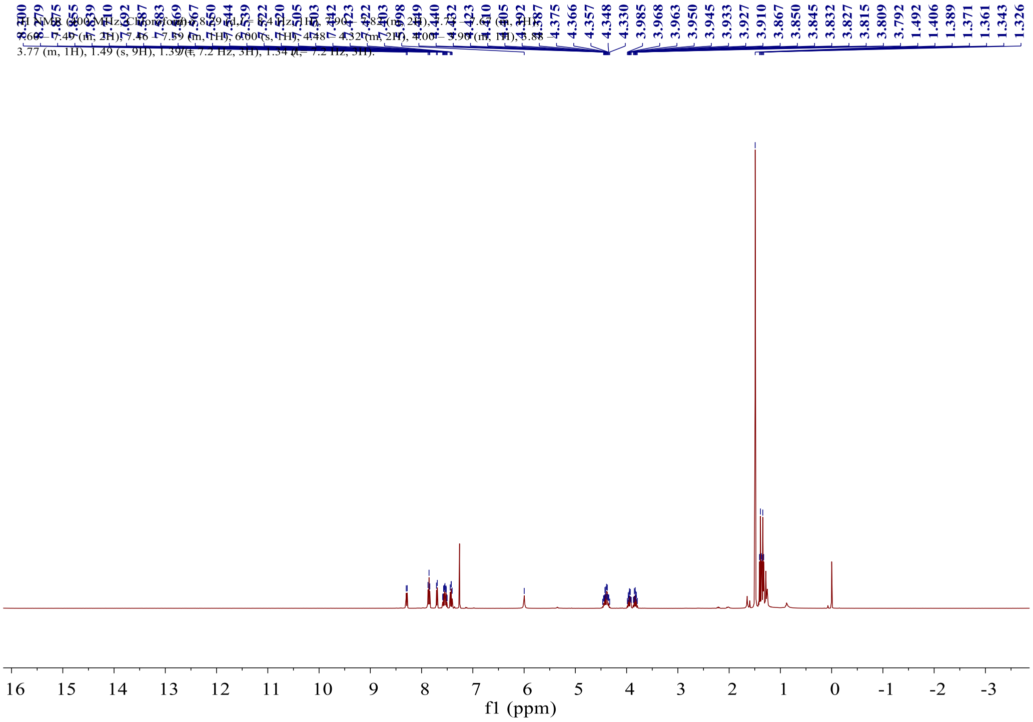

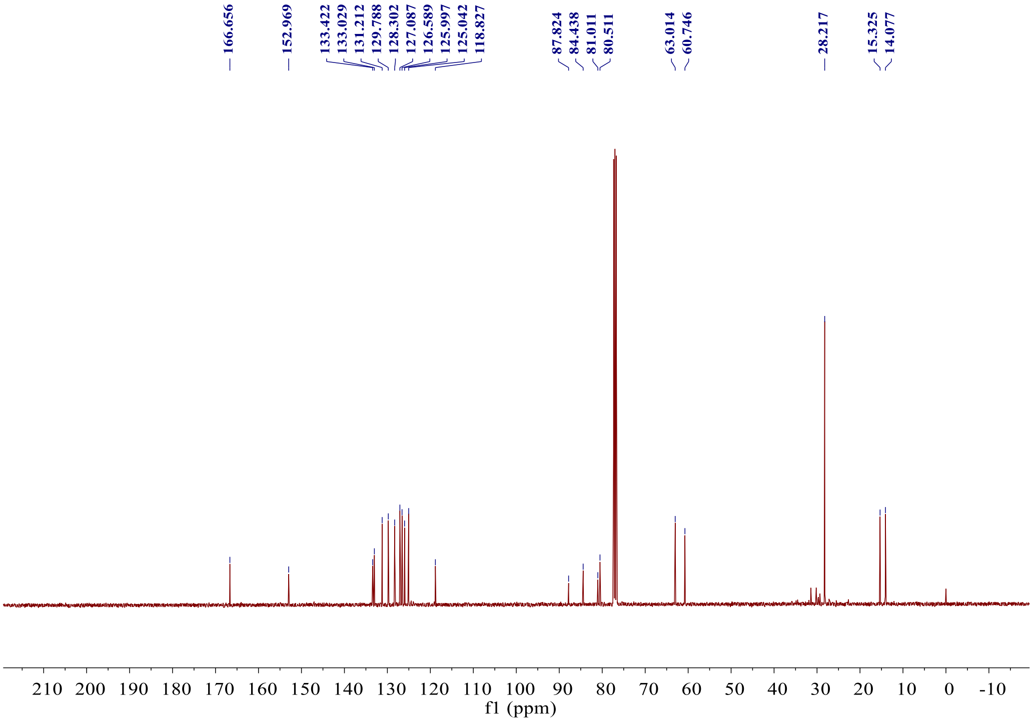

Supplementary Fig. 26. ^{1}H NMR & ^{13}C NMR spectra of compound **3j** in $CDCl_3$

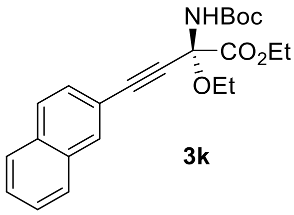

3k

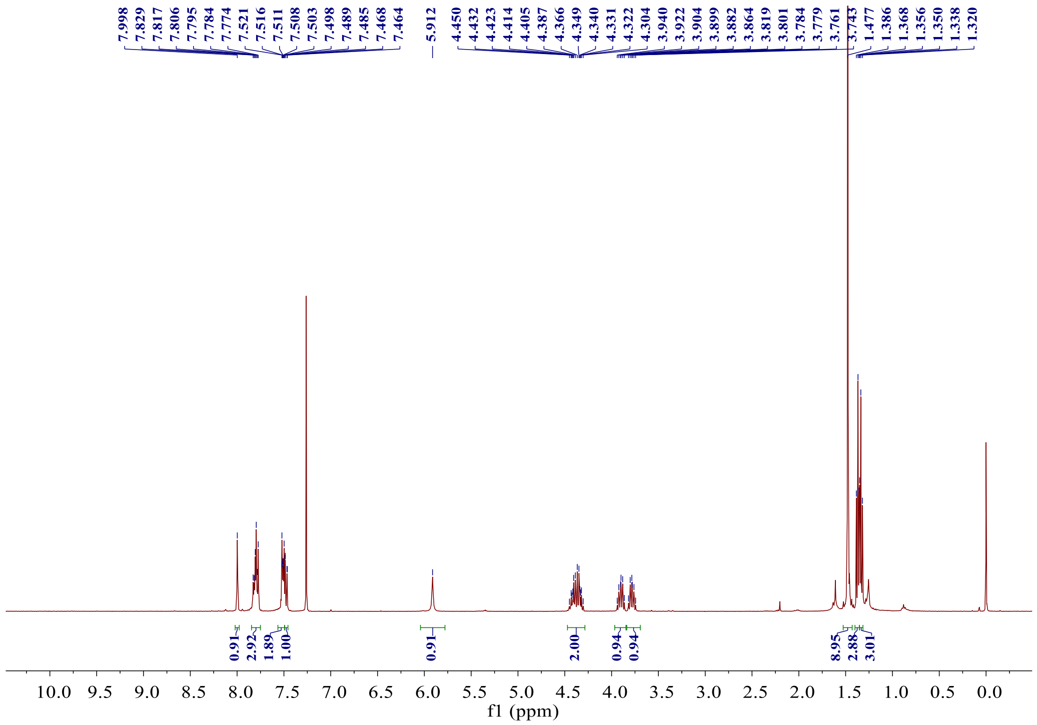

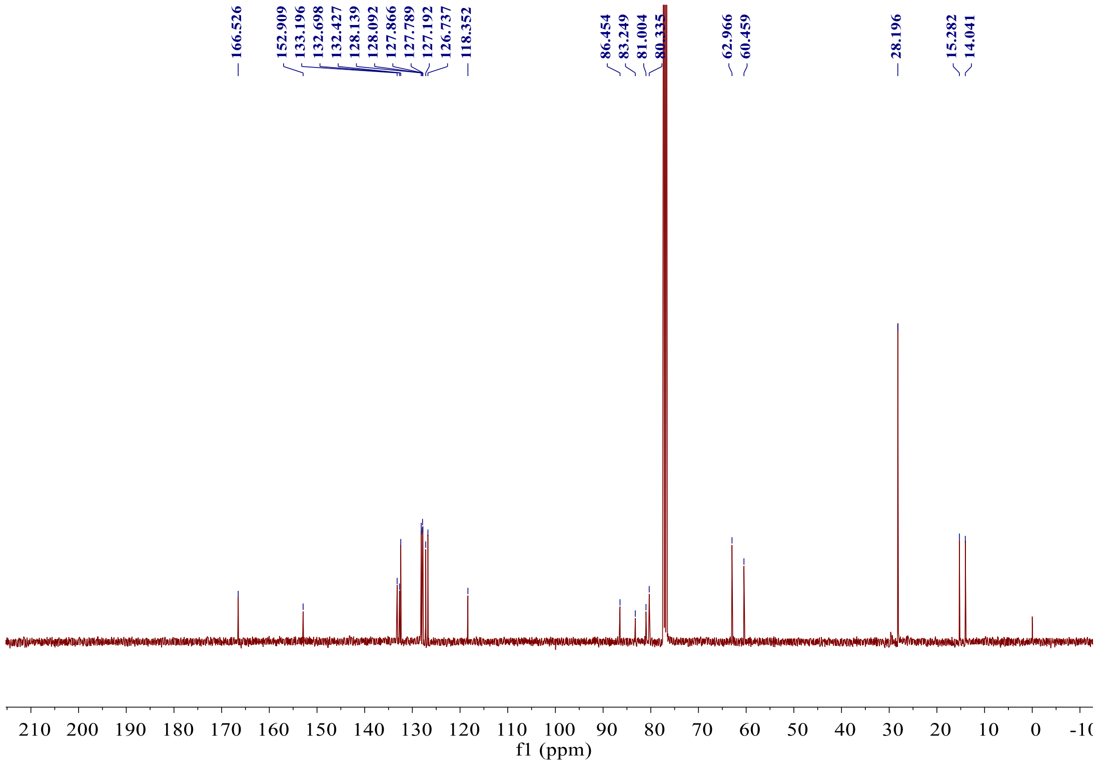

Supplementary Fig. 27. ^{1}H NMR & ^{13}C NMR spectra of compound **3k** in $CDCl_3$

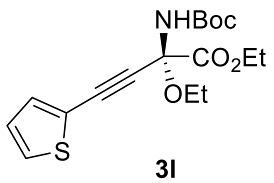

3l

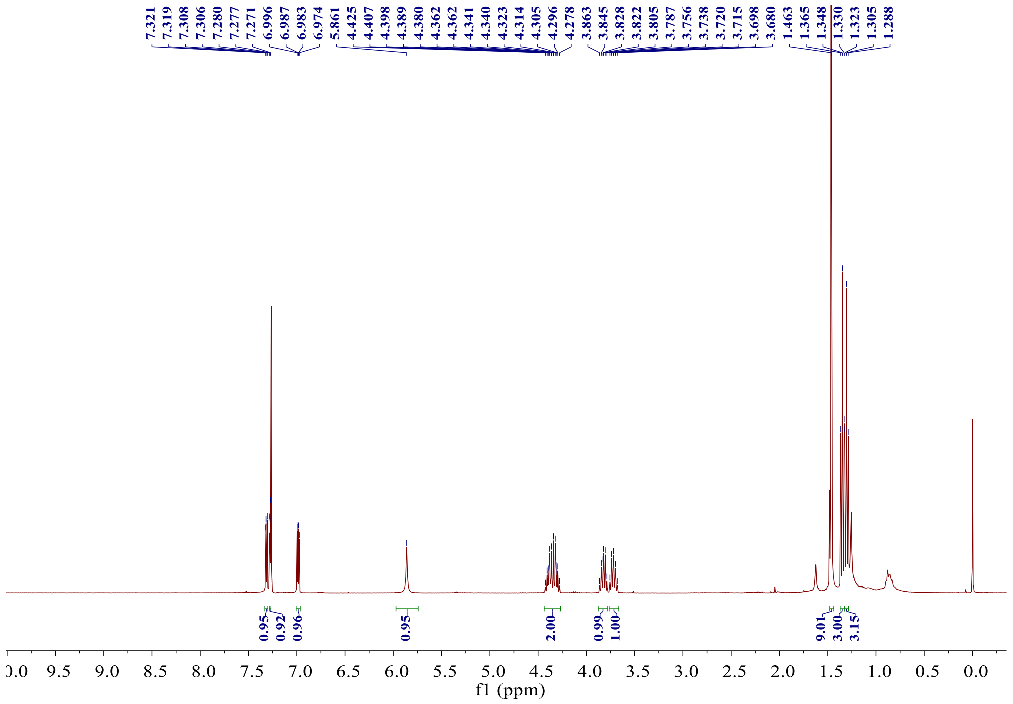

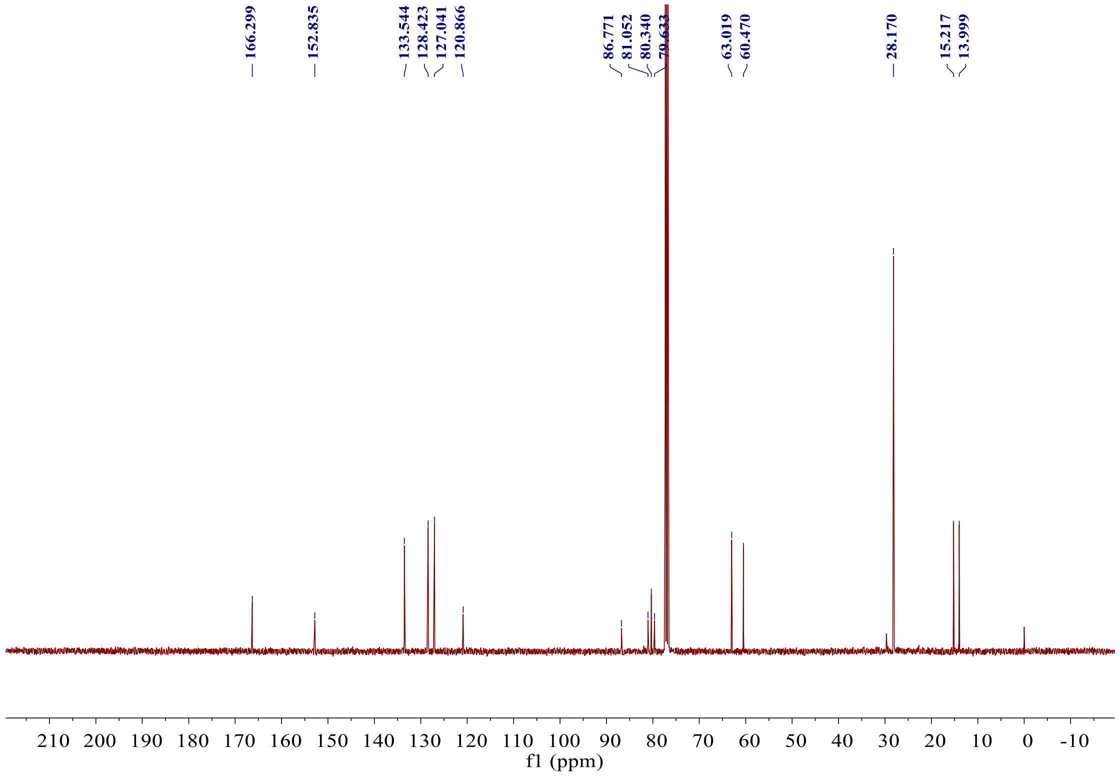

Supplementary Fig. 28. ^{1}H NMR & ^{13}C NMR spectra of compound **3l** in $CDCl_3$

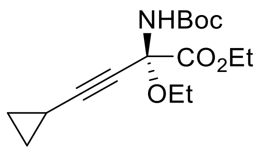

3m

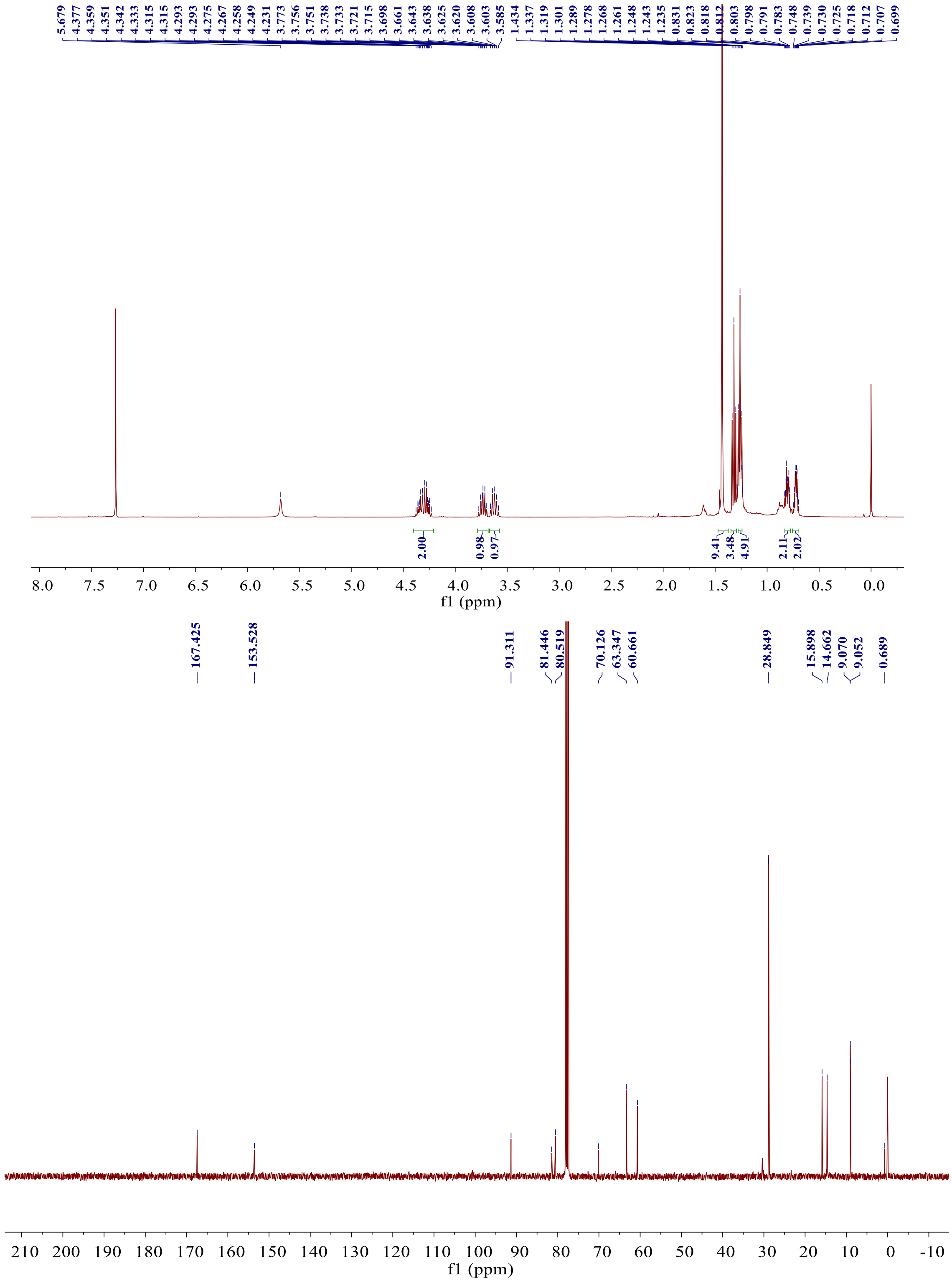

Supplementary Fig. 29. ^{1}H NMR & ^{13}C NMR spectra of compound **3m** in $CDCl_3$

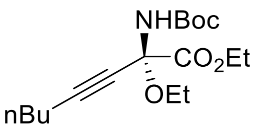

3n

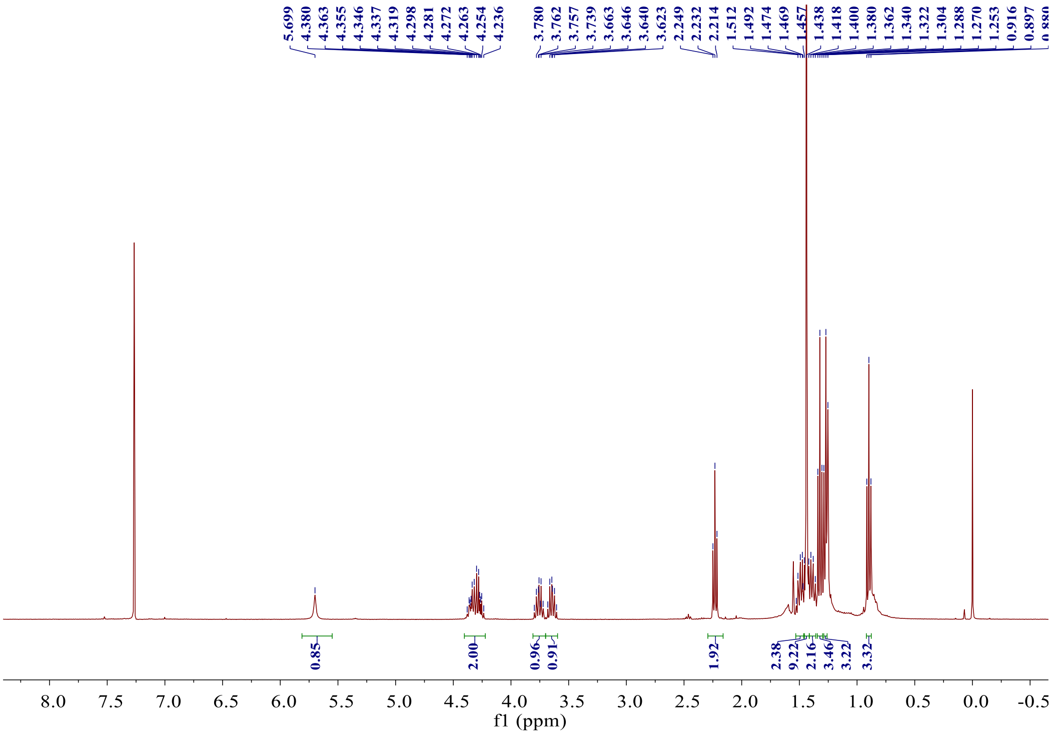

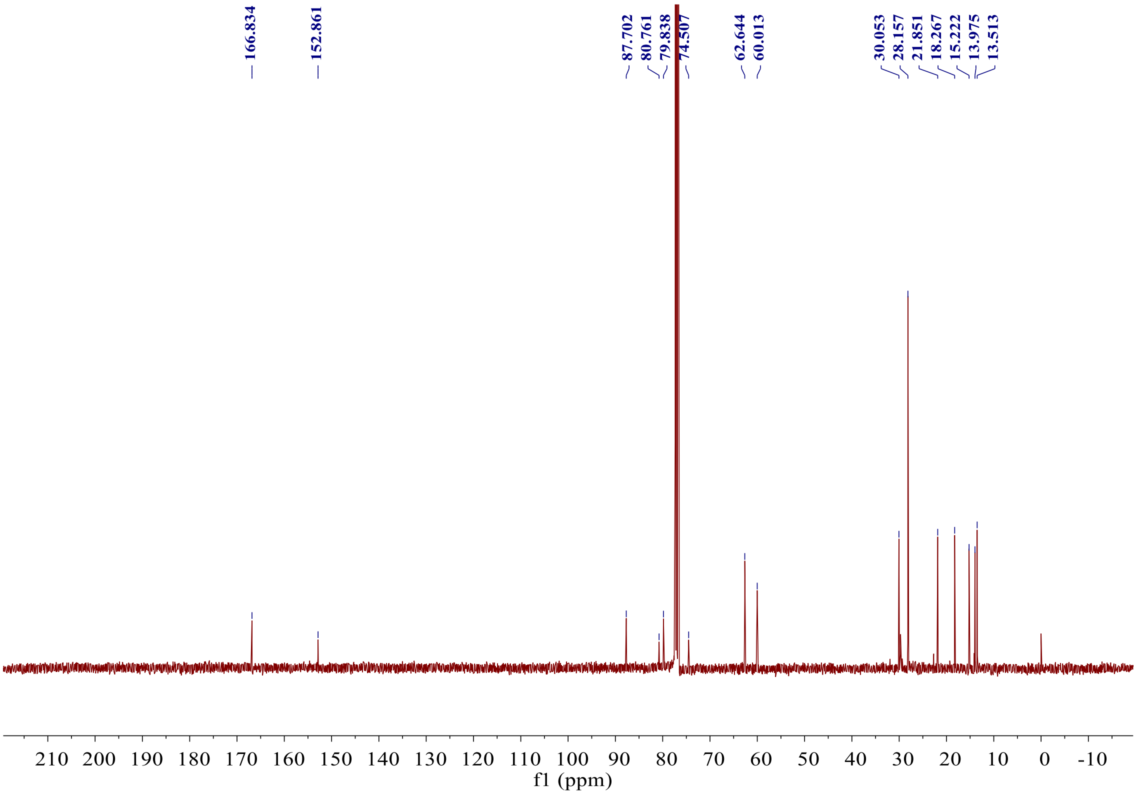

Supplementary Fig. 30. ^{1}H NMR & ^{13}C NMR spectra of compound **3n** in $CDCl_3$

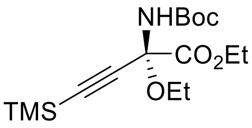

3o

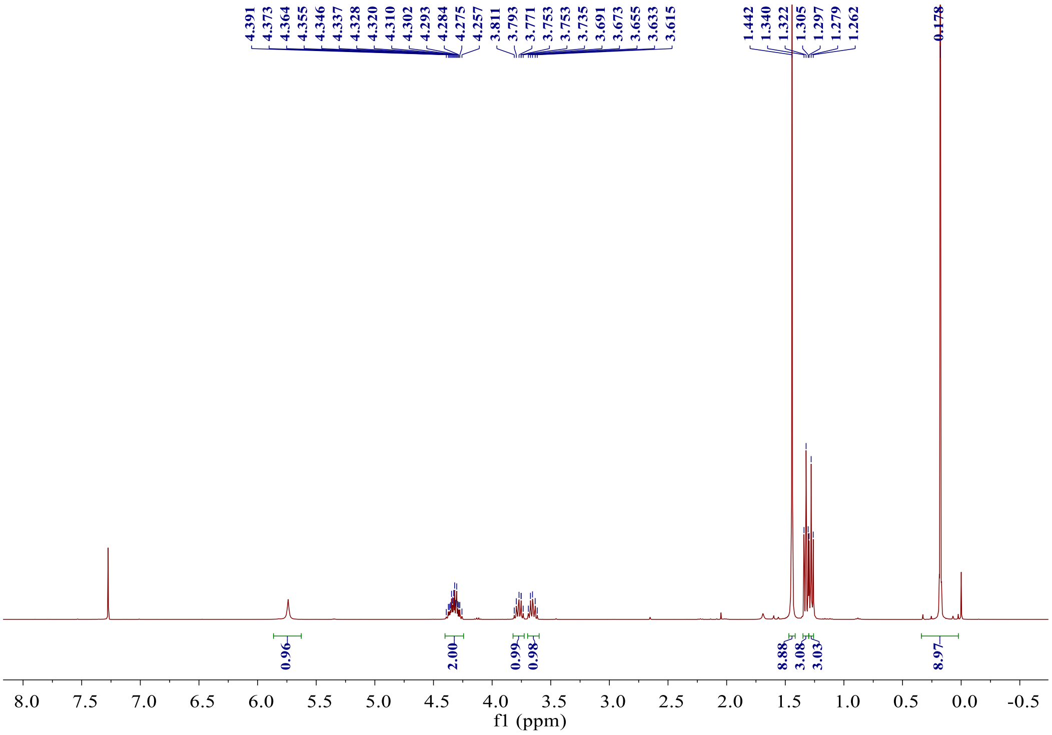

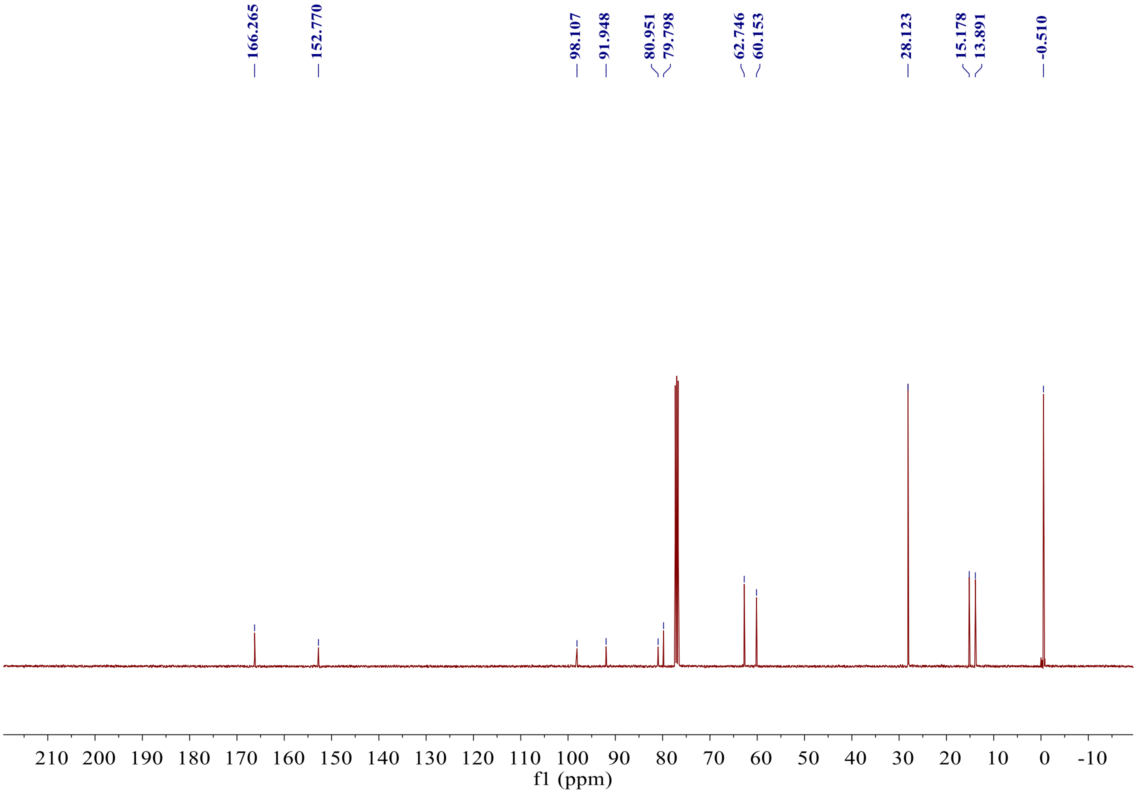

Supplementary Fig. 31. ^{1}H NMR & ^{13}C NMR spectra of compound **3o** in $CDCl_3$

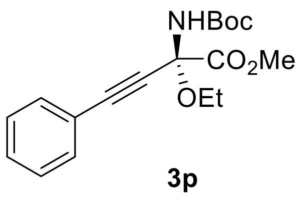

3p

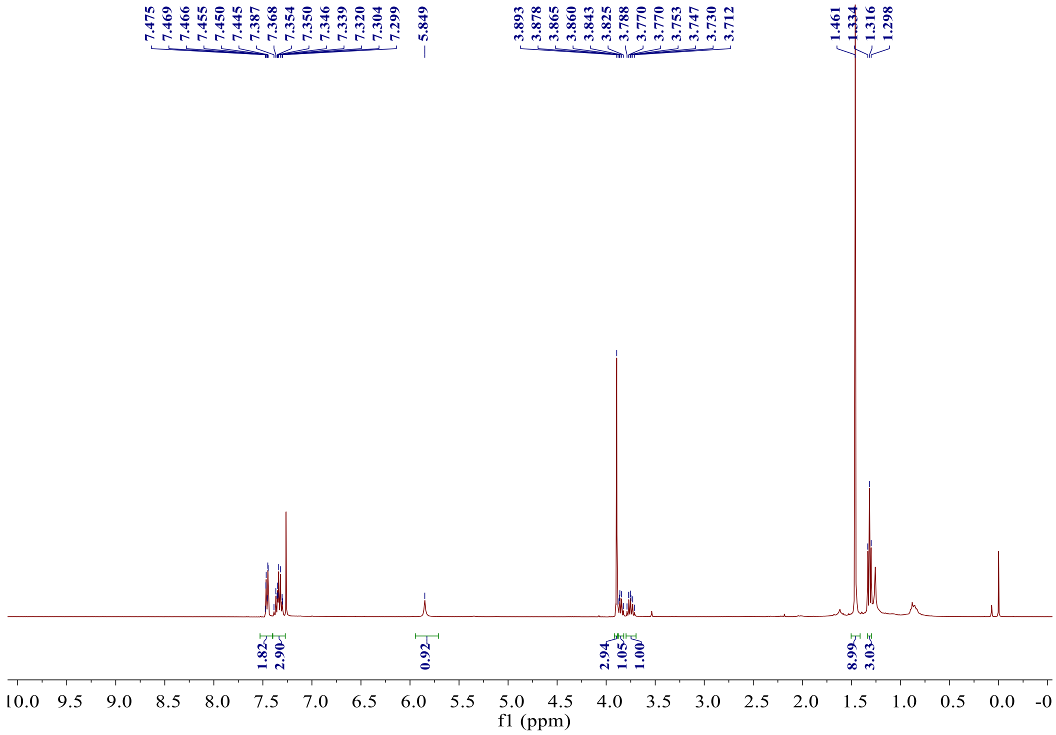

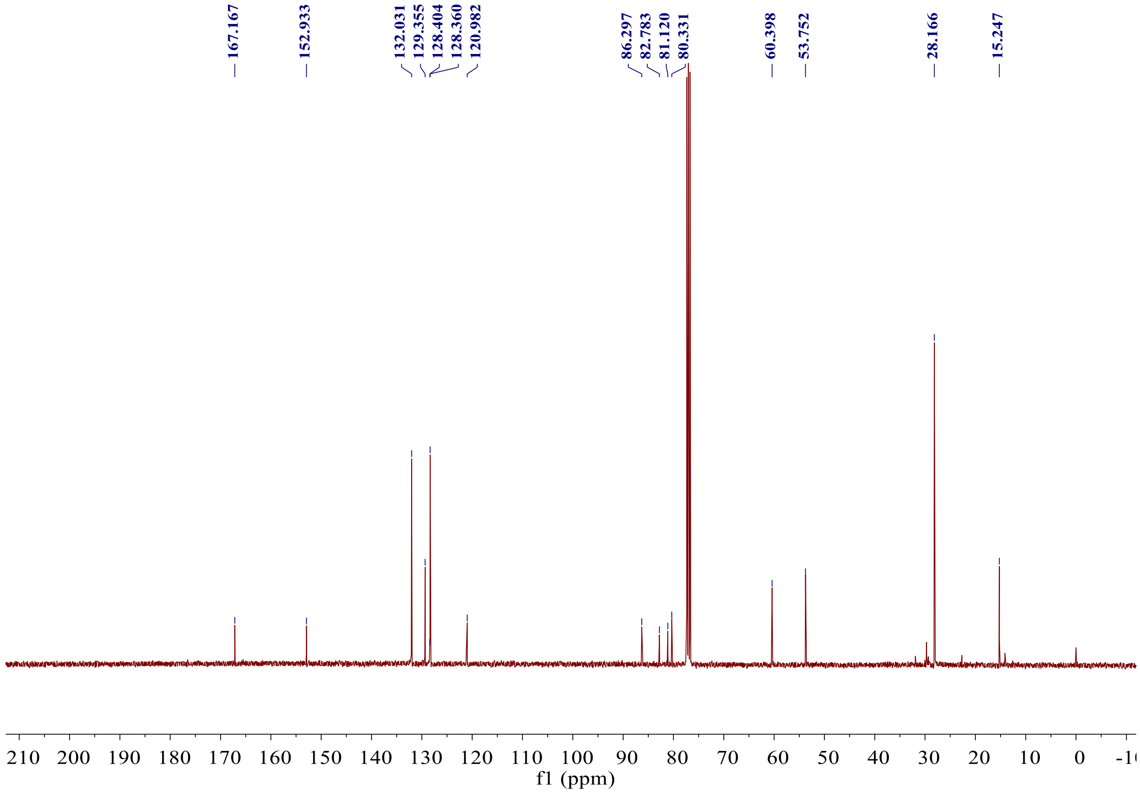

Supplementary Fig. 32. ^{1}H NMR & ^{13}C NMR spectra of compound **3p** in $CDCl_3$

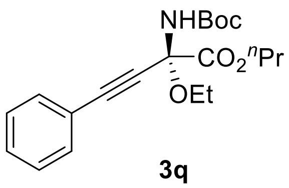

3q

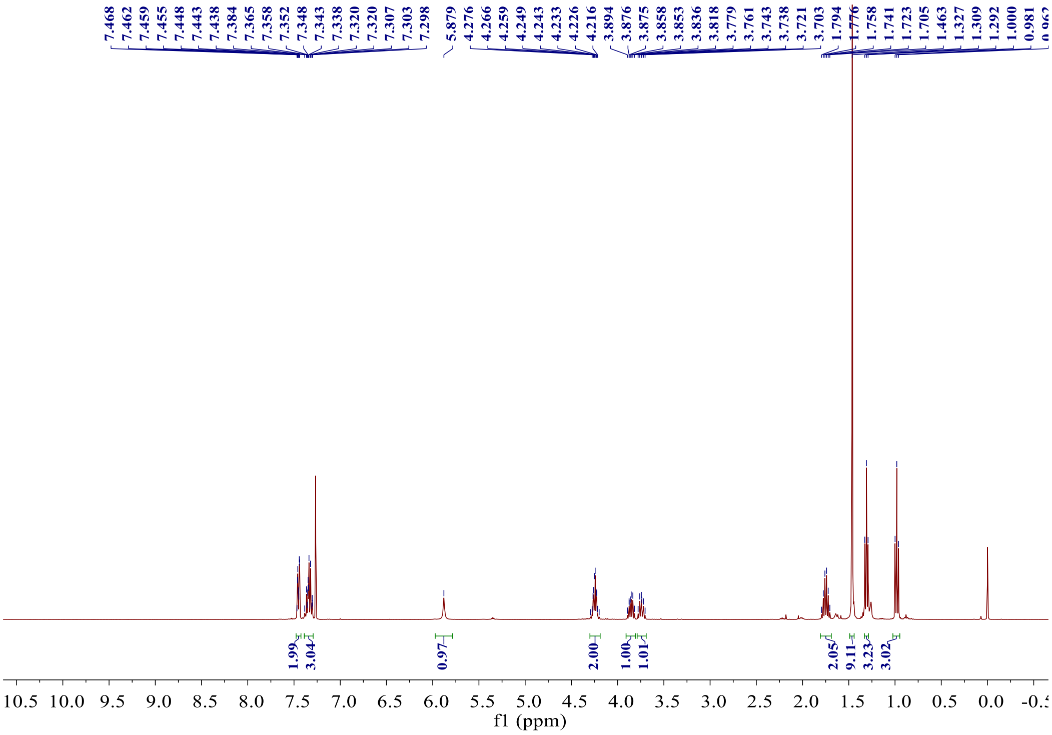

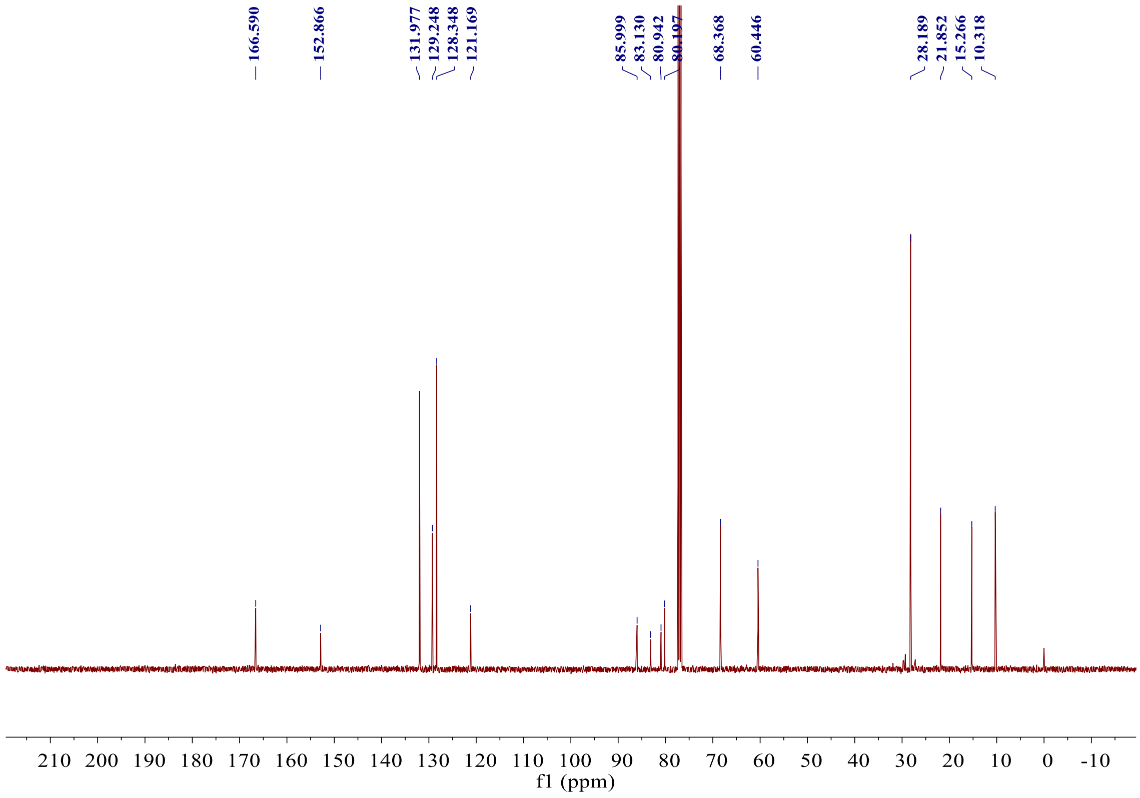

Supplementary Fig. 33. ^{1}H NMR & ^{13}C NMR spectra of compound **3q** in $CDCl_3$

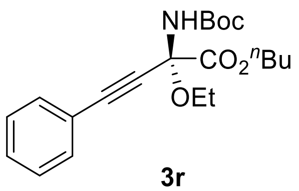

3r

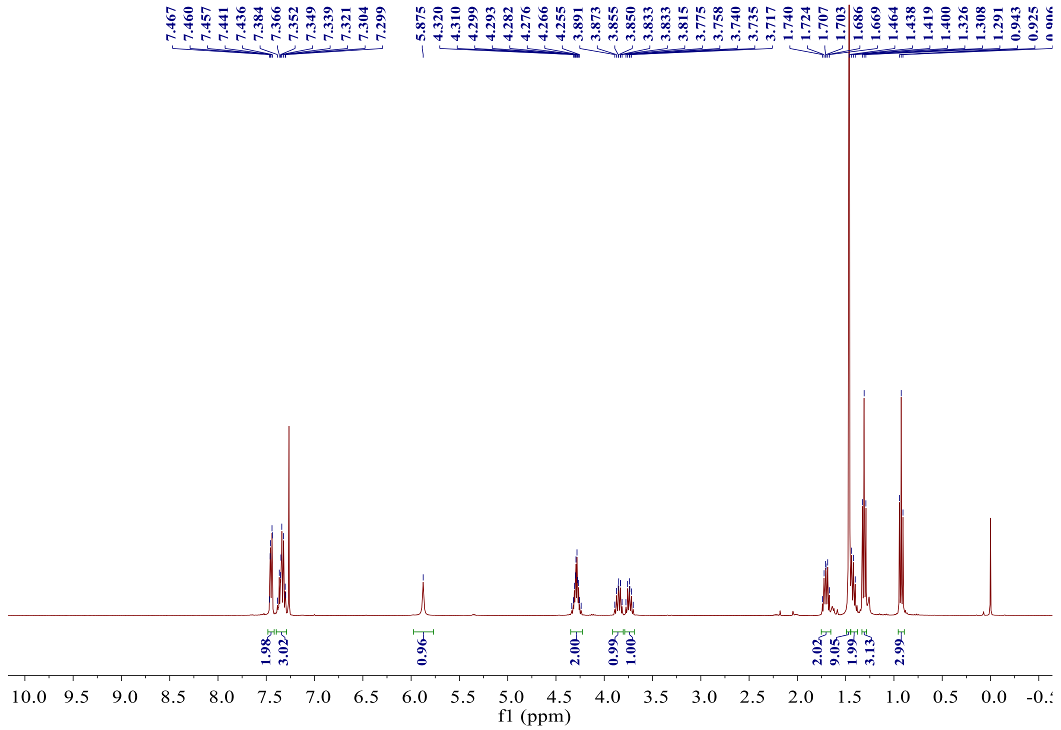

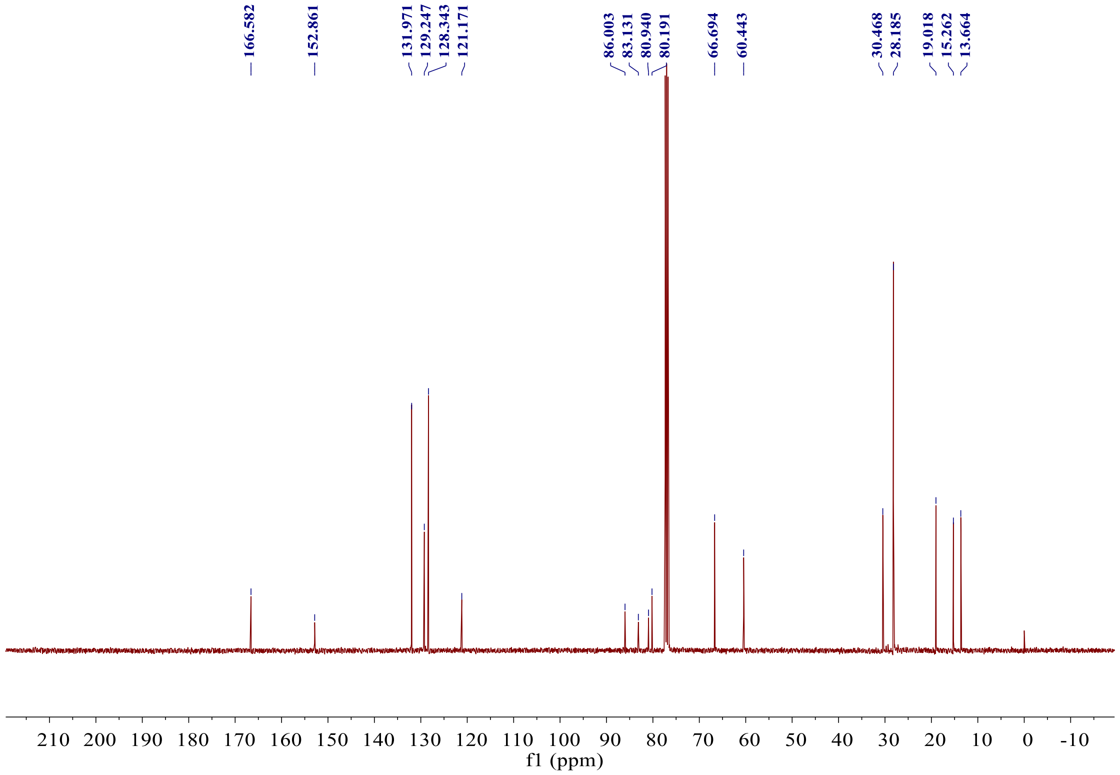

Supplementary Fig. 34. ^{1}H NMR & ^{13}C NMR spectra of compound **3r** in $CDCl_3$

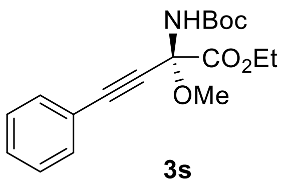

3s

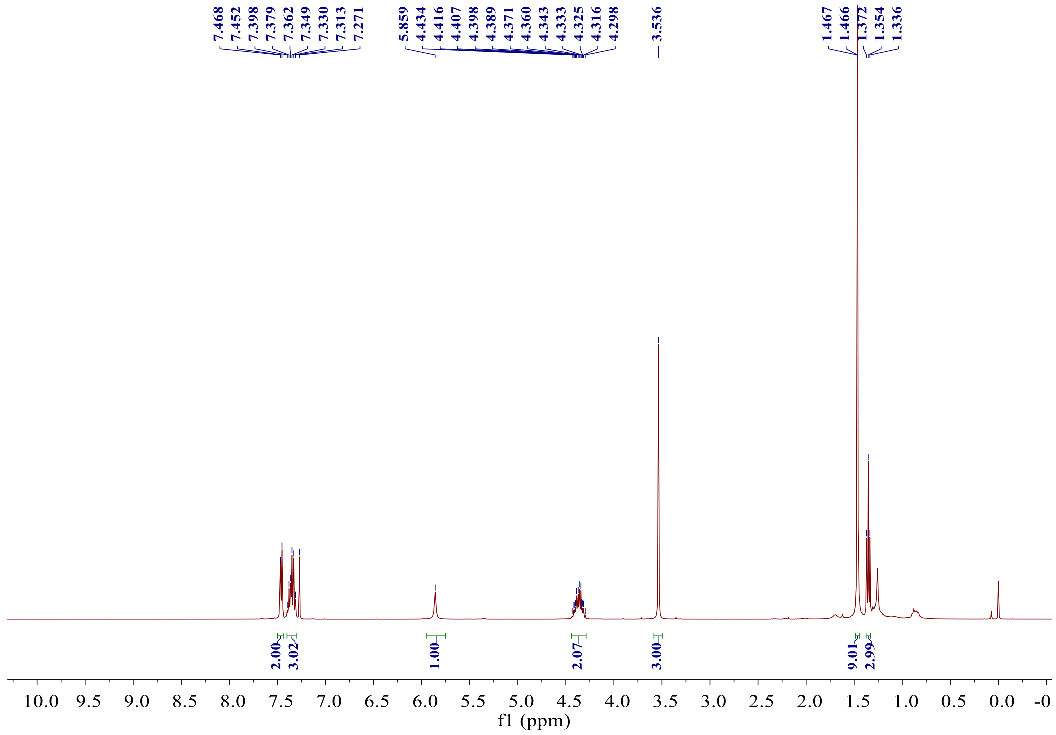

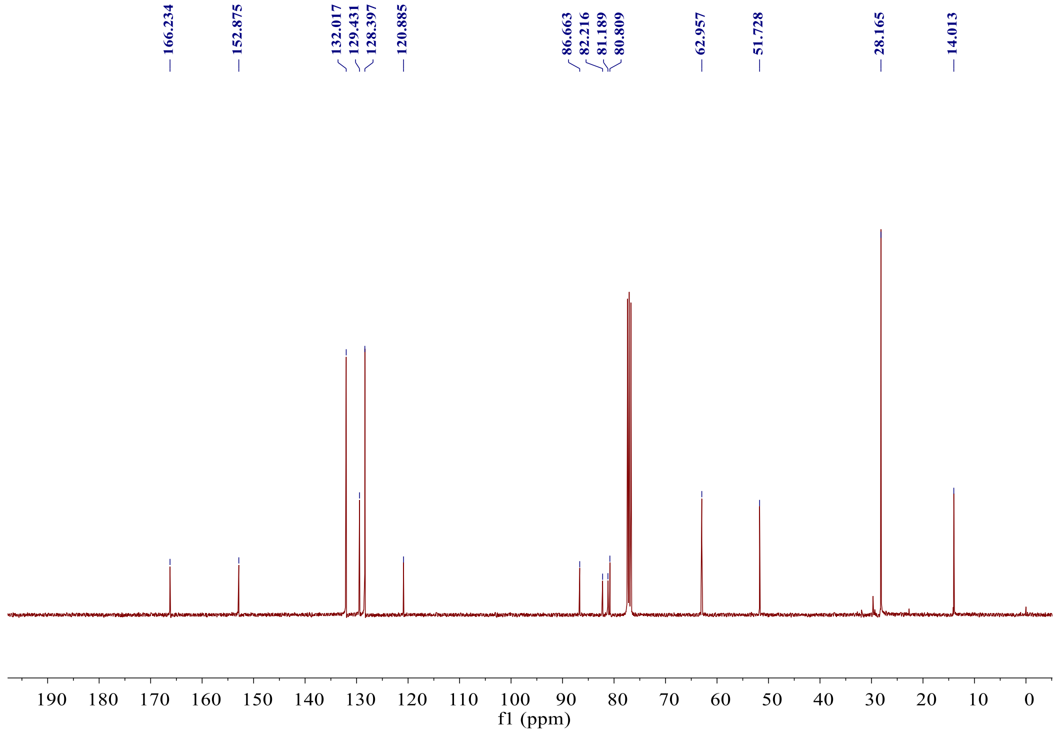

Supplementary Fig. 35. ^{1}H NMR & ^{13}C NMR spectra of compound **3s** in $CDCl_3$

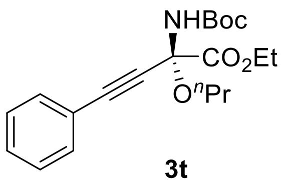

3t

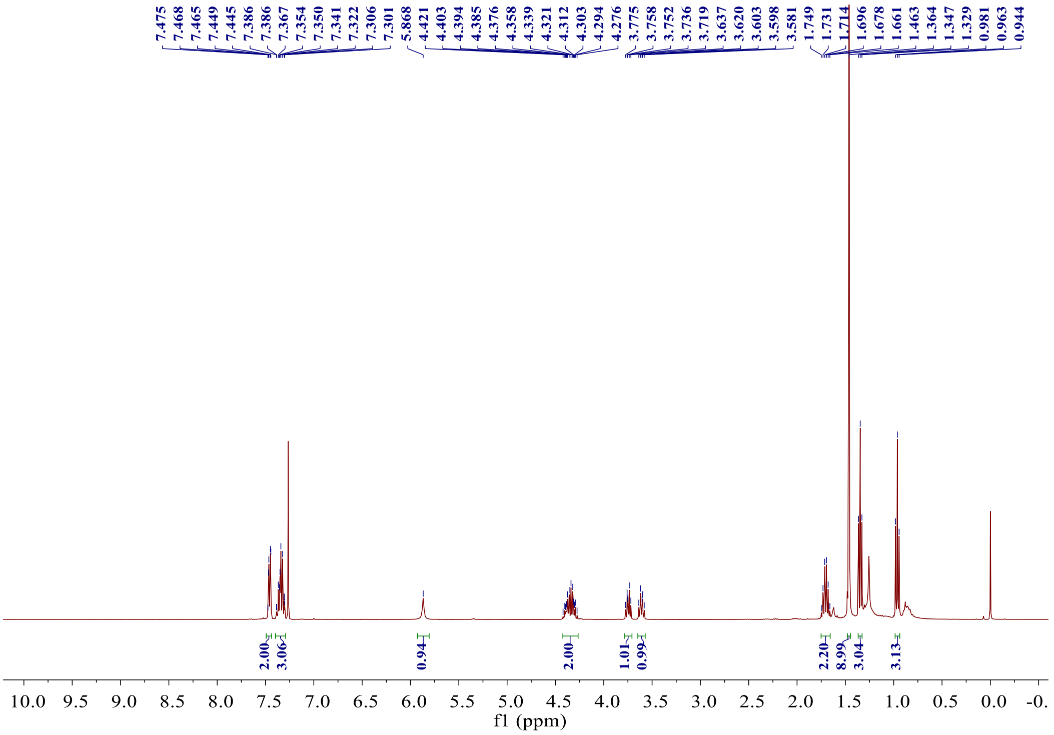

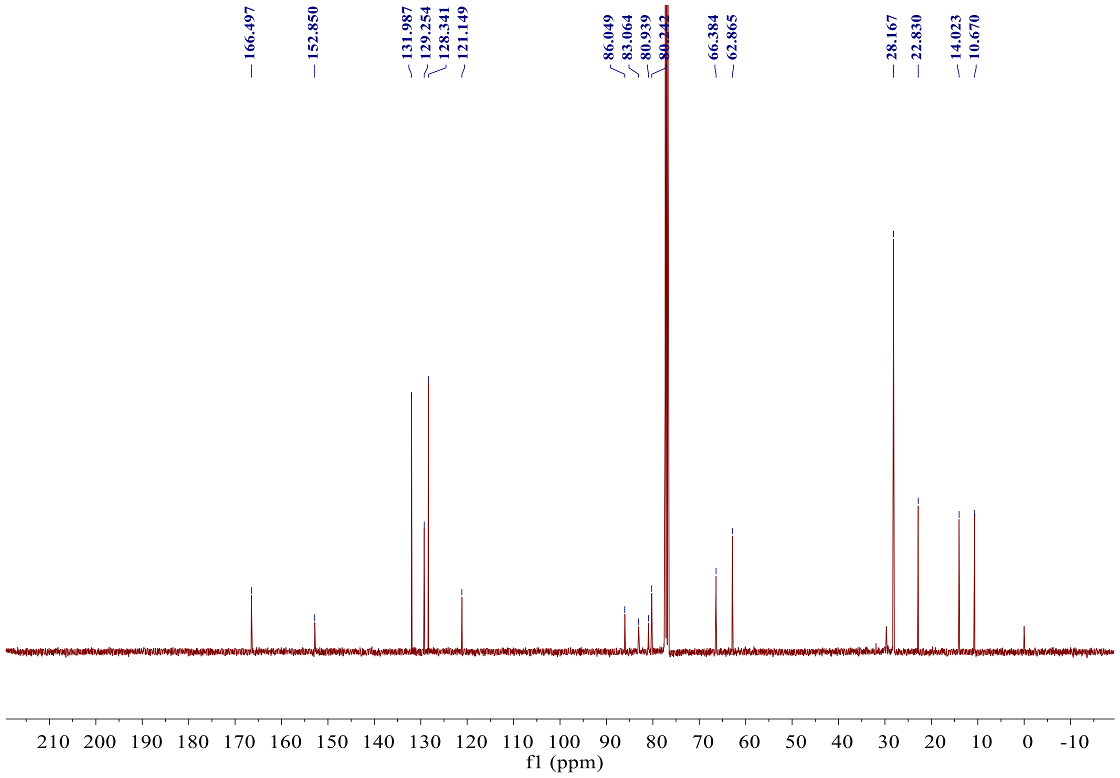

Supplementary Fig. 36. ^{1}H NMR & ^{13}C NMR spectra of compound **3t** in $CDCl_3$

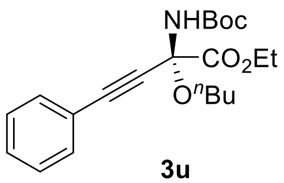

3u

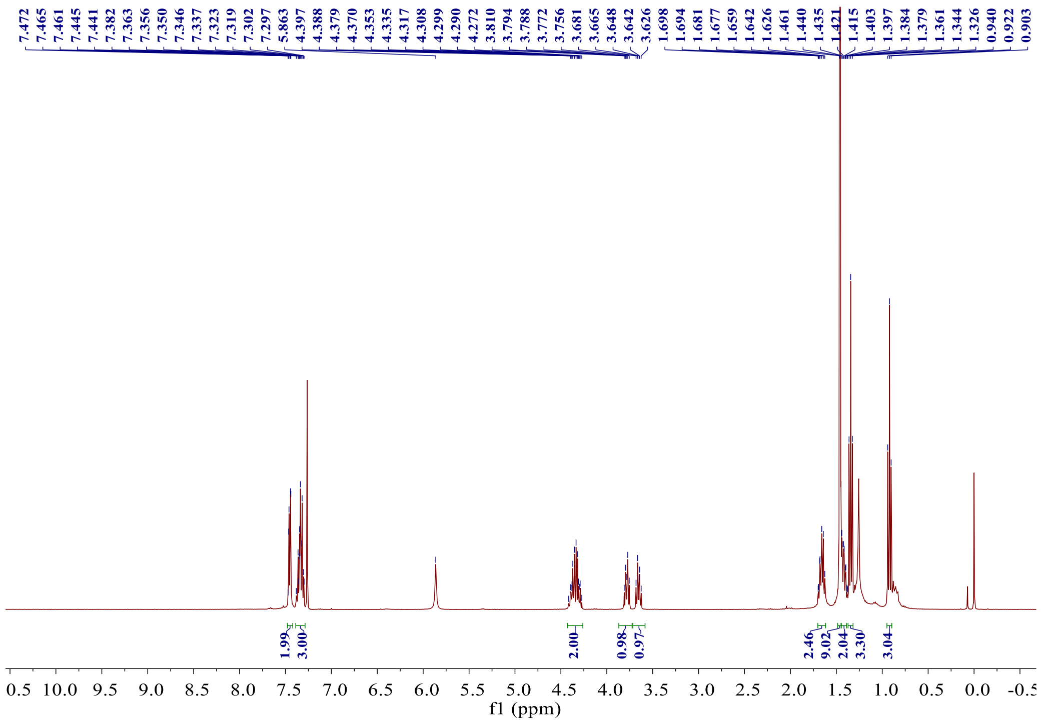

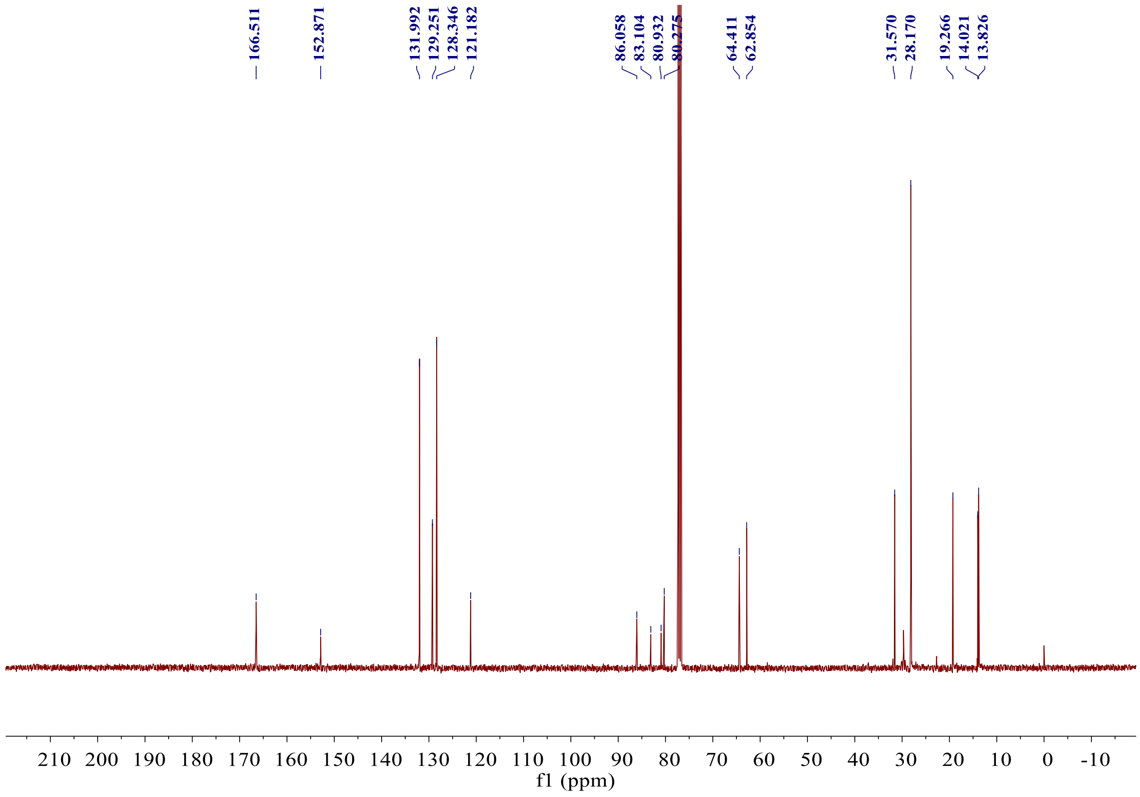

Supplementary Fig. 37. ^{1}H NMR & ^{13}C NMR spectra of compound **3u** in $CDCl_3$

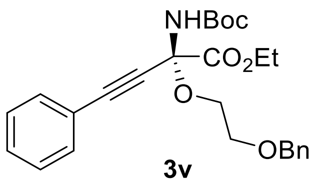

3v

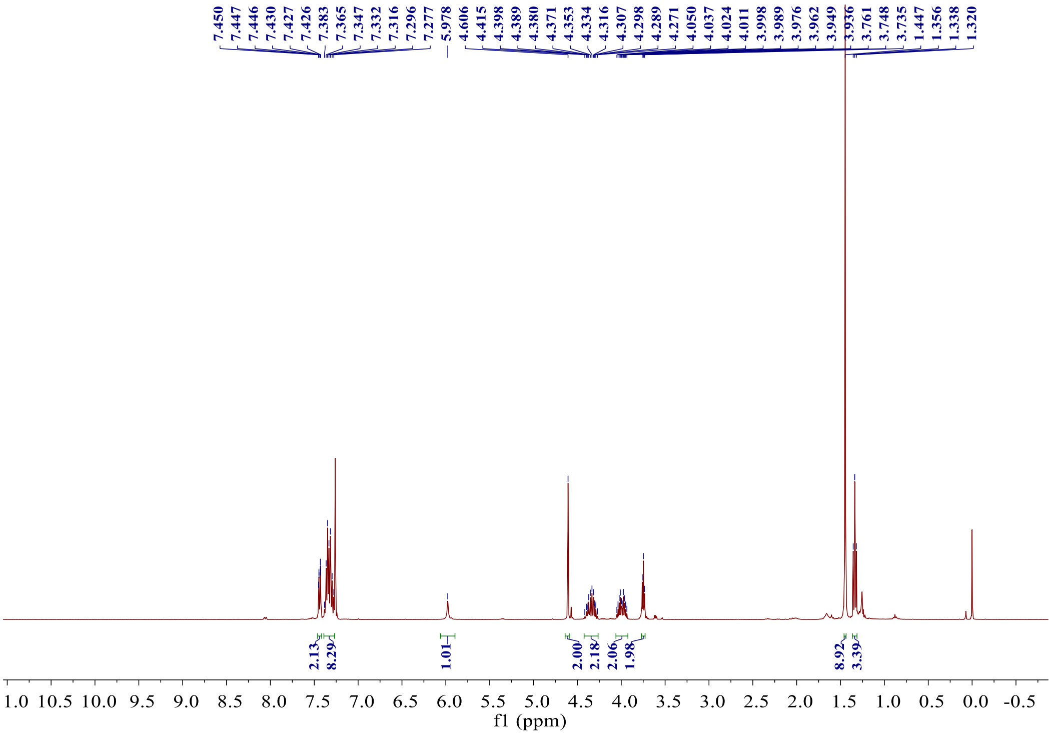

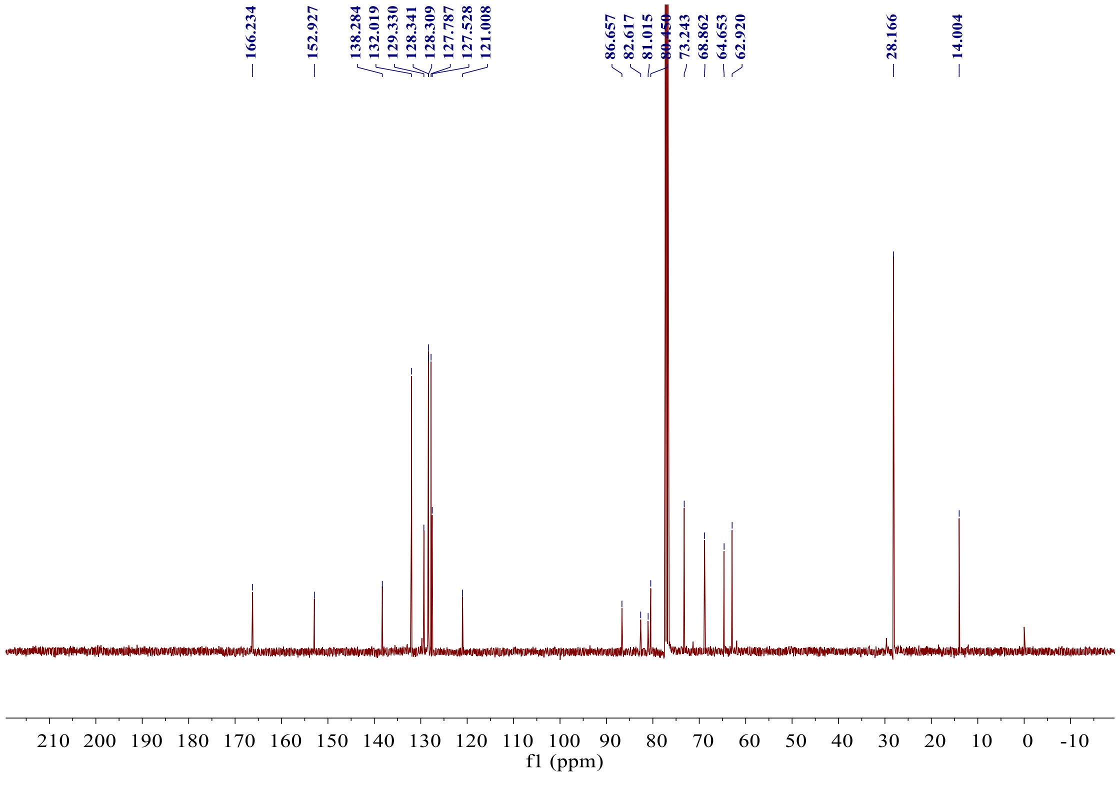

Supplementary Fig. 38. ^{1}H NMR & ^{13}C NMR spectra of compound **3v** in $CDCl_3$

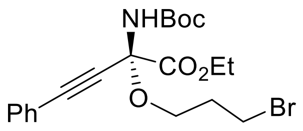

3w

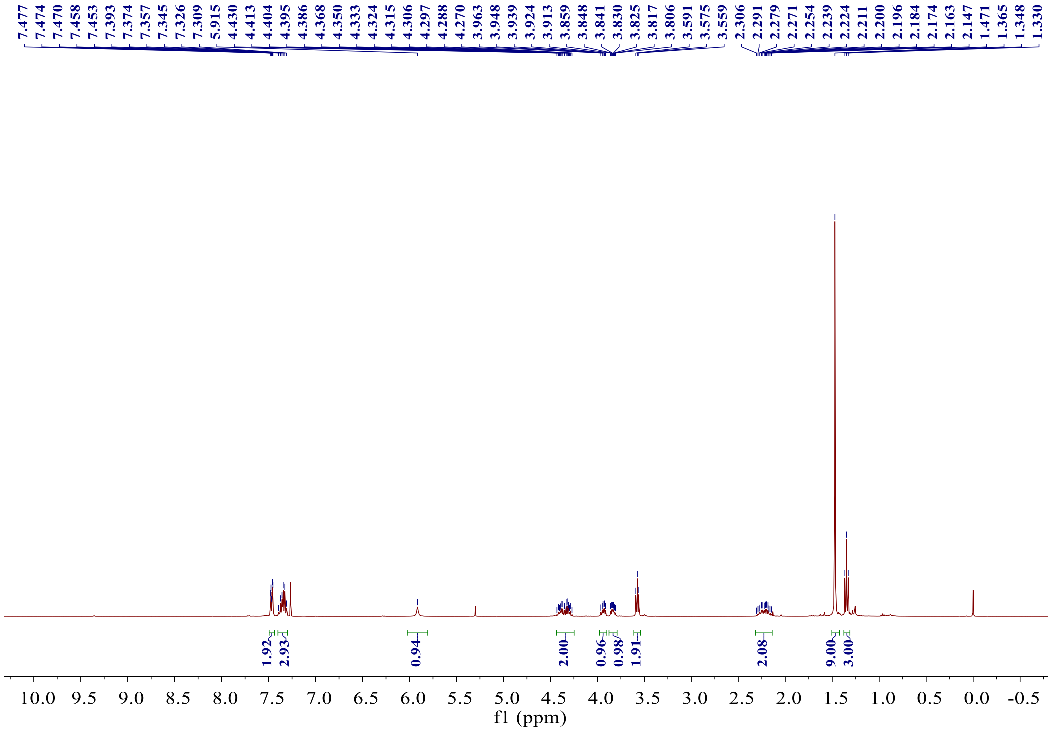

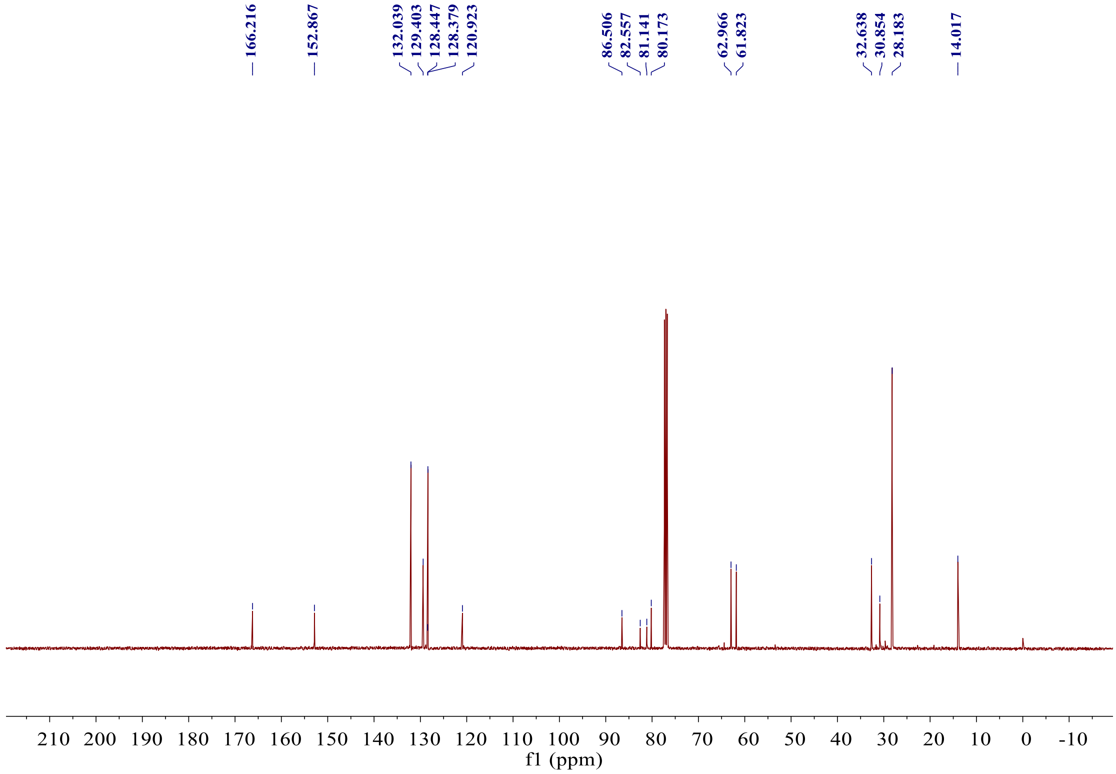

Supplementary Fig. 39. ^{1}H NMR & ^{13}C NMR spectra of compound **3w** in $CDCl_3$

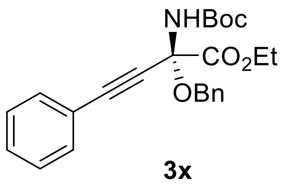

3x

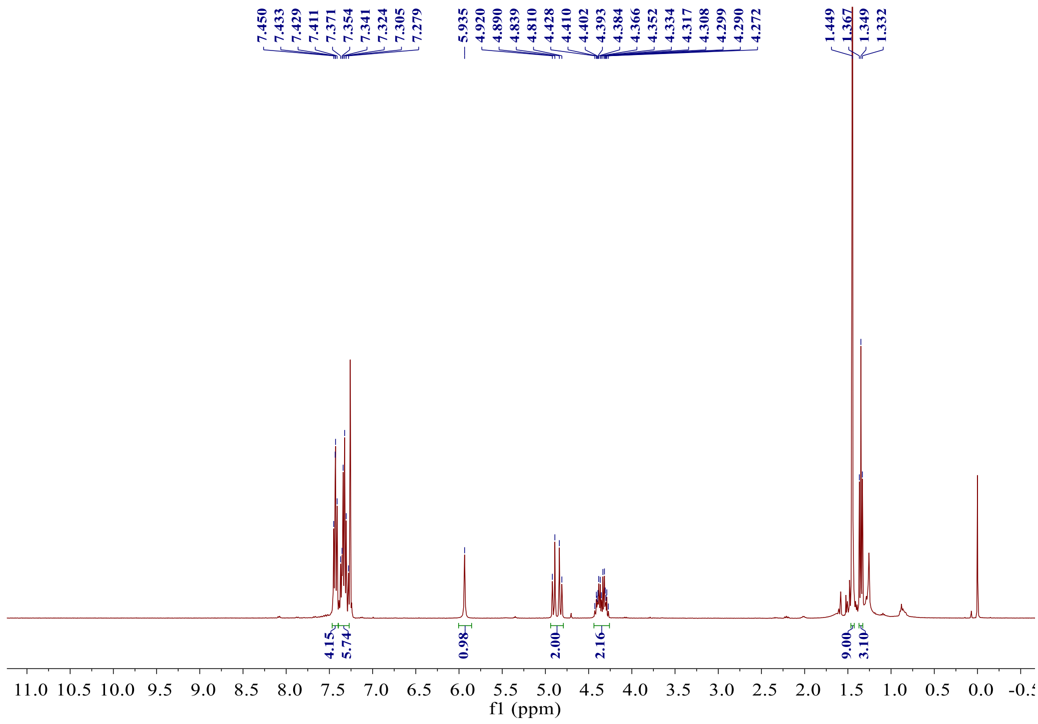

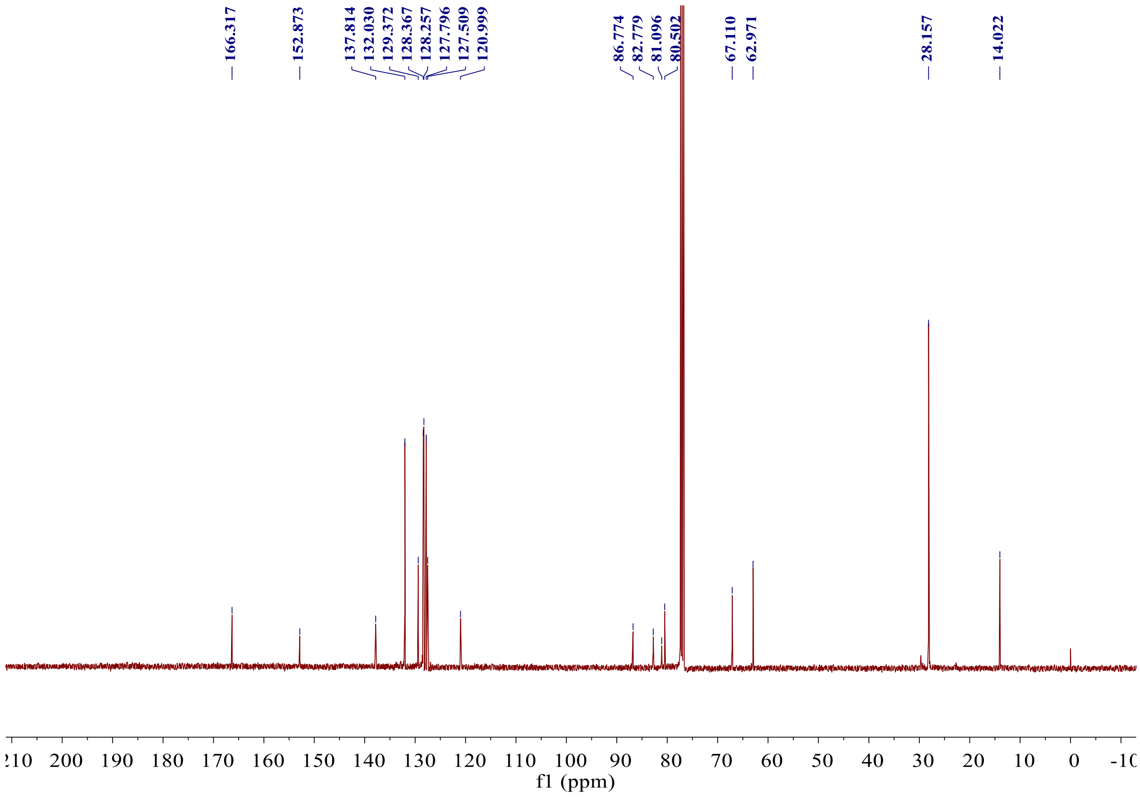

Supplementary Fig. 40. ^{1}H NMR & ^{13}C NMR spectra of compound **3x** in $CDCl_3$

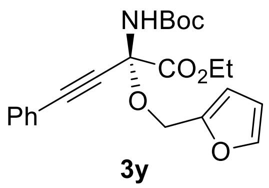

3y

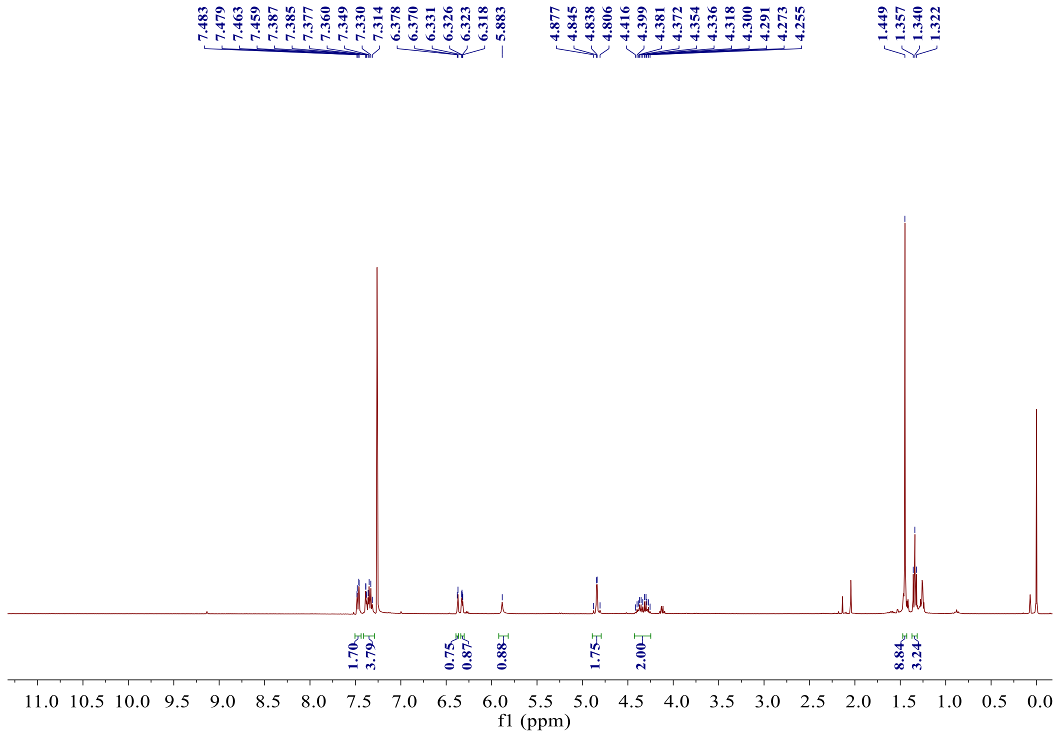

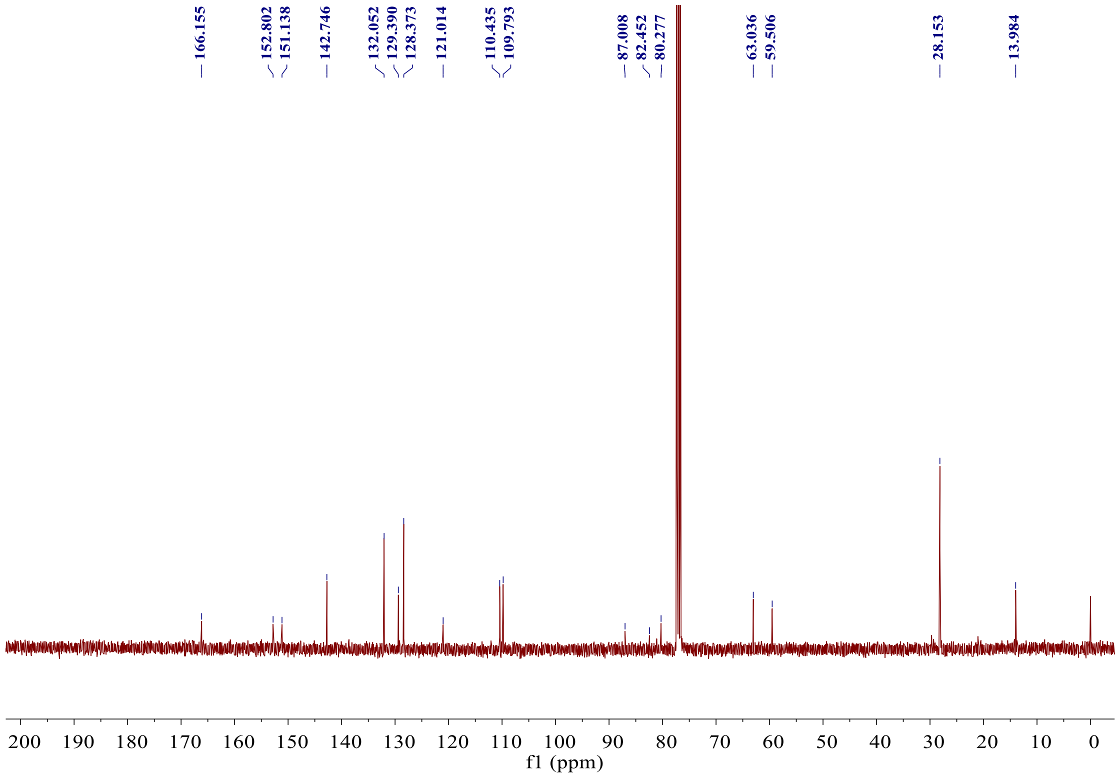

Supplementary Fig. 41. ^{1}H NMR & ^{13}C NMR spectra of compound **3y** in $CDCl_3$

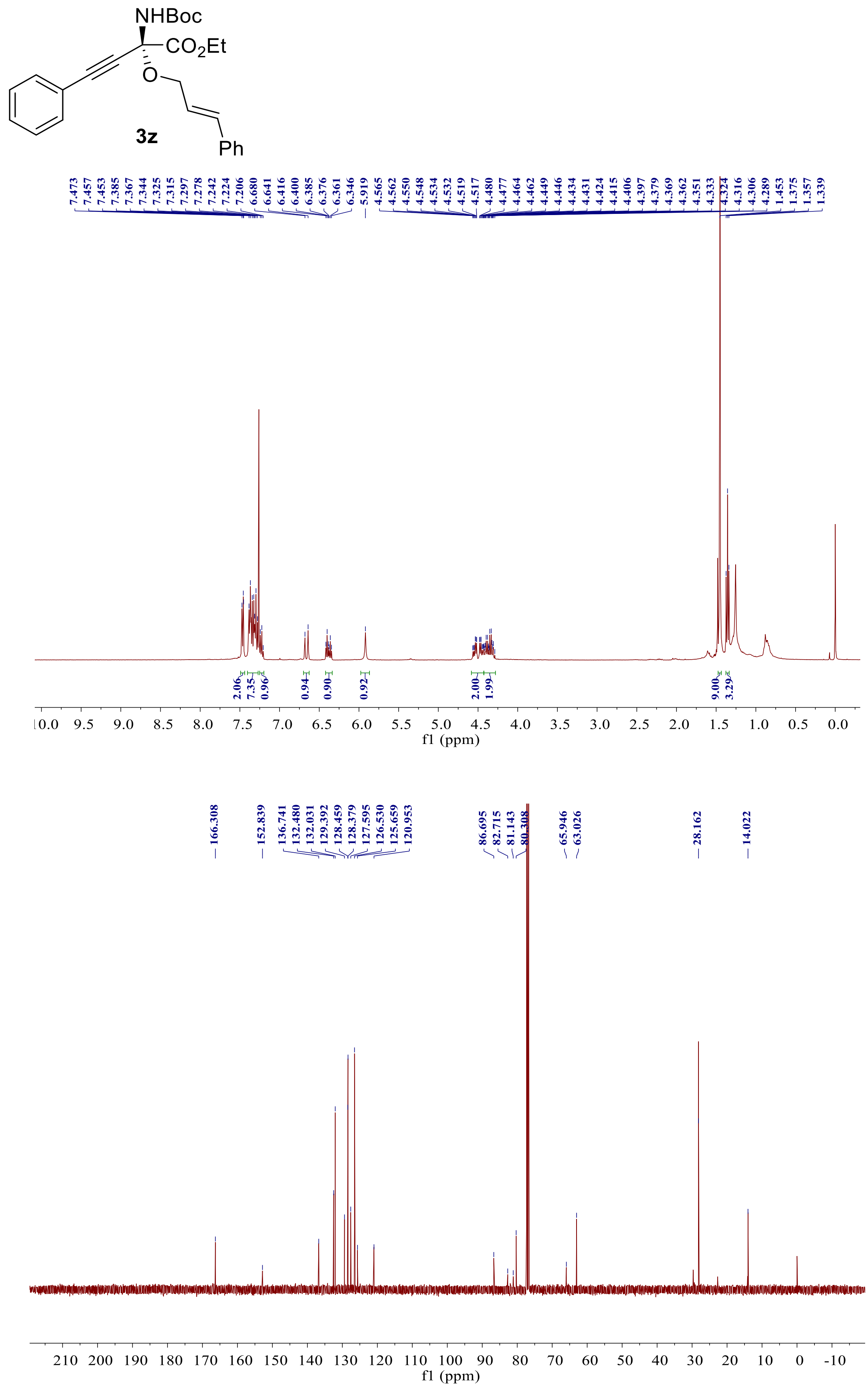

Supplementary Fig. 42. ^{1}H NMR & ^{13}C NMR spectra of compound **3z** in $CDCl_3$

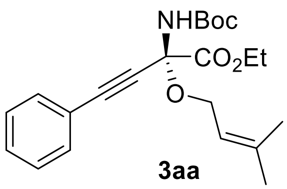

3aa

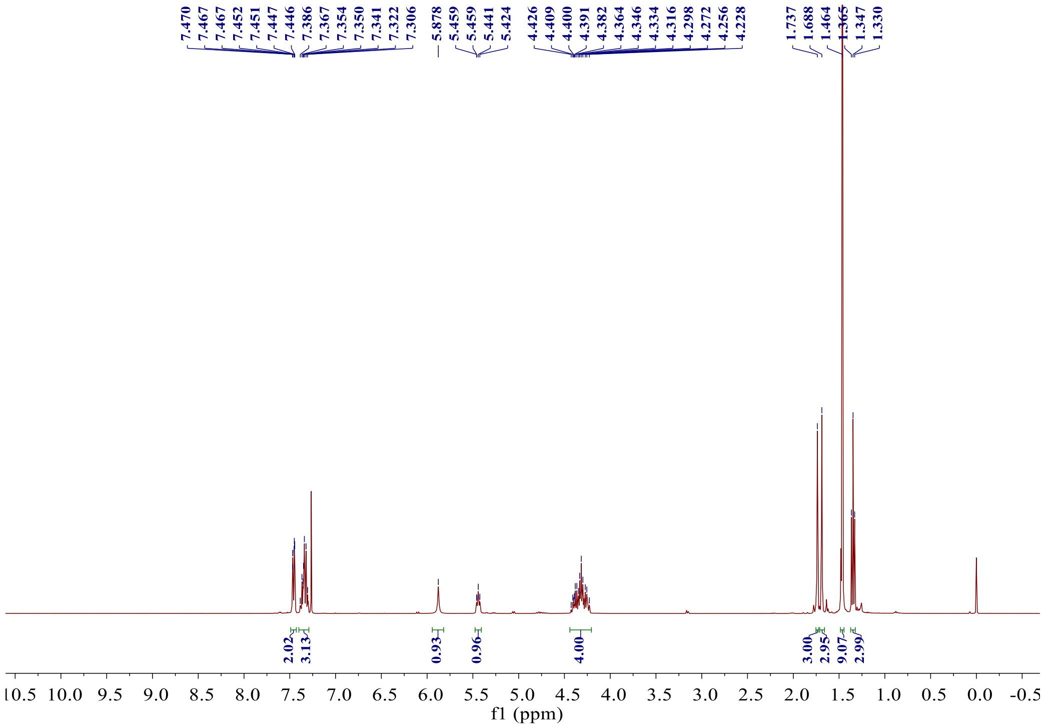

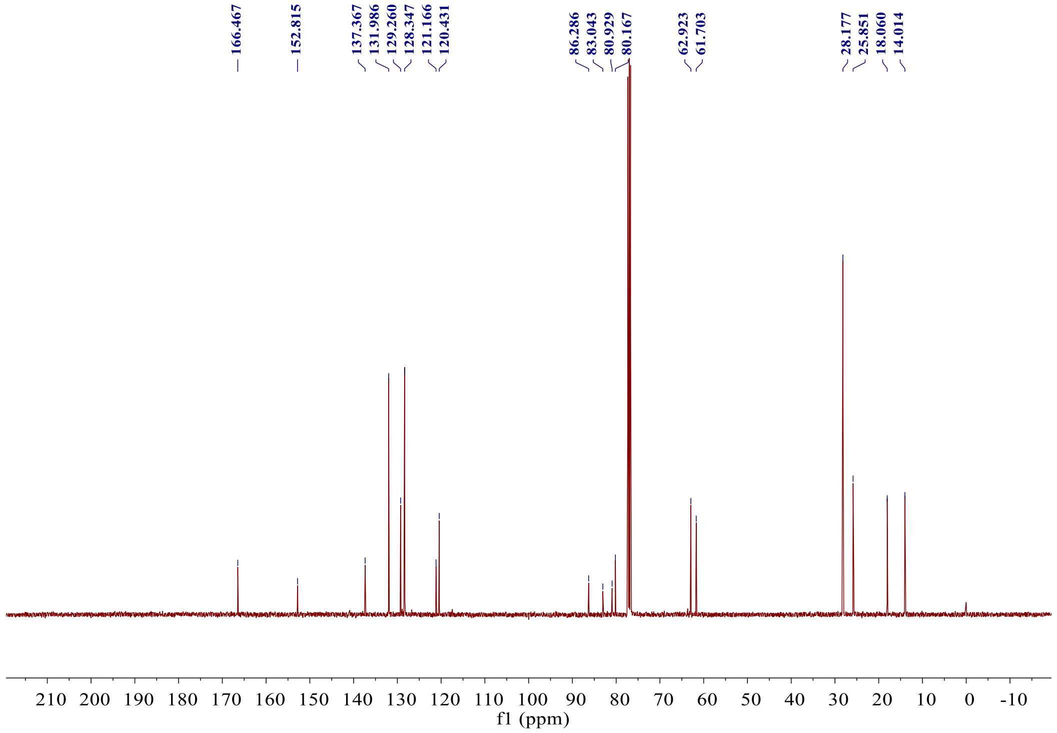

Supplementary Fig. 43. ^1H NMR & ^{13}C NMR spectra of compound **3aa** in $CDCl_3$

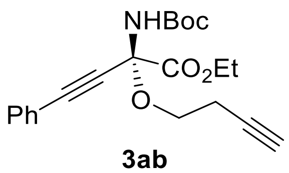

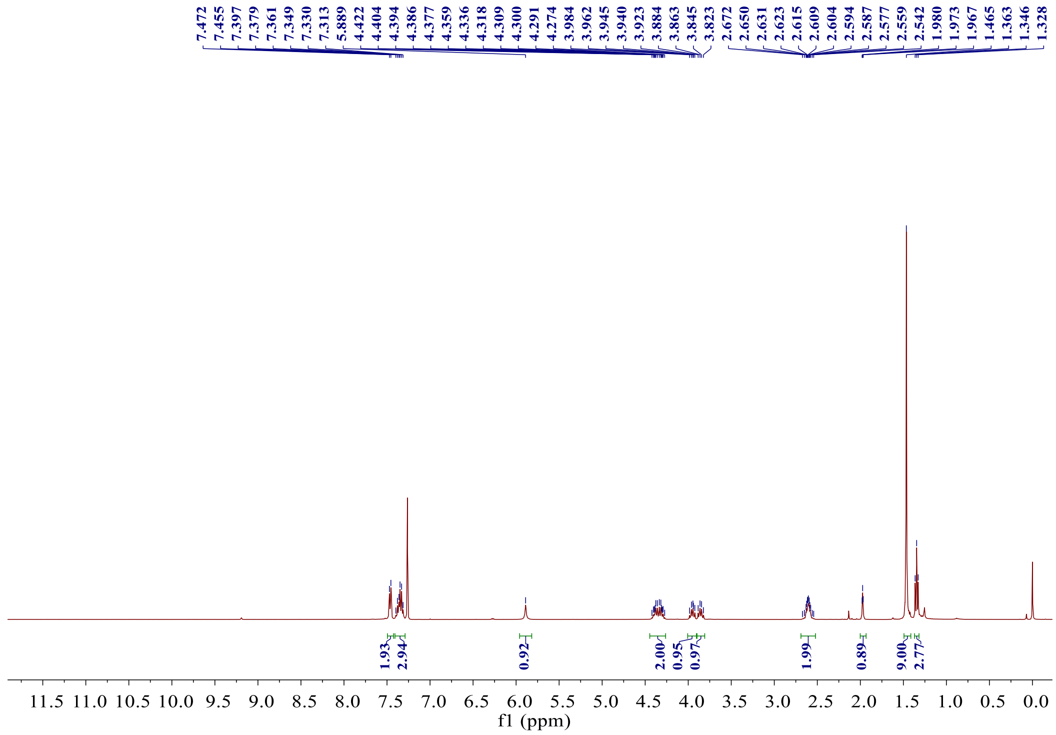

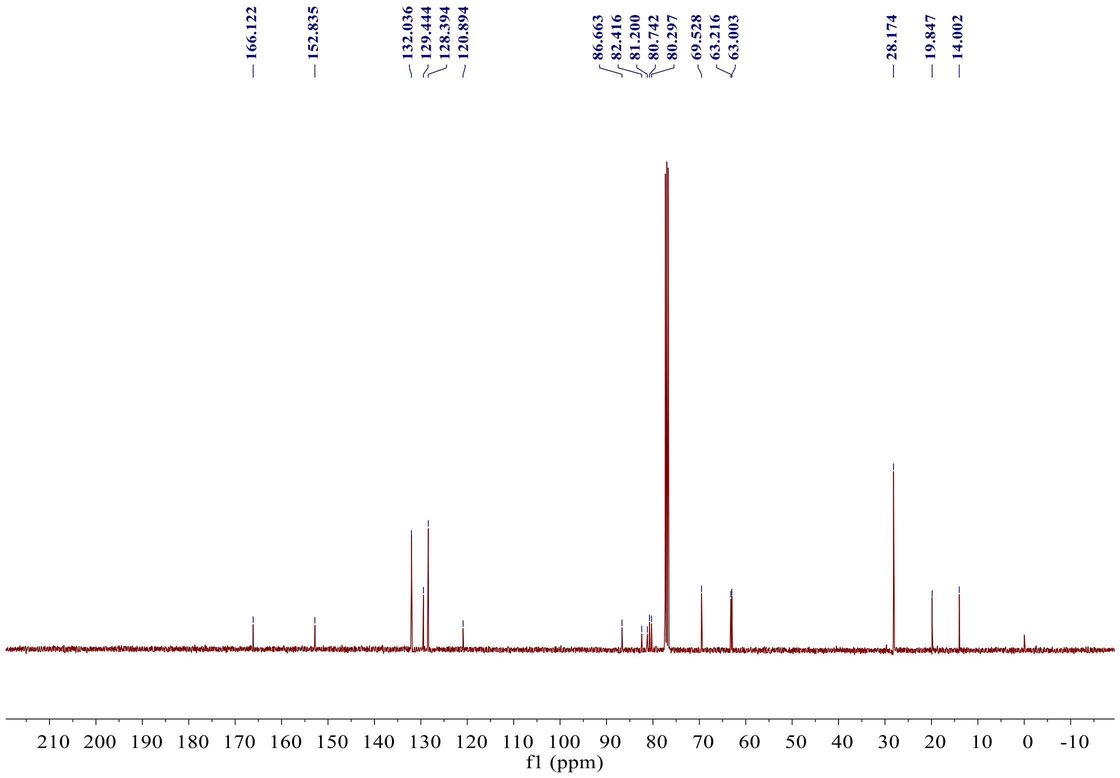

Supplementary Fig. 44. ^{1}H NMR & ^{13}C NMR spectra of compound **3ab** in $CDCl_3$

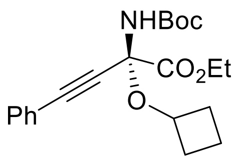

3ac

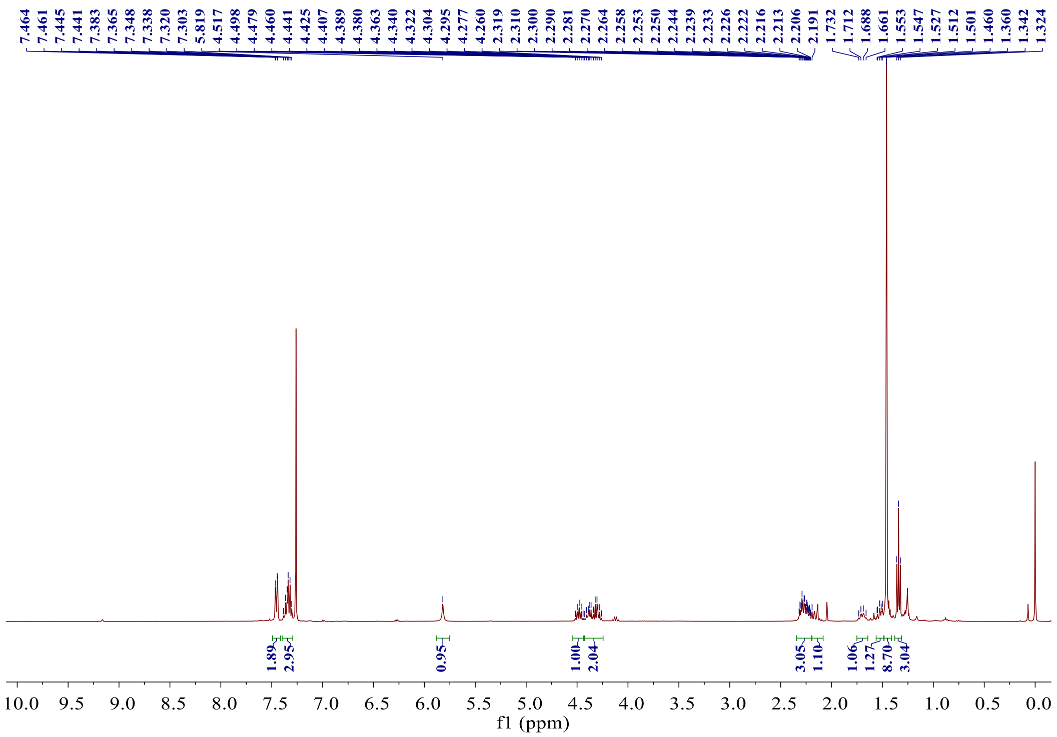

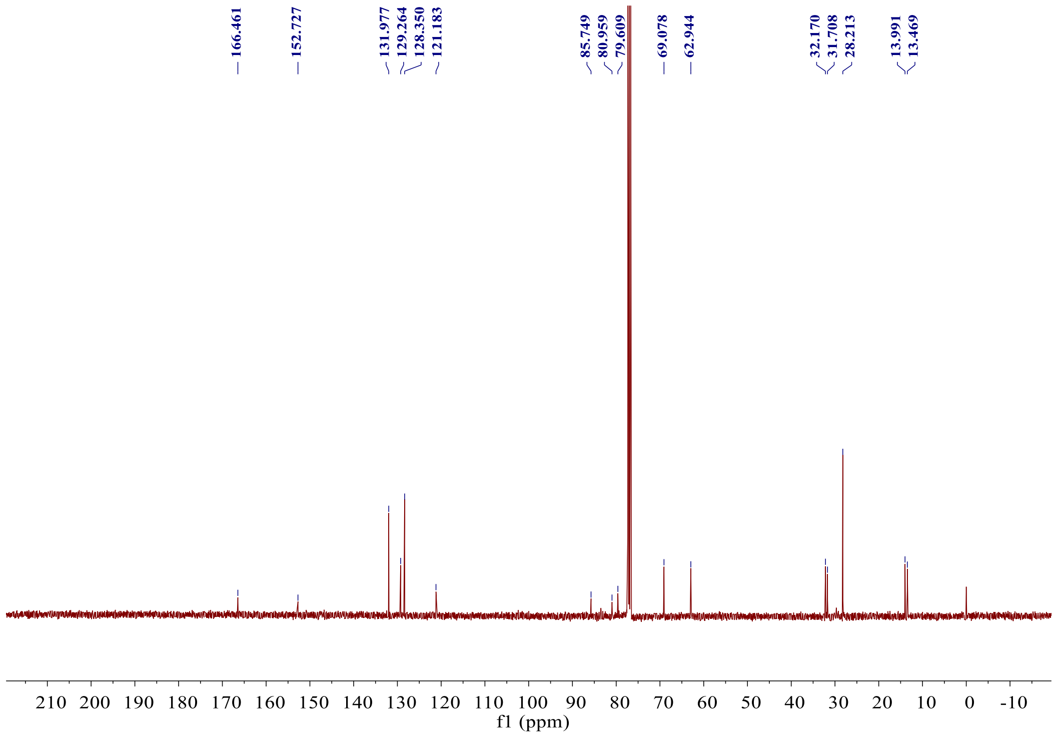

Supplementary Fig. 45. ^{1}H NMR & ^{13}C NMR spectra of compound **3ac** in $CDCl_3$

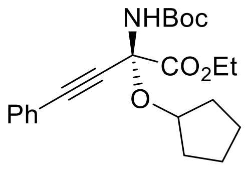

3ad

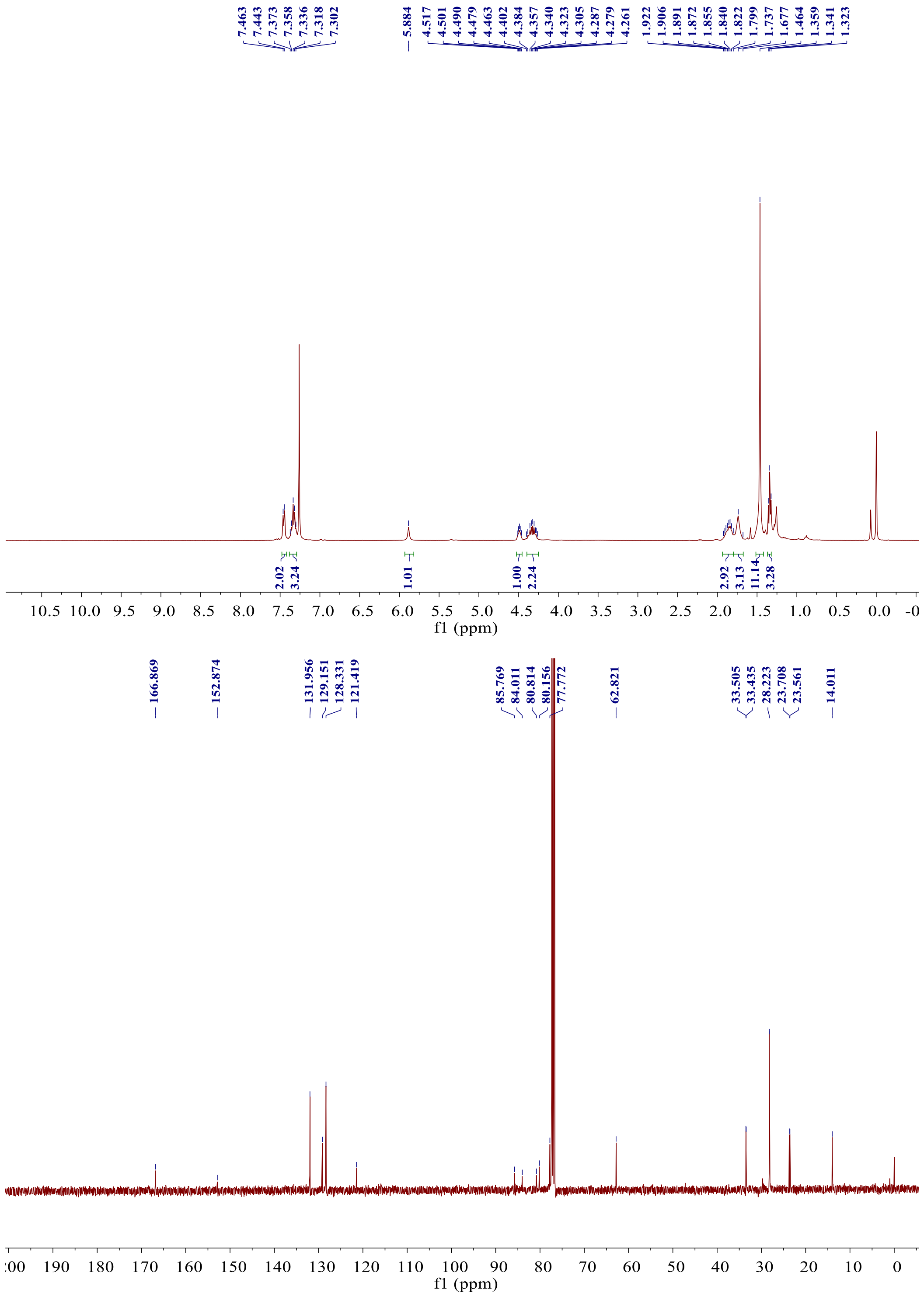

Supplementary Fig. 46. ^{1}H NMR & ^{13}C NMR spectra of compound **3ad** in $CDCl_3$

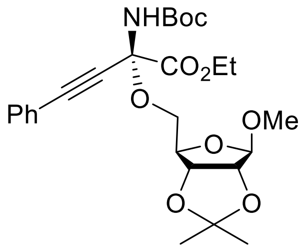

3ae

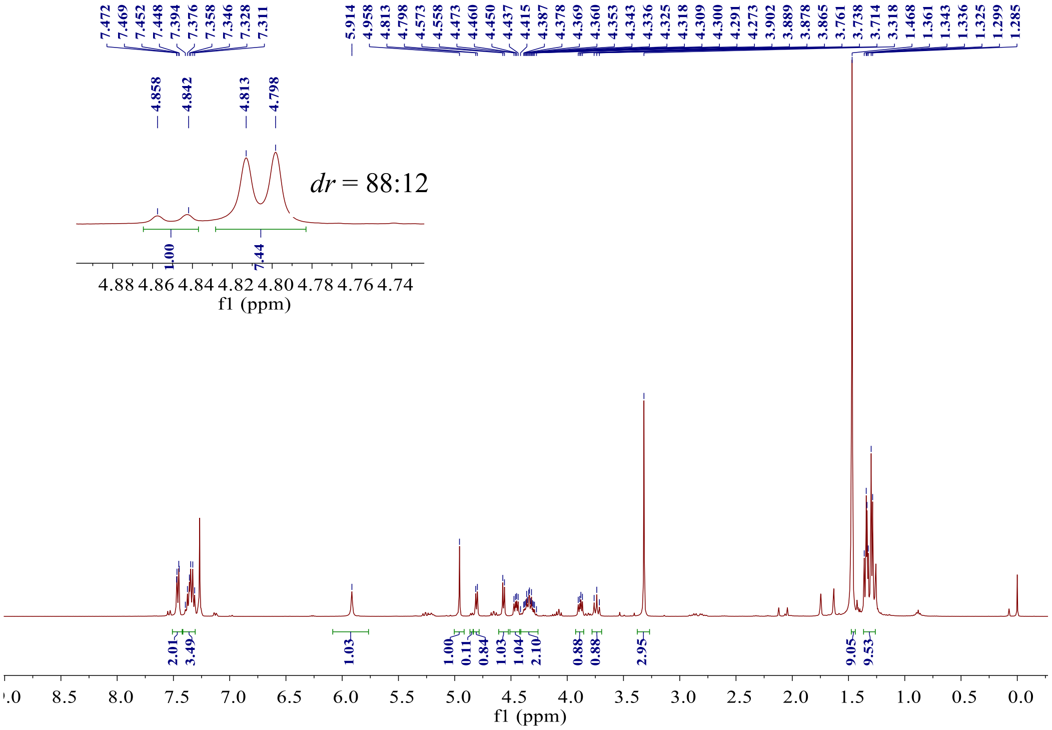

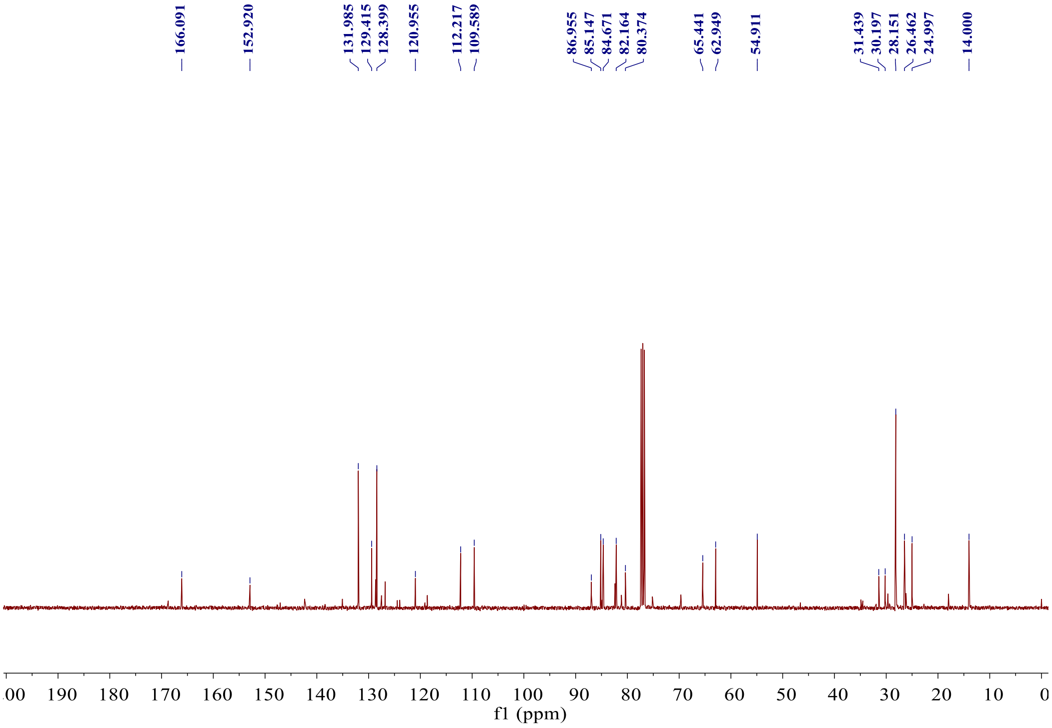

Supplementary Fig. 47. ¹H NMR & ¹³C NMR spectra of compound **3ae** in $CDCl_3$

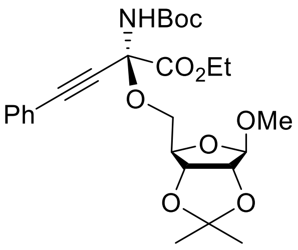

3ae'

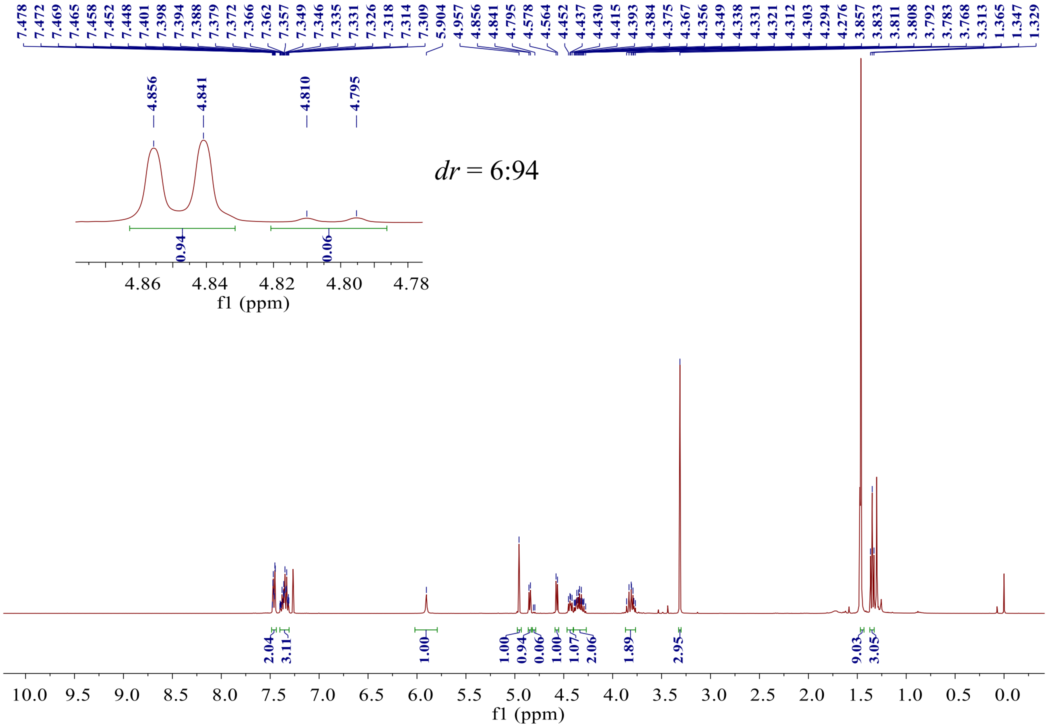

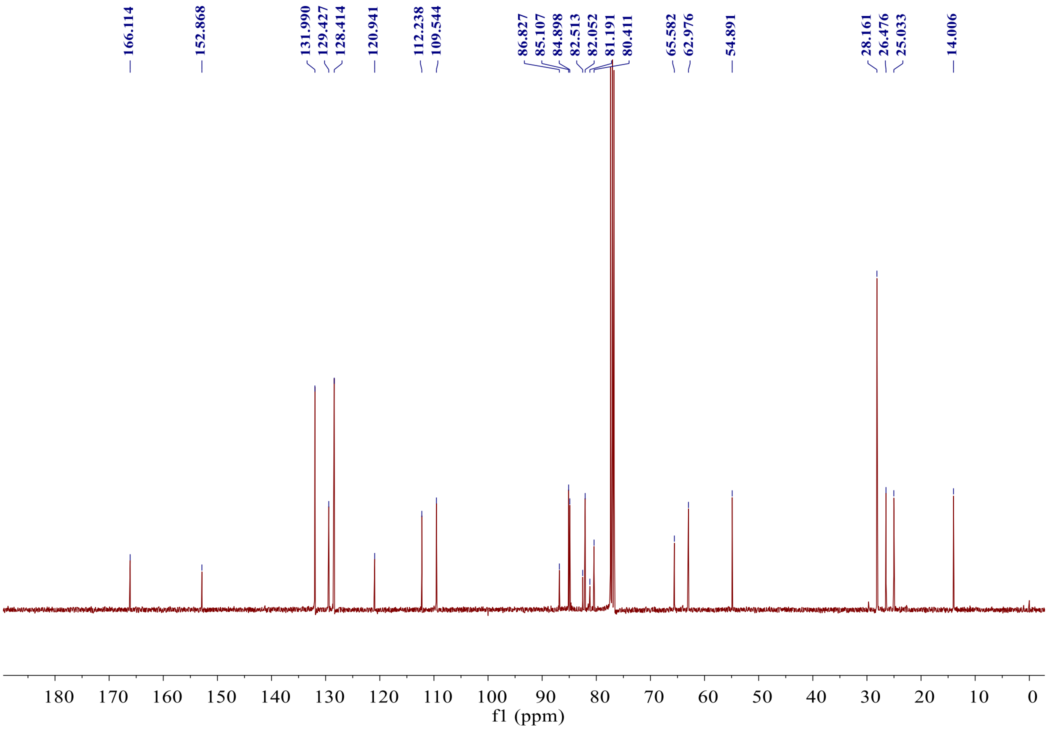

Supplementary Fig. 48. ^{1}H NMR & ^{13}C NMR spectra of compound **3ae′** in $CDCl_3$

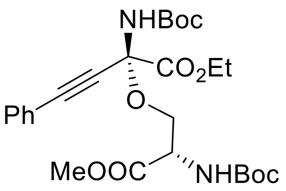

3af

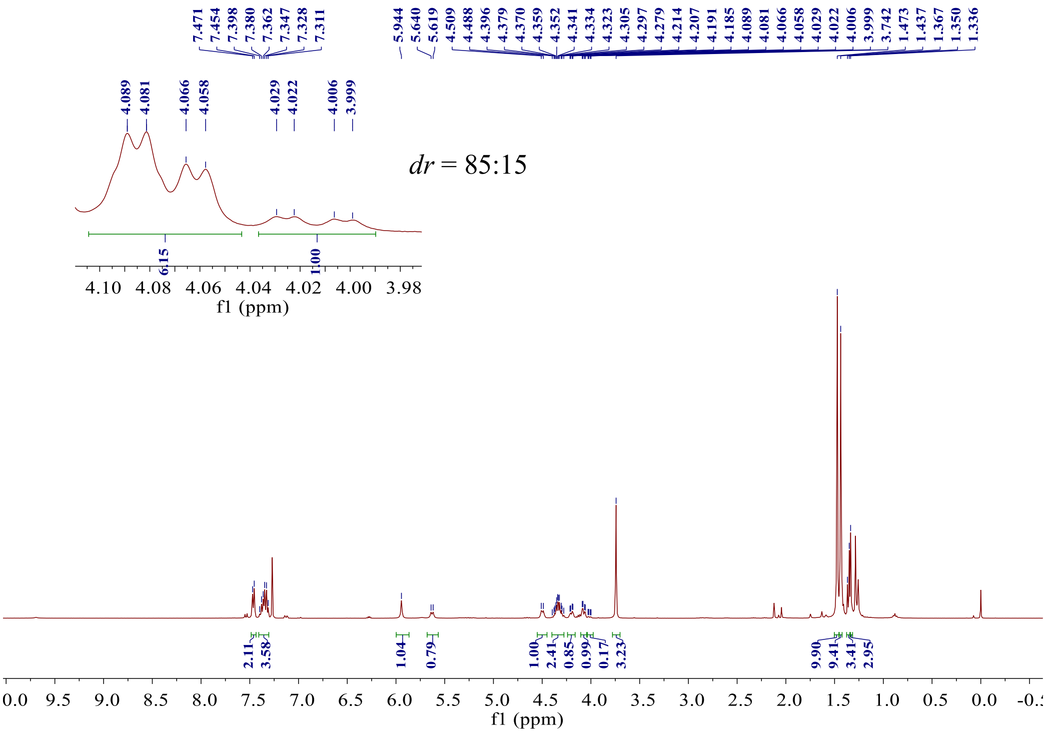

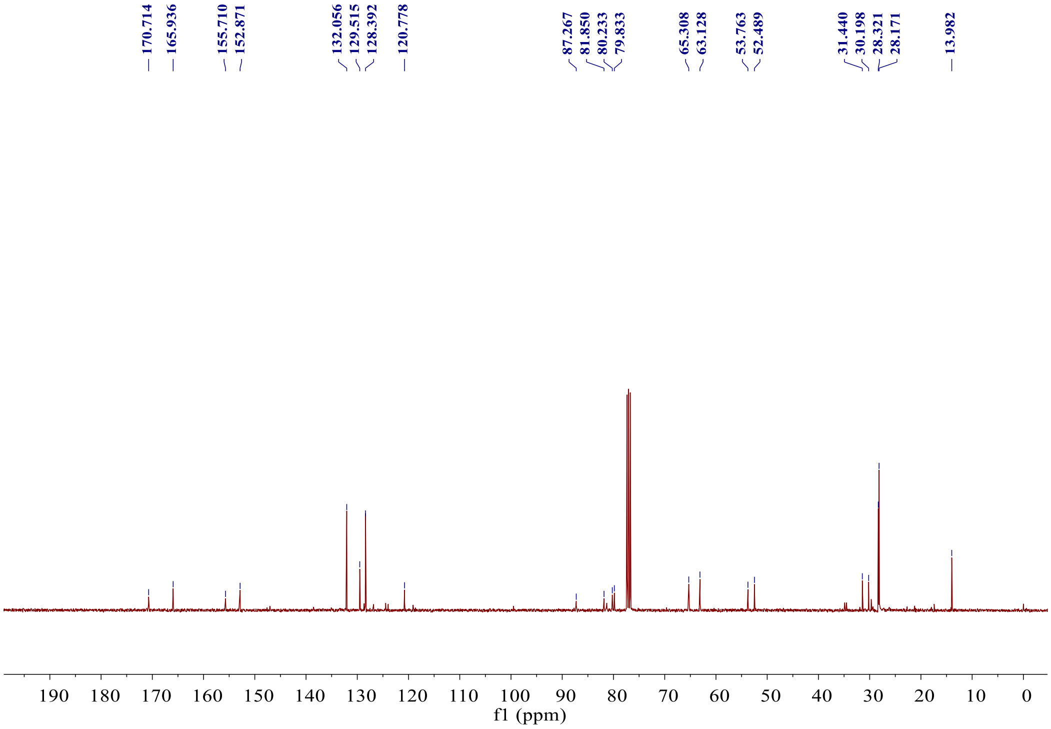

Supplementary Fig. 49. ^{1}H NMR & ^{13}C NMR spectra of compound **3af** in $CDCl_3$

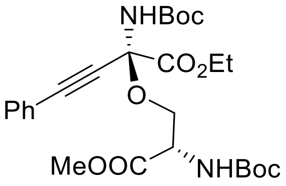

3af'

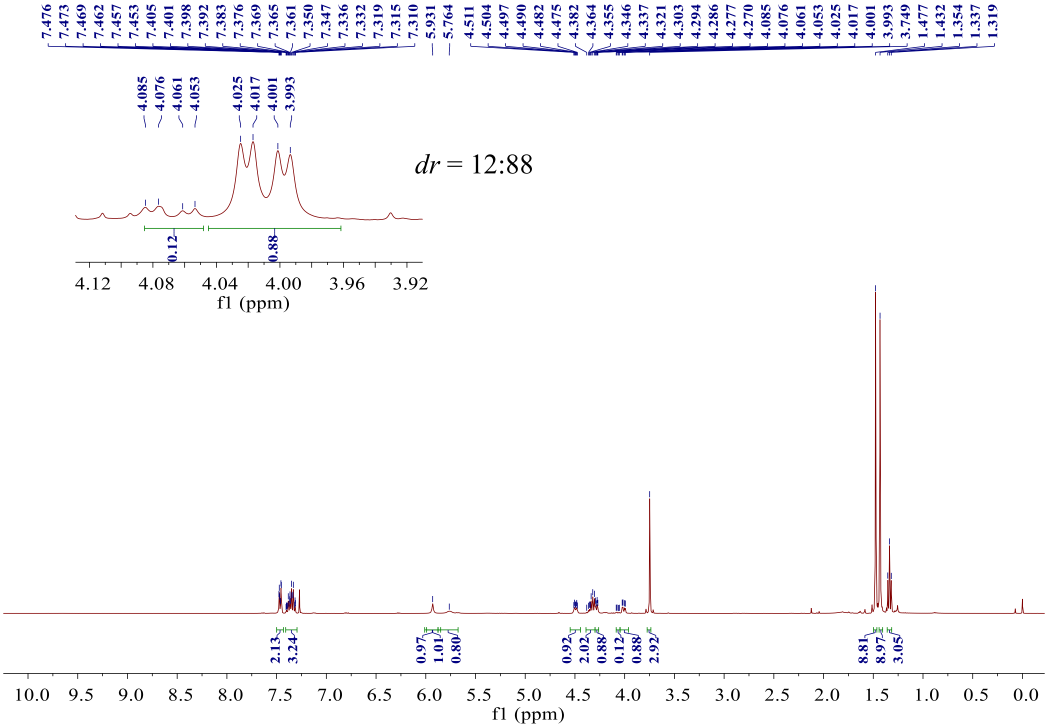

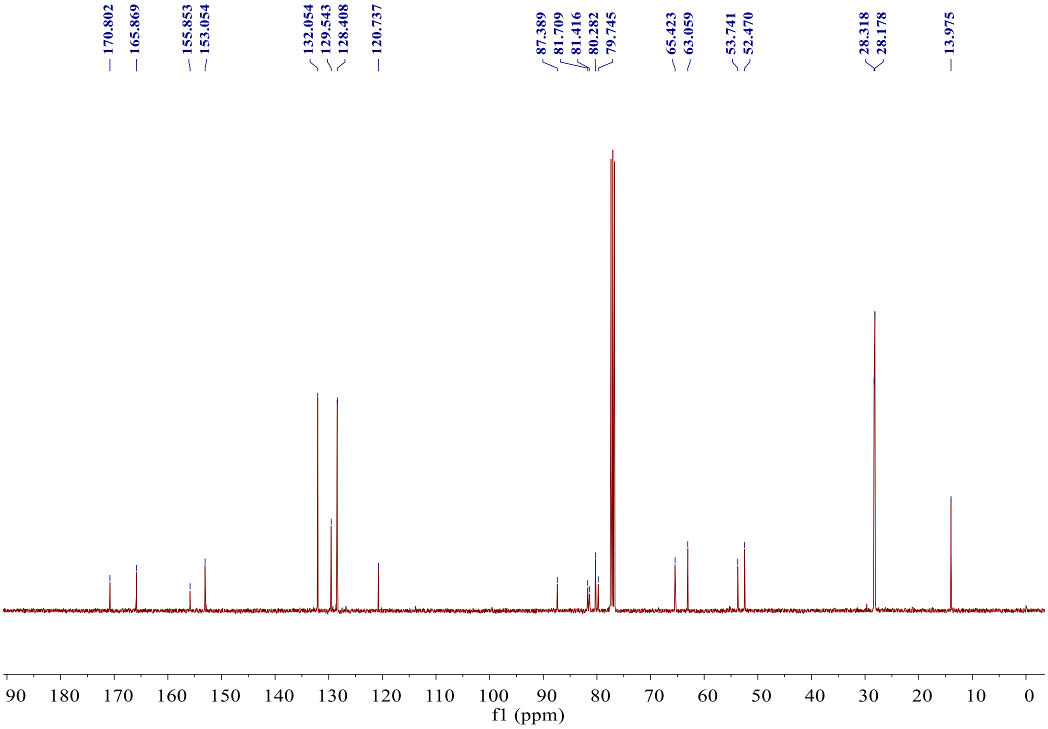

Supplementary Fig. 50. ^{1}H NMR & ^{13}C NMR spectra of compound **3af′** in $CDCl_3$

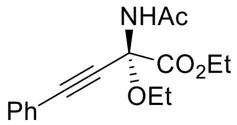

3ah

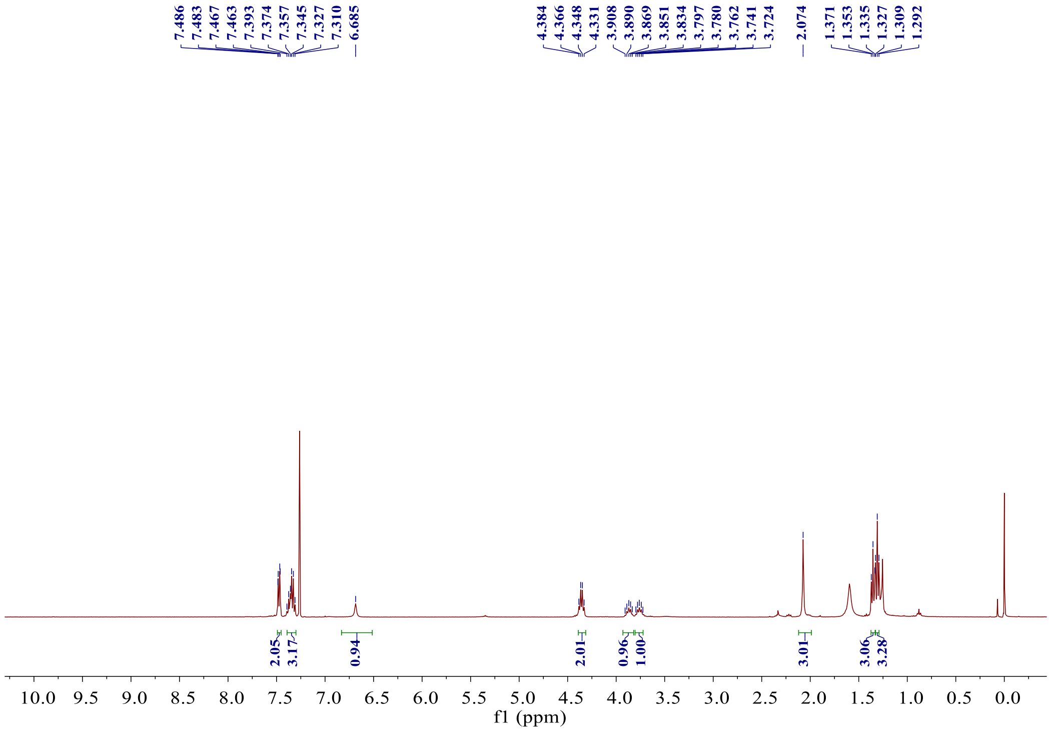

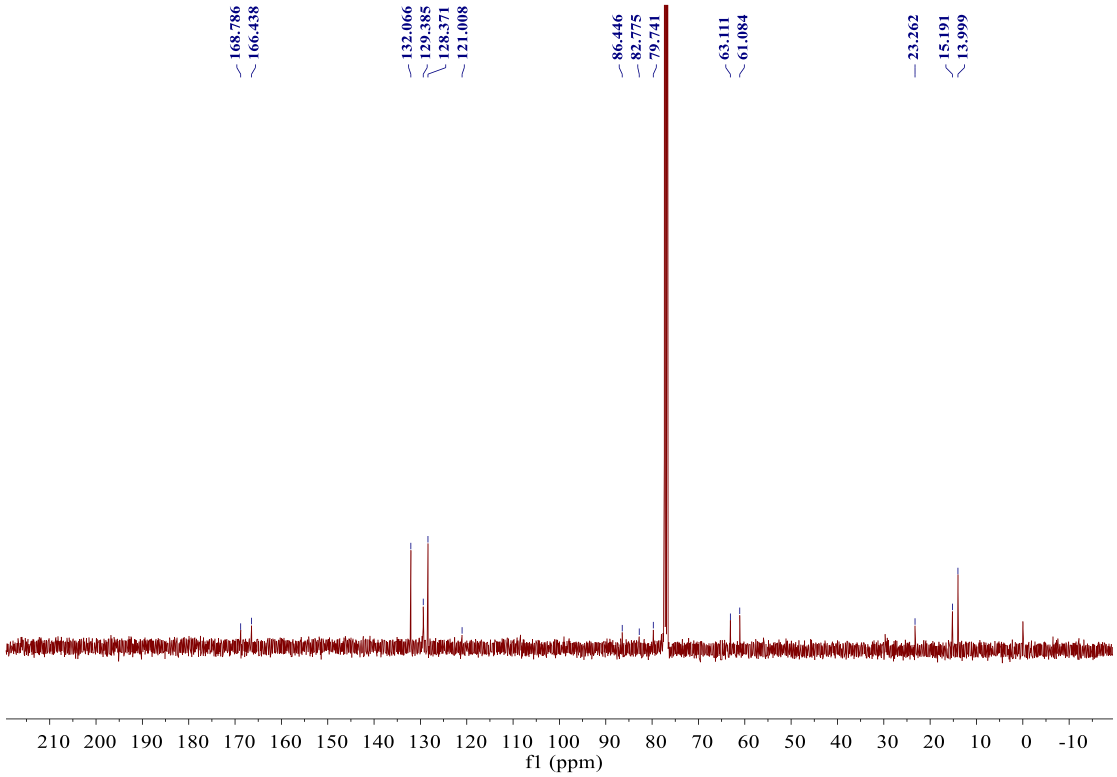

Supplementary Fig. 51. ^{1}H NMR & ^{13}C NMR spectra of compound **3ah** in $CDCl_3$

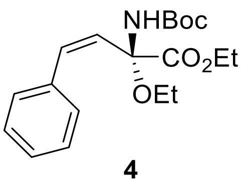

4

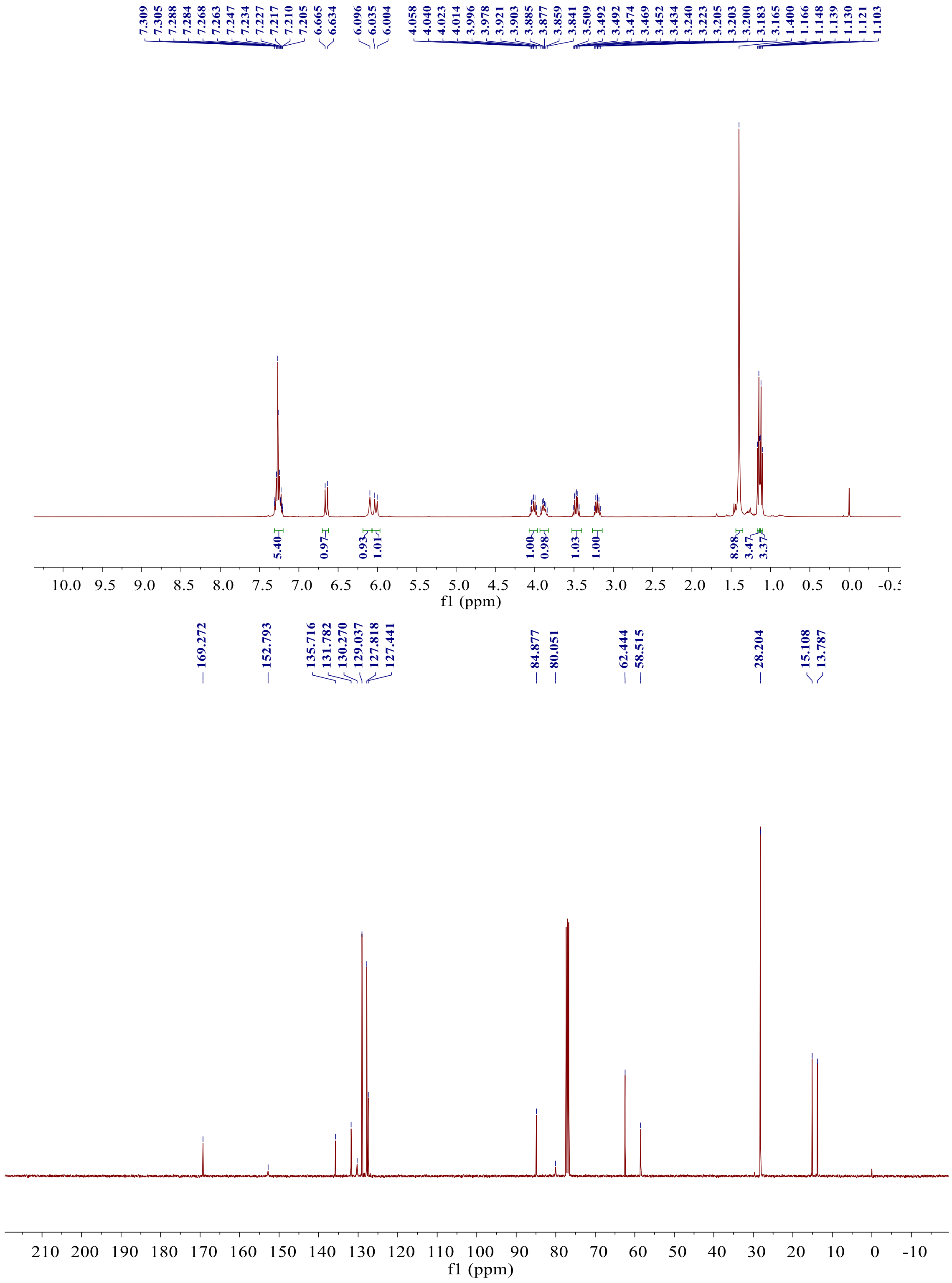

Supplementary Fig. 52. ^{1}H NMR & ^{13}C NMR spectra of compound **4** in $CDCl_3$

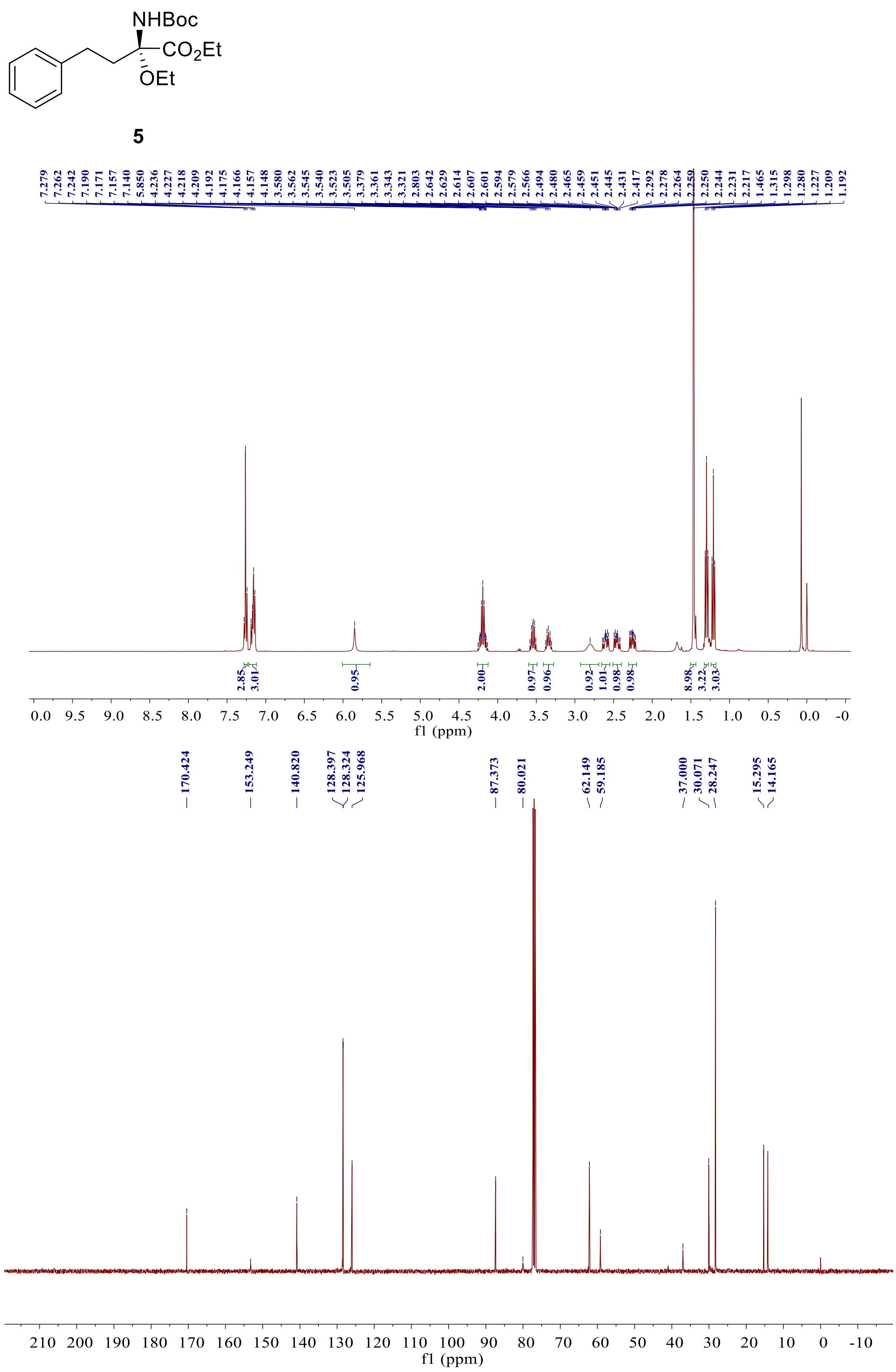

Supplementary Fig. 53. ^{1}H NMR & ^{13}C NMR spectra of compound **5** in $CDCl_3$

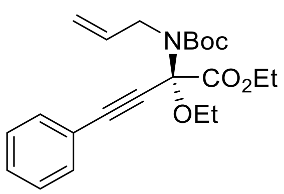

6

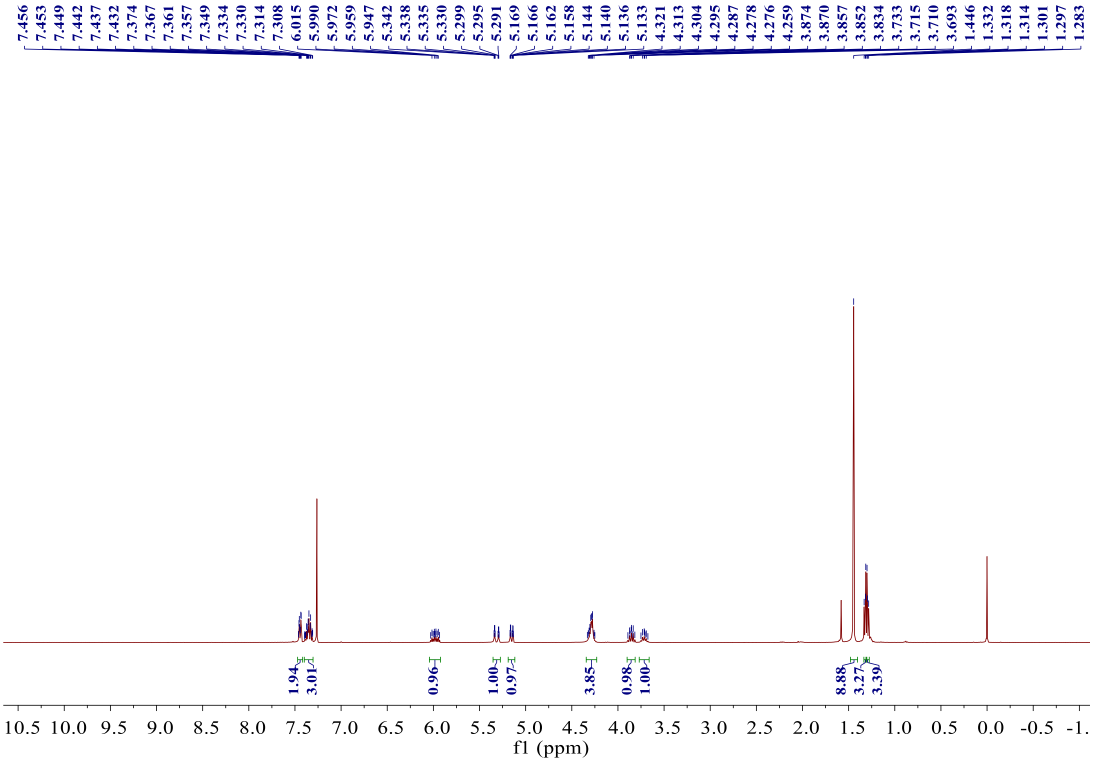

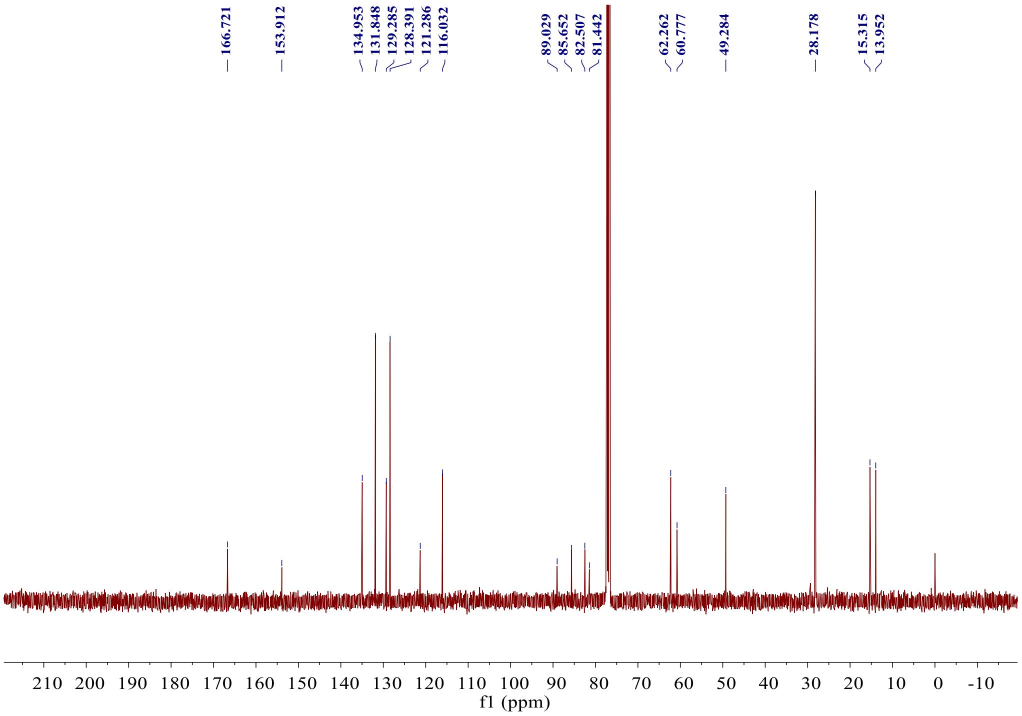

Supplementary Fig. 54. ^{1}H NMR & ^{13}C NMR spectra of compound **6** in $CDCl_3$

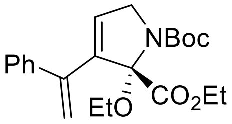

7

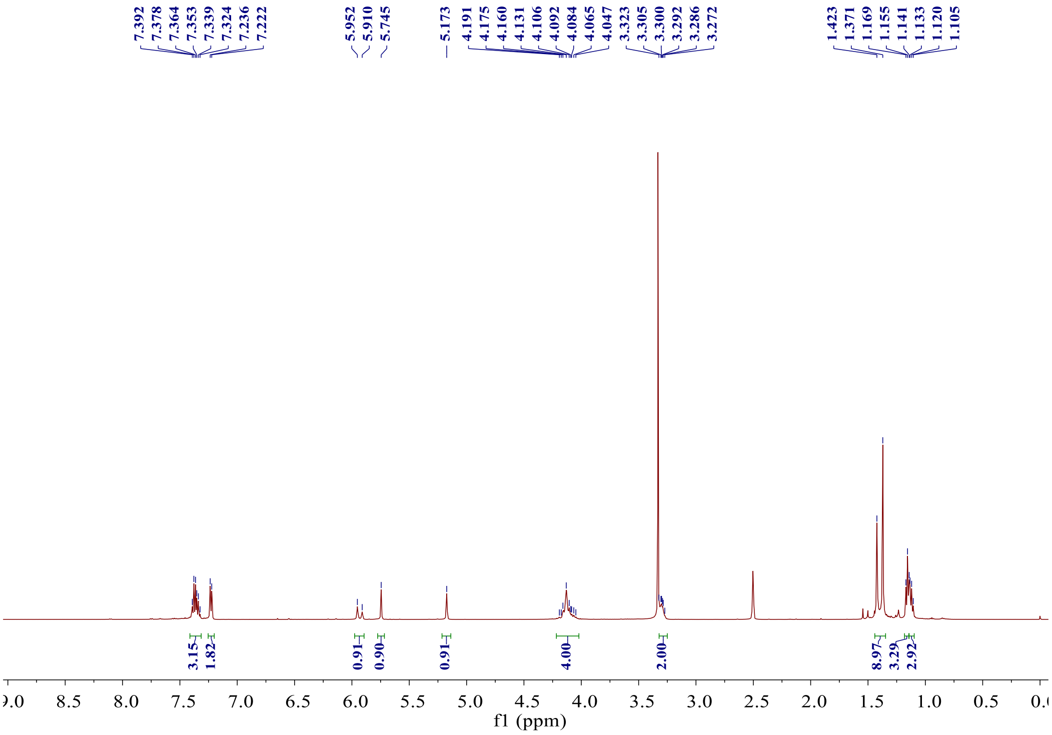

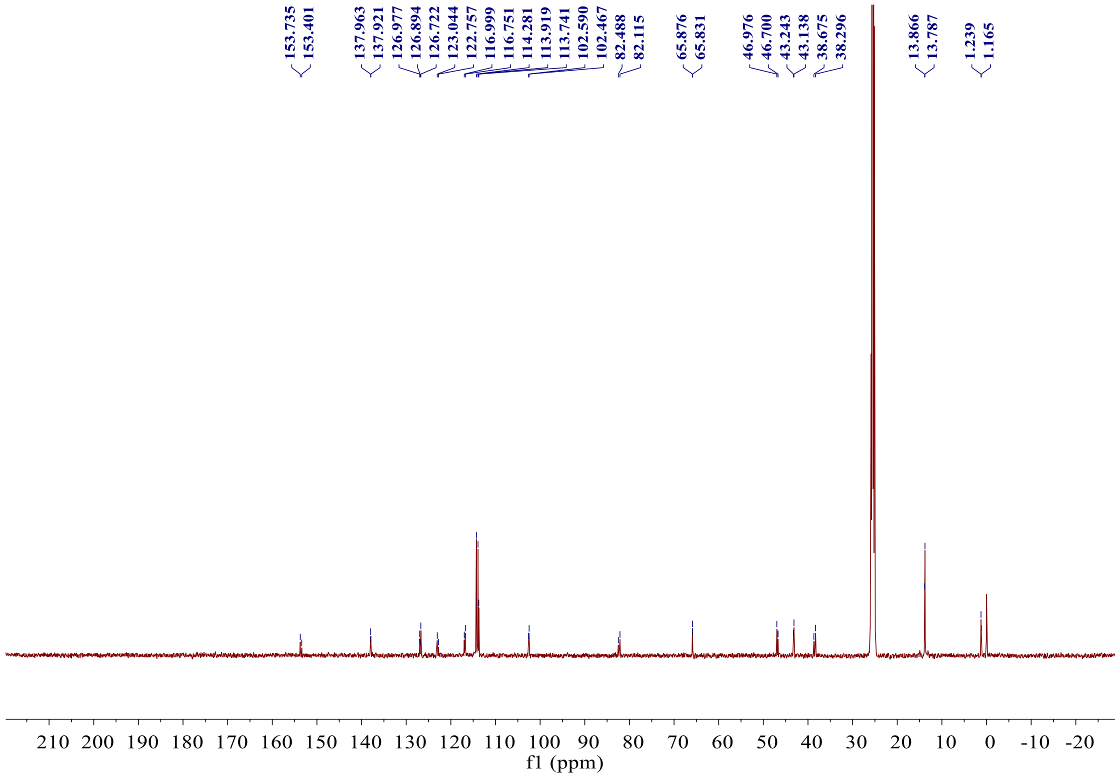

Supplementary Fig. 55. ^{1}H NMR & ^{13}C NMR spectra of compound **7** in DMSO-d_6

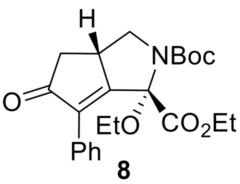

8

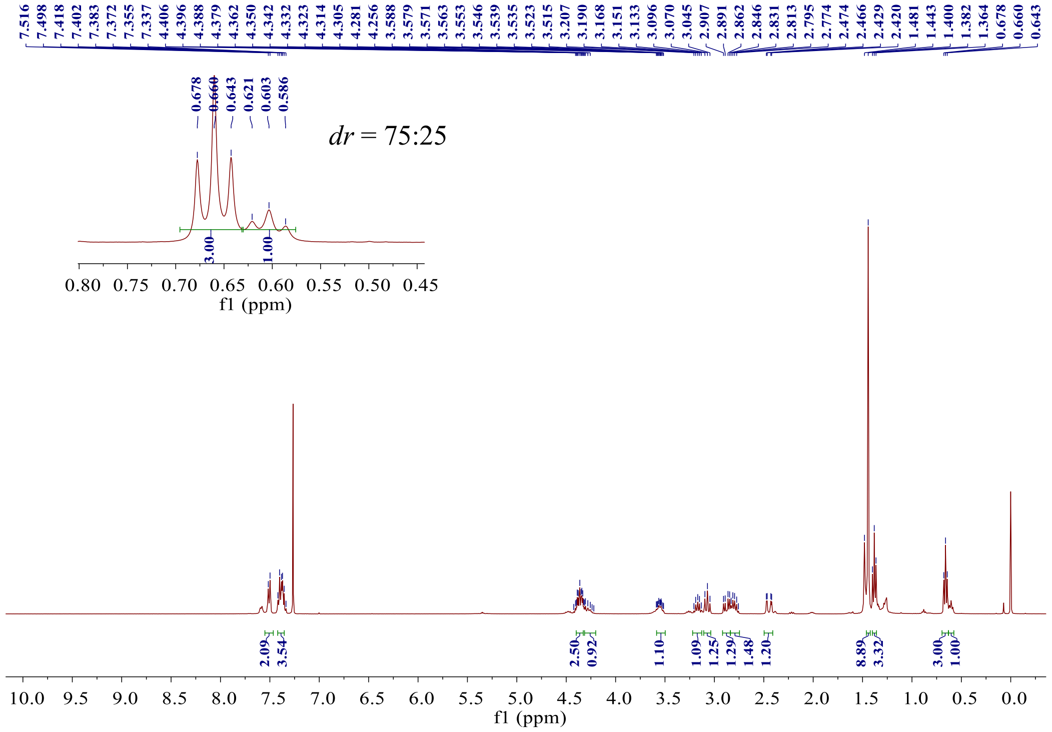

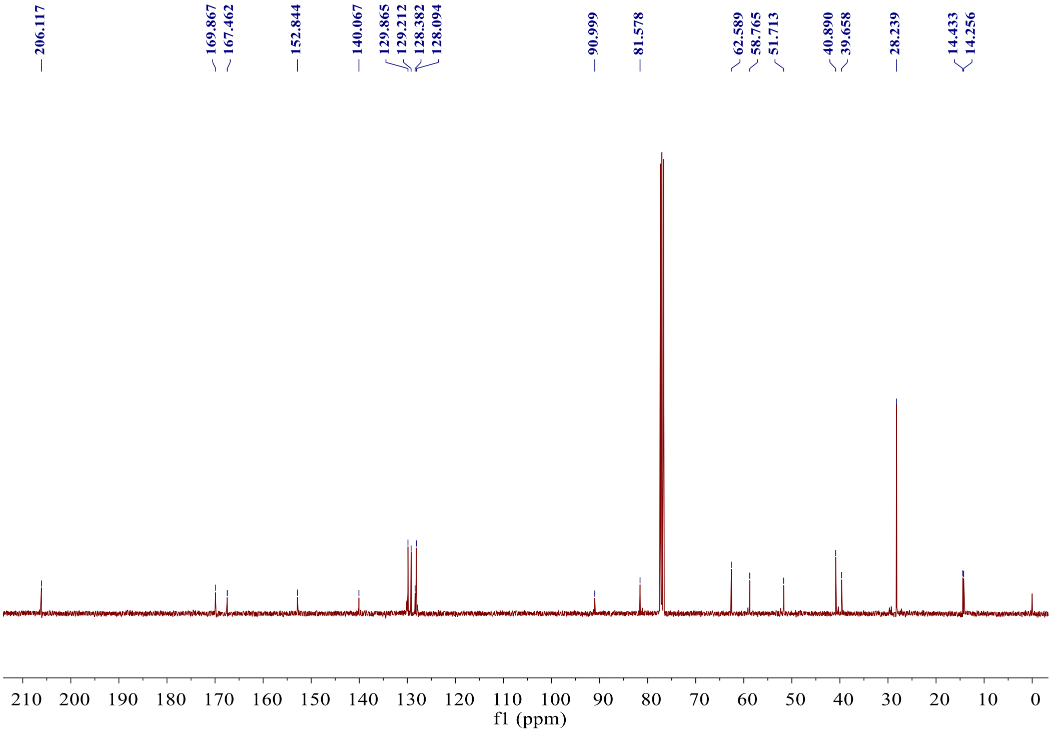

Supplementary Fig. 56. ^{1}H NMR & ^{13}C NMR spectra of compound **8** in $CDCl_3$

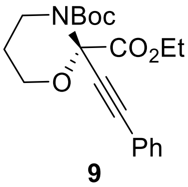

9

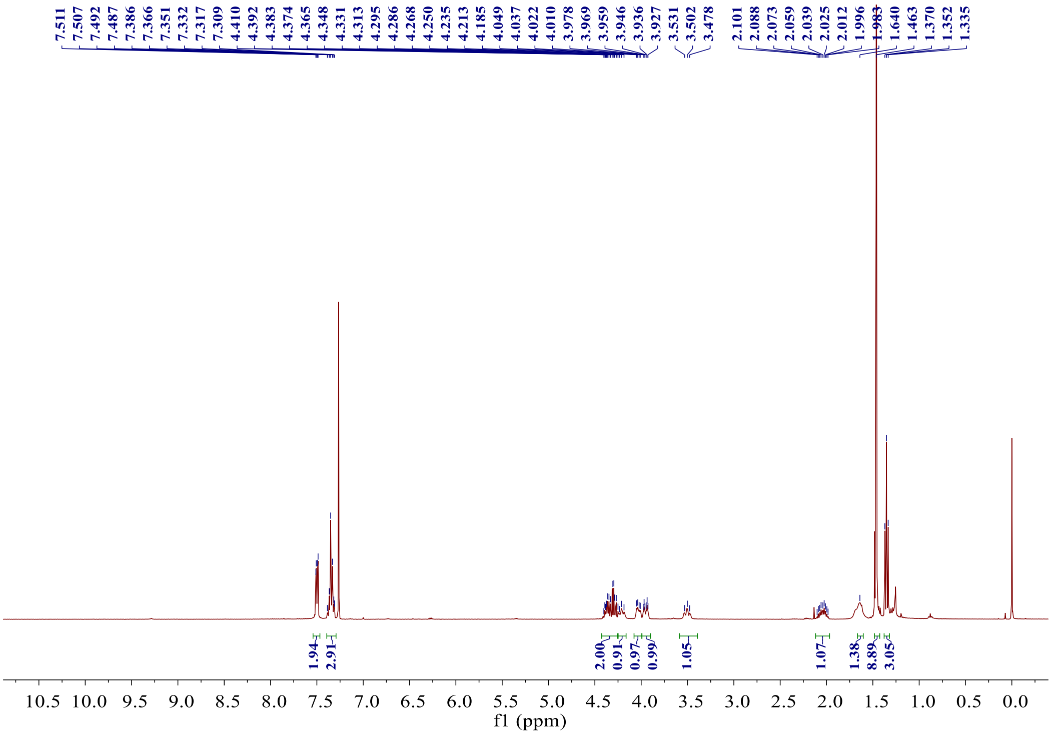

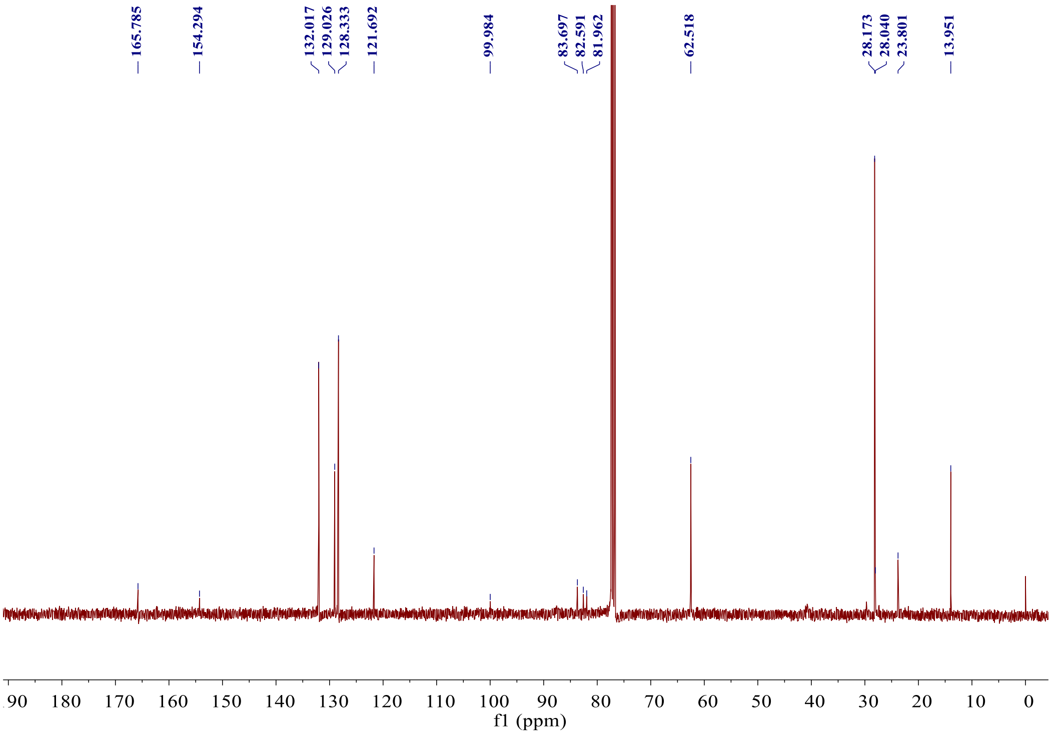

Supplementary Fig. 57. ^{1}H NMR & ^{13}C NMR spectra of compound **9** in $CDCl_3$

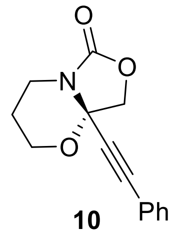

10

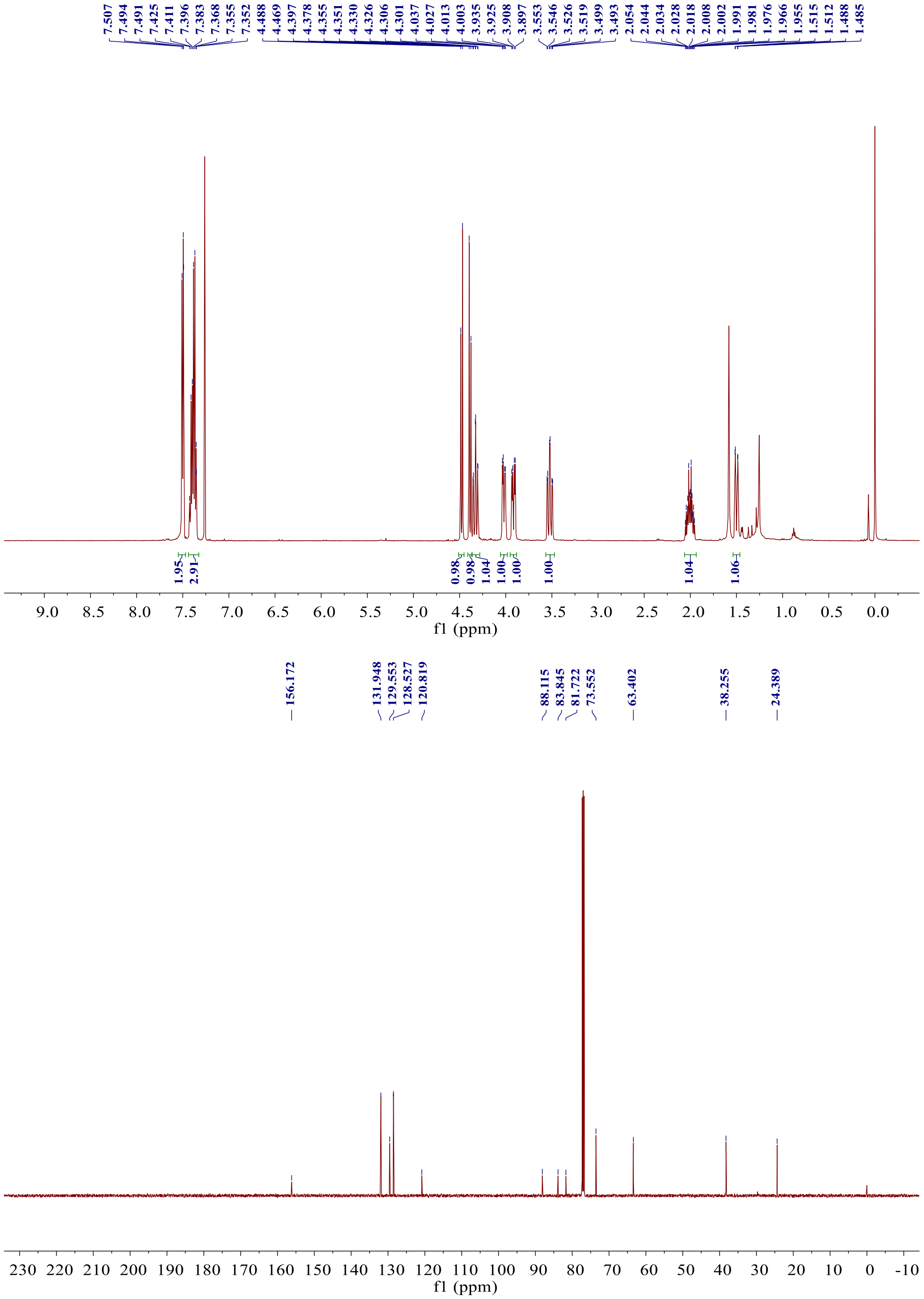

Supplementary Fig. 58. ^{1}H NMR & ^{13}C NMR spectra of compound **10** in $CDCl_3$

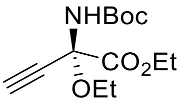

11

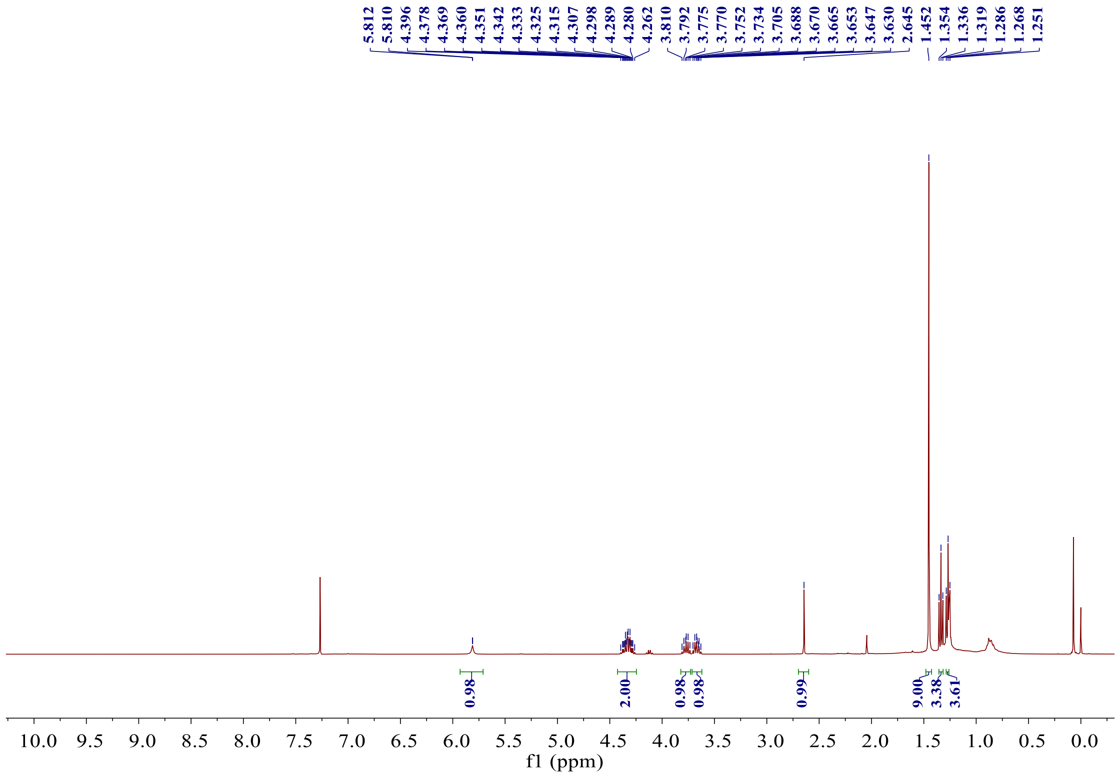

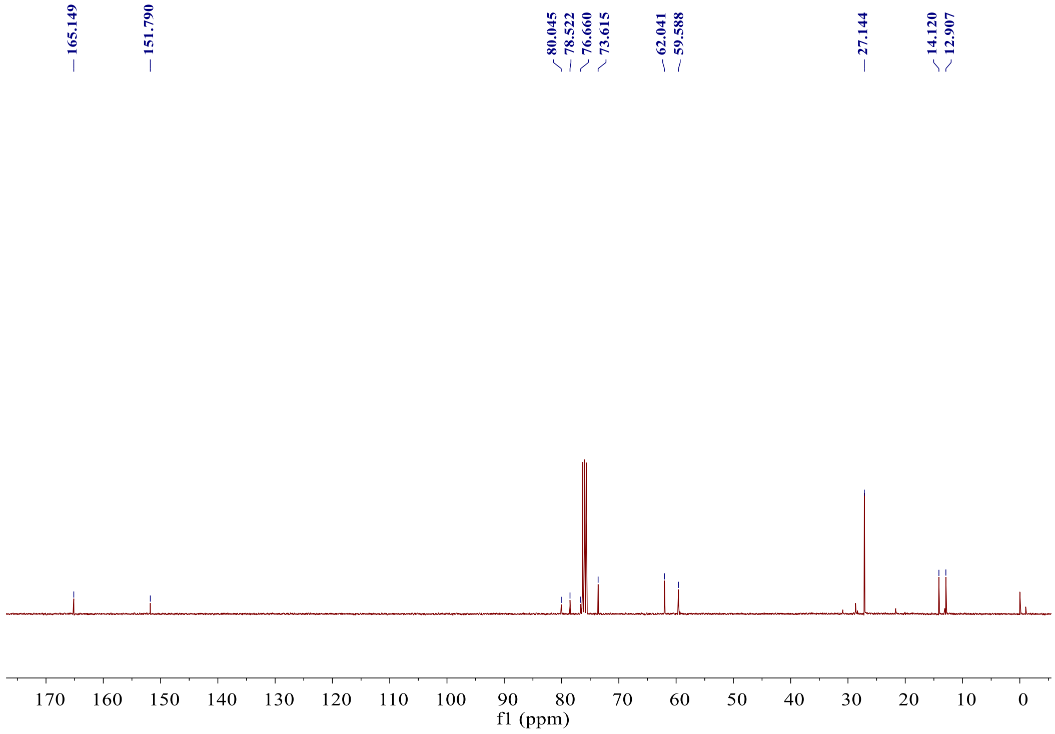

Supplementary Fig. 59. ^{1}H NMR & ^{13}C NMR spectra of compound **11** in $CDCl_3$

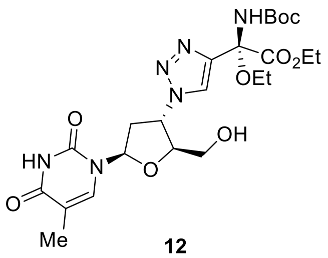

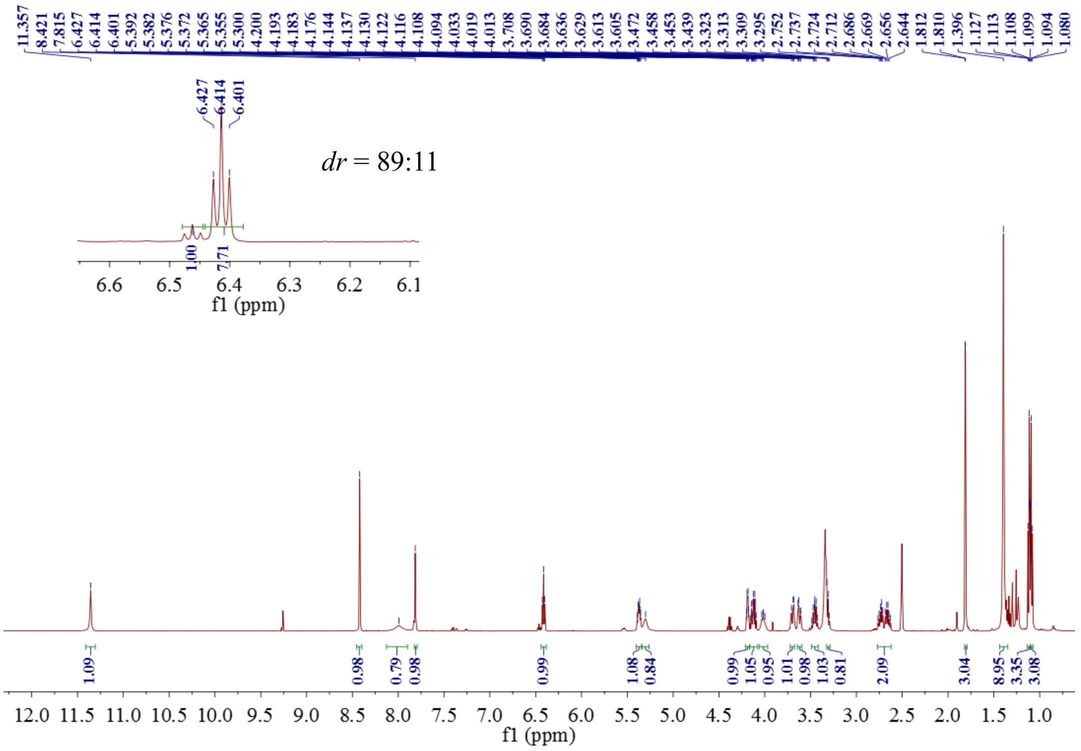

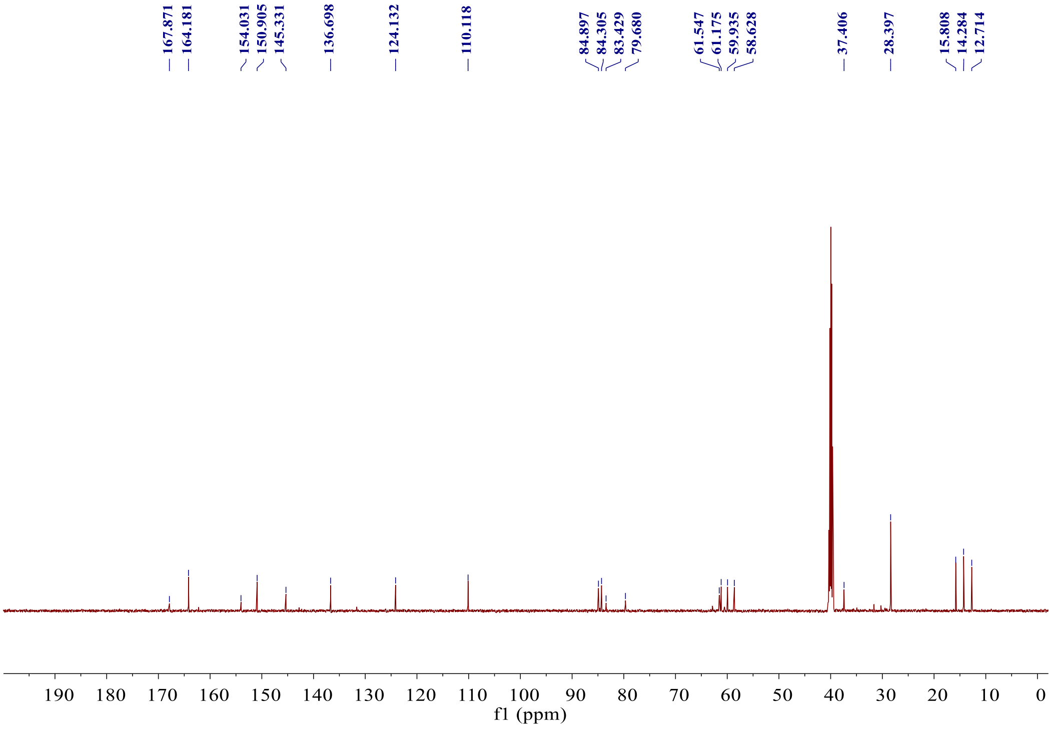

Supplementary Fig. 60. ^{1}H NMR & ^{13}C NMR spectra of compound **12** in DMSO-d_6

HPLC Data

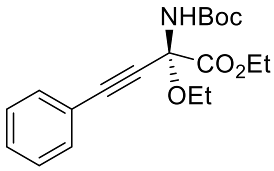

3a

Racemate:

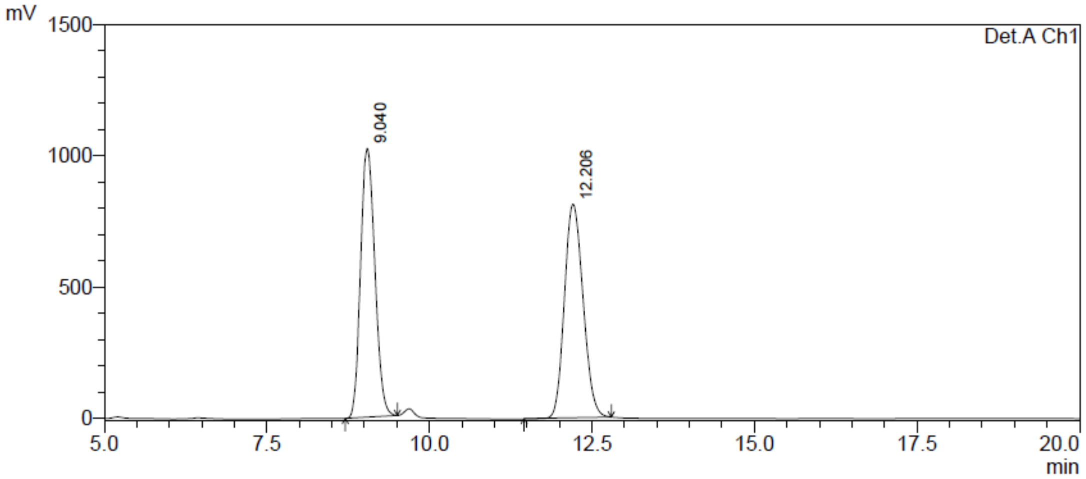

Chiral:

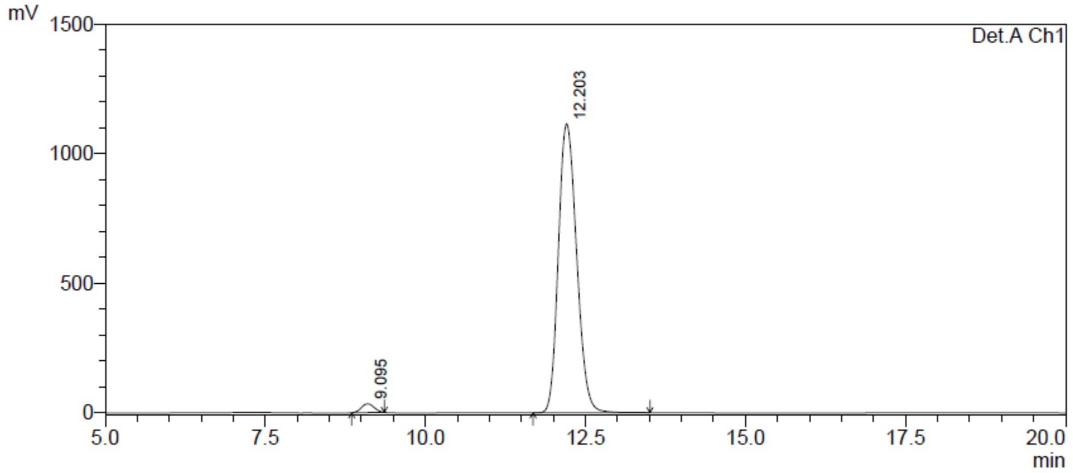

Detector A Ch1 254nm

Peak#	Ret. Time	Area	Area%
1	9.095	463630	2.053
2	12.203	22114825	97.947
Total		22578456	100.000

Supplementary Fig. 61. HPLC spectra of compound **3a**

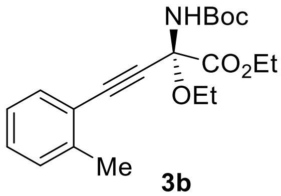

3b

Racemate:

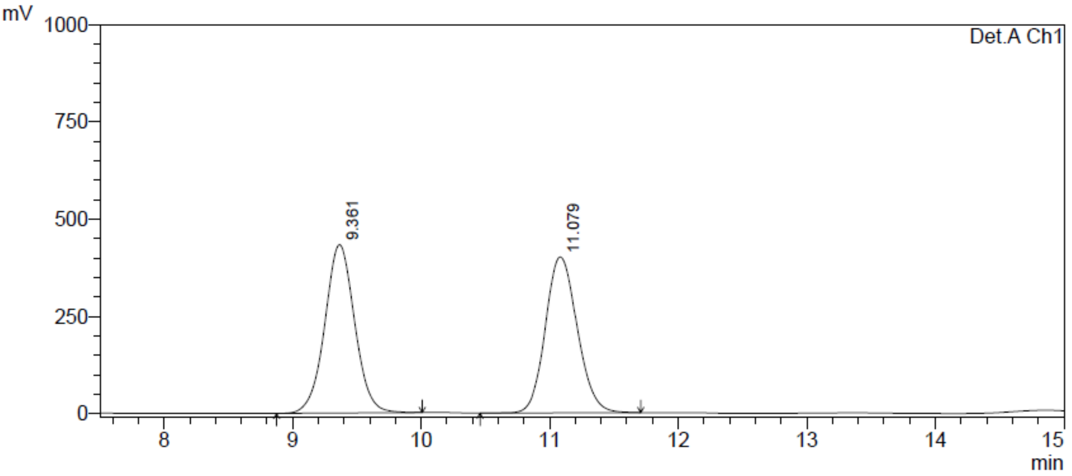

Chiral:

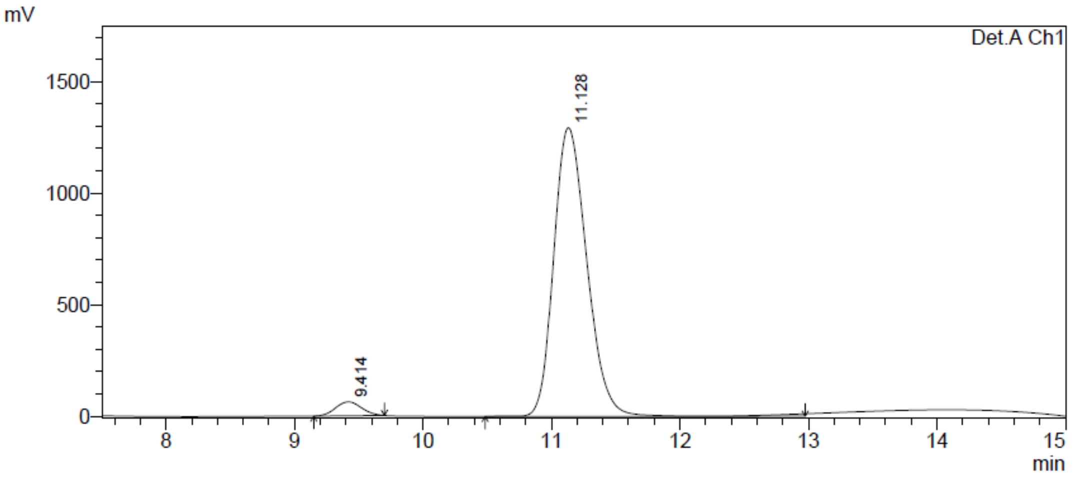

Detector A Ch1 254nm

Peak#	Ret. Time	Area	Area%
1	9.414	880306	3.668
2	11.128	23119455	96.332
Total		23999760	100.000

Supplementary Fig. 62. HPLC spectra of compound **3b**

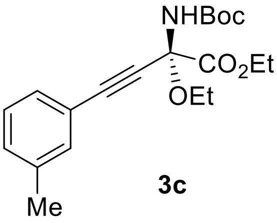

3c

Racemate:

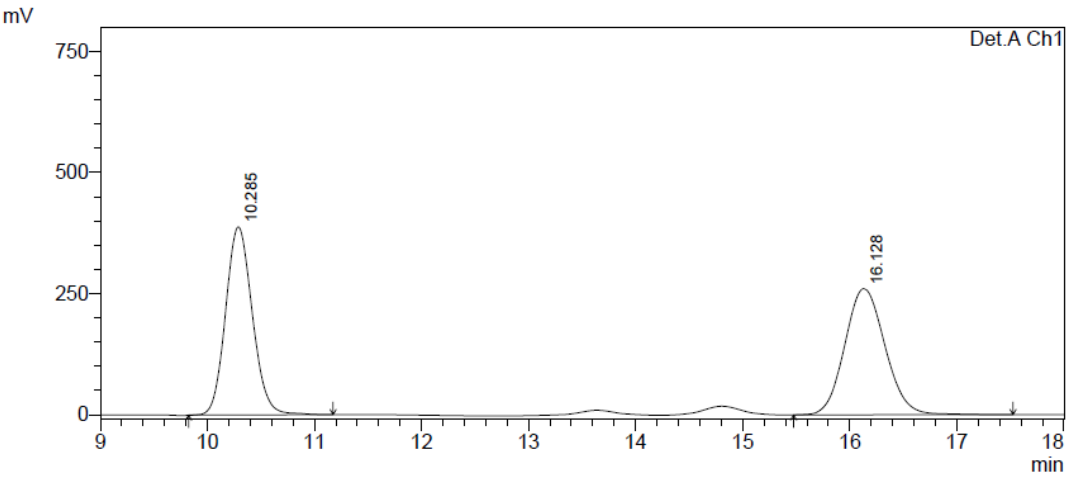

Chiral:

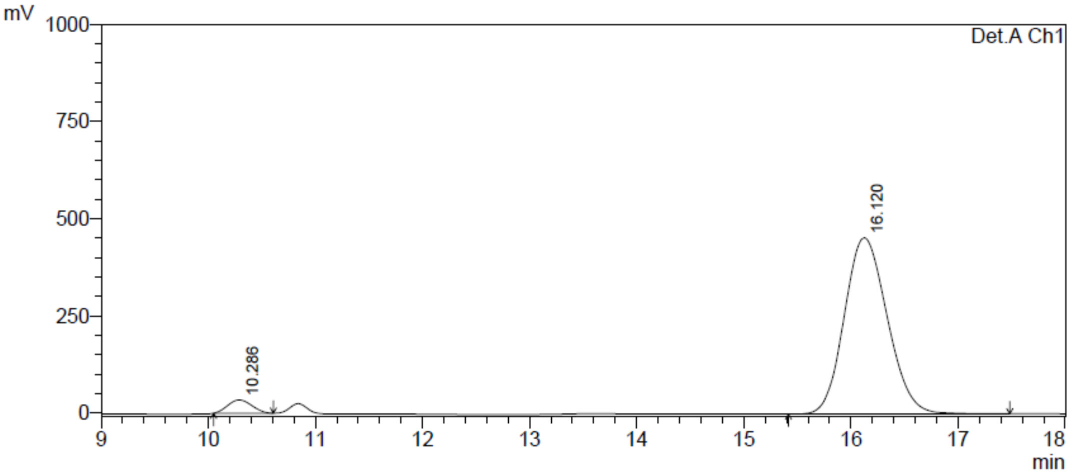

Detector A Ch1 254nm

Peak#	Ret. Time	Area	Area%
1	10.286	528798	4.017
2	16.120	12636666	95.983
Total		13165464	100.000

Supplementary Fig. 63. HPLC spectra of compound **3c**

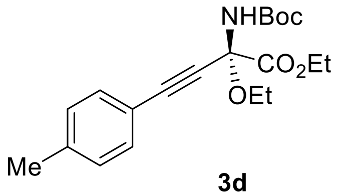

3d

Racemate:

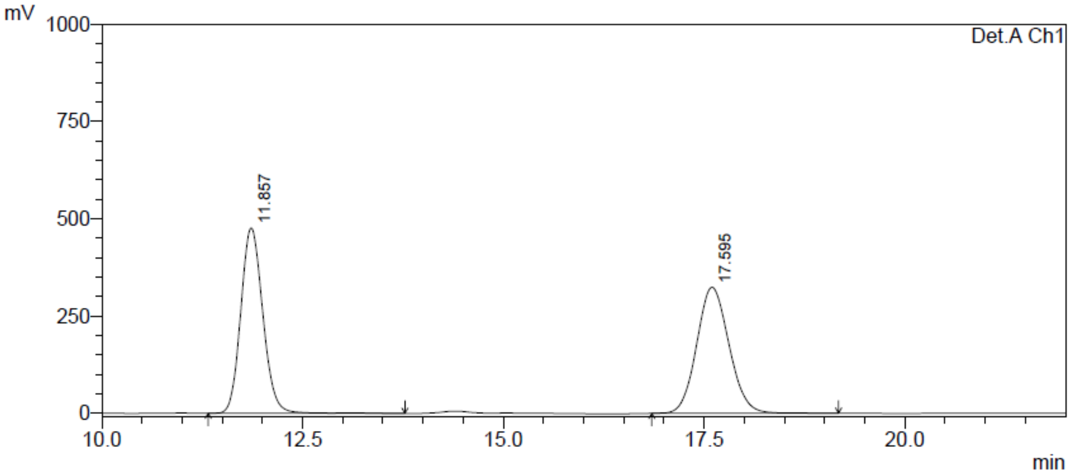

Chiral:

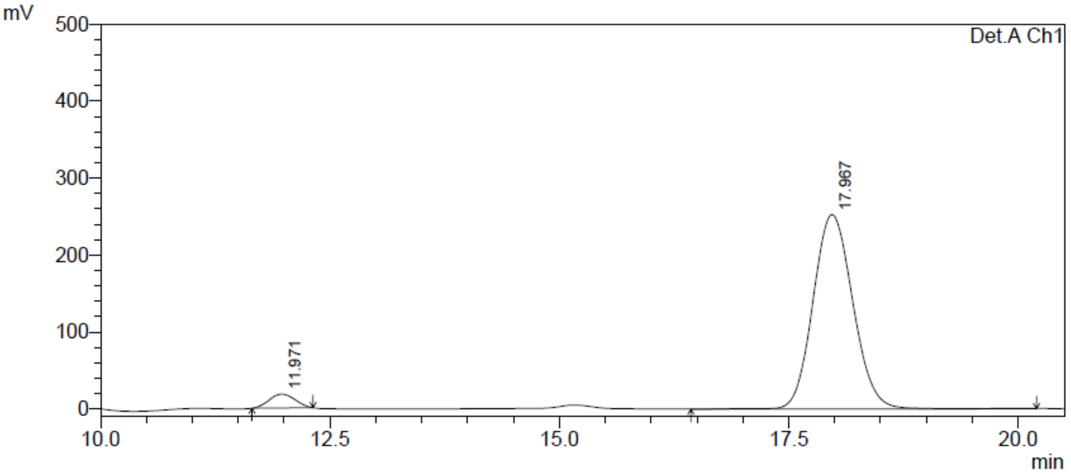

Detector A Ch1 254nm

Peak#	Ret. Time	Area	Area%
1	11.971	353760	4.435
2	17.967	7622592	95.565
Total		7976352	100.000

Supplementary Fig. 64. HPLC spectra of compound **3d**

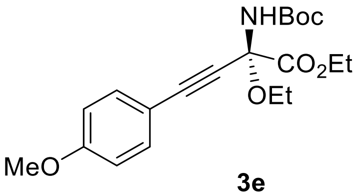

3e

Racemate:

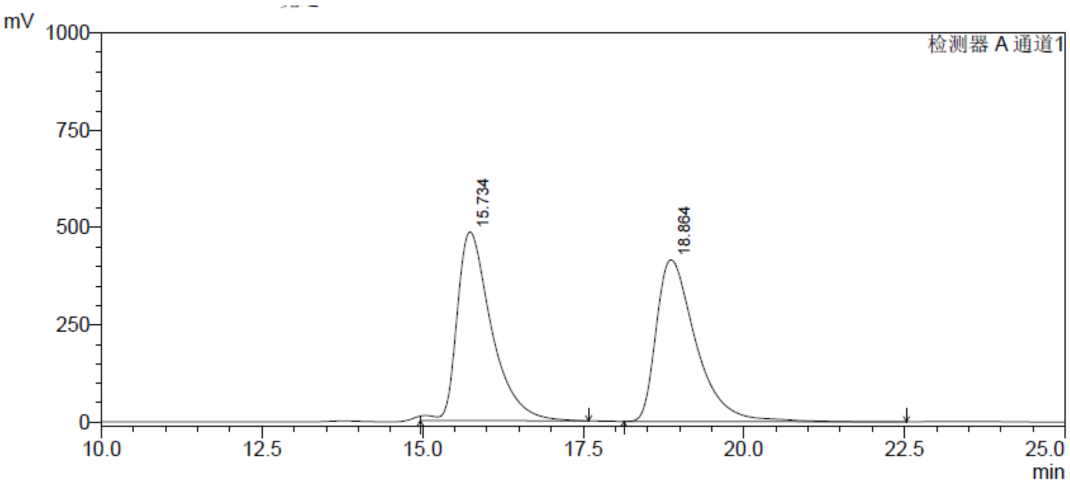

Chiral:

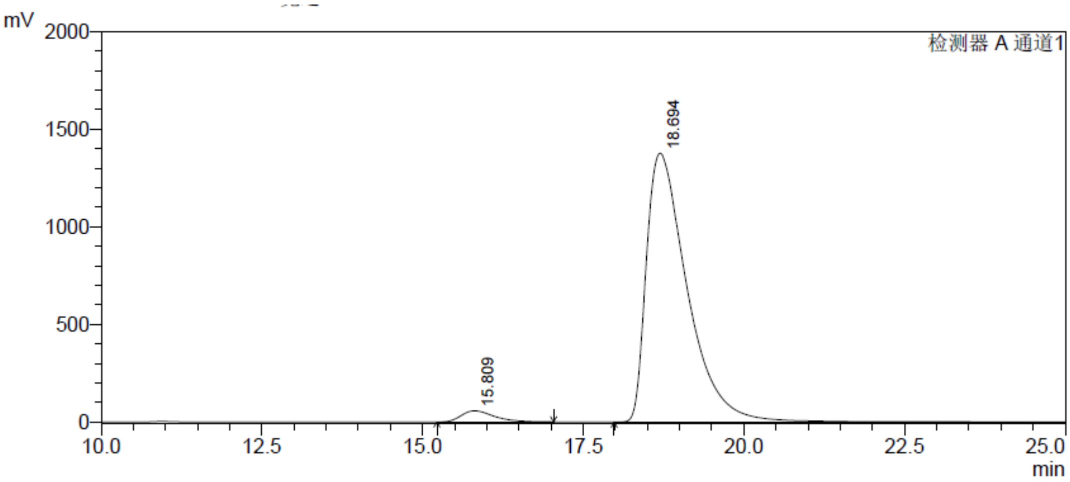

Detector A Ch1 254nm

Peak#	Ret. Time	Area	Area%
1	15.809	2030484	3.213
2	18.694	61161593	96.787
Total		63192078	100.000

Supplementary Fig. 65. HPLC spectra of compound **3e**

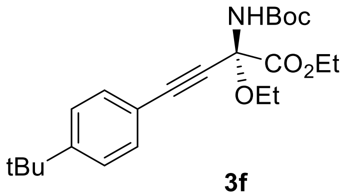

3f

Racemate:

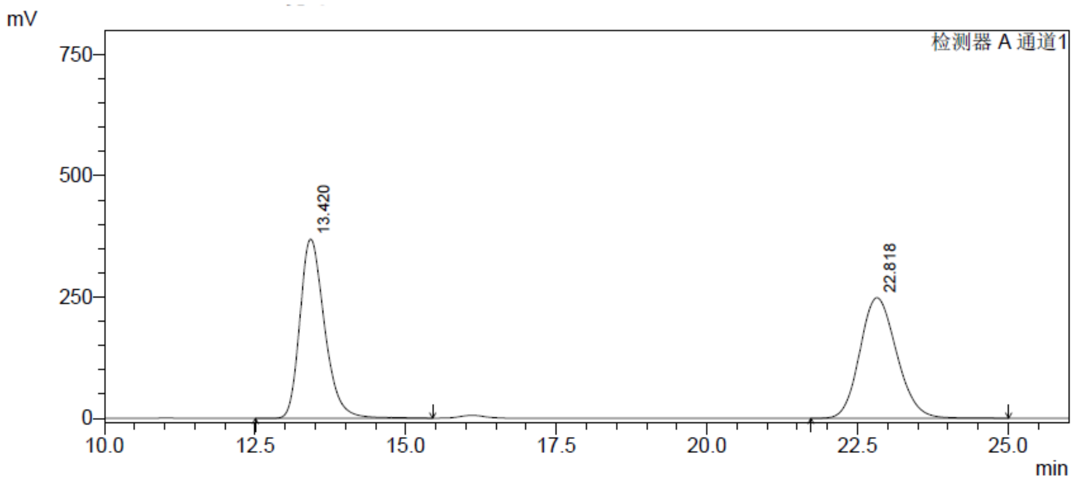

Chiral:

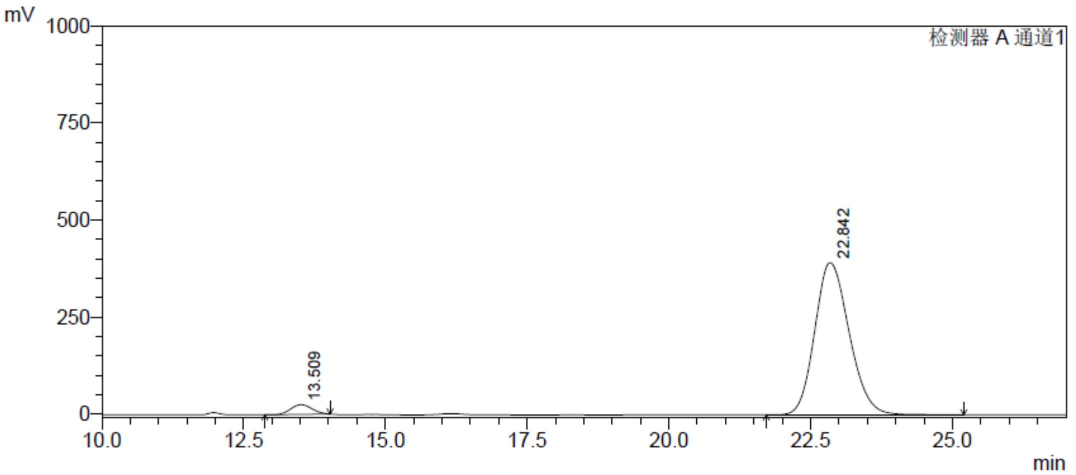

Detector A Ch1 254nm

Peak#	Ret. Time	Area	Area%
1	13.509	663486	3.848
2	22.842	16578773	96.152
Total		17242259	100.000

Supplementary Fig. 66. HPLC spectra of compound **3f**

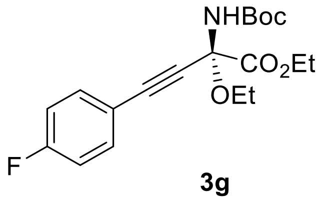

3g

Racemate:

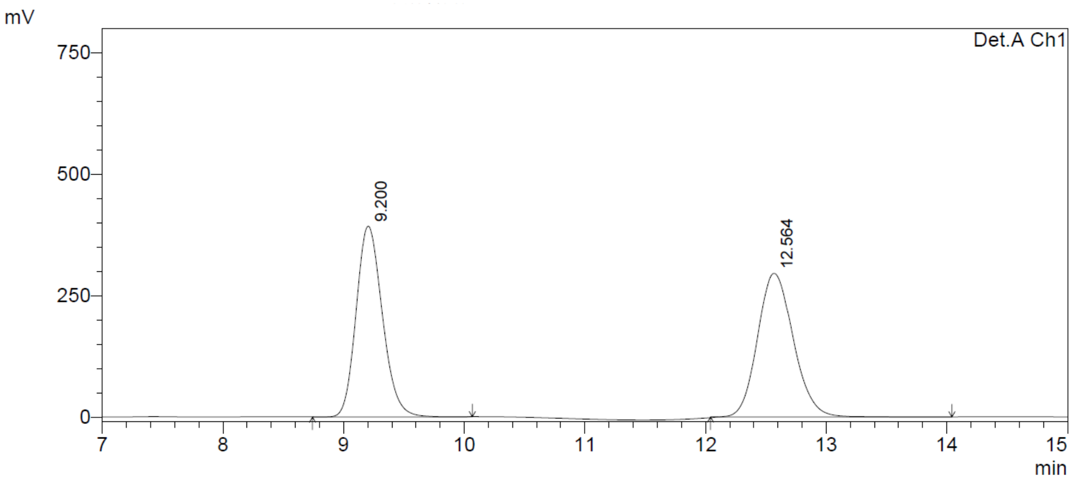

Chiral:

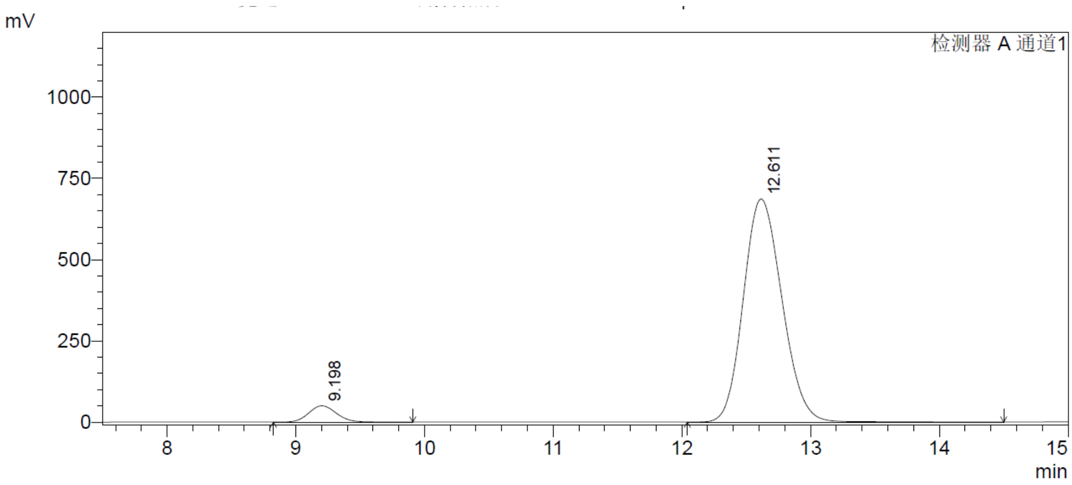

Detector A Ch1 254nm

Peak#	Ret. Time	Area	Area%
1	9.198	748104	4.959
2	12.611	14337128	95.041
Total		15085232	100.000

Supplementary Fig. 67. HPLC spectra of compound **3g**

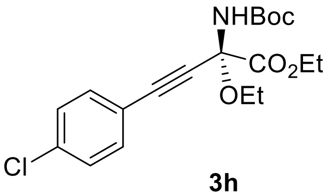

3h

Racemate:

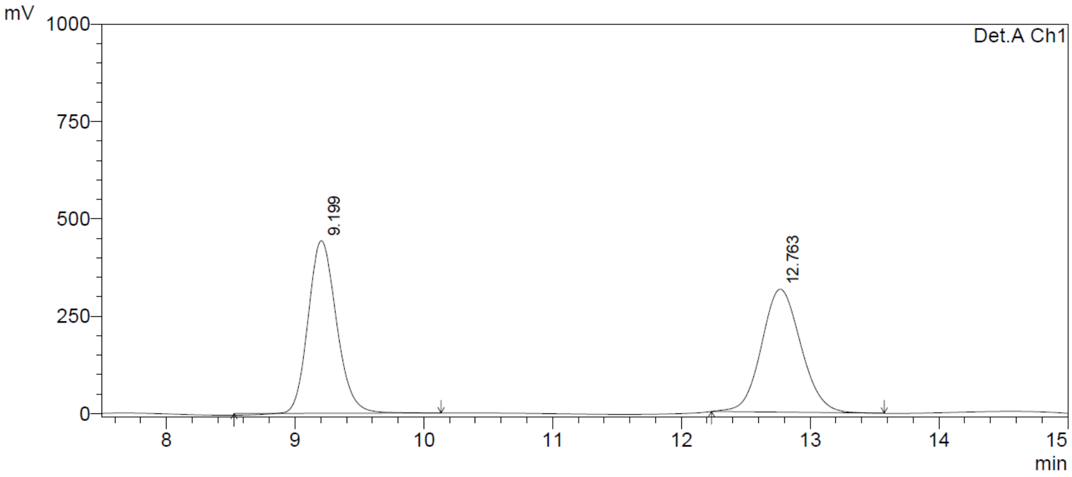

Chiral:

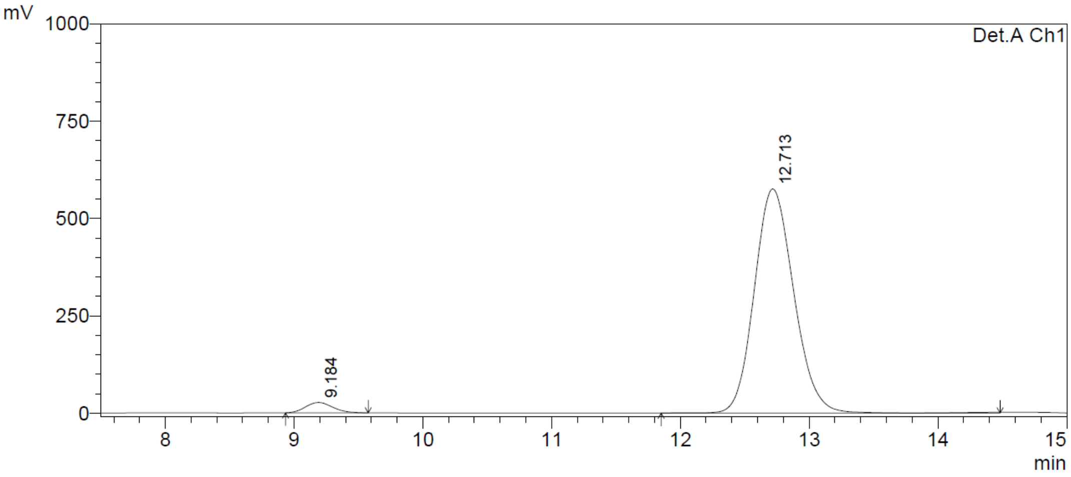

Detector A Ch1 254nm

Peak#	Ret. Time	Area	Area%
1	9.184	393963	3.113
2	12.713	12259454	96.887
Total		12653417	100.000

Supplementary Fig. 68. HPLC spectra of compound **3h**

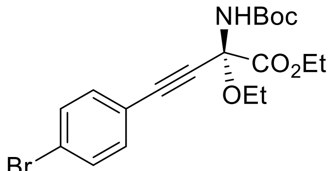

3i

Racemate:

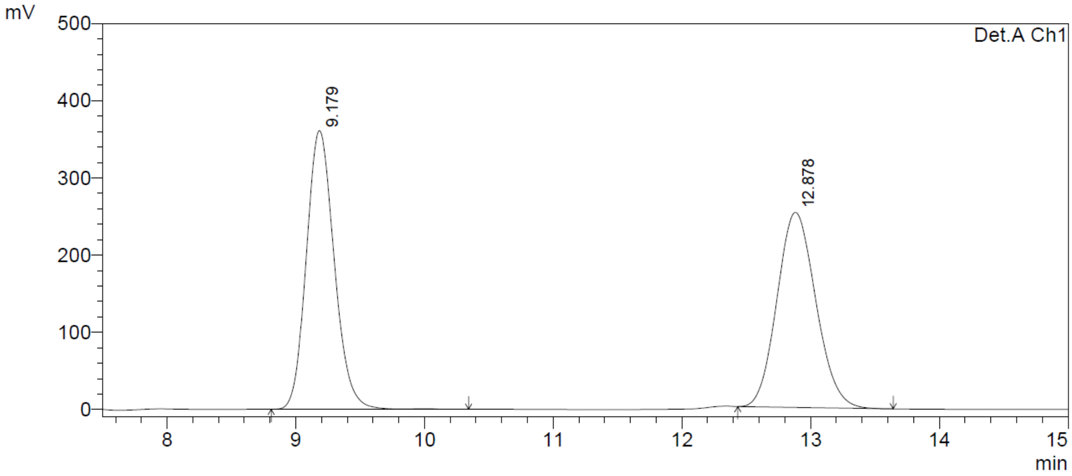

Chiral:

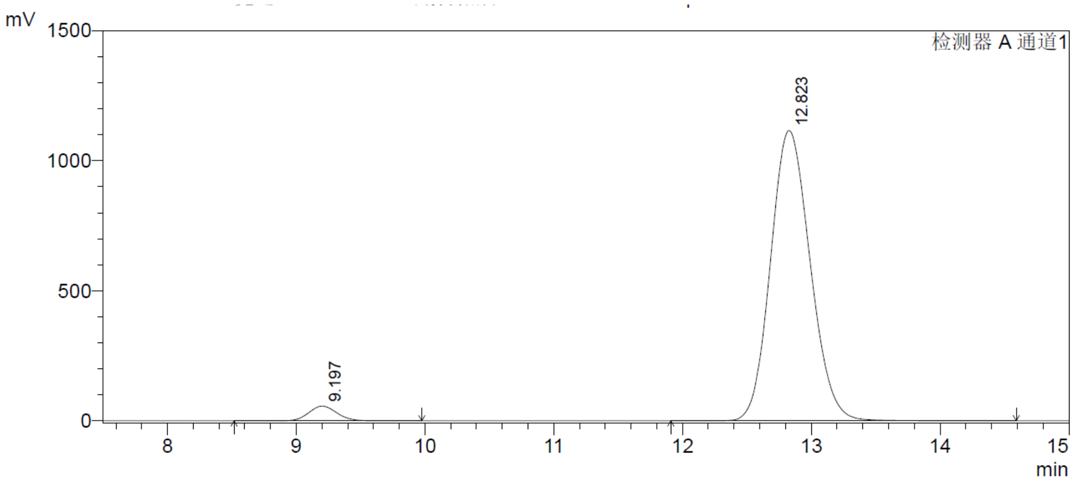

Detector A Ch1 254nm

Peak#	Ret. Time	Area	Area%
1	9.197	863433	3.492
2	12.823	23865644	96.508
Total		24729077	100.000

Supplementary Fig. 69. HPLC spectra of compound **3i**

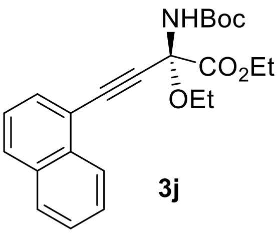

3j

Racemate:

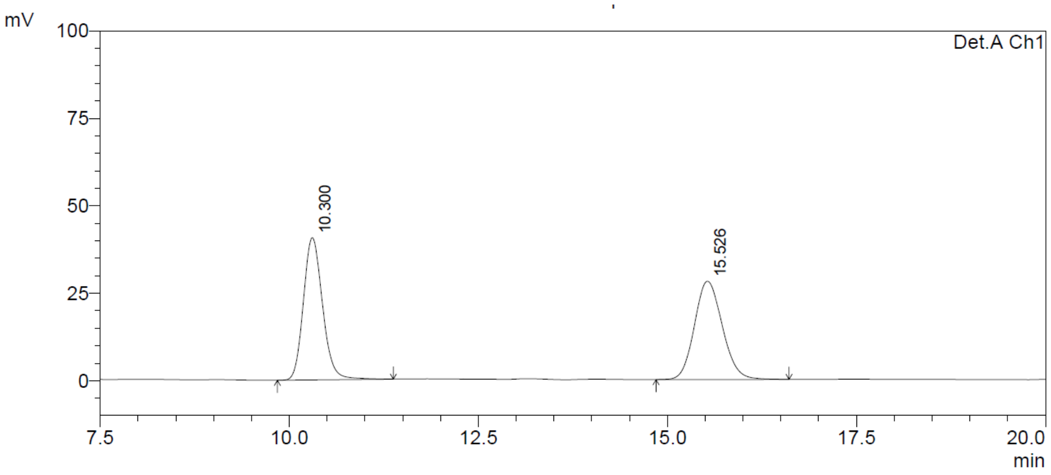

Chiral:

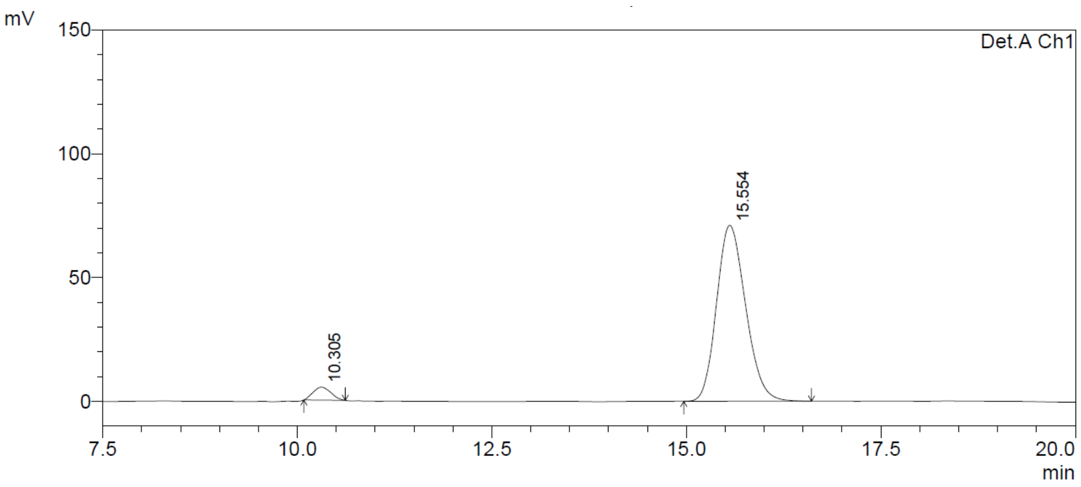

Detector A Ch1 254nm

Peak#	Ret. Time	Area	Area%
1	10.305	82722	4.288
2	15.554	1846281	95.712
Total		1929003	100.000

Supplementary Fig. 70. HPLC spectra of compound **3j**

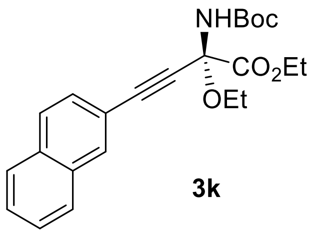

3k

Racemate:

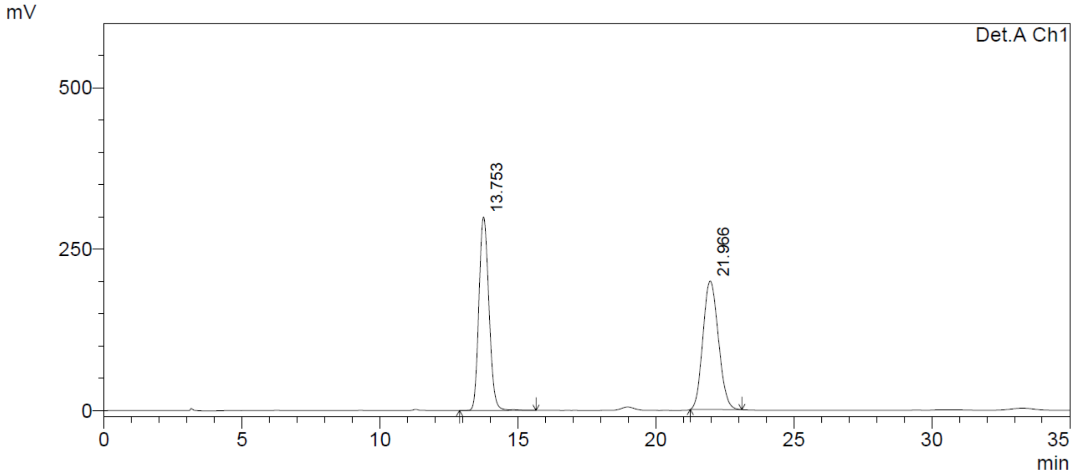

Chiral:

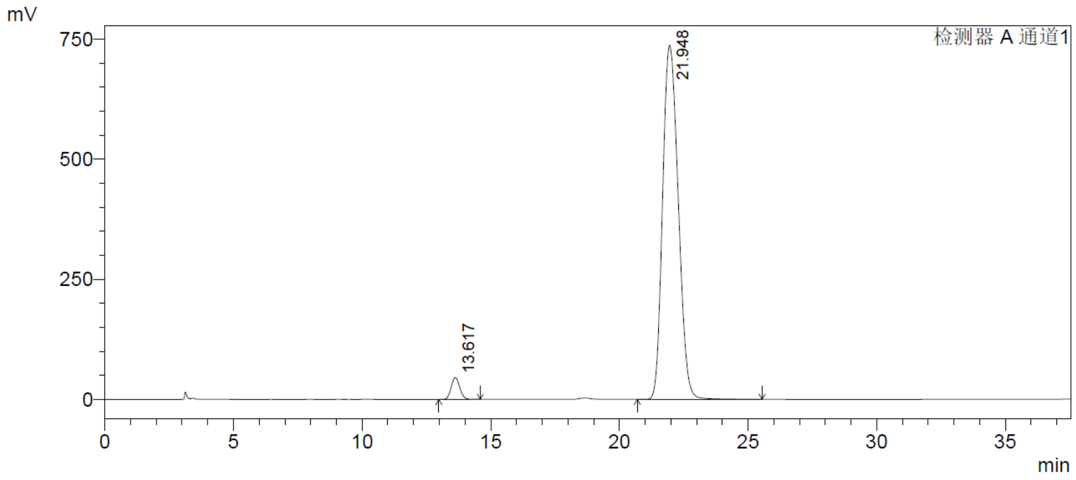

Detector A Ch1 254nm

Peak#	Ret. Time	Area	Area%
1	13.617	1055450	3.277
2	21.948	31156239	96.723
Total		32211689	100.000

Supplementary Fig. 71. HPLC spectra of compound **3k**

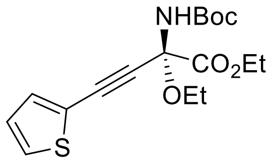

3l

Racemate:

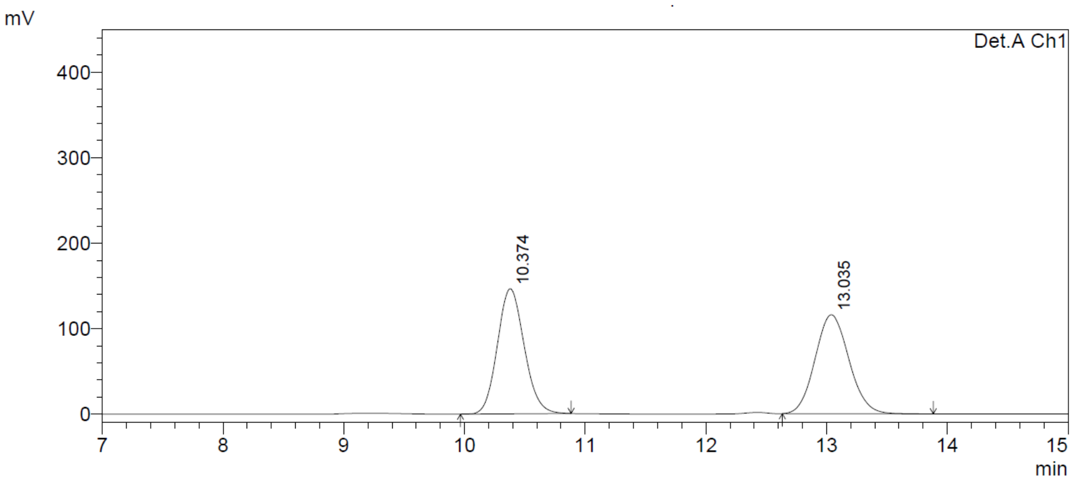

Chiral:

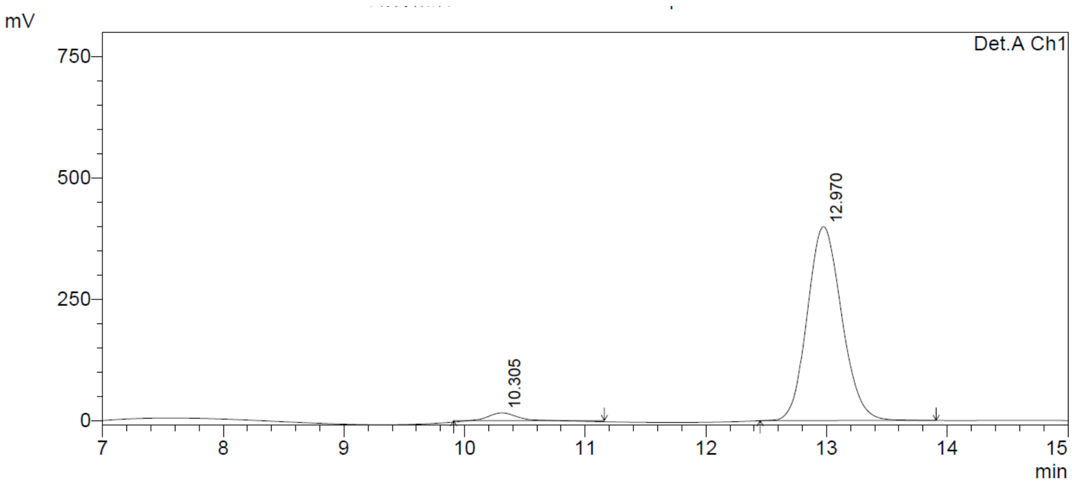

Detector A Ch1 254nm

Peak#	Ret. Time	Area	Area%
1	10.305	255005	3.125
2	12.970	7903929	96.875
Total		8158934	100.000

Supplementary Fig. 72. HPLC spectra of compound **3l**

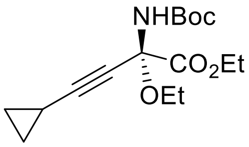

3m

Racemate:

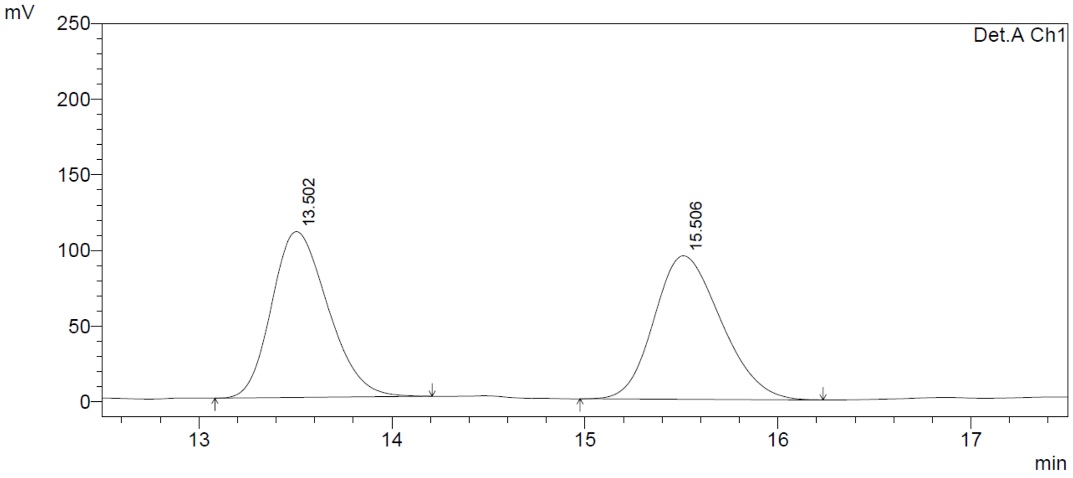

Chiral:

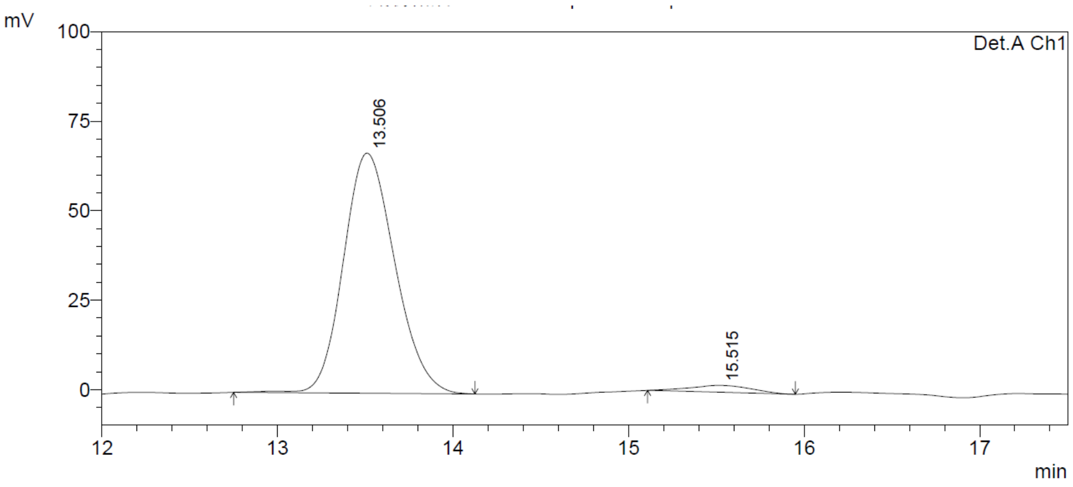

Detector A Ch1 220nm

Peak#	Ret. Time	Area	Area%
1	13.506	1369245	96.761
2	15.515	45829	3.239
Total		1415074	100.000

Supplementary Fig. 73. HPLC spectra of compound **3m**

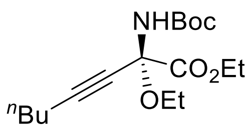

3n

Racemate:

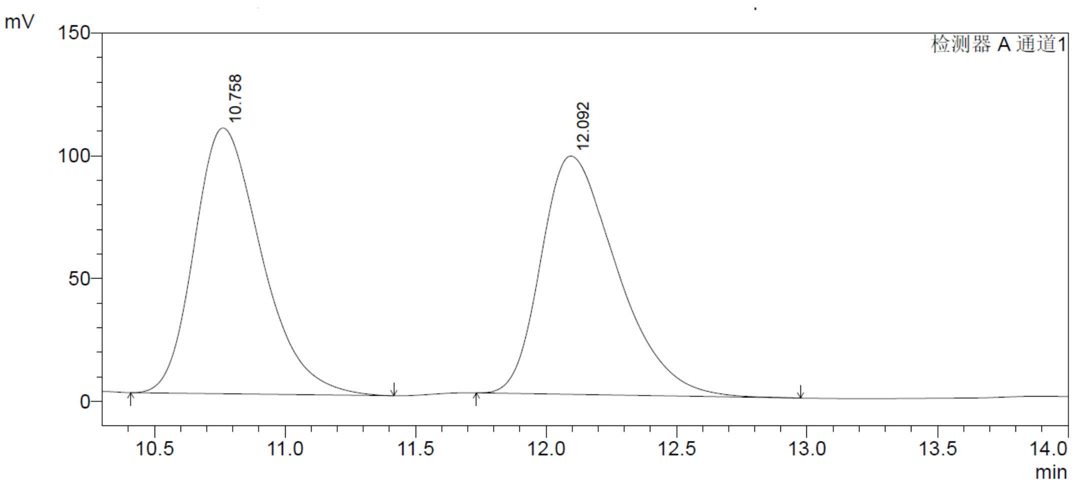

Chiral:

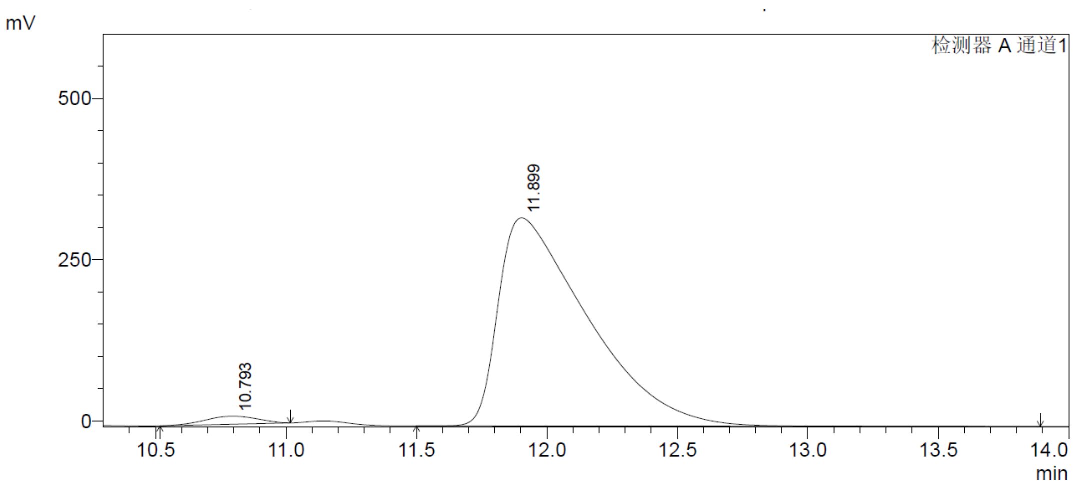

Detector A Ch1 220nm

Peak#	Ret. Time	Area	Area%
1	10.793	173966	2.211
2	11.899	7693667	97.789
Total		7867633	100.000

Supplementary Fig. 74. HPLC spectra of compound **3n**

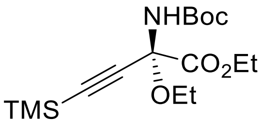

3o

Racemate:

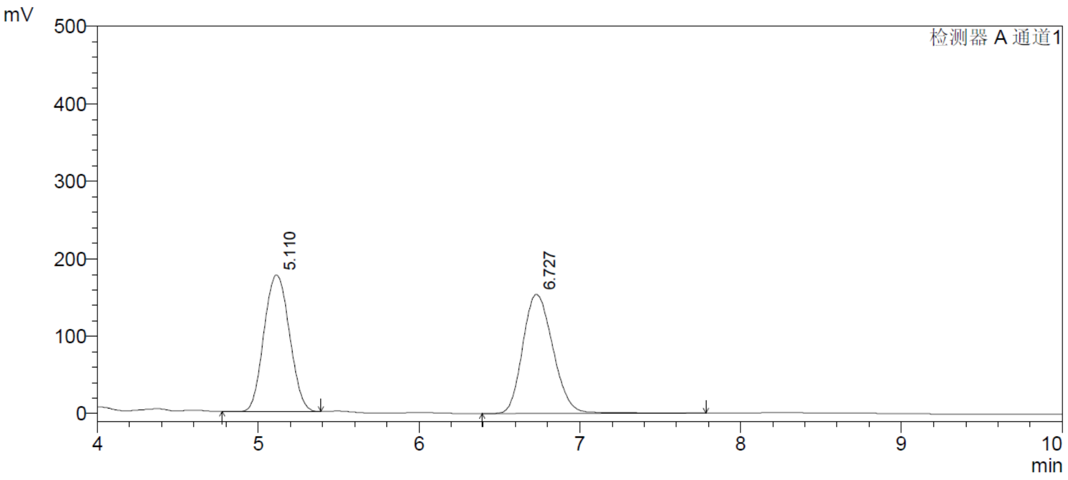

Chiral:

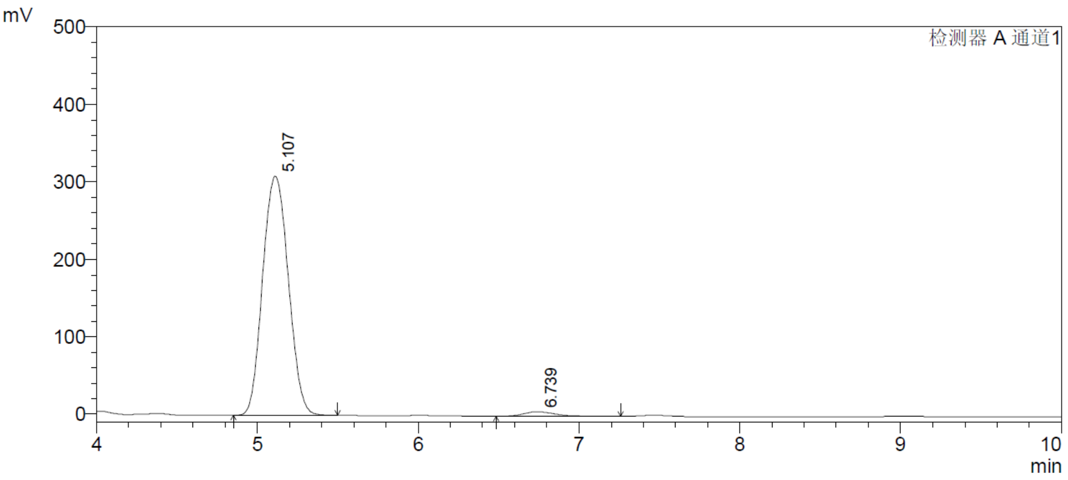

Detector A Ch1 220nm

Peak#	Ret. Time	Area	Area%
1	5.107	3427823	97.702
2	6.739	80623	2.298
Total		3508446	100.000

Supplementary Fig. 75. HPLC spectra of compound **3o**

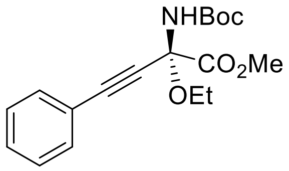

3p

Racemate:

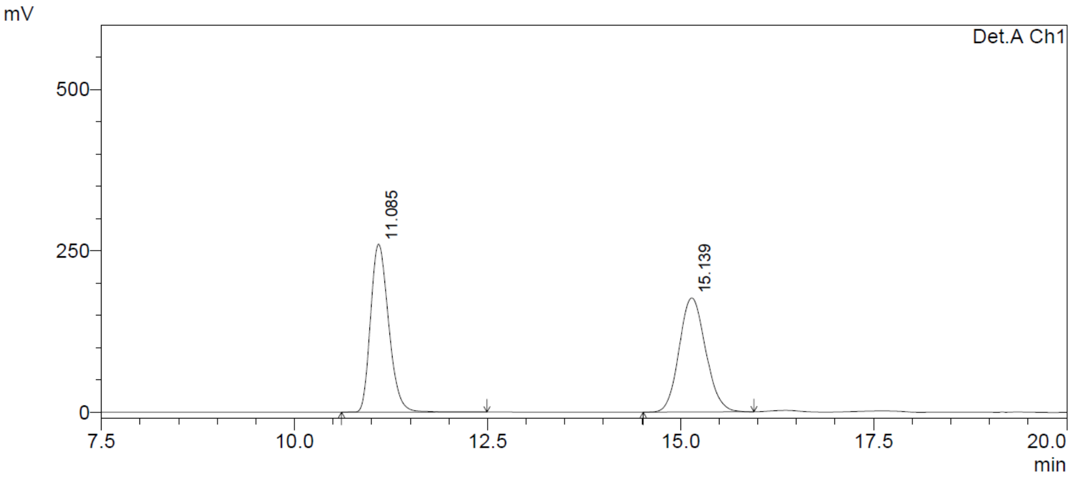

Chiral:

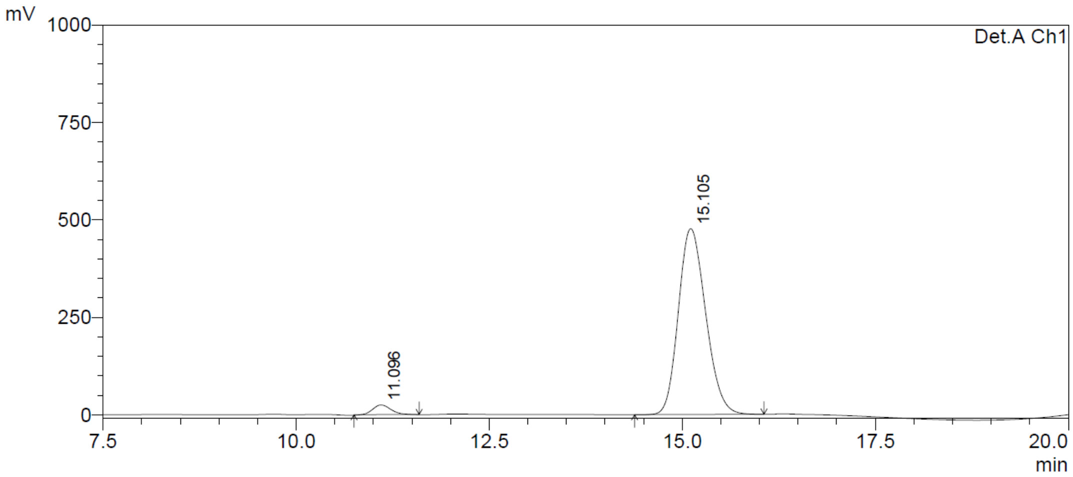

Detector A Ch1 254nm

Peak#	Ret. Time	Area	Area%
1	11.096	399922	3.293
2	15.105	11746515	96.707
Total		12146438	100.000

Supplementary Fig. 76. HPLC spectra of compound **3p**

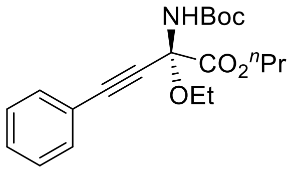

3q

Racemate:

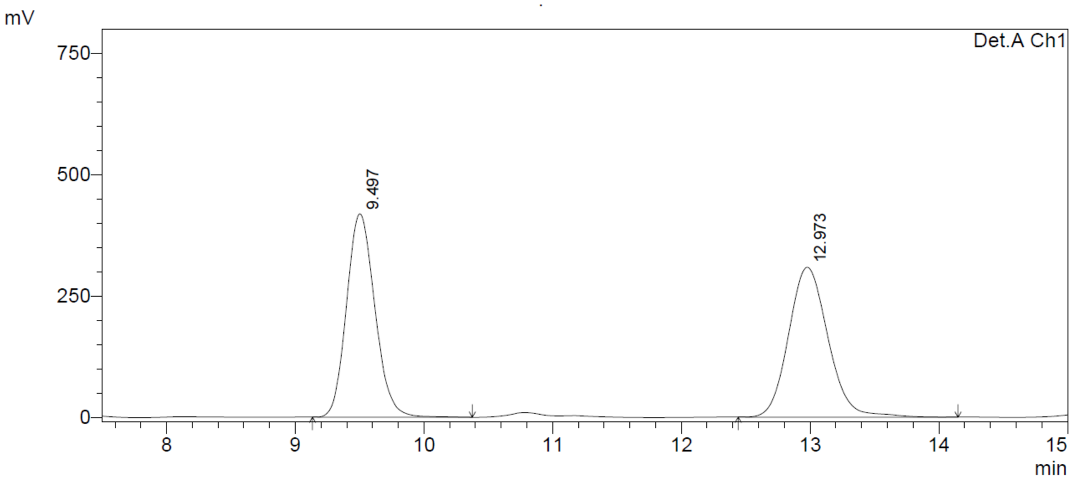

Chiral:

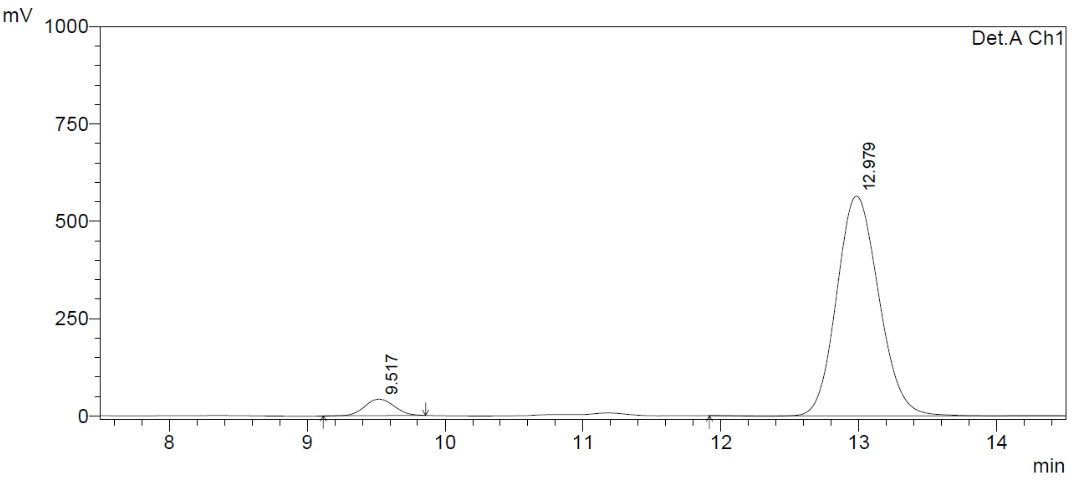

Detector A Ch1 254nm

Peak#	Ret. Time	Area	Area%
1	9.517	630384	5.000
2	12.979	11976632	95.000
Total		12607016	100.000

Supplementary Fig. 77. HPLC spectra of compound **3q**

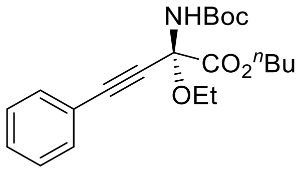

3r

Racemate:

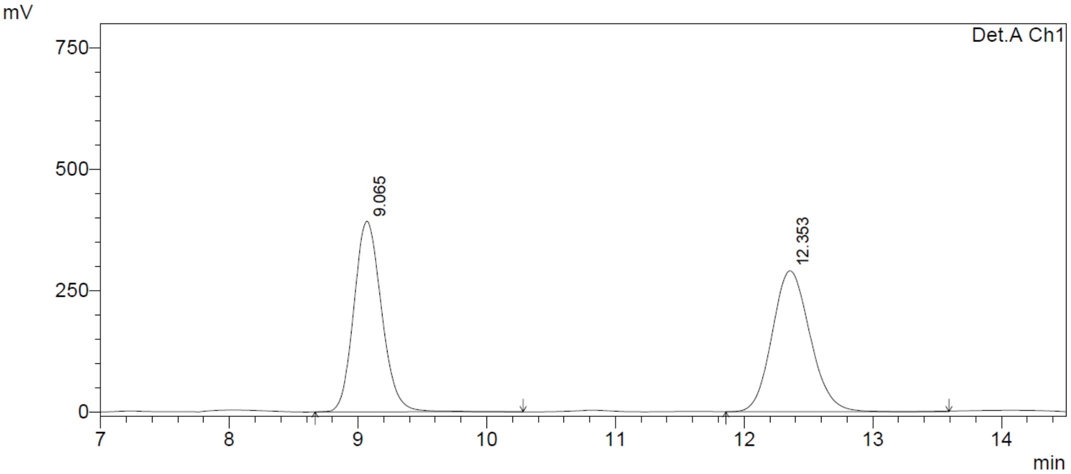

Chiral:

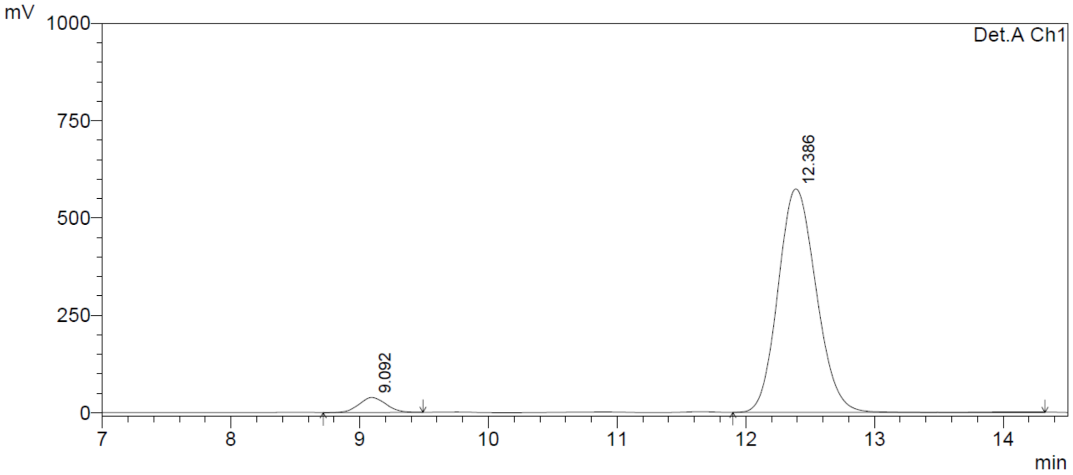

Detector A Ch1 254nm

Peak#	Ret. Time	Area	Area%
1	9.092	560861	4.522
2	12.386	11843051	95.478
Total		12403911	100.000

Supplementary Fig. 78. HPLC spectra of compound **3r**

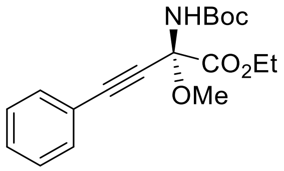

3s

Racemate:

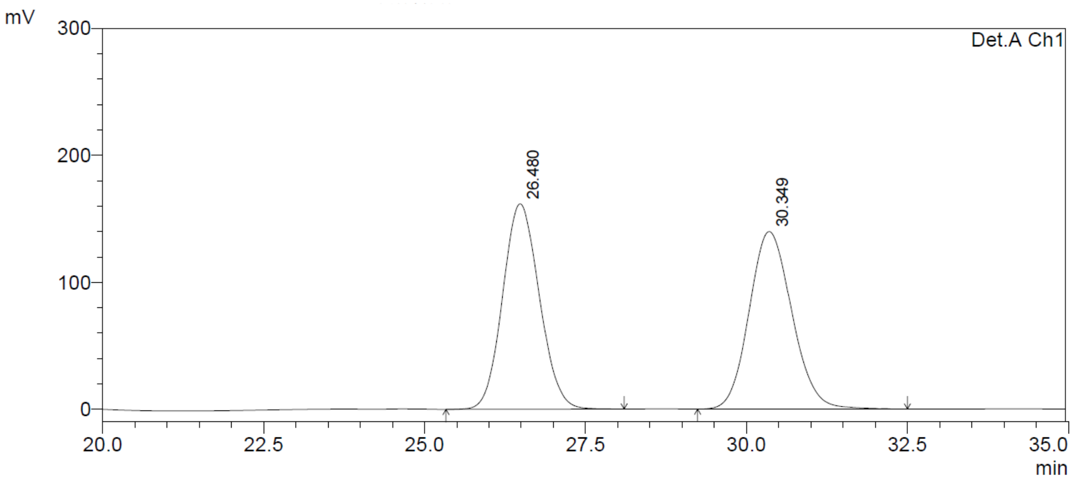

Chiral:

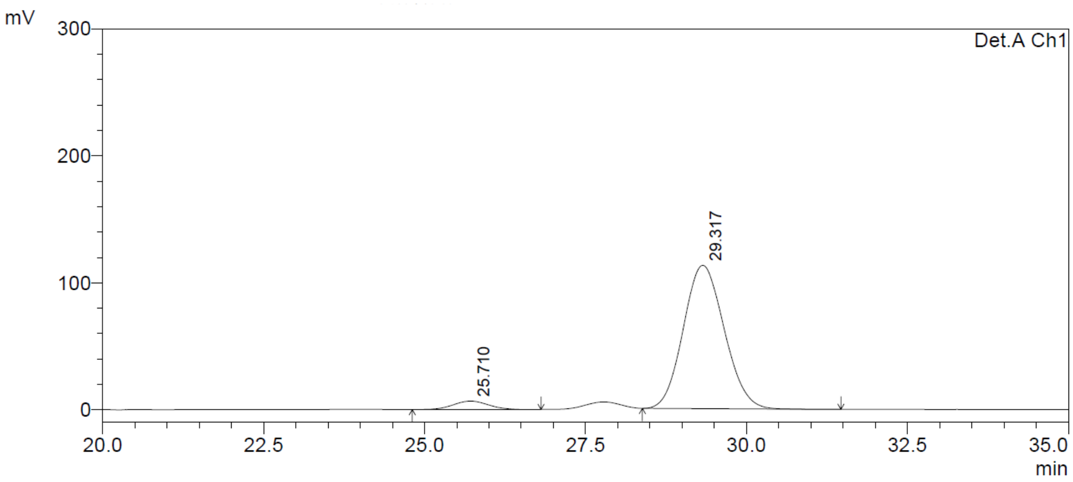

Detector A Ch1 254nm

Peak#	Ret. Time	Area	Area%
1	25.710	253987	4.837
2	29.317	4997412	95.163
Total		5251399	100.000

Supplementary Fig. 79. HPLC spectra of compound **3s**

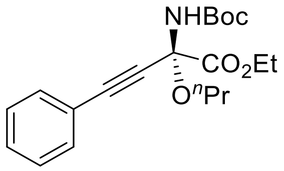

3t

Racemate:

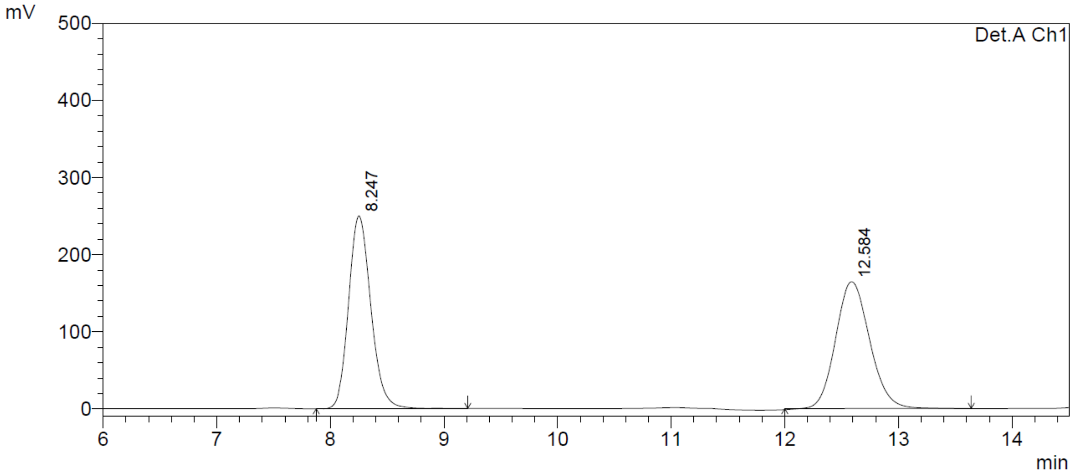

Chiral:

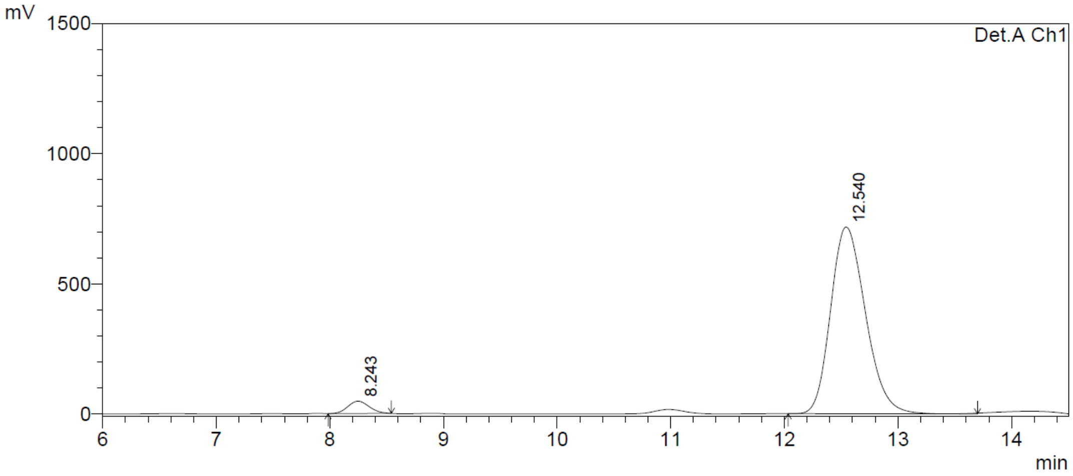

Detector A Ch1 254nm

Peak#	Ret. Time	Area	Area%
1	8.243	619907	3.890
2	12.540	15318039	96.110
Total		15937946	100.000

Supplementary Fig. 80. HPLC spectra of compound **3t**

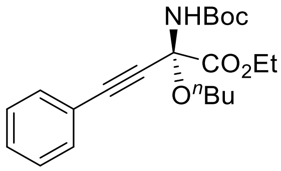

3u

Racemate:

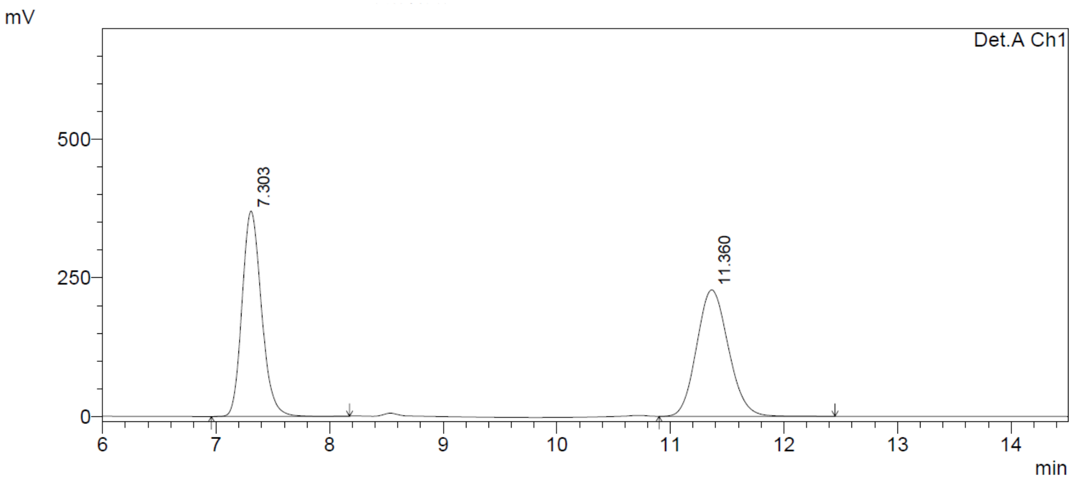

Chiral:

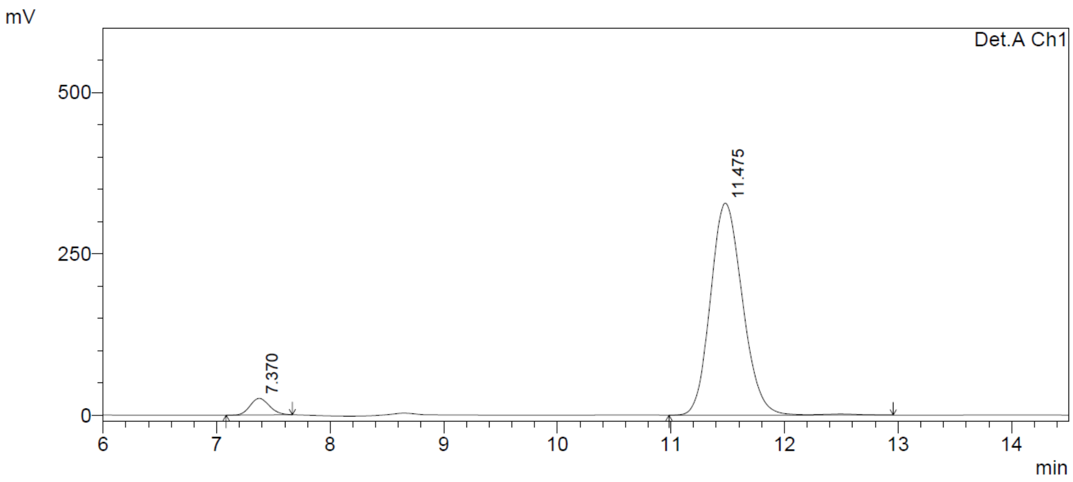

Detector A Ch1 254nm

Peak#	Ret. Time	Area	Area%
1	7.370	304135	4.493
2	11.475	6465402	95.507
Total		6769537	100.000

Supplementary Fig. 81. HPLC spectra of compound **3u**

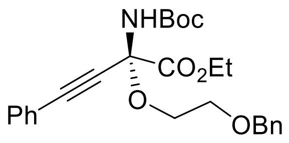

3v

Racemate:

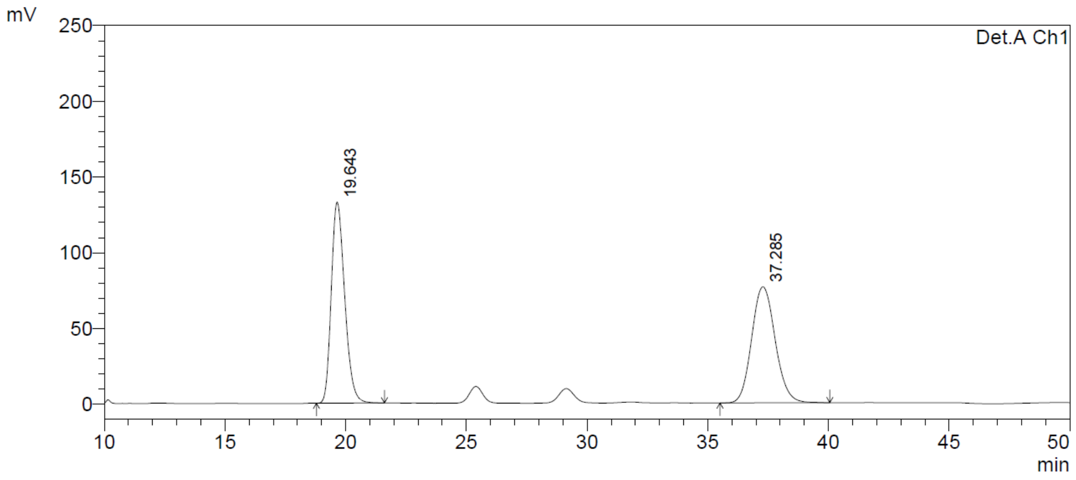

Chiral:

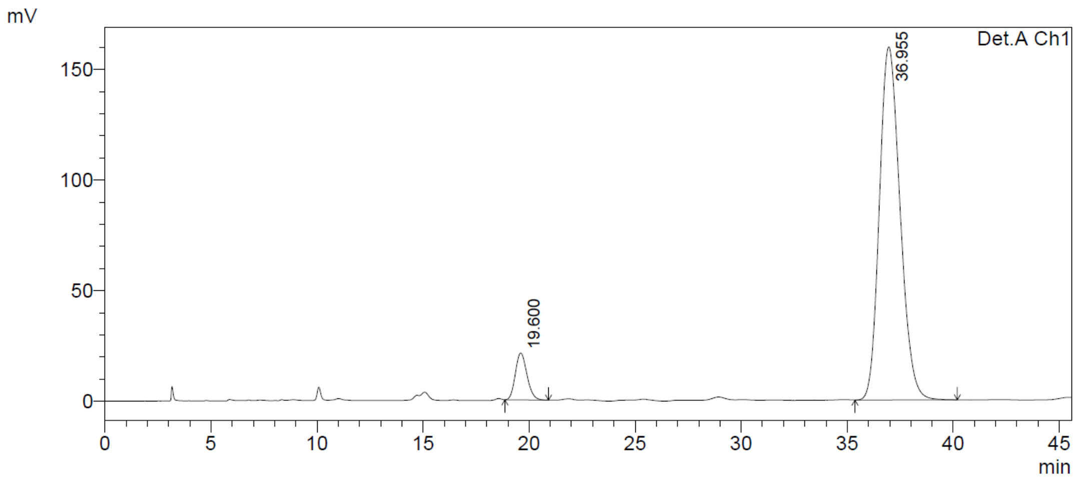

Detector A Ch1 254nm

Peak#	Ret. Time	Area	Area%
1	19.600	810642	6.954
2	36.955	10847329	93.046
Total		11657971	100.000

Supplementary Fig. 82. HPLC spectra of compound **3v**

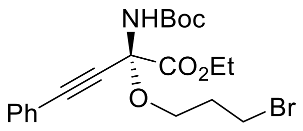

3w

Racemate:

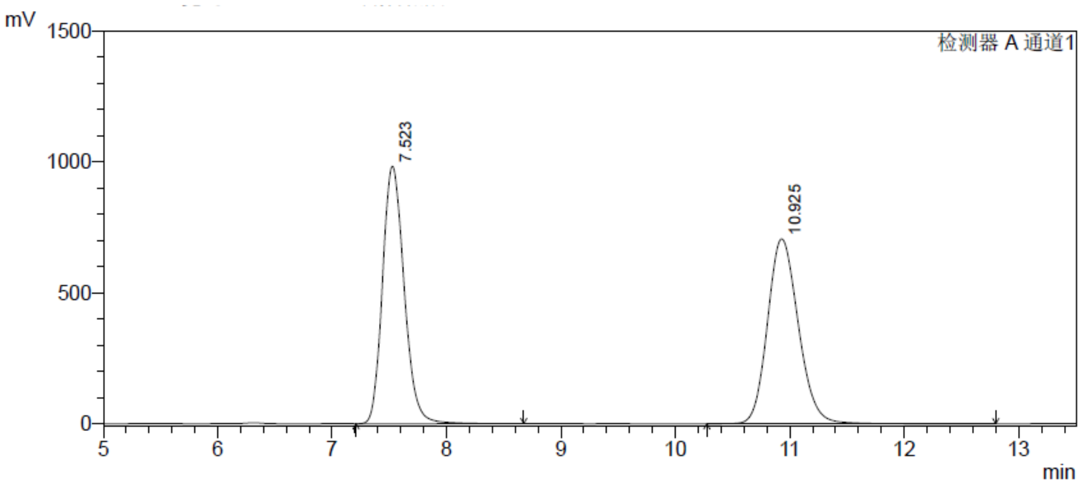

Chiral:

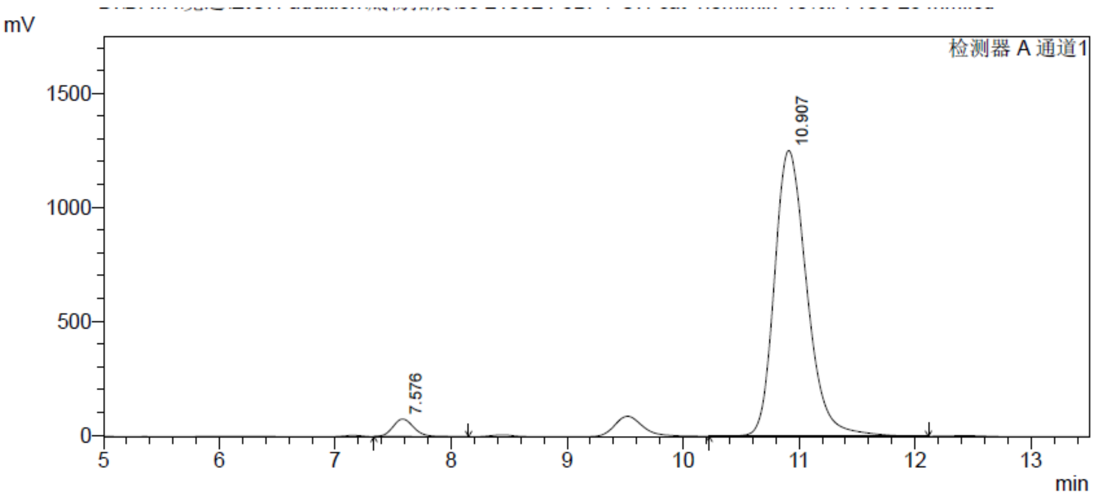

Detector A Ch1 254nm

Peak#	Ret. Time	Area	Area%
1	7.576	947619	3.733
2	10.907	24438096	96.267
Total		25385715	100.000

Supplementary Fig. 83. HPLC spectra of compound **3w**

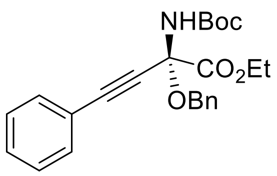

3x

Racemate:

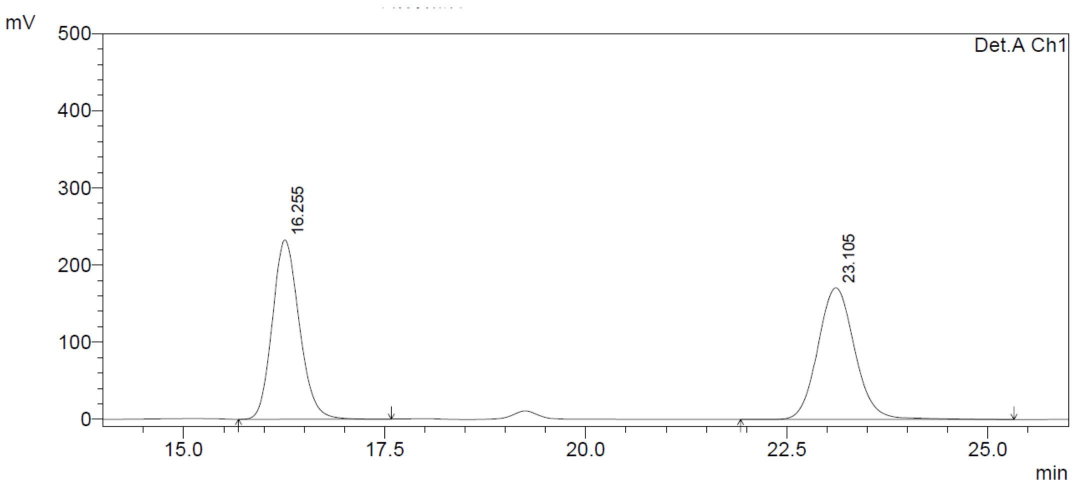

Chiral:

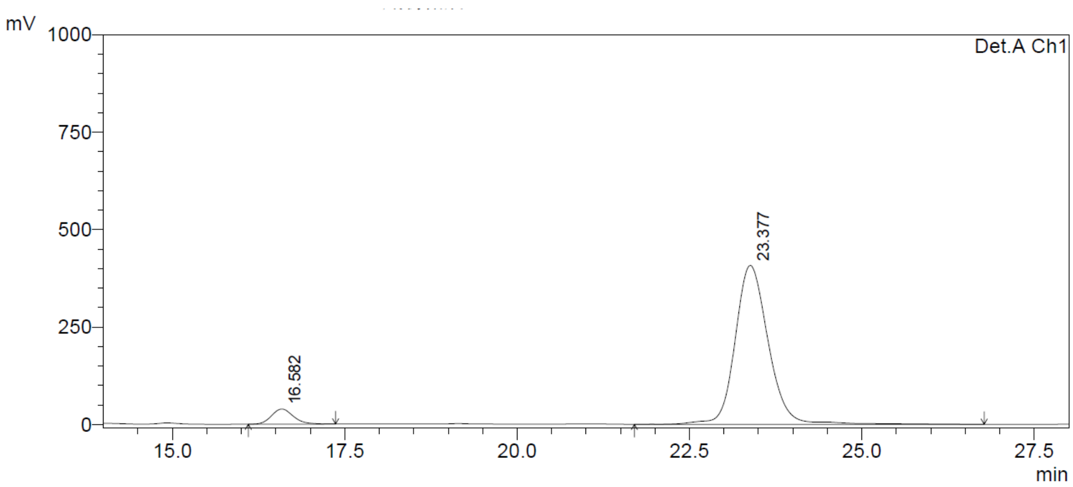

Detector A Ch1 254nm

Peak#	Ret. Time	Area	Area%
1	16.582	854280	5.885
2	23.377	13662014	94.115
Total		14516295	100.000

Supplementary Fig. 84. HPLC spectra of compound **3x**

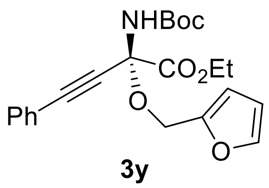

3y

Racemate：

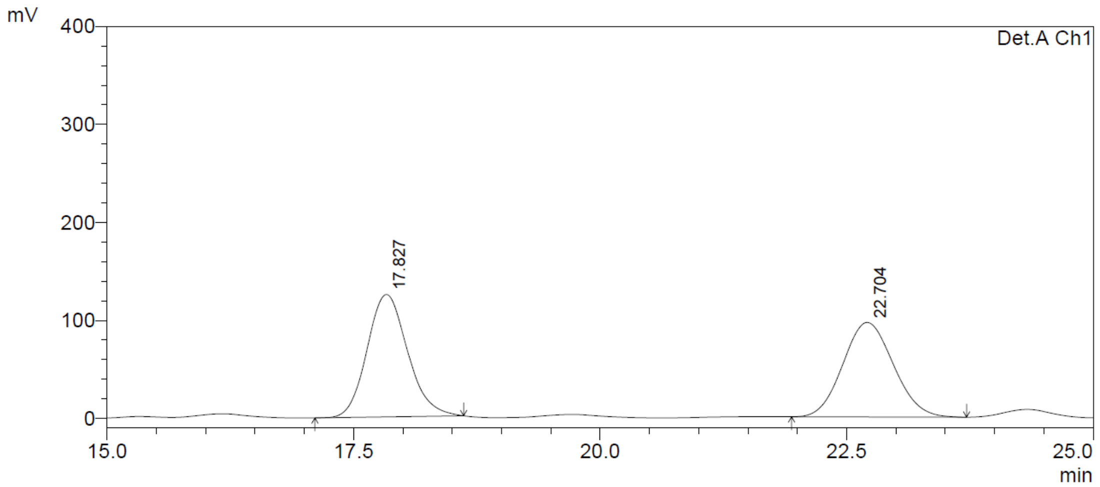

Chiral:

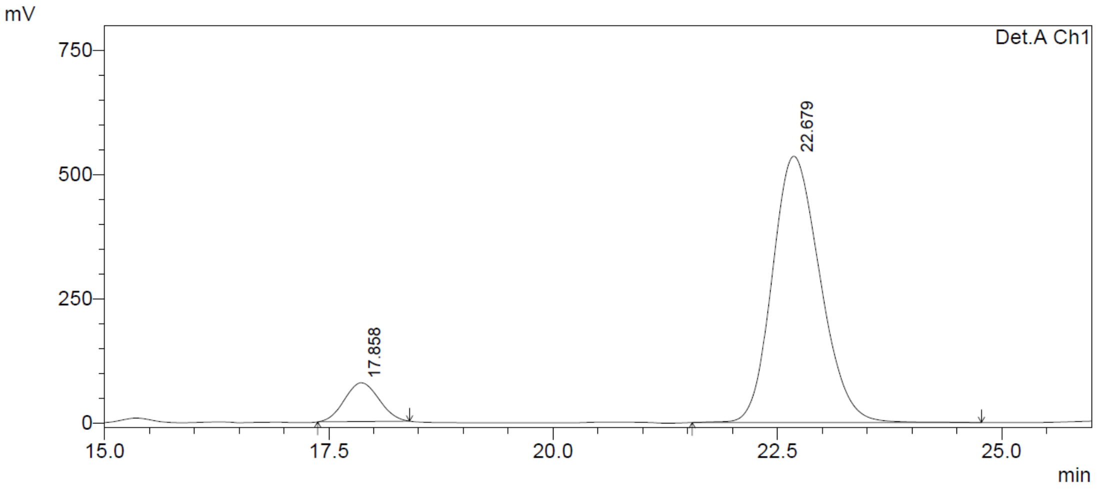

Detector A Ch1 254nm

Peak#	Ret. Time	Area	Area%
1	17.858	2093889	9.528
2	22.679	19883237	90.472
Total		21977126	100.000

Supplementary Fig. 85. HPLC spectra of compound **3y**

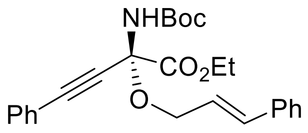

3z

Racemate:

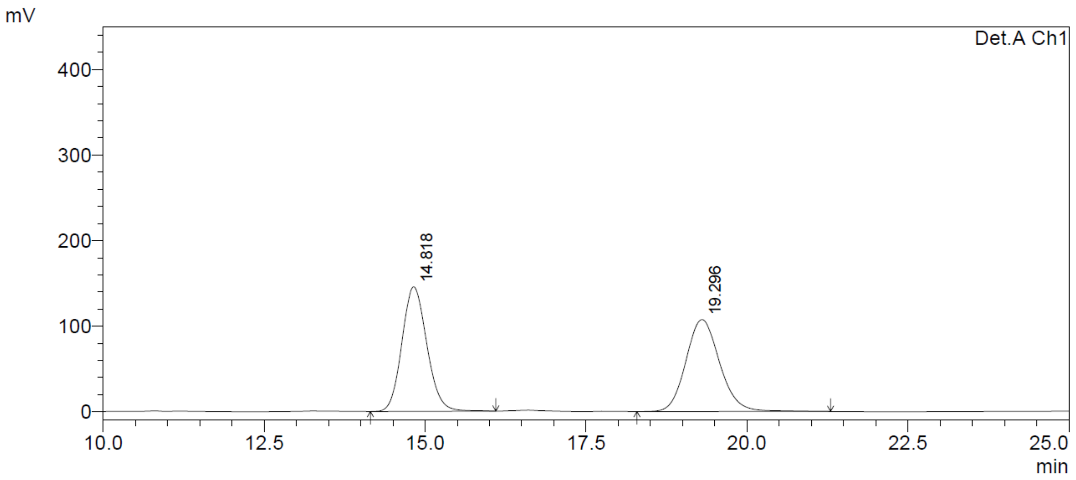

Chiral:

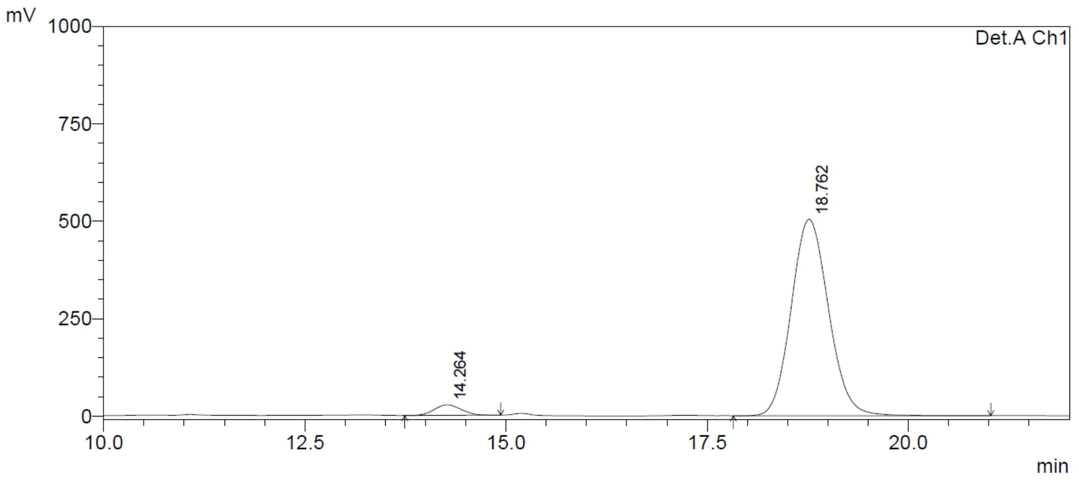

Detector A Ch1 254nm

Peak#	Ret. Time	Area	Area%
1	14.264	624278	3.683
2	18.762	16324531	96.317
Total		16948809	100.000

Supplementary Fig. 86. HPLC spectra of compound **3z**

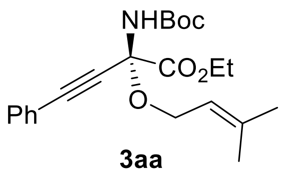

3aa

Racemate:

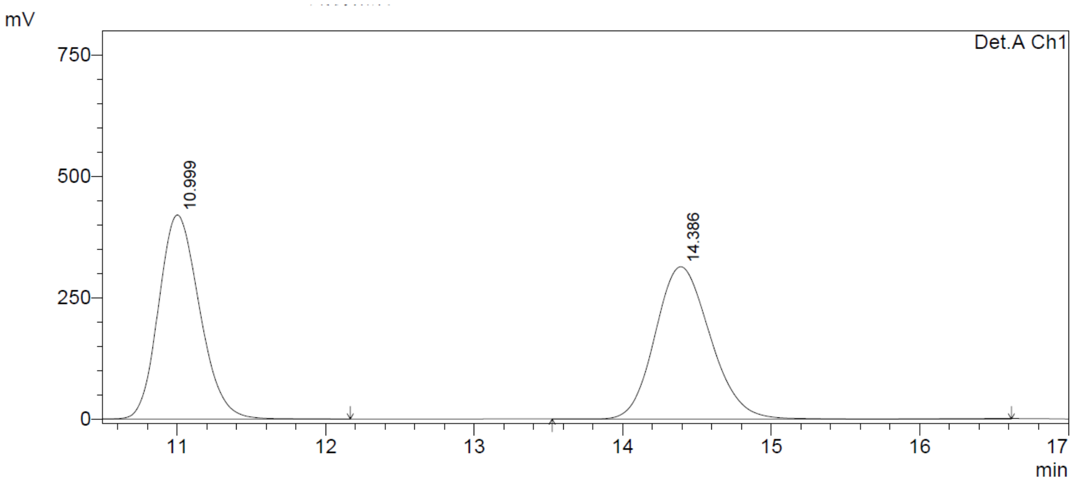

Chiral:

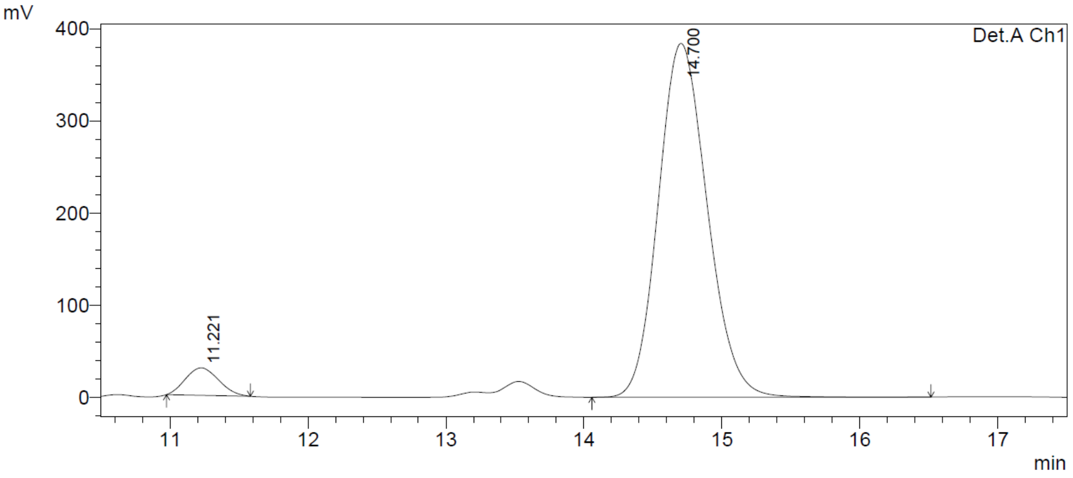

Detector A Ch1 254nm

Peak#	Ret. Time	Area	Area%
1	11.221	495980	4.976
2	14.700	9470679	95.024
Total		9966659	100.000

Supplementary Fig. 87. HPLC spectra of compound **3aa**

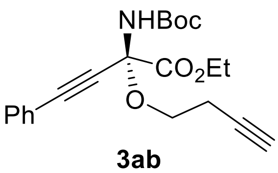

3ab

Racemate:

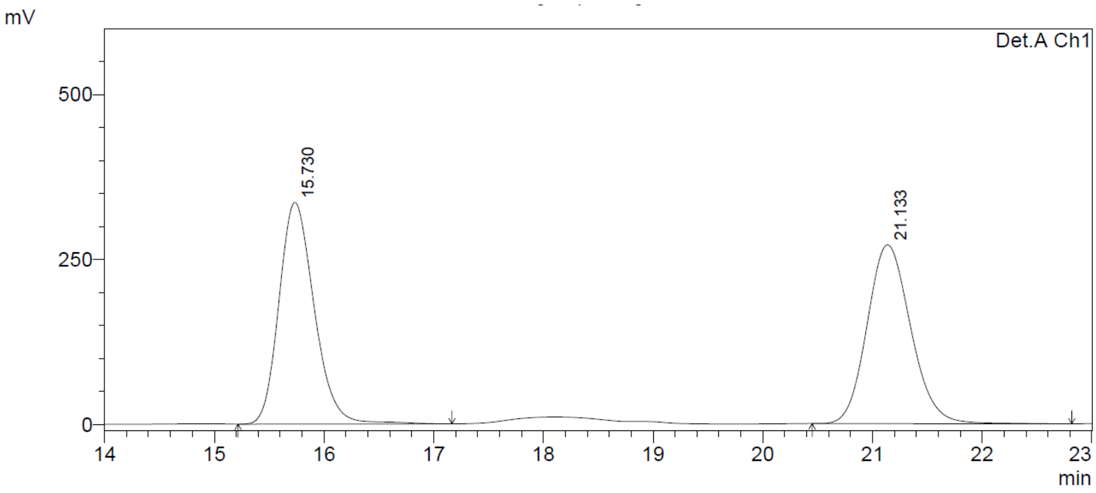

Chiral:

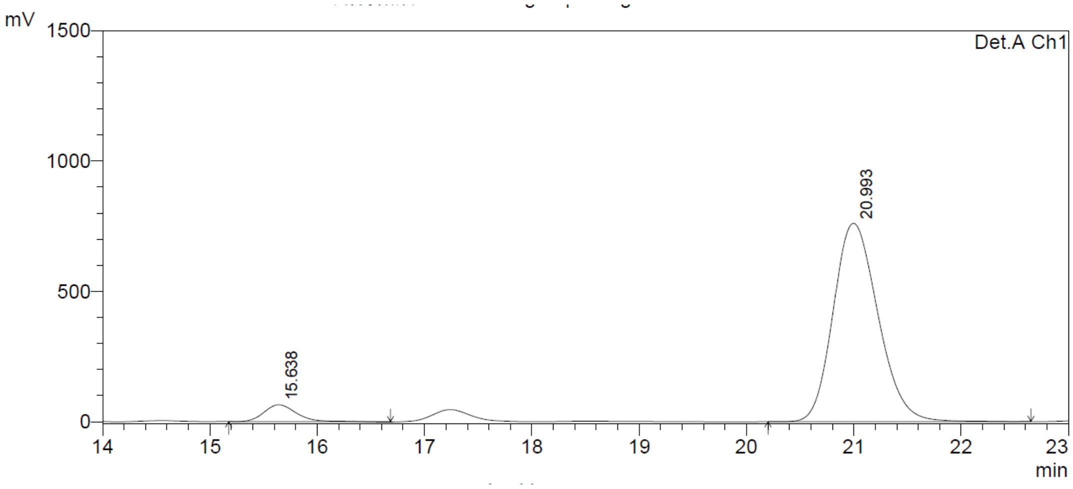

Detector A Ch1 254nm

Peak#	Ret. Time	Area	Area%
1	15.638	1358074	5.796
2	20.993	22071621	94.204
Total		23429695	100.000

Supplementary Fig. 88. HPLC spectra of compound **3ab**

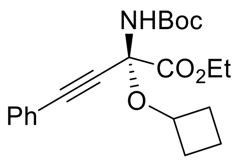

3ac

Racemate:

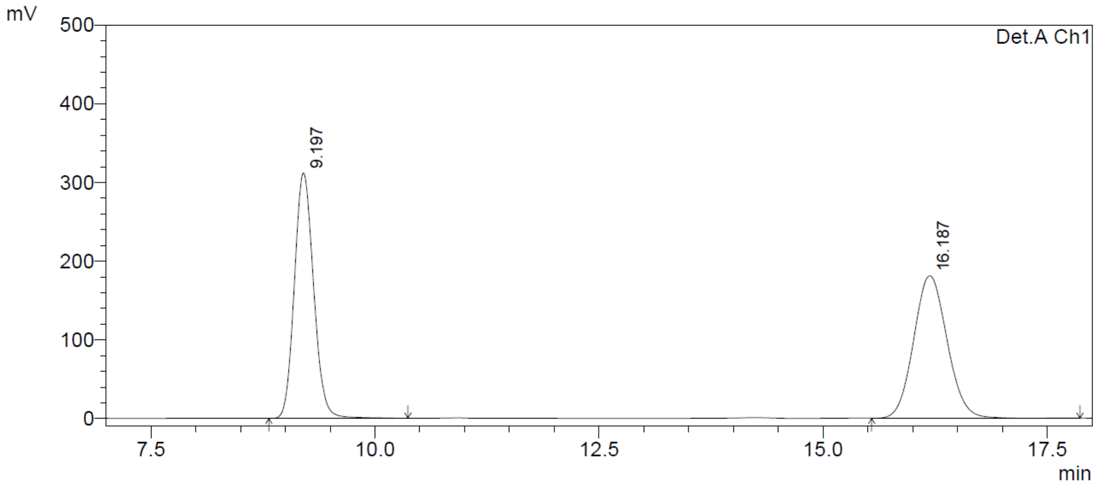

Chiral:

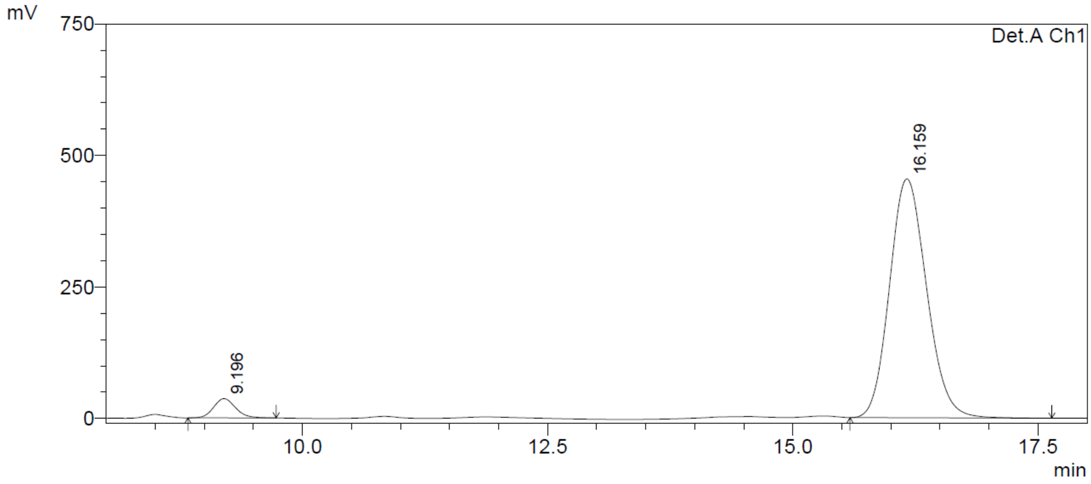

Detector A Ch1 254nm

Peak#	Ret. Time	Area	Area%
1	9.196	573390	4.580
2	16.159	11946235	95.420
Total		12519625	100.000

Supplementary Fig. 89. HPLC spectra of compound **3ac**

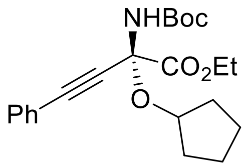

3ad

Racemate:

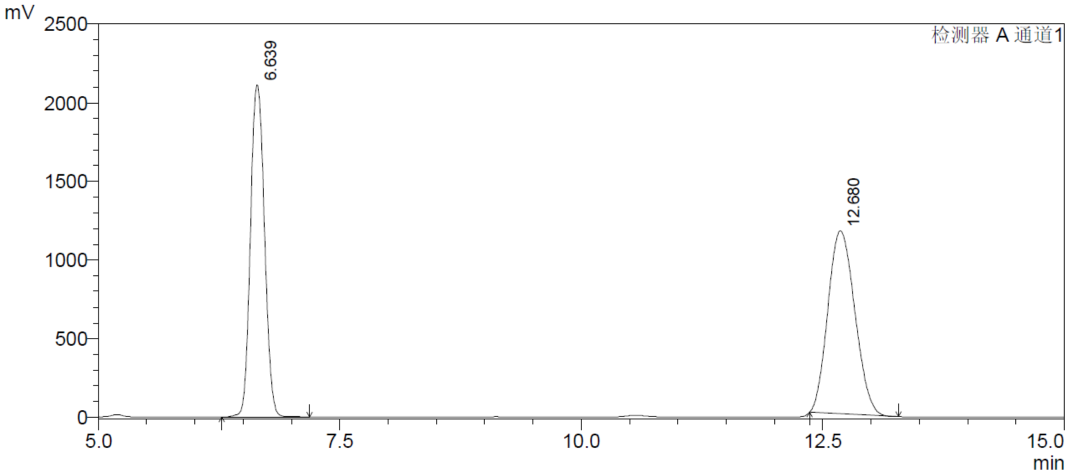

Chiral:

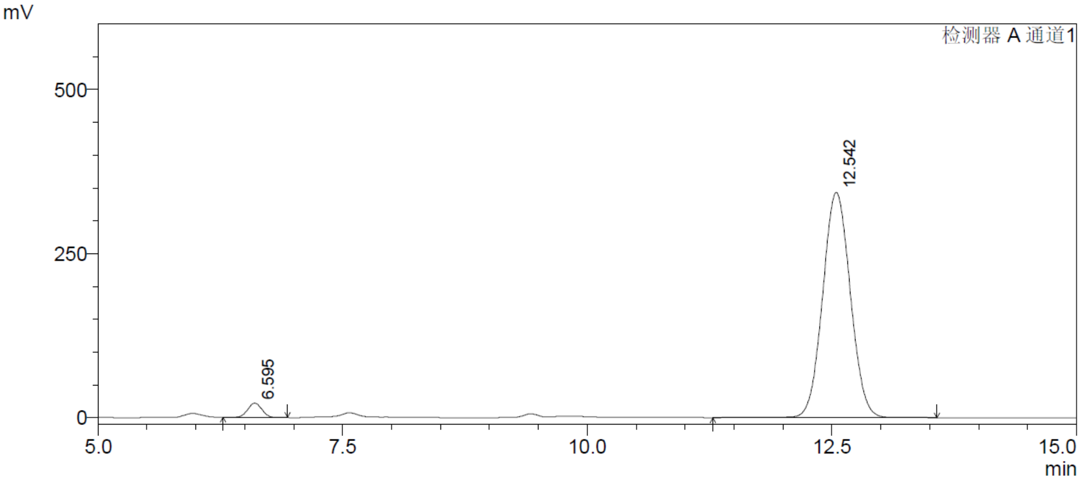

Detector A Ch1 254nm

Peak#	Ret. Time	Area	Area%
1	6.595	222525	3.191
2	12.542	6749931	96.809
Total		6972456	100.000

Supplementary Fig. 90. HPLC spectra of compound **3ad**

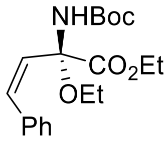

4

Racemate:

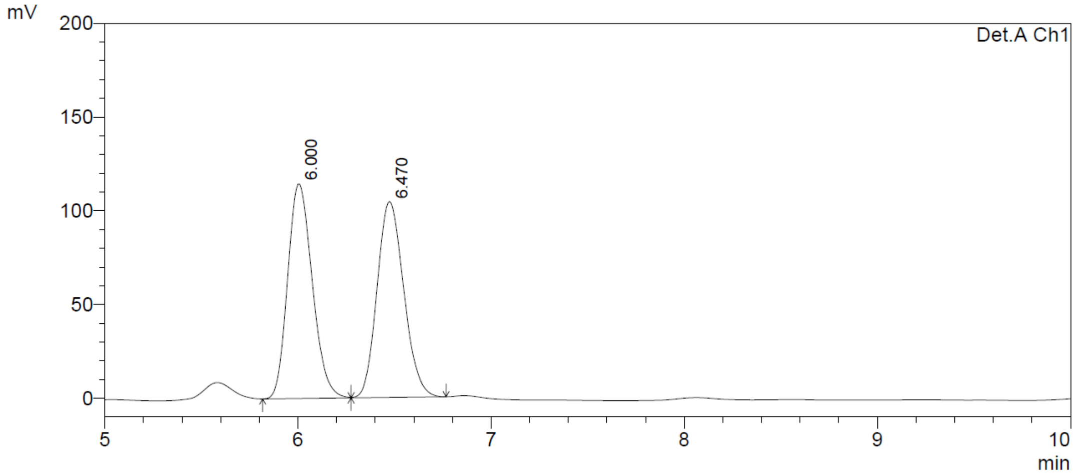

Chiral:

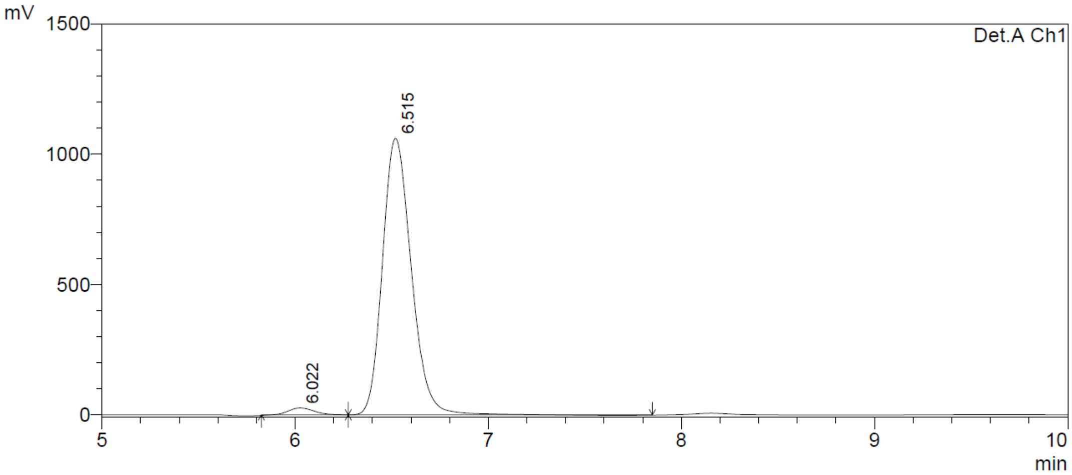

Detector A Ch1 220nm

Peak#	Ret. Time	Area	Area%
1	6.022	248869	2.207
2	6.515	11025207	97.793
Total		11274076	100.000

Supplementary Fig. 91. HPLC spectra of compound **4**

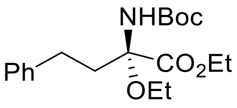

5

Racemate:

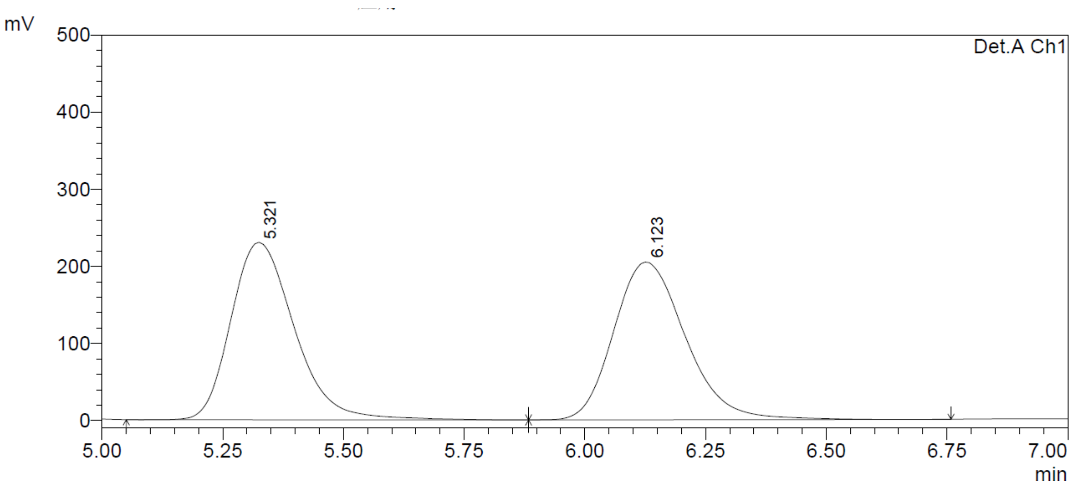

Chiral:

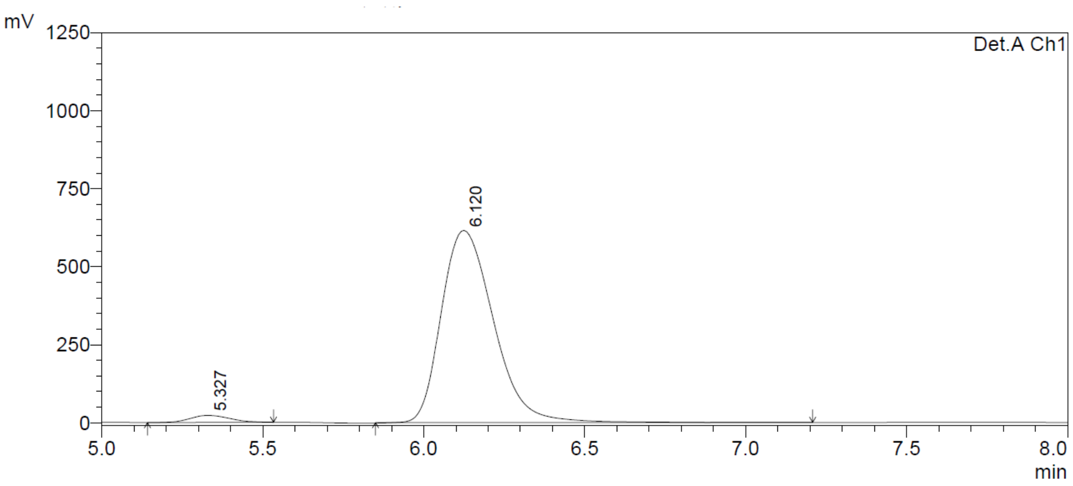

Detector A Ch1 220nm

Peak#	Ret. Time	Area	Area%
1	5.327	190066	2.667
2	6.120	6937765	97.333
Total		7127831	100.000

Supplementary Fig. 92. HPLC spectra of compound **5**

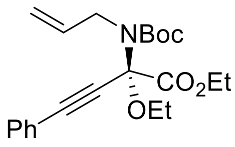

6

Racemate:

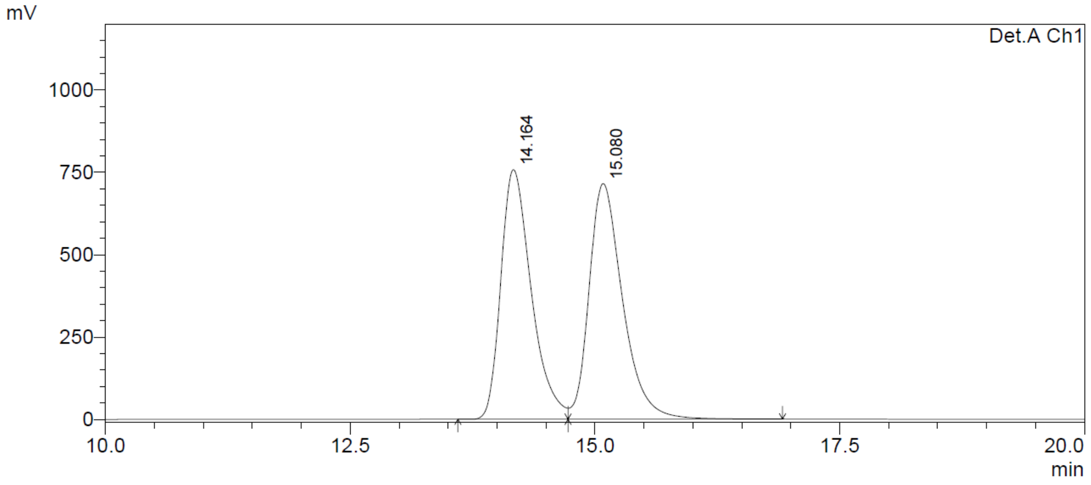

Chiral:

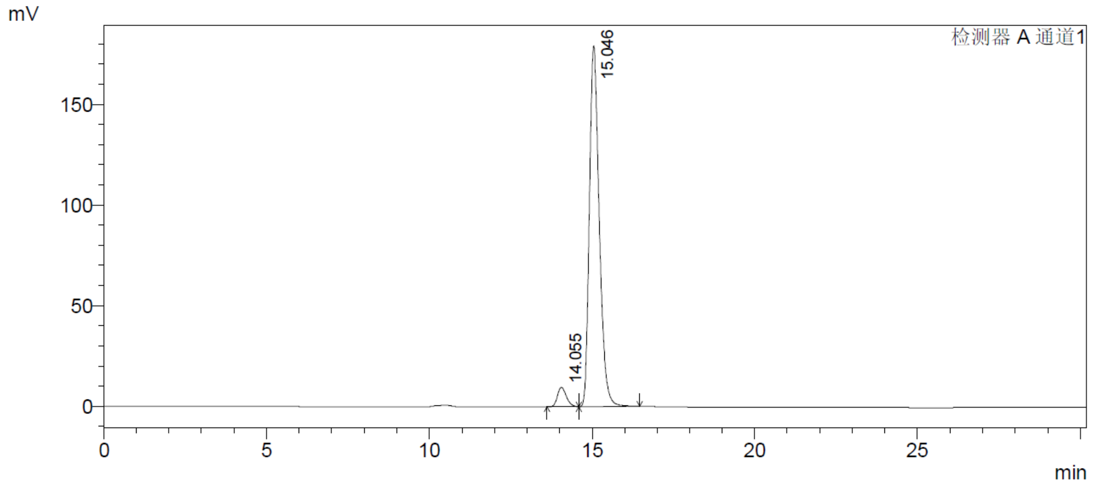

Detector A Ch1 220nm

Peak#	Ret. Time	Area	Area%
1	14.055	188216	4.750
2	15.046	3774116	95.250
Total		3962331	100.000

Supplementary Fig. 93. HPLC spectra of compound **6**

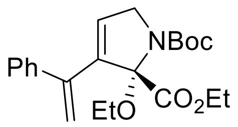

7

Racemate:

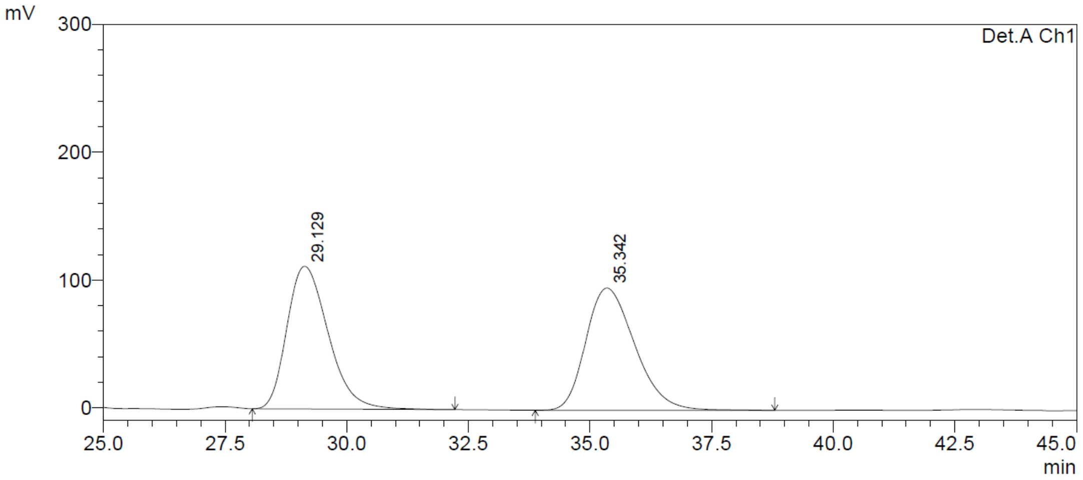

Chiral:

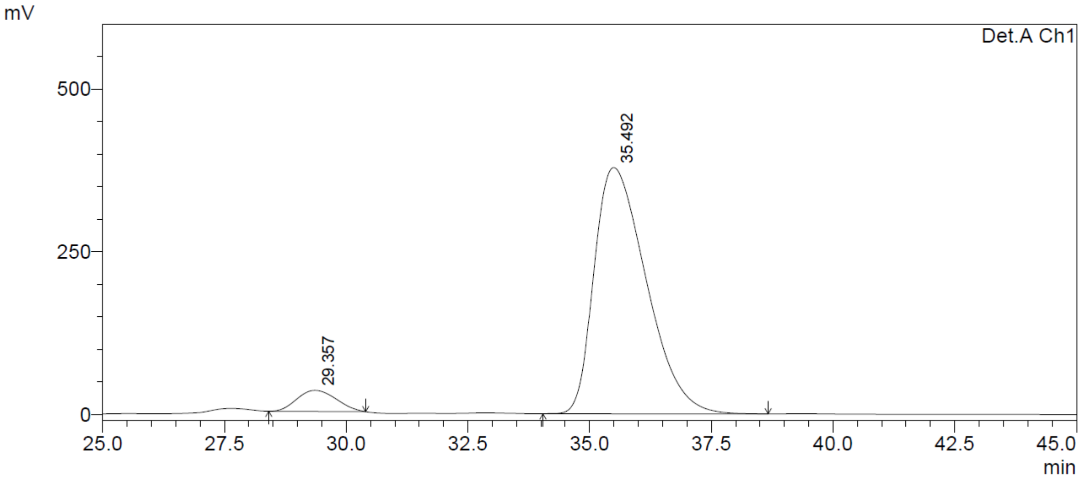

Detector A Ch1 220nm

Peak#	Ret. Time	Area	Area%
1	29.357	1821899	6.071
2	35.492	28189001	93.929
Total		30010899	100.000

Supplementary Fig. 94. HPLC spectra of compound **7**

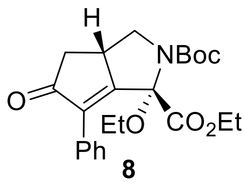

8

Racemate:

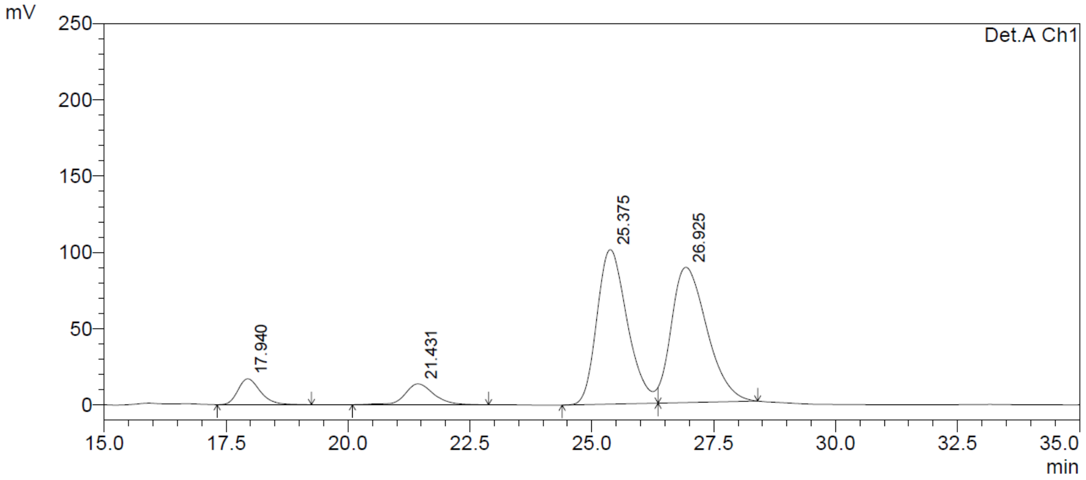

Chiral:

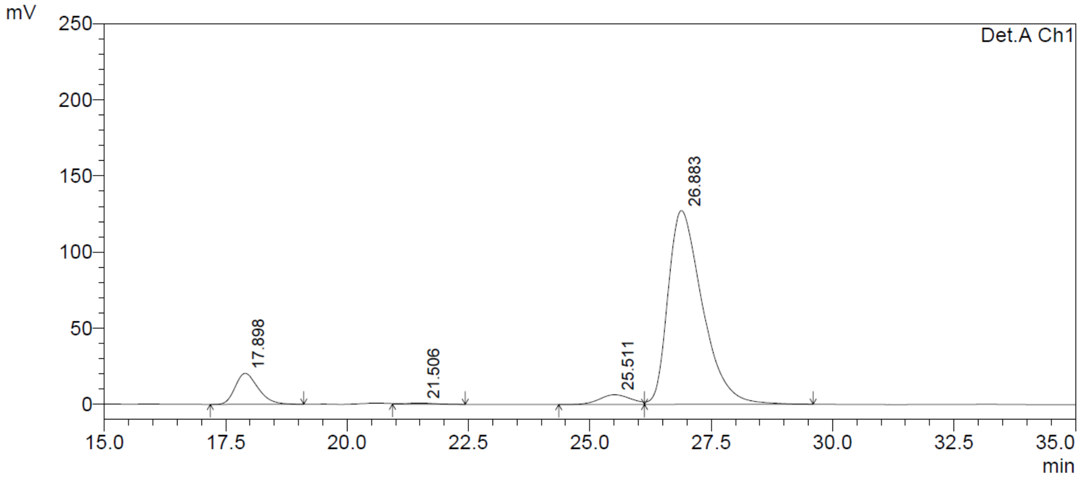

Detector A Ch1 220nm

Peak#	Ret. Time	Area	Area%
1	17.898	671404	9.255
2	21.506	28228	0.389
3	25.511	285057	3.929
4	26.883	6270181	86.427
Total		7254871	100.000

Supplementary Fig. 95. HPLC spectra of compound **8**

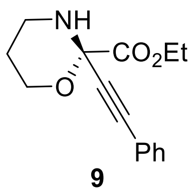

9

Racemate:

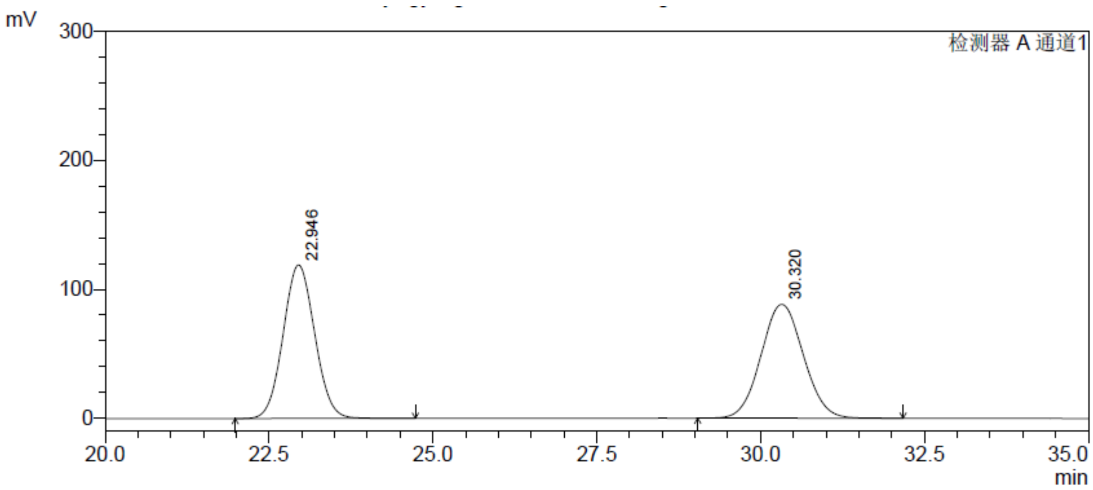

Chiral:

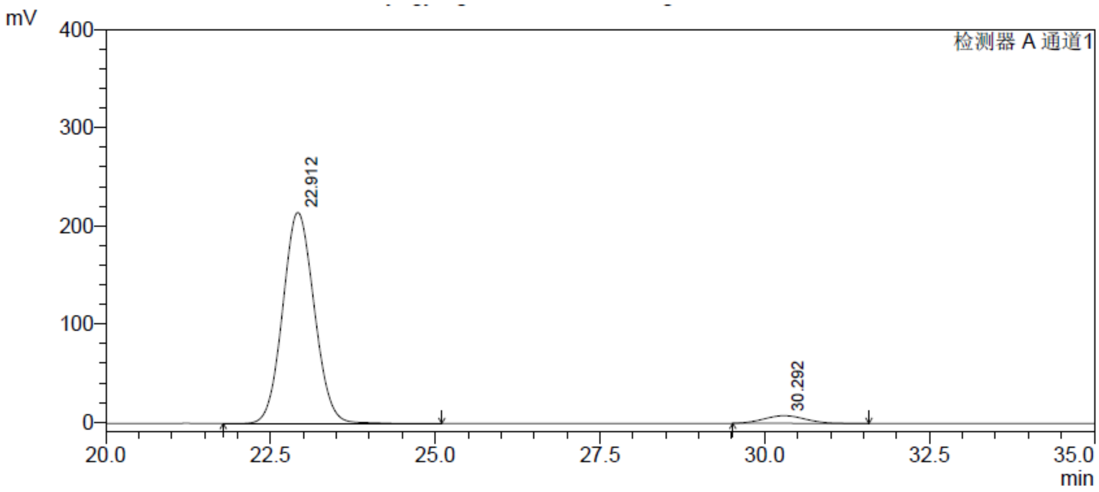

Detector A Ch1 220nm

Peak#	Ret. Time	Area	Area%
1	22.912	7159802	95.464
2	30.292	340225	4.536
Total		7500027	100.000

Supplementary Fig. 96. HPLC spectra of compound **9**

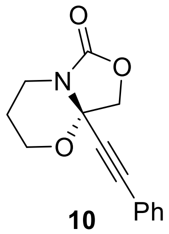

10

Racemate:

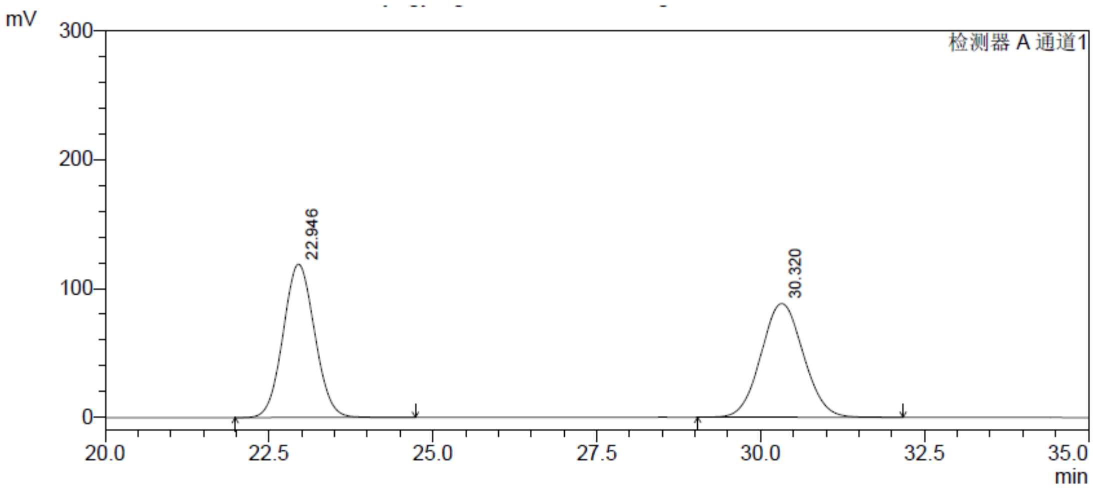

Chiral:

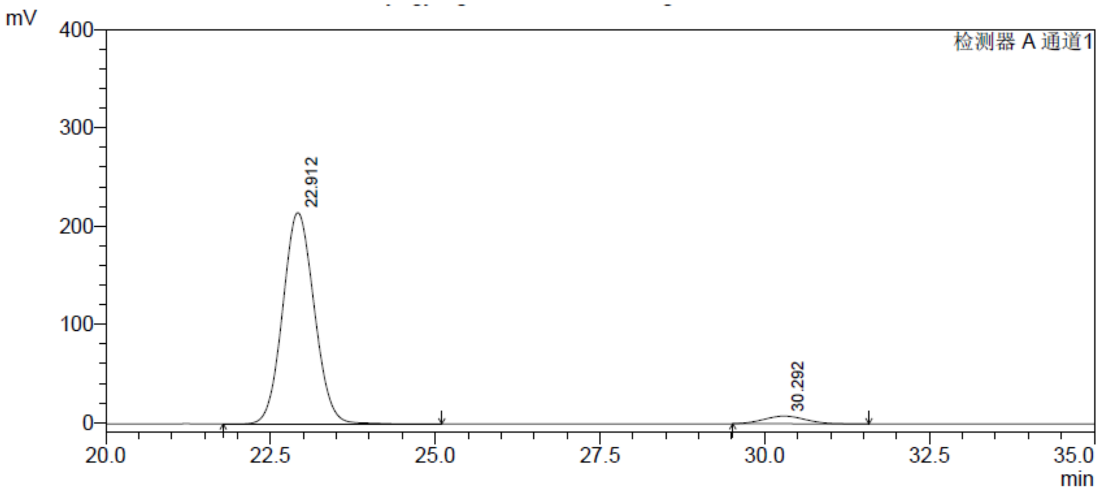

Detector A Ch1 220nm

Peak#	Ret. Time	Area	Area%
1	22.912	7159802	95.464
2	30.292	340225	4.536
Total		7500027	100.000

Supplementary Fig. 97. HPLC spectra of compound **10**

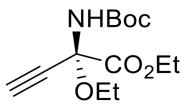

11

Racemate:

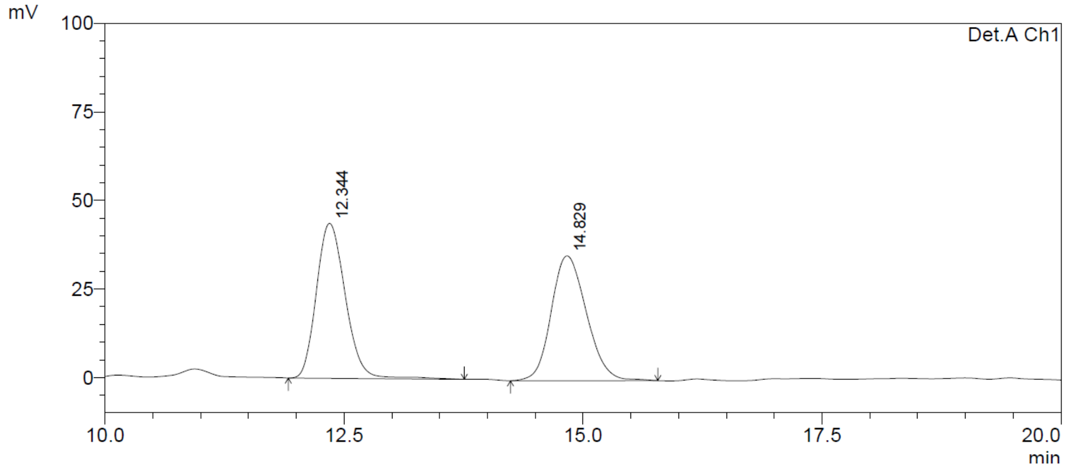

Chiral:

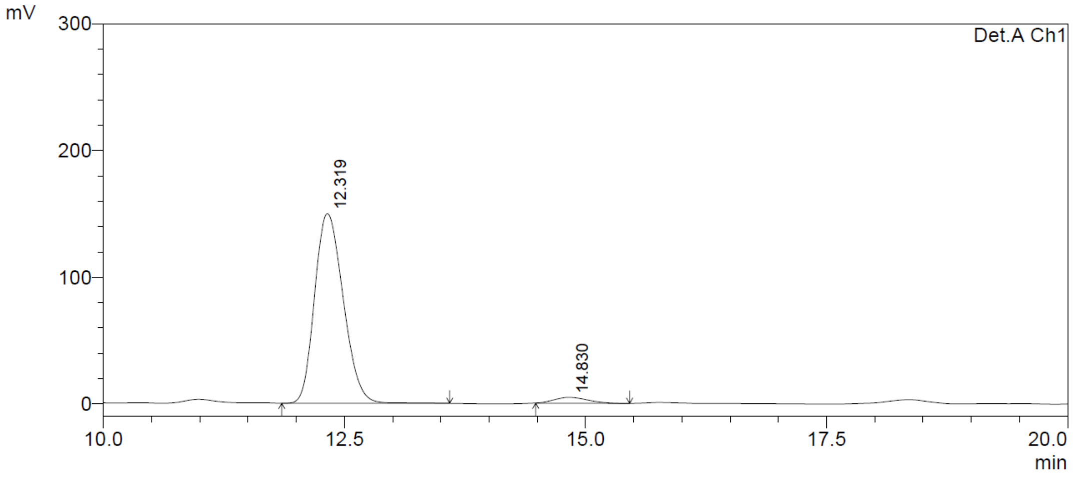

Detector A Ch1 220nm

Peak#	Ret. Time	Area	Area%
1	12.319	3090965	96.516
2	14.830	111584	3.484
Total		3202549	100.000

Supplementary Fig. 98. HPLC spectra of compound **11**

Supplementary References

1. Guo, M.; Li, D.; Zhang, Z., Novel Synthesis of 2-Oxo-3-butynoates by Copper-Catalyzed Cross-Coupling Reaction of Terminal Alkynes and Monooxalyl Chloride. *J. Org. Chem.,* **68**, 10172-10174, (2003).

2. Yang, J.; Wang, Z.; He, Z.; Li, G.; Hong, L.; Sun, W.; Wang, R., Organocatalytic Enantioselective Synthesis of Tetrasubstituted α-Amino Allenoates by Dearomative γ-Addition of 2,3-Disubstituted Indoles to β,γ-Alkynyl-α-imino Esters. *Angew. Chem. Int. Ed.,* **59**, 642-647, (2020).

3. Hatano, M.; Yamashita, K.; Mizuno, M.; Ito, O.; Ishihara, K., C-Selective and Diastereoselective Alkyl Addition to β,γ-Alkynyl-α-imino Esters with Zinc(II)ate Complexes. *Angew. Chem. Int. Ed.,* **54**, 2707-2711, (2015).

4. Akullian, L. C.; Snapper, M. L.; Hoveyda, A. H., Three-Component Enantioselective Synthesis of Propargylamines through Zr-Catalyzed Additions of Alkyl Zinc Reagents to Alkynylimines. *Angew. Chem. Int. Ed.,* **42**, 4244-4247, (2003).

5. Raikar, S. N.; Malinakova, H. C., Divergent Reaction Pathways of Homologous and Isosteric Propargyl Amides in Sequential Ru/Pd-Catalyzed Annulations for the Synthesis of Heterocycles. *J. Org. Chem.,* **78**, 3832-3846, (2013).

6. Koradin, C.; Polborn, K.; Knochel, P., Enantioselective Synthesis of Propargylamines by Copper-Catalyzed Addition of Alkynes to Enamines. *Angew. Chem. Int. Ed.,* **41**, 2535-2538, (2002).

7. Guo, W.; Zuo, L.; Cui, M.; Yan, B.; Ni, S., Propargylic Amination Enabled the Access to Enantioenriched Acyclic α-Quaternary α-Amino Ketones. *J. Am. Chem. Soc.,* **143**, 7629-7634, (2021).

8. Adamo, C.; Barone, V., Toward reliable density functional methods without adjustable parameters: The PBE0 model. *J. Chem. Phys.,* **110**, 6158-6170, (1999).

9. (a) Grimme, S.; Ehrlich, S.; Goerigk, L., Effect of the damping function in dispersion corrected density functional theory. *J. Comput. Chem.,* **32**, 1456-1465, (2011); (b) Grimme, S.; Antony, J.; Ehrlich, S.; Krieg, H., A consistent and accurate ab initio parametrization of density functional dispersion correction (DFT-D) for the 94 elements H-Pu. *J. Chem. Phys.,* **132**, 154104, (2010).

10. (a) Balabanov, N. B.; Peterson, K. A., Systematically convergent basis sets for transition metals. I. All-electron correlation consistent basis sets for the 3d elements Sc–Zn. *J. Chem. Phys.,* **123**, 064107, (2005); (b) Balabanov, N. B.; Peterson, K. A., Basis set limit electronic excitation energies, ionization potentials, and electron affinities for the 3d transition metal atoms: Coupled cluster and multireference methods. *J. Chem. Phys.,* **125**, 074110, (2006); (c) Jr., T. H. D., Gaussian basis sets for use in correlated molecular calculations. I. The atoms boron through neon and hydrogen. *J. Chem. Phys.,* **90**, 1007-1023, (1989).

11. Li, X.; Frisch, M. J., Energy-Represented Direct Inversion in the Iterative Subspace within a Hybrid Geometry Optimization Method. *J. Chem. Theory. Comput.,* **2**, 835-839, (2006).

12. Fukui, K., The path of chemical reactions - the IRC approach. *Acc. Chem. Res.,* **14**, 363-368, (1981).

13. Kashinski, D. O.; Chase, G. M.; Nelson, R. G.; Di Nallo, O. E.; Scales, A. N.; VanderLey, D. L.; Byrd, E. F. C., Harmonic Vibrational Frequencies: Approximate Global Scaling Factors

for TPSS, M06, and M11 Functional Families Using Several Common Basis Sets. *The Journal of Physical Chemistry A,* **121**, 2265-2273, **(**2017).
14. Grimme, S., Supramolecular Binding Thermodynamics by Dispersion-Corrected Density Functional Theory. *Chemistry – A European Journal.,* **18**, 9955-9964, **(**2012).
15. Lu, T.; Chen, Q., Shermo: A general code for calculating molecular thermochemistry properties. *Computational and Theoretical Chemistry,* 113249, **(**2021).
16. Helgaker, T.; Klopper, W.; Tew, D. P., Quantitative quantum chemistry. *Mol. Phys.,* **106**, 2107-2143, **(**2008).
17. Marenich, A. V.; Cramer, C. J.; Truhlar, D. G., Universal Solvation Model Based on Solute Electron Density and on a Continuum Model of the Solvent Defined by the Bulk Dielectric Constant and Atomic Surface Tensions. *The Journal of Physical Chemistry B,* **113**, 6378-6396, (2009).
